# Causal pathways in preeclampsia: a Mendelian randomization study in European populations

**DOI:** 10.3389/fendo.2024.1453277

**Published:** 2024-09-02

**Authors:** Zilong Tan, Mengdi Ding, Jianwu Shen, Yuxiao Huang, Junru Li, Aochuan Sun, Jing Hong, Yan Yang, Sheng He, Chao Pei, Ran Luo

**Affiliations:** ^1^ Department of Urology, Xiyuan Hospital, China Academy of Chinese Medical Sciences, Beijing, China; ^2^ Department of Urology, Qinghai Provincial Hospital of Traditional Chinese Medicine, Xining, China; ^3^ Department of Gynecology, Xiyuan Hospital, China Academy of Chinese Medical Sciences, Beijing, China; ^4^ Department of Internal Medicine, Qinghai Provincial Hospital of Traditional Chinese Medicine, Xining, China; ^5^ Department of Geriatrics, Xiyuan Hospital, China Academy of Chinese Medical Sciences, Beijing, China; ^6^ Department of Integration of Chinese and Western Medicine, Key Laboratory of Carcinogenesis and Translational Research, Peking University Cancer Hospital and Institute, Beijing, China; ^7^ Department of Critical Care Medicine, Jiangsu Provincial Hospital of Traditional Chinese Medicine, Nanjing, China; ^8^ The First Clinical Medical College of Anhui University of Traditional Chinese Medicine, Hefei, China; ^9^ Department of Ophthalmology, China Academy of Traditional Chinese Medicine Hospital of Ophthalmology, Beijing, China

**Keywords:** preeclampsia, Mendelian randomization, genetic determinants, causal associations, metabolic disorders, immune and inflammatory factors, serum uric acid levels

## Abstract

**Purpose:**

Our study utilizes Mendelian Randomization (MR) to explore the causal relationships between a range of risk factors and preeclampsia, a major contributor to maternal and perinatal morbidity and mortality.

**Methods:**

Employing the Inverse Variance Weighting (IVW) approach, we conducted a comprehensive multi-exposure MR study analyzing genetic variants linked to 25 risk factors including metabolic disorders, circulating lipid levels, immune and inflammatory responses, lifestyle choices, and bone metabolism. We applied rigorous statistical techniques such as sensitivity analyses, Cochran’s Q test, MR Egger regression, funnel plots, and leave-one-out sensitivity analysis to address potential biases like pleiotropy and population stratification.

**Results:**

Our analysis included 267,242 individuals, focusing on European ancestries and involving 2,355 patients with preeclampsia. We identified strong genetic associations linking increased preeclampsia risk with factors such as hyperthyroidism, BMI, type 2 diabetes, and elevated serum uric acid levels. Conversely, no significant causal links were found with gestational diabetes, total cholesterol, sleep duration, and bone mineral density, suggesting areas for further investigation. A notable finding was the causal relationship between systemic lupus erythematosus and increased preeclampsia risk, highlighting the significant role of immune and inflammatory responses.

**Conclusion:**

This extensive MR study sheds light on the complex etiology of preeclampsia, underscoring the causal impact of specific metabolic, lipid, immune, lifestyle, and bone metabolism factors. Our findings advocate for a multidimensional approach to better understand and manage preeclampsia, paving the way for future research to develop targeted preventive and therapeutic strategies.

## 1 Introduction

Preeclampsia is a leading complication in maternal health, characterized by hypertension and often proteinuria, presenting significant challenges in public health and clinical management (1, 2). Despite considerable advances in our understanding of maternal health (3, 4), the complex etiology of preeclampsia remains only partially elucidated, emphasizing the need for innovative research approaches to explore its underlying causes (5, 6).

Traditional investigative methods, such as randomized controlled trials (RCTs), face significant ethical and practical challenges in pregnancy-related research, often leaving gaps in causal understanding (7–9). Observational studies, while informative, are susceptible to confounding and reverse causation, which can obscure causal inferences (10–12).

Mendelian Randomization (MR) emerges as a pivotal tool in genetic epidemiology, using genetic variants as instrumental variables to infer causal relationships between risk factors and health outcomes (13, 14). This study employs the Inverse Variance Weighted (IVW) method of MR (15, 16) to dissect the causal relationships between a broad spectrum of risk factors—including metabolic disorders, lipid levels, immune responses, lifestyle factors, and bone metabolism—and the onset of preeclampsia. These risk factors were selected based on preliminary evidence suggesting their potential roles in the pathophysiology of preeclampsia. Specifically, metabolic disorders and lipid levels are implicated in endothelial dysfunction; immune responses are central to the inflammatory processes in preeclampsia; lifestyle factors contribute to overall maternal health and pregnancy outcomes; and bone metabolism reflects broader systemic changes during pregnancy. This genetic approach helps overcome the biases inherent in traditional observational studies and provides a deeper understanding of the disorder’s pathophysiology.

Our comprehensive analysis traverses multiple domains, revealing intricate associations that are crucial to understanding preeclampsia’s development and suggesting the involvement of diverse biological pathways. This study not only enhances our knowledge of preeclampsia but also opens avenues for early detection and targeted intervention strategies.

Furthermore, by addressing inherent limitations of MR, such as potential pleiotropy and population stratification, through advanced statistical methodologies and rigorous sensitivity analyses, we ensure the robustness of our findings. Grounded in a solid methodological framework and building on seminal works in the field, our research offers valuable insights into the causal mechanisms of preeclampsia. These findings advocate for a multifaceted approach to both the research and management of this complex condition, potentially guiding future research directions and improving clinical practices to enhance maternal and perinatal health outcomes.

## 2 Materials and methods

### 2.1 Data sources and availability

Our investigation into the genetic underpinnings of preeclampsia utilizes a multifaceted MR framework (17–19), drawing from extensive datasets primarily focused on European ancestry populations. This approach strategically mitigates potential confounding issues, particularly those related to population stratification, thus enhancing the accuracy and specificity of our causal assessments. We have conducted a comprehensive analysis across five primary exposure categories, crucial to understanding preeclampsia: metabolic disorders, lipid metabolism factors, immune and inflammatory factors, lifestyle variables, and bone metabolism, in addition to other potential contributors(a total of 25 exposure factors). Within each category, multiple phenotypes have been examined to elucidate their associations with preeclampsia. Key to our analysis is the incorporation of extensive Genome-Wide Association Study (GWAS) data. Notably, we leveraged the UK Biobank and FinnGen datasets, encompassing a total of 628,000 participants. This includes a detailed GWAS for preeclampsia involving 267,242 individuals, comprising 2,355 female cases of preeclampsia and a wide array of single nucleotide polymorphisms (24,165,538 Single Nucleotide Polymorphisms [SNPs]) (ID: ebi-a-GCST90018906). Furthermore, we augmented our genetic analysis with findings from Saori Sakaue et al.’s phenome-wide association study within the Japanese biobank, which identified approximately 5,000 novel loci across 179,000 individuals.

For metabolic factors, our MR analysis probed into type 1 diabetes (520,580 participants, 59,999,551 SNPs, ID: ebi-a-GCST90014023), type 2 diabetes (490,089 participants, 24,167,560 SNPs, ID: ebi-a-GCST90018926), gestational diabetes (123,579 participants, 16,379,784 SNPs, ID: finn-b-GEST_DIABETES), hyperthyroidism (460,499 participants, 24,189,279 SNPs, ID: ebi-a-GCST90018860), and BMI (461,460 participants, 9,851,867 SNPs, ID: ukb-b-19953). In the lipid metabolism domain, we scrutinized total cholesterol (437,878 participants, 4,232,052 SNPs, ID: ebi-a-GCST90025953), triglycerides (343,992 participants, 19,052,580 SNPs, ID: ebi-a-GCST90018975), and apolipoproteins. Immune and inflammatory conditions such as systemic lupus erythematosus (482,911 participants, 24,198,877 SNPs, ID: ebi-a-GCST90018917), gout (484,598 participants, 9,587,836 SNPs, ID: ebi-a-GCST90038687), and rheumatoid arthritis (484,598 participants, 9,587,836 SNPs, ID: ebi-a-GCST90038685) were also analyzed. Furthermore, we analyzed immune and inflammatory conditions, including systemic lupus erythematosus (482,911 participants), gout (484,598 participants), and rheumatoid arthritis (484,598 participants). Lifestyle factors like sleep duration and bone metabolism elements, including bone mineral density (365,403 participants, 10,783,906 SNPs), calcium levels (400,792 participants), and serum 25-Hydroxyvitamin D levels (496,946 participants), were also scrutinized.

**Figure 1:**
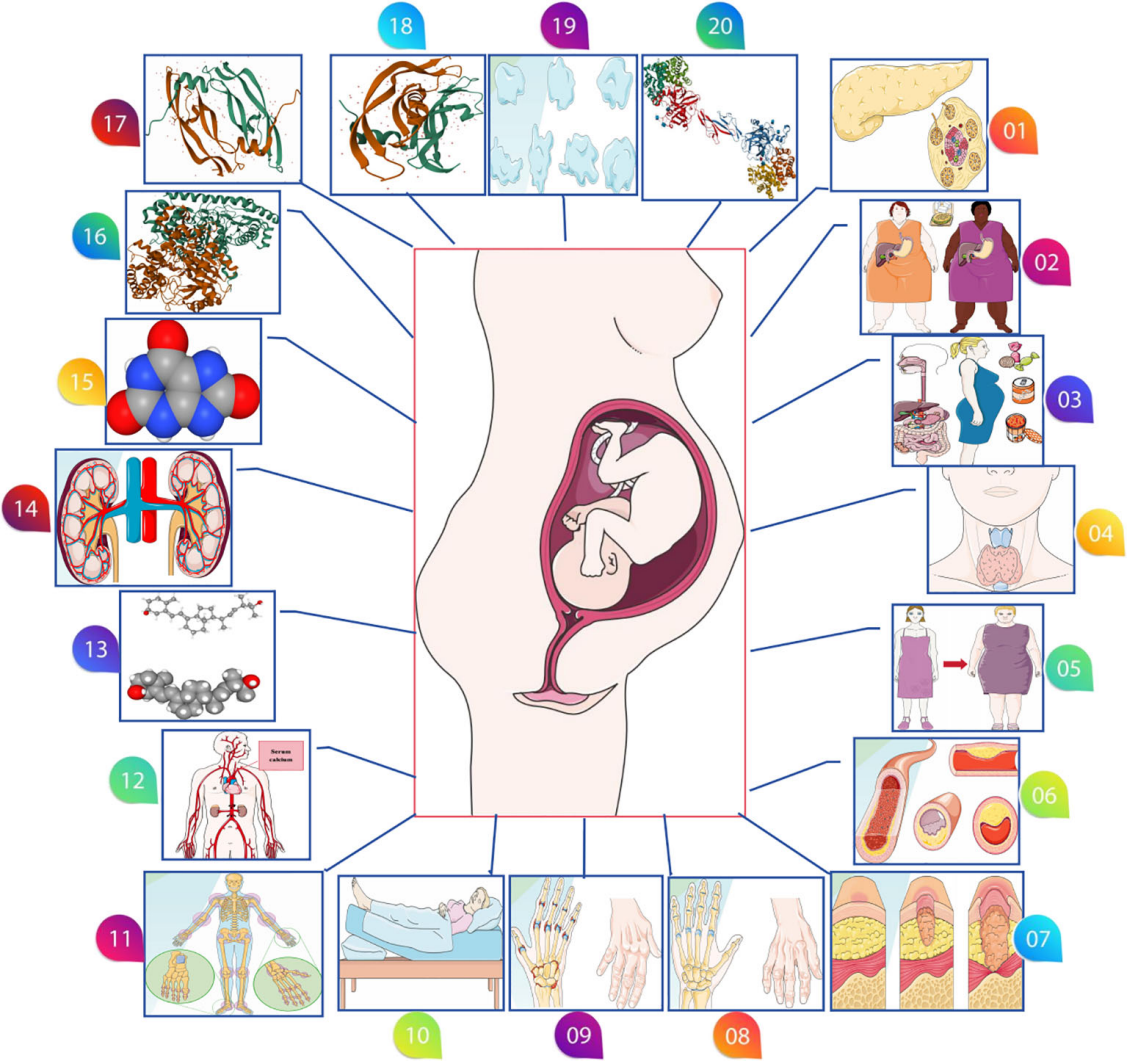
Various possible risk factors for preeclampsia. (01: Type 1 diabetes, 02: Type 2 diabetes 03: Gestational diabetes, 04: Hyperthyroidism, 05: Body mass index, 06: Circulating lipid metabolism factors, 07: Systemic lupus erythematosus, 08: Gout, 09: Rheumatoid arthritis, 10: Sleep duration, 11: Bone mineral density, 12: Calcium levels, 13: Serum 25-Hydroxyvitamin D levels, 14: Chronic kidney disease, 15: Serum uric acid levels, 16: Alanine aminotransferase levels, 17: Placental growth factor, 18: Vascular endothelial growth factor levels, 19: Platelet count, 20: Hemoglobin concentration).

Lastly, we investigated other potential exposure factors, including chronic kidney disease (117,165 participants, 2,179,497 SNPs), uric acid levels (343,836 participants, 19,041,286 SNPs), alanine aminotransferase levels (437,724 participants, 4,231,965 SNPs), placental growth factor (3,394 participants), vascular endothelial growth factor (21,758 participants), hemoglobin concentration (396,624 participants), and platelet count (600,968 participants). These additional factors provide a broader perspective on the genetic dimensions potentially influencing preeclampsia (**Figure 1**). The comprehensive GWAS summaries for each phenotype, crucial for our genetic analysis, are detailed in **Table 1**. For an in-depth statistical exploration, these datasets are accessible through the MRC Integrative Epidemiology Unit GWAS database (https://gwas.mrcieu.ac.uk/). This extensive and varied genetic data is pivotal in our exploration of the complex genetic landscape associated with the development of preeclampsia.

**Table 1:** Baseline characteristics of metabolic disorders, circulating lipid level factors, immune and inflammatory factors, lifestyle variables, bone metabolism, in addition to other potential factors (a total of 25 exposure factors) and Preeclampsia datasets.

Trait Contains		ID	Year	PMID	Population	Sample Size	n SNPs	n Case	n Control
**Metabolic factors**	**Type 1 diabetes**	**ebi-a-GCST90014023**	**2021**	**34012112**	**European**	**520,580**	**59,999,551**	**18,942**	**501,638**
	**Type 2 diabetes**	**ebi-a-GCST90018926**	**2021**	**34594039**	**European**	**490,089**	**24,167,560**	**38,841**	**451,248**
	**Gestational diabetes**	**finn-b-GEST_DIABETES**	**2021**	**NA**	**European**	**123,579**	**16,379,784**	**5,687**	**117,892**
	**Hyperthyroidism**	**ebi-a-GCST90018860**	**2021**	**34594039**	**European**	**460,499**	**24,189,279**	**3,557**	**456,942**
	**Body mass index (BMI)**	**ukb-b-19953**	**2018**	**32042192**	**European**	**461,460**	**9,851,867**	**NA**	**NA**
**Circulating lipid level factors**	**Total cholesterol levels**	**ebi-a-GCST90025953**	**2021**	**34226706**	**European**	**437,878**	**4,232,052**	**NA**	**NA**
	**Triglycerides**	**ebi-a-GCST90018975**	**2021**	**34594039**	**European**	**343,992**	**19,052,580**	**NA**	**NA**
	**HDL cholesterol levels**	**ebi-a-GCST90025956**	**2021**	**34226706**	**European**	**400,754**	**4,218,934**	**NA**	**NA**
	**LDL cholesterol levels**	**ebi-a-GCST90002412**	**2020**	**32493714**	**European**	**431,167**	**16,293,344**	**NA**	**NA**
	**Apolipoprotein A1 levels**	**ebi-a-GCST90025955**	**2021**	**34226706**	**European**	**398,508**	**4,218,115**	**NA**	**NA**
	**Apolipoprotein B levels**	**ebi-a-GCST90025952**	**2021**	**34226706**	**European**	**435,744**	**4,231,412**	**NA**	**NA**
**Immune and inflammatory factors**	**Systemic lupus erythematosus**	**ebi-a-GCST90018917**	**2021**	**34594039**	**European**	**482,911**	**24,198,877**	**647**	**482,264**
	**Gout**	**ebi-a-GCST90038687**	**2021**	**33959723**	**European**	**484,598**	**9,587,836**	**6,810**	**477,788**
	**Rheumatoid arthritis**	**ebi-a-GCST90038685**	**2021**	**33959723**	**European**	**484,598**	**9,587,836**	**5,427**	**479,171**
**Lifestyle and Bone metabolism factors**	**Sleep duration**	**ukb-b-4424**	**2018**	**NA**	**European**	**460,099**	**9,851,867**	**NA**	**NA**
	**Bone mineral density**	**ebi-a-GCST90014022**	**2021**	**34017140**	**European**	**365,403**	**10,783,906**	**NA**	**NA**
	**Calcium levels**	**ebi-a-GCST90025990**	**2021**	**34226706**	**European**	**400,792**	**4,218,949**	**NA**	**NA**
	**Serum 25-Hydroxyvitamin D levels**	**ebi-a-GCST90000618**	**2020**	**32242144**	**European**	**496,946**	**496,946**	**NA**	**NA**
**Other potential factors**	**Chronic kidney disease**	**ebi-a-GCST003374**	**2016**	**26831199**	**European**	**117,165**	**2,179,497**	**12,385**	**104,780**
	**Serum uric acid levels**	**ebi-a-GCST90018977**	**2021**	**34594039**	**European**	**343,836**	**19,041,286**	**NA**	**NA**
	**Alanine aminotransferase levels**	**ebi-a-GCST90025979**	**2021**	**34226706**	**European**	**437,724**	**4,231,965**	**NA**	**NA**
	**Placental growth factor**	**prot-b-66**	**2018**	**28369058**	**European**	**3,394**	**5,270,646**	**NA**	**NA**
	**Vascular endothelial growth factor levels**	**ebi-a-GCST90011995**	**2020**	**33067605**	**European**	**21,758**	**12,717,927**	**NA**	**NA**
	**Hemoglobin concentration**	**ebi-a-GCST90013978**	**2021**	**34017140**	**European**	**396,624**	**10,783,698**	**NA**	**NA**
	**Platelet count**	**ebi-a-GCST90028999**	**2018**	**29892013**	**European**	**600,968**	**11,973,076**	**NA**	**NA**
**Outcome factors**	**Preeclampsia**	**ebi-a-GCST90018906**	**2021**	**34594039**	**European**	**267,242**	**24,165,538**	**2,355**	**264,887**

NA, Not Applicable.

### 2.2 Selection of genetic instruments and data harmonization

**Figure 2:**
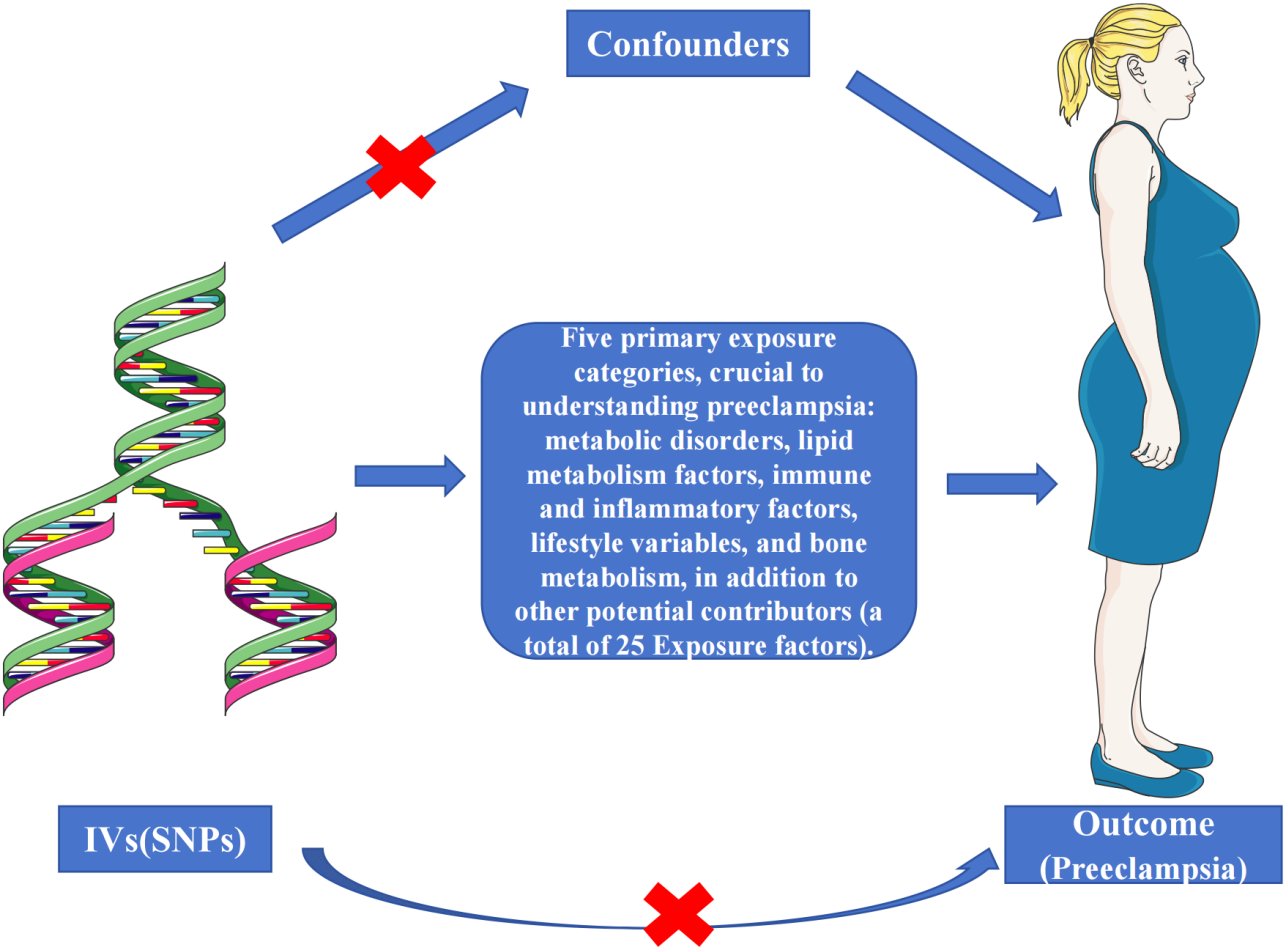
Schematic representation of the three assumptions and study design. (1) The selected genetic instrumental variables (IVs) are robustly associated with the exposure, ensuring a reliable link; (2) These IVs demonstrate no connections to potential confounding factors, safeguarding against bias; (3) The IVs exclusively affect the outcome risk via the exposure in a dependent manner, maintaining the integrity of the causal pathway.

**Figure 3:**
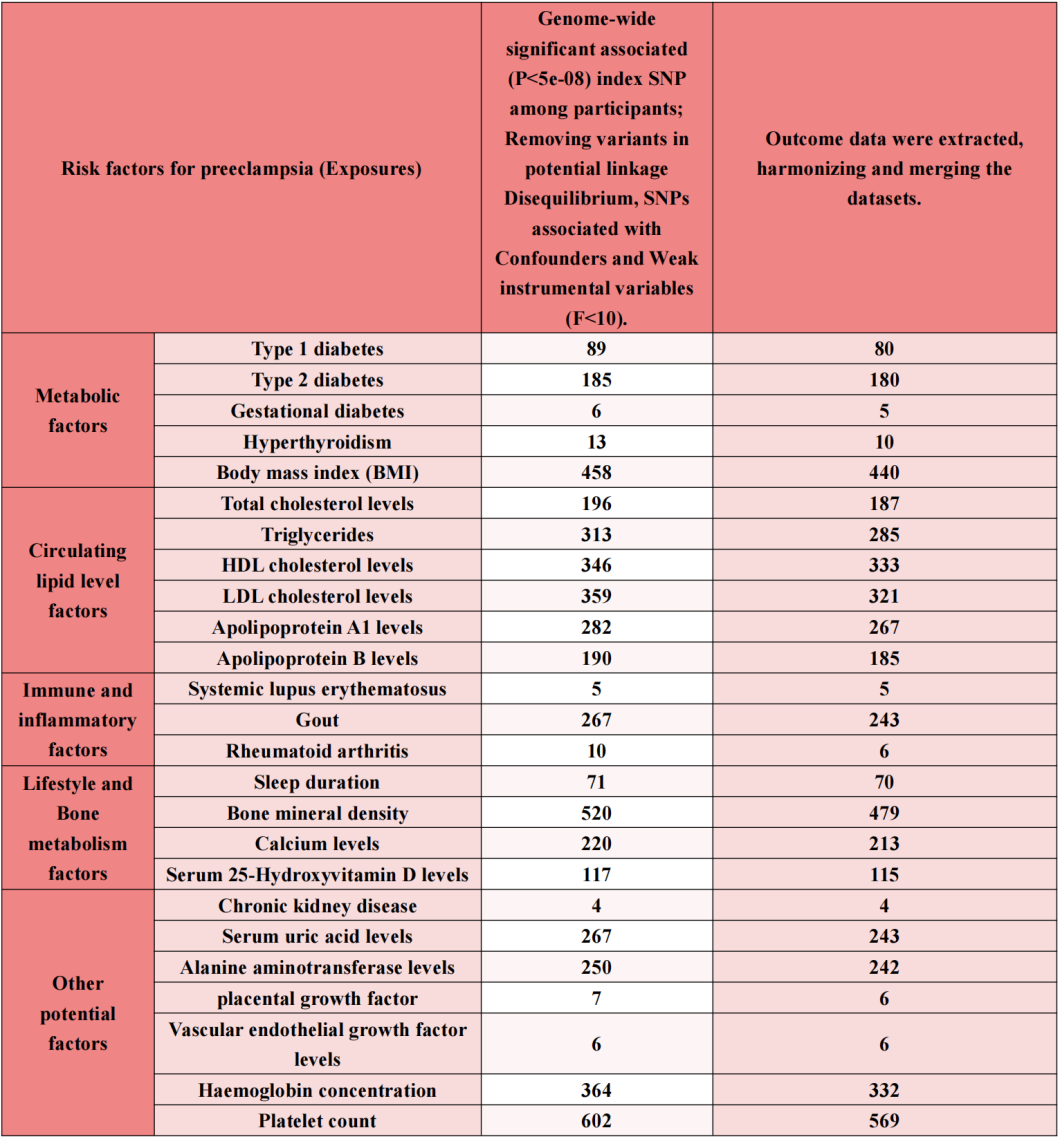
Genetic instrument selection of single-variable Mendelian randomization study.

Our Multi-exposure MR analysis hinged on the systematic identification of independent SNPs associated with exposure factors. We employed a stringent testing process for three critical hypotheses to validate these SNPs as reliable instrumental variables on a genome-wide scale (**Figure 2**), adhering to strict significance thresholds (P<5e-08). During this phase, we meticulously excluded SNPs with potential confounding effects on the outcome variables. The selection process involved a stringent linkage disequilibrium (LD) criterion (r^2^ < 0.001 over 10,000 kilobase pairs), utilizing the LD reference panel from the European superpopulation of the 1000 Genomes Project. This criterion was particularly focused on two-allele SNPs with minor allele frequencies above 0.01, enhancing the independence and relevance of our genetic instruments. We then extracted and harmonized summary-level data from diverse GWAS datasets, aligning key details such as SNP effects, allele frequencies, sample sizes, and statistical measures. This harmonization was critical to ensure the precise alignment of genetic variant association estimates across the exposure and outcome datasets. Where specific SNPs were missing in the outcome dataset, we identified and employed suitable surrogate SNPs, maintaining the analytical integrity of our study. Post-harmonization, we applied a rigorous criterion for the selection of instrumental variables, requiring an F-statistic exceeding 10. This threshold was instrumental in bolstering the validity of our findings and minimizing bias, marking a significant advancement in the application of MR methodologies. Detailed information on the SNPs used as instruments, including proxies for unavailable SNPs in the outcome dataset, is comprehensively documented in **Figure 3** and **Supplementary Tables S1**-**S25**. These resources exemplify our commitment to methodological transparency and robustness, offering valuable contributions to the field of genetic epidemiology.

### 2.3 Statistical analyses

The Multi-exposure MR analysis was conducted using TwoSampleMR version 0.5.7 (https://github.com/MRCIEU/TwoSampleMR) within the R 4.2.3 environment. In our pursuit of evaluating causality, we employed a comprehensive suite of five MR analysis methods. The primary method employed was the IVW MR with multiplicative random effects (20).To fortify the robustness of our findings against potential violations of MR assumptions, we supplemented the IVW method with four additional methodologies: MR Egger, Weighted Median, Simple Mode, and Weighted Mode. The IVW method, our central approach, assigned weights to each ratio based on their standard errors (SE), adeptly addressing potential heterogeneity in measurements and ensuring reliable estimates even across diverse data sources (21, 22).Concurrently, with these five robust MR analysis methods, we conducted supplementary sensitivity analyses. Initially, we assessed the presence of heterogeneity among variable-specific causal estimates using Cochran’s Q test (23, 24). This, in turn, was utilized to detect and adjust for pleiotropy through MR-Egger regression, determining whether directional level pleiotropy directly influenced the outcome (25, 26).Furthermore, we meticulously constructed funnel plots to visualize the precision of each variable-specific causal estimate in relation to the estimates themselves. These plots were designed to reflect symmetry in the context of IVW estimates, with more precise estimates indicative of reduced variability (21). Lastly, leave-one-out sensitivity analyses were executed to identify and assess influential data points within each instrumental variable (27). This technique was instrumental in uncovering the dependency of causal effect estimates on individual genetic variants, thus reinforcing the overall validity and robustness of our MR analysis outcomes.

## 3 Results

### 3.1 Unveiling causal connections between metabolic risk factors and preeclampsia

**Figure 4:**
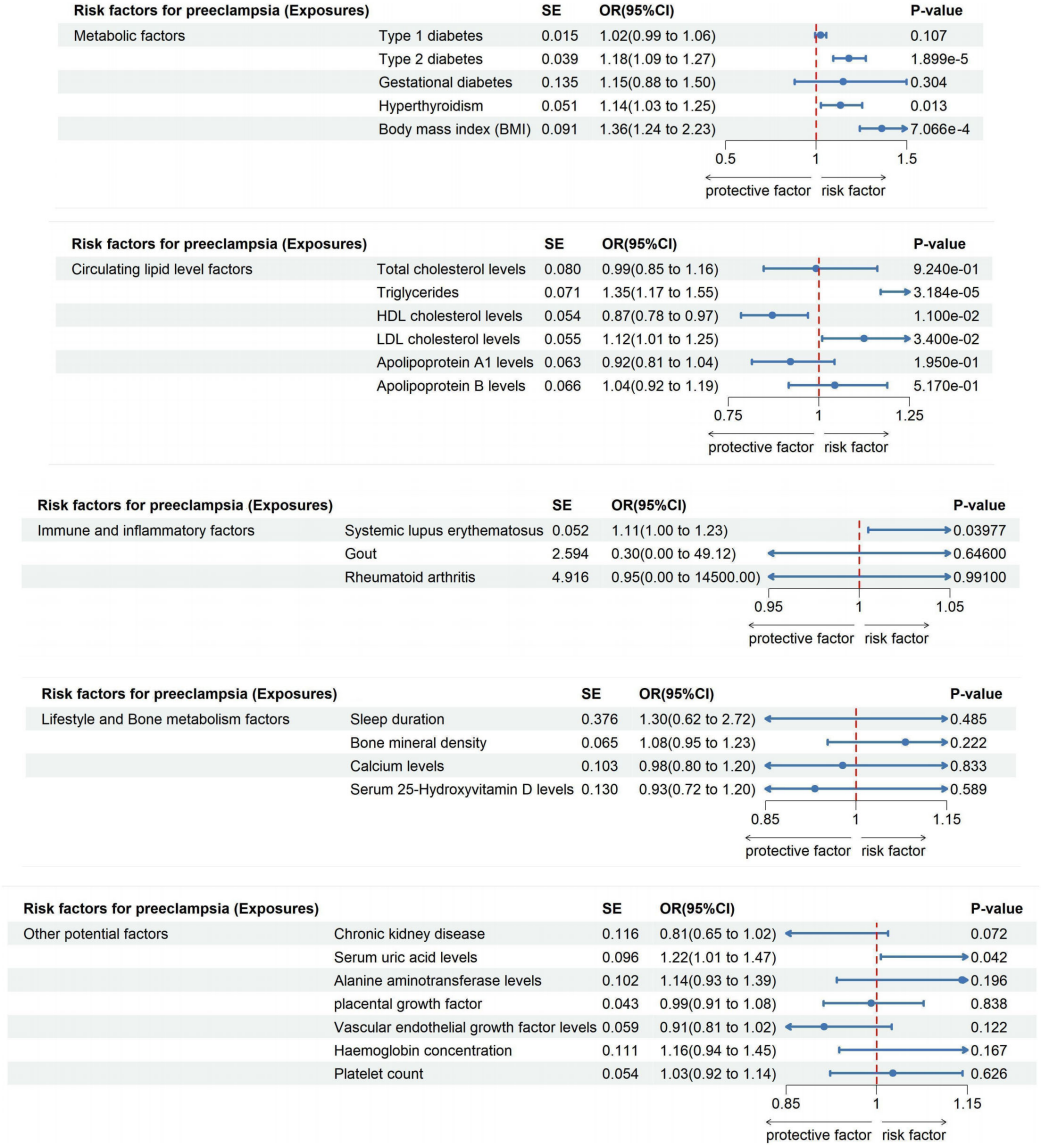
The impact of exposure factors on the risk of preeclampsia was assessed through MR analysis utilizing the IVW model.

In our examination of the complex interplay between metabolic risk factors and Preeclampsia, we employed the IVW method within Mendelian Randomization to ensure rigorous analysis (**Figure 4**). Our findings revealed that Type 1 diabetes does not significantly influence Preeclampsia risk (Odds Ratio, OR: 1.025; 95% CI: 0.995-1.056, P=0.107). This lack of association was consistent across various statistical methods, as meticulously detailed in **Supplementary Table S26**, and corroborated by the heterogeneity analysis (Q=96.581, P=0.065; **Table 2**) and MR-Egger regression (Egger intercept = 0.001, P=0.858; **Table 3**), with visual supports provided in **Supplementary Figures S2**, **S3**.

**Table 2:** Cochran’s Q tests for heterogeneity from MR-IVW analyses.

Exposure		Q	df	P-value
**Metabolic factors**	**Type 1 diabetes**	**96.581**	**77**	**0.065**
	**Type 2 diabetes**	**176.845**	**170**	**0.344**
	**Gestational diabetes**	**16.770**	**4**	**0.002**
	**Hyperthyroidism**	**4.486**	**8**	**0.811**
	**Body mass index (BMI)**	**423.955**	**417**	**0.396**
**Circulating lipid metabolism factors**	**Total cholesterol levels**	**222.723**	**185**	**0.030**
	**Triglycerides**	**276.256**	**272**	**0.417**
	**HDL cholesterol levels**	**334.400**	**324**	**0.333**
	**LDL cholesterol levels**	**302.701**	**312**	**0.637**
	**Apolipoprotein A1 levels**	**290.202**	**260**	**0.096**
	**Apolipoprotein B levels**	**220.763**	**182**	**0.026**
**Immune and inflammatory factors**	**Systemic lupus erythematosus**	**7.512**	**4**	**0.111**
	**Gout**	**37.458**	**26**	**0.068**
	**Rheumatoid arthritis**	**4.380**	**5**	**0.496**
**Lifestyle and Bone metabolism factors**	**Sleep duration**	**76.946**	**66**	**0.168**
	**Bone mineral density**	**426.393**	**406**	**0.234**
	**Calcium levels**	**267.586**	**210**	**0.004**
	**Serum 25-Hydroxyvitamin D levels**	**125.080**	**113**	**0.206**
**Other unidentified factors**	**Chronic kidney disease**	**1.104**	**3**	**0.776**
	**Serum uric acid levels**	**267.320**	**234**	**0.066**
	**Alanine aminotransferase levels**	**296.922**	**235**	**0.004**
	**Placental growth factor**	**3.729**	**5**	**0.589**
	**Vascular endothelial growth factor levels**	**2.865**	**4**	**0.581**
	**Haemoglobin concentration**	**292.159**	**279**	**0.282**
	**Platelet count**	**626.886**	**550**	**0.013**

**Table 3:** MR-Egger intercept tests for horizontal pleiotropy.

Exposure		Egger intercept	SE	P-value
Metabolic factors	Type 1 diabetes	0.001	0.005	0.858
	Type 2 diabetes	0.002	0.006	0.772
	Gestational diabetes	-0.051	0.096	0.630
	Hyperthyroidism	-0.028	0.026	0.320
	Body mass index (BMI)	-0.008	0.004	0.081
Circulating lipid metabolism factors	Total cholesterol levels	-0.005	0.004	0.245
	Triglycerides	0.007	0.003	0.030
	HDL cholesterol levels	-0.002	0.003	0.549
	LDL cholesterol levels	-0.002	0.008	0.449
	Apolipoprotein A1 levels	0.001	0.003	0.741
	Apolipoprotein B levels	-0.005	0.004	0.150
Immune and inflammatory factors	Systemic lupus erythematosus	0.196	0.075	0.079
	Gout	0.010	0.013	0.450
	Rheumatoid arthritis	0.005	0.034	0.884
Lifestyle and Bone metabolism factors	Sleep duration	-0.007	0.0177	0.697
	Bone mineral density	0.001	0.003	0.868
	Calcium levels	-0.003	0.005	0.500
	Serum 25-Hydroxyvitamin D levels	0.001	0.006	0.923
Other unidentified factors	Chronic kidney disease	-0.047	0.055	0.481
	Serum uric acid levels	-0.003	0.004	0.437
	Alanine aminotransferase levels	0.005	0.005	0.297
	Placental growth factor	0.031	0.034	0.414
	Vascular endothelial growth factor levels	-0.011	0.025	0.694
	Hemoglobin concentration	0.005	0.004	0.230
	Platelet count	0.002	0.003	0.471

Conversely, Type 2 diabetes demonstrated a clear causal link with an increased risk of Preeclampsia (OR: 1.181; 95% CI: 1.094-1.275, P=1.899e-05), a relationship that persisted in both Weighted Median and Weighted Mode analyses (**Supplementary Table S27**). This correlation translates to an 18.12% increase in risk per standard deviation increase in genetically predicted Type 2 diabetes, with robust findings supported by leave-one-out sensitivity analyses and funnel plots depicted in **Supplementary Figures S3**, **S4**.

Interestingly, no causal link was found between Gestational diabetes and Preeclampsia risk (OR: 1.149; 95% CI: 0.882-1.498, P=0.304; **Supplementary Table S28**), as consistent results across methodologies indicated no directional pleiotropic effects (MR-Egger intercept =-0.051, P=0.630), outlined in **Tables 2** and **3**. Furthermore, our analysis highlighted a significant association between Hyperthyroidism and increased Preeclampsia risk (OR: 1.135; 95% CI: 1.027-1.254; P = 0.013; **Supplementary Table S29**), with additional evidence from Weighted Median and Weighted Mode analyses supporting this finding (**Supplementary Figure S2**). Similarly, an elevated BMI significantly escalated the risk of Preeclampsia (OR: 1.362; 95% CI: 1.241-2.235; P=7.066e-4), with no excessive heterogeneity observed (Q=423.955, P=0.396; **Supplementary Table S30**), as illustrated in **Supplementary Figures S1**, **S2**.

### 3.2 Unveiling causal connections between circulating lipid level factors and preeclampsia

Employing the IVW Mendelian Randomization model, our analysis explored the impacts of various lipid levels on Preeclampsia risk. We found no significant causal association between total cholesterol levels and Preeclampsia (OR: 0.992; 95% CI: 0.848-1.161, P=0.924), with this finding consistently supported across multiple analytical methods as shown in **Supplementary Figures S1**, **S2**, and detailed in **Supplementary Table S31**. Notably, heterogeneity was present (Q=222.723, P=0.030; **Table 2**), but MR-Egger regression indicated no directional pleiotropic effects (Egger intercept =-0.005, P=0.245; **Table 3**). Our results’ robustness was further confirmed by leave-one-out sensitivity analyses and funnel plots (**Supplementary Figures S3**, **S4**).

In contrast, triglycerides were linked to an elevated risk of Preeclampsia, with the IVW analysis indicating a significant association (OR: 1.346; 95% CI: 1.170-1.549, P=3.184e-05; **Supplementary Table S32**, **Supplementary Figure S1**). For each standard deviation increase in genetically predicted triglyceride levels, Preeclampsia risk escalated by 34.62%. This association persisted across diverse analytical methods (**Supplementary Figure S2**) and showed no excessive heterogeneity (Q=276.256, P=0.417; **Table 2**). However, the presence of potential pleiotropy suggested by MR-Egger regression (Egger intercept=0.007, P=0.030; **Table 3**) necessitates cautious interpretation of these results.

Furthermore, High-Density Lipoprotein Cholesterol (HDL-C) demonstrated a protective effect, reducing the risk of developing Preeclampsia by 12.75% for each standard deviation increase (OR: 0.872; 95% CI: 0.785-0.970, P=0.011; **Supplementary Table S33**, **Supplementary Figure S1**). This consistent finding across methods (**Supplementary Figure S2**) was mirrored by the absence of directional pleiotropic effects in MR-Egger regression (Egger intercept =-0.002, P=0.549; **Table 3**). Conversely, Low-Density Lipoprotein Cholesterol (LDL-C) was associated with an increased Preeclampsia risk (OR: 1.125; 95% CI: 1.009-1.254, P=0.034; **Supplementary Table S34**, **Supplementary Figures S1**, **S2**), with the primary IVW analysis suggesting a 12.49% risk escalation for each standard deviation increase. MR-Egger regression confirmed the absence of pleiotropic effects influencing these results (Egger intercept =-0.002, P=0.449; **Table 3**). Lastly, analyses for Apolipoprotein A-1 (APOA-1) and Apolipoprotein B (APOB) revealed no significant causal links with Preeclampsia (APOA-1 OR: 0.922; 95% CI: 0.815-1.043, P=0.195; APOB OR: 1.044; 95% CI: 0.917-1.189, P=0.517), with these findings consistently supported by further analytical methods as detailed in **Supplementary Tables S35**, **S36**, and shown in **Supplementary Figure S2**.

### 3.3 Unveiling causal connections between immune and inflammatory factors and preeclampsia

Our comprehensive MR investigation explored the causal impact of immune and inflammatory factors on the risk of Preeclampsia, revealing notable distinctions among different conditions. Systemic lupus erythematosus was found to significantly increase the risk of Preeclampsia (OR: 1.114; 95% CI: 1.005-1.234; P = 0.039), as shown in **Supplementary Figure S1** and quantified in **Supplementary Table S37**. This positive correlation indicates an 11.35% escalation in Preeclampsia risk for each standard deviation increase in genetically inferred Systemic lupus erythematosus levels. The Cochran’s Q statistic confirmed minimal heterogeneity (Q = 7.512, P=0.111), and MR-Egger regression showed no significant directional effects among genetic variants (Egger intercept = 0.196, P = 0.079), supporting the validity of our results (**Supplementary Figures S3**, **S4**).

Conversely, no substantial causal relationships were identified for Gout or Rheumatoid arthritis. The IVW analysis for Gout showed a non-significant correlation with Preeclampsia risk (OR: 0.304; 95% CI: 0.00188 to 49.121; P=0.646; **Supplementary Figure S1**, **Supplementary Table S38**), and similar findings were observed for Rheumatoid arthritis, which displayed no positive association with Preeclampsia (OR: 0.947; 95% CI: 0.0000619 to 14500; P=0.991; **Supplementary Figure S1**, **Supplementary Table S39**). These outcomes suggest a lack of direct causal links between these conditions and Preeclampsia, further corroborated by multiple analytical approaches indicating robustness and stability of the interpretations (**Supplementary Figures S3**, **S4**).

### 3.4 Unveiling the influence of lifestyle and bone metabolism factors on preeclampsia risk

Our extensive MR analysis also assessed the potential influences of lifestyle and bone metabolism factors on Preeclampsia risk. Notably, no discernible association was found between sleep duration and Preeclampsia risk (OR: 1.300; 95% CI: 0.622-2.717, P=0.485), with consistent outcomes validated across different analytical methods (**Supplementary Figure S1**, **Supplementary Table S40**). The analysis revealed no evidence of heterogeneity or directed pleiotropic effects (Egger intercept = -0.007, P=0.697), underscoring the stability of our findings (**Supplementary Figures S3**, **S4**).

Similarly, our investigation into bone mineral density and calcium levels showed no causal connections with Preeclampsia risk (Bone mineral density OR: 1.082; 95% CI: 0.953-1.229, P=0.222; Calcium levels OR: 0.978; 95% CI: 0.799-1.198, P=0.833; **Supplementary Figures S1**, **Supplementary Tables S41**, **S42**). These results indicate that neither bone mineral density nor calcium levels play a significant role in the development of Preeclampsia.

Furthermore, analyses of serum 25-Hydroxyvitamin D levels also revealed no causal association with an increased risk of Preeclampsia (OR: 0.932; 95% CI: 0.722-1.203, P=0.589; **Figure 4**, **Supplementary Table S43**). This finding, supported by a lack of evidence for heterogeneity or pleiotropic effects (Egger intercept = 0.001, P=0.923), suggests that 25-Hydroxyvitamin D levels are not a significant factor in Preeclampsia development, as confirmed by consistent results across various analytical methods and highlighted in our robustness checks (**Supplementary Figures S3**, **S4**).

### 3.5 Unveiling the influence of other potential factors on preeclampsia risk

In this MR investigation, we explored the impact of various undisclosed factors on Preeclampsia risk, focusing on Chronic kidney disease, Serum uric acid levels, Alanine aminotransferase levels, Placental growth factor, Vascular endothelial growth factor levels, Hemoglobin concentration, and Platelet count. Our analyses revealed a significant causal relationship between elevated Serum uric acid levels and an increased risk of Preeclampsia (OR: 1.215; 95% CI: 1.007-1.465; P = 0.042), indicating a 21.47% rise in risk for each standard deviation increase in genetically predicted levels. This robust association was observed consistently across various analytical methods, shown in **Supplementary Figure S1** and detailed in **Supplementary Table S45**. Minimal heterogeneity (Q = 267.320, P = 0.066) and the absence of significant pleiotropic effects (Egger intercept = -0.003, P = 0.437) from MR-Egger regression analysis suggest a strong link.

Conversely, no substantial causal associations were found for Chronic kidney disease, with results detailed in **Supplementary Figure S1** and **Supplementary Table S44** (OR: 0.811; 95% CI: 0.646-1.019; P = 0.072). Similar non-significant findings were observed for Alanine aminotransferase levels (OR: 1.141; 95% CI: 0.934-1.393; P = 0.196; **Supplementary Figure S1**, **Supplementary Table S46**) and other measured factors such as Placental Growth Factor, Vascular Endothelial Growth Factor Levels, Hemoglobin Concentration, and Platelet Count, with comprehensive results presented in **Supplementary Tables S47**-**S50**. These findings were consistently non-significant across various models, with heterogeneity within acceptable limits (as shown in **Supplementary Figures S3**, **S4**) and no evidence of directional effects, indicating these factors do not significantly impact Preeclampsia risk.

## 4 Discussion

### 4.1 Main findings

Our comprehensive MR analyses illuminate the intricate network of risk factors associated with preeclampsia. Utilizing the IVW method, supplemented by other MR techniques, we examined 25 exposure factors across five distinct categories. This approach allowed us to critically address potential biases and confounding factors, thereby reinforcing the validity of our findings. Several factors have been identified with a causal relationship to the risk of developing preeclampsia, which are pivotal in guiding future research and intervention strategies.

### 4.2 Detailed comparison with other studies on preeclampsia risk factors

#### 4.2.1 Metabolic risk factors

Our exploration of metabolic risk factors uncovers complex relationships with preeclampsia, adding new dimensions to the existing literature (28–34). Contrary to some observational studies (35–38), our findings indicate that type 1 diabetes does not significantly increase preeclampsia risk. This could be attributed to the autoimmune nature of type 1 diabetes, which may not involve the same metabolic pathways that exacerbate preeclampsia, such as insulin resistance and systemic inflammation typically associated with type 2 diabetes. Conversely, our robust evidence supports a causal link between type 2 diabetes and increased preeclampsia risk, reinforcing the concept of metabolic syndrome’s impact on endothelial function and inflammatory status (31, 39, 40). Additionally, the lack of a causal relationship between gestational diabetes and preeclampsia invites a reevaluation of its role, suggesting transient hyperglycemia may not reach the threshold necessary to influence preeclampsia pathogenesis. Moreover, we identify a genetic predisposition to hyperthyroidism and elevated BMI as risk enhancers for preeclampsia, highlighting the intricate interplay between various metabolic disorders and preeclampsia risk (41–44). These insights emphasize the need for personalized management strategies in maternal healthcare, tailored to the metabolic profiles of pregnant women.

#### 4.2.2 Lipid-related factors

In examining lipid metabolism, we provide detailed insights into how specific lipid fractions influence preeclampsia risk (2, 45–48). While total cholesterol does not show a causal relationship with preeclampsia, triglycerides and LDL-C are implicated in enhancing risk, possibly through mechanisms involving oxidative stress and lipid peroxidation, which adversely affect placental function (49). On the other hand, HDL-C exhibits protective effects, likely due to its role in promoting endothelial health and reducing inflammation. These findings underscore the critical need for monitoring lipid profiles in pregnancy, suggesting that targeted lipid management could serve as an early intervention strategy to mitigate the risk of developing preeclampsia.

#### 4.2.3 Immune and inflammatory factors

Our rigorous MR analysis extends to immune and inflammatory factors, where we establish a causal relationship between genetically inferred systemic lupus erythematosus (SLE) and increased preeclampsia risk (50–54). This supports the theory that autoimmunity, through its disruptive influence on immune homeostasis, contributes significantly to the pathophysiology of preeclampsia. In contrast, the absence of associations with gout and rheumatoid arthritis indicates that not all inflammatory conditions exert similar effects, underscoring the specificity of immune pathways involved in preeclampsia (51). This differentiation is crucial for developing targeted therapies and preventive measures in managing preeclampsia risk among different patient populations.

#### 4.2.4 Lifestyle and bone metabolism factors

Our further exploration into lifestyle and bone metabolism factors, specifically sleep duration, bone mineral density, calcium levels, and serum 25-hydroxyvitamin D levels, reveals no significant association with preeclampsia. This finding challenges previous observational studies (55–60) that suggested potential links between these factors and preeclampsia risk. The robustness of our results is underscored by the lack of heterogeneity in IVW analyses and the absence of directed pleiotropic effects. These rigorous, genetics-based analyses provide a more reliable assessment compared to traditional observational studies, highlighting the necessity for continued use of such robust methodologies in future research to accurately discern the risk factors associated with preeclampsia.

#### 4.2.5 Other potential factors

The inclusion of controversial factors such as chronic kidney disease (61, 62), serum uric acid levels (63, 64), alanine aminotransferase levels (65, 66), placental growth factor (67–69), vascular endothelial growth factor levels (70), hemoglobin concentration (71), and platelet count (72, 73) provides convincing evidence. Notably, no causal relationship is identified between chronic kidney disease and preeclampsia. This finding suggests that while chronic kidney disease is a known risk factor for many pregnancy complications, its mechanisms may not directly contribute to preeclampsia pathogenesis.

However, our results establish a significant causal link between genetically determined serum uric acid levels and increased preeclampsia risk. Elevated serum uric acid levels may contribute to endothelial dysfunction, oxidative stress, and inflammation, which are key mechanisms in the development of preeclampsia (63). This underscores the potential of serum uric acid as a biomarker for preeclampsia risk, providing a target for early intervention.

Importantly, placental growth factor (PlGF) and vascular endothelial growth factor (VEGF) exhibit no causal association with preeclampsia. These findings align with certain observational studies and suggest that despite their critical roles in placental health and vascular function (74), other mechanisms may be more significant in preeclampsia onset. Additionally, our analyses do not support an association between alanine aminotransferase levels, hemoglobin concentrations, and platelet counts with preeclampsia. This suggests these markers may not directly influence preeclampsia pathogenesis, despite being often altered in the condition. These findings clarify debated factors, adding new insights into preeclampsia.

### 4.3 Strengths and weakness of the study

While our study significantly advances the field of genetic epidemiology in preeclampsia, it acknowledges inherent limitations related to the assumptions required for MR analyses, such as the absence of pleiotropy and the proper handling of population stratification. Methods like MR-Egger regression have been employed to mitigate these issues, though they cannot fully eliminate the possibility of residual confounding. Additionally, the generalizability of our findings may be limited, as our data primarily derive from European ancestry datasets, highlighting the need for more inclusive genomic research.

Furthermore, our study primarily focused on modifiable lifestyle factors, such as sleep duration, while other important risk factors like age and gynecological history were not directly investigated. Although age was considered and accounted for as a confounding factor in our analysis, we recognize that including a broader range of risk factors could provide a more comprehensive understanding of preeclampsia risk. This focus on modifiable factors, while aligned with our study’s objectives, may have inadvertently overlooked the contributions of non-modifiable factors that also play crucial roles in the development of preeclampsia.

### 4.4 Implications for clinical practice and research

Our comprehensive MR analyses enhance understanding of the multifaceted nature of preeclampsia risk factors. By employing diverse MR methods, we not only confirm known associations but also challenge existing paradigms, paving the way for a deeper understanding of the complex genetic interactions influencing the risk of developing preeclampsia. This could potentially lead to more targeted prevention strategies and therapeutic interventions tailored to individual risk profiles, enhancing outcomes in maternal and perinatal health.

## 5 Conclusion

Our extensive MR investigation unravels the intricate genetic tapestry influencing the risk of preeclampsia, encompassing a diverse array of risk factors. Our findings provide robust insights into the complex interplay of genetic determinants across metabolic, lipid-related, immune, inflammatory, lifestyle, and other debated factors. This study not only refines established relationships but also unveils novel insights, elucidating the nuanced genetic interactions in conditions such as hyperthyroidism, BMI, and diabetes subtypes in relation to preeclampsia, while delineating the variable impacts of different lipid subtypes.

The significant causal link between genetically determined serum uric acid levels and increased preeclampsia risk underscores a crucial element in the risk landscape of this disorder. Our investigation into immune and inflammatory factors enhances our understanding by emphasizing the specificity of individual elements in the etiology of preeclampsia. Furthermore, by exploring novel elements, our study contributes to the evolving discourse in preeclampsia research, highlighting new avenues for inquiry.

While acknowledging the inherent limitations of our approach, the transparency and thoroughness of our methods reinforce the validity of our findings. This study deepens our understanding of the complex genetic architecture underpinning preeclampsia and lays a solid foundation for future research. It opens avenues for targeted interventions and preventive strategies in at-risk populations, underscoring the potential for personalized medicine in managing this multifaceted condition. Our research exemplifies the power of genetic epidemiology in unraveling the complexities of maternal health, providing a compass for future explorations that could transform our approach to preeclampsia and maternal care.

## Data Availability

The original contributions presented in the study are included in the article/**Supplementary Material**. Further inquiries can be directed to the corresponding author.
