# Supplementary material for: Causal pathways in preeclampsia: a Mendelian randomization study in European populations
Source: Front Endocrinol (Lausanne). 2024 Sep 2;15:1453277. doi: 10.3389/fendo.2024.1453277 (PMC11402816; doi:10.3389/fendo.2024.1453277)
Supplement: Supplementary file 1 [file DataSheet1.pdf]

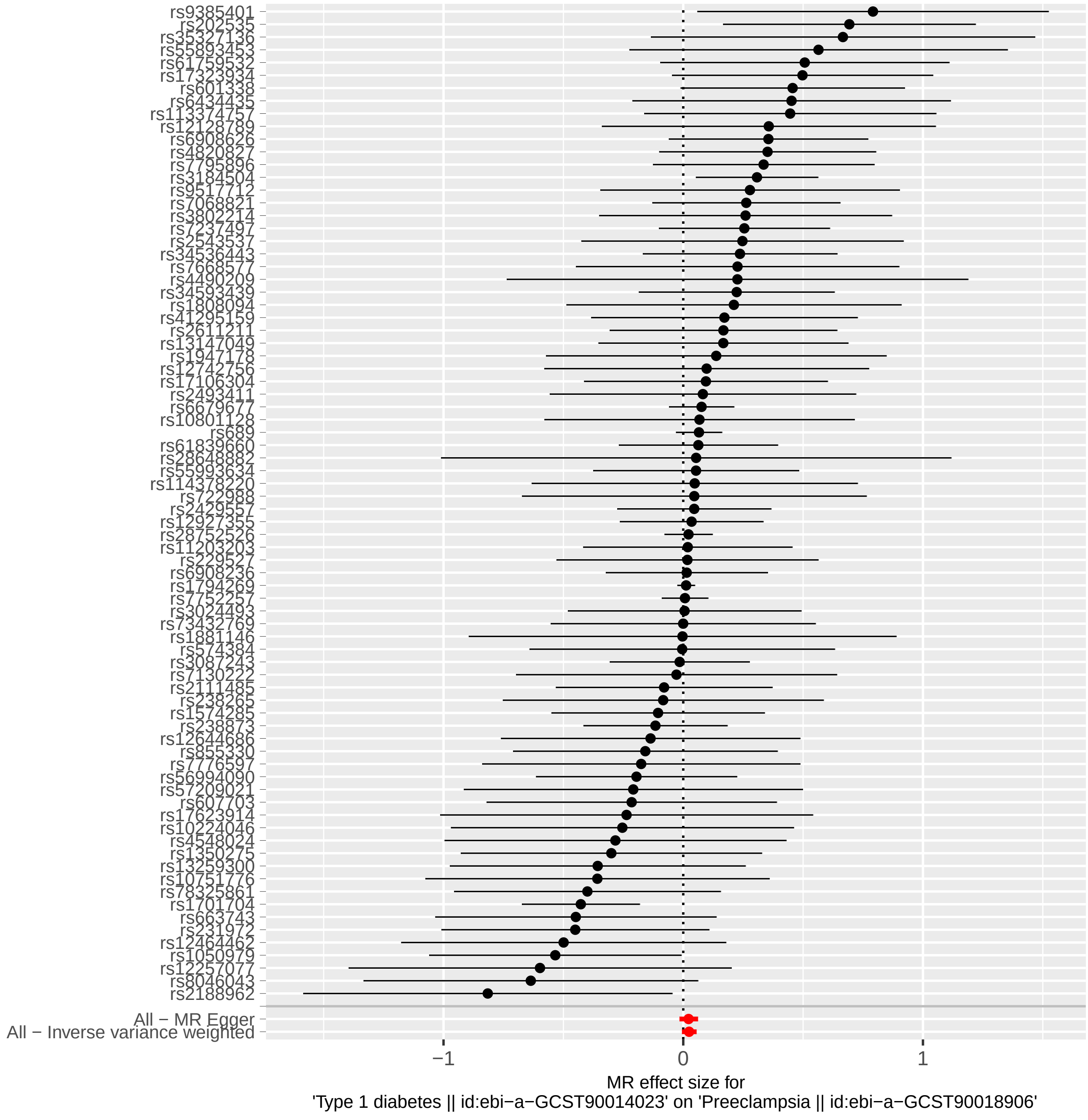
rs9385401
rs202535
rs35327136
rs55893453
rs61759532
rs17323934
rs601338
rs6434435
rs113374757
rs12128789
rs6908626
rs4820827
rs7795896
rs3184504
rs9517712
rs7068821
rs3802214
rs7237497
rs2543537
rs34536443
rs7668577
rs4490209
rs34593439
rs1808094
rs41295159
rs2611211
rs13147049
rs1947178
rs12742756
rs17106304
rs2493411
rs6679677
rs10801128
rs689
rs61839660
rs28648882
rs55993634
rs114378220
rs722988
rs2429557
rs12927355
rs28752526
rs11203203
rs229527
rs6908236
rs1794269
rs7752257
rs3024493
rs73432769
rs1881146
rs574384
rs3087243
rs7130222
rs2111485
rs238265
rs1574285
rs238873
rs12644686
rs855330
rs7776597
rs56994090
rs57209021
rs607703
rs17623914
rs10224046
rs4548024
rs1350275
rs13259300
rs10751776
rs78325861
rs1701704
rs663743
rs231972
rs12464462
rs1050979
rs12257077
rs8046043
rs2188962
All − MR Egger
All − Inverse variance weighted
−1
0
1
MR effect size for
'Type 1 diabetes || id:ebi−a−GCST90014023' on 'Preeclampsia || id:ebi−a−GCST90018906'

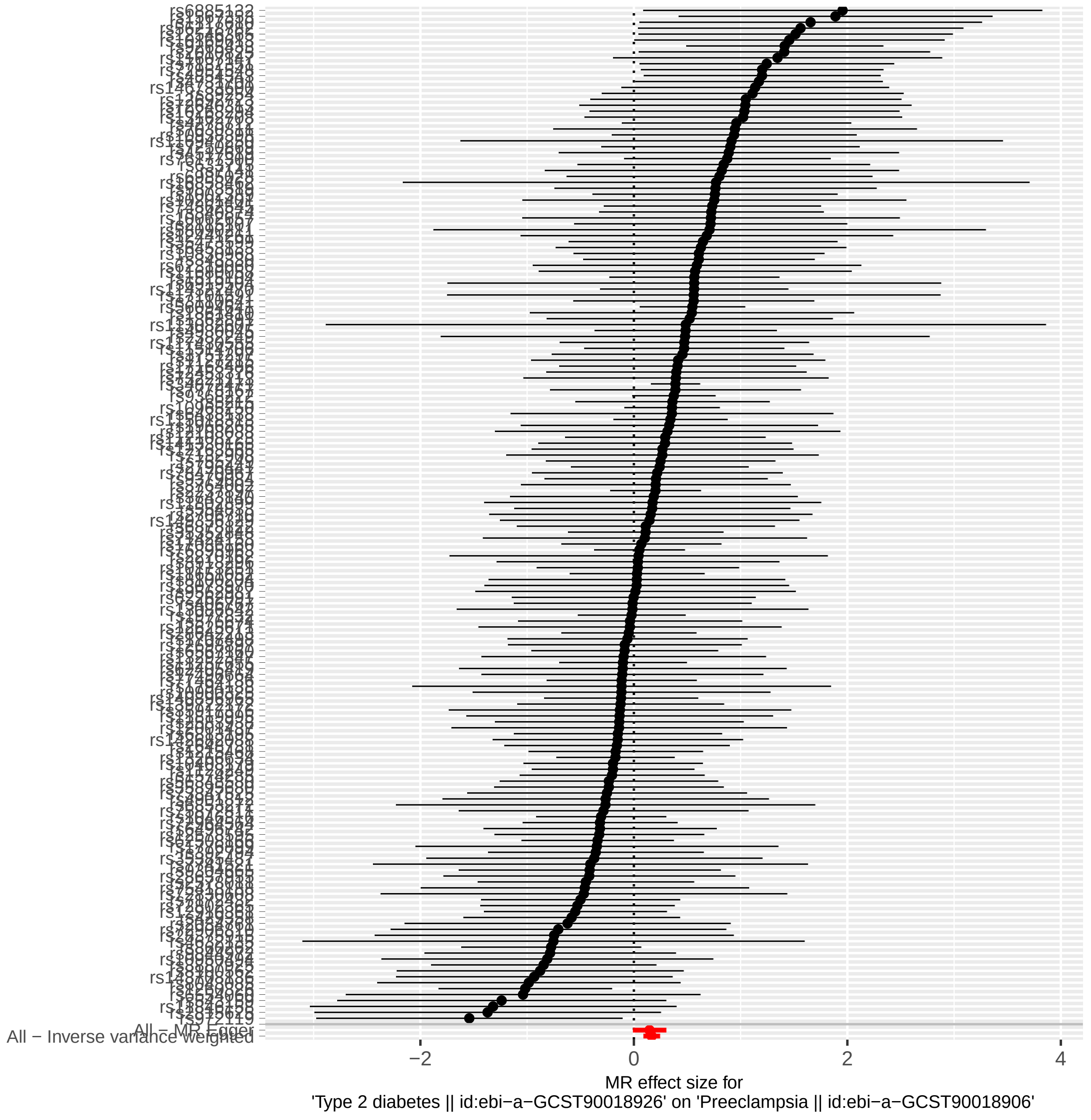
rs6885122
All − MR Egger
All − Inverse variance weighted
−2
0
2
4
MR effect size for
'Type 2 diabetes || id:ebi−a−GCST90018926' on 'Preeclampsia || id:ebi−a−GCST90018906'

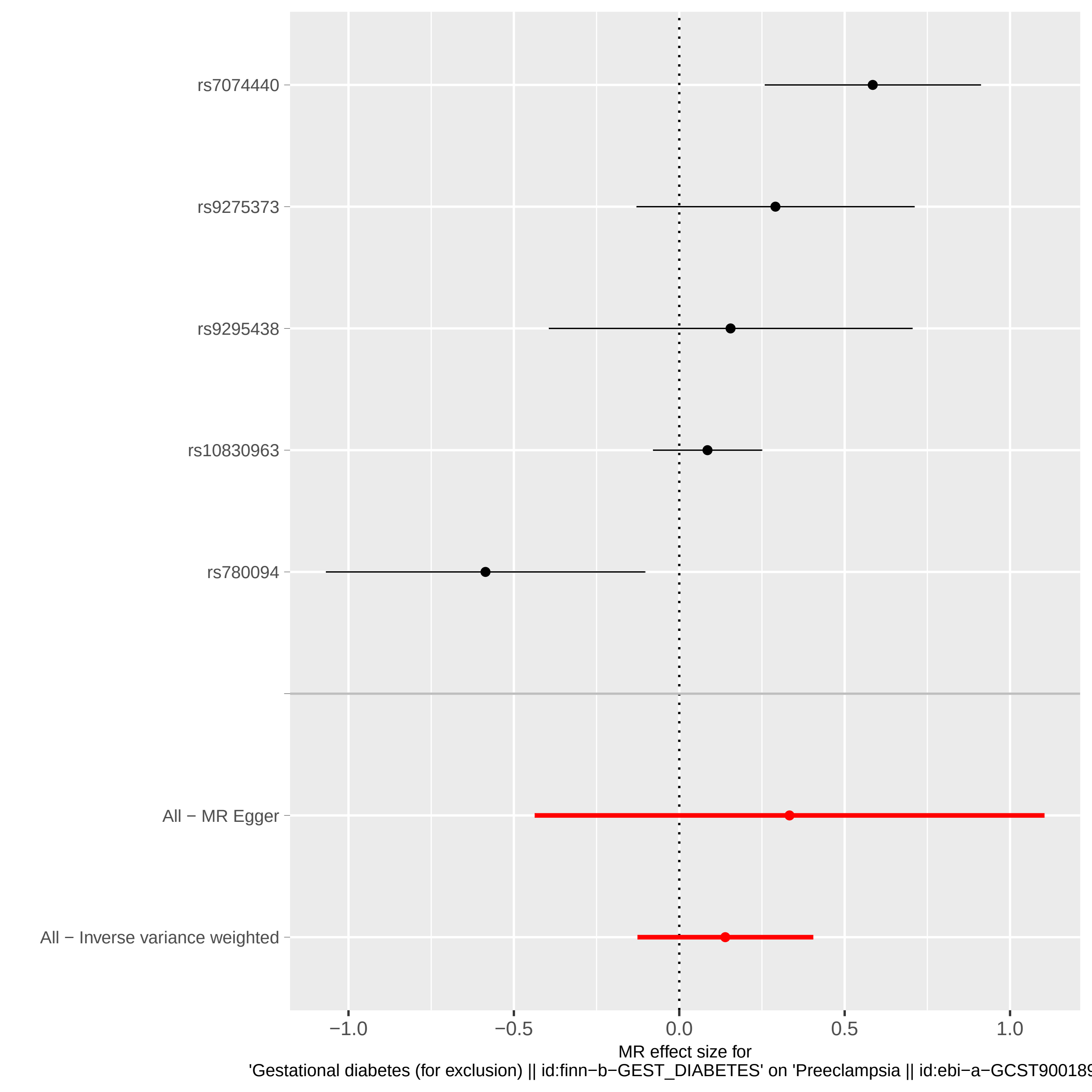

rs7074440
rs9275373
rs9295438
rs10830963
rs780094
All − MR Egger
All − Inverse variance weighted
−1.0
−0.5
0.0
0.5
1.0
MR effect size for
'Gestational diabetes (for exclusion) || id:finn−b−GEST_DIABETES' on 'Preeclampsia || id:ebi−a−GCST90018

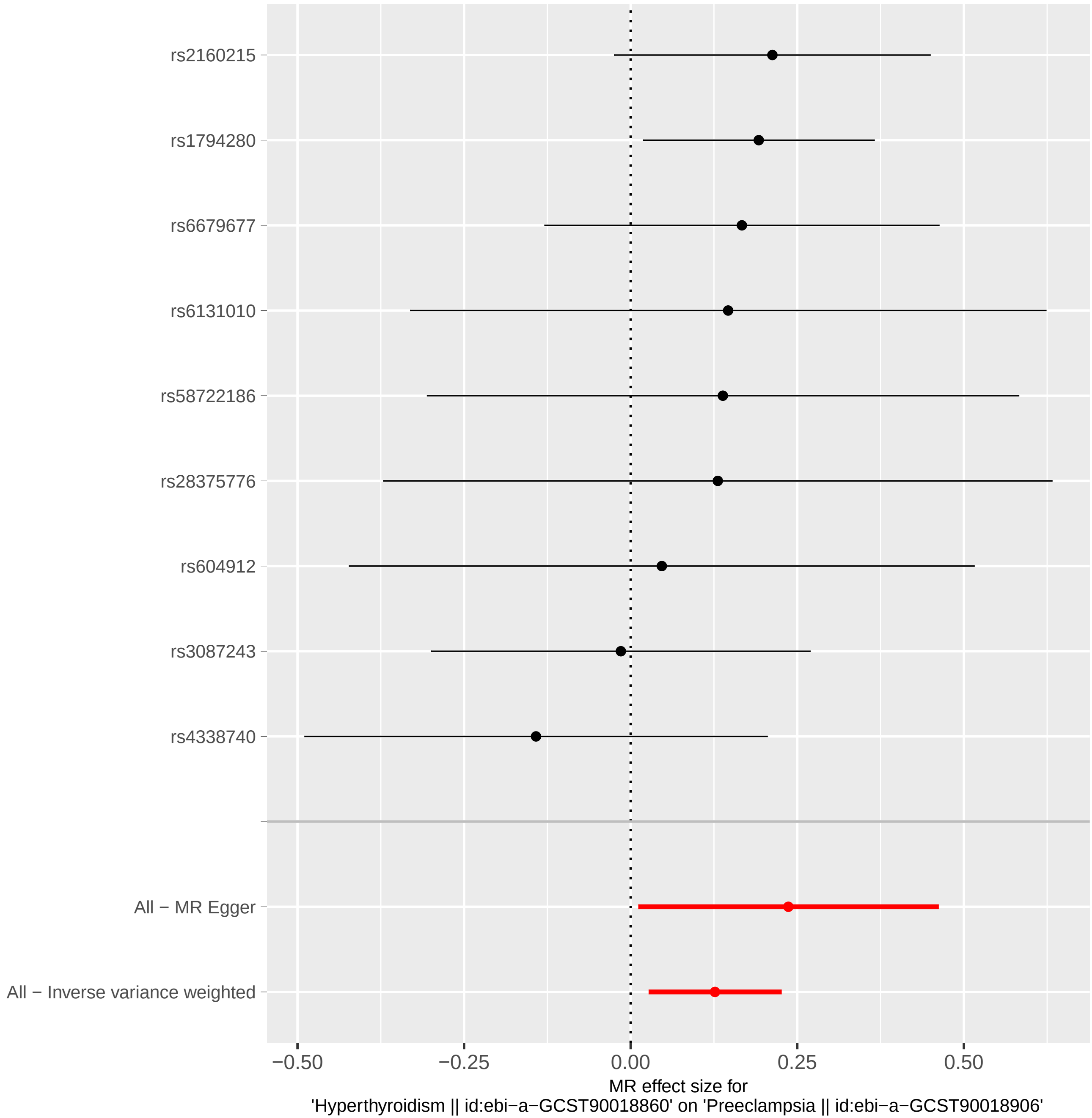

rs2160215
rs1794280
rs6679677
rs6131010
rs58722186
rs28375776
rs604912
rs3087243
rs4338740
All − MR Egger
All − Inverse variance weighted
−0.50
−0.25
0.00
0.25
0.50
MR effect size for
'Hyperthyroidism || id:ebi−a−GCST90018860' on 'Preeclampsia || id:ebi−a−GCST90018906'

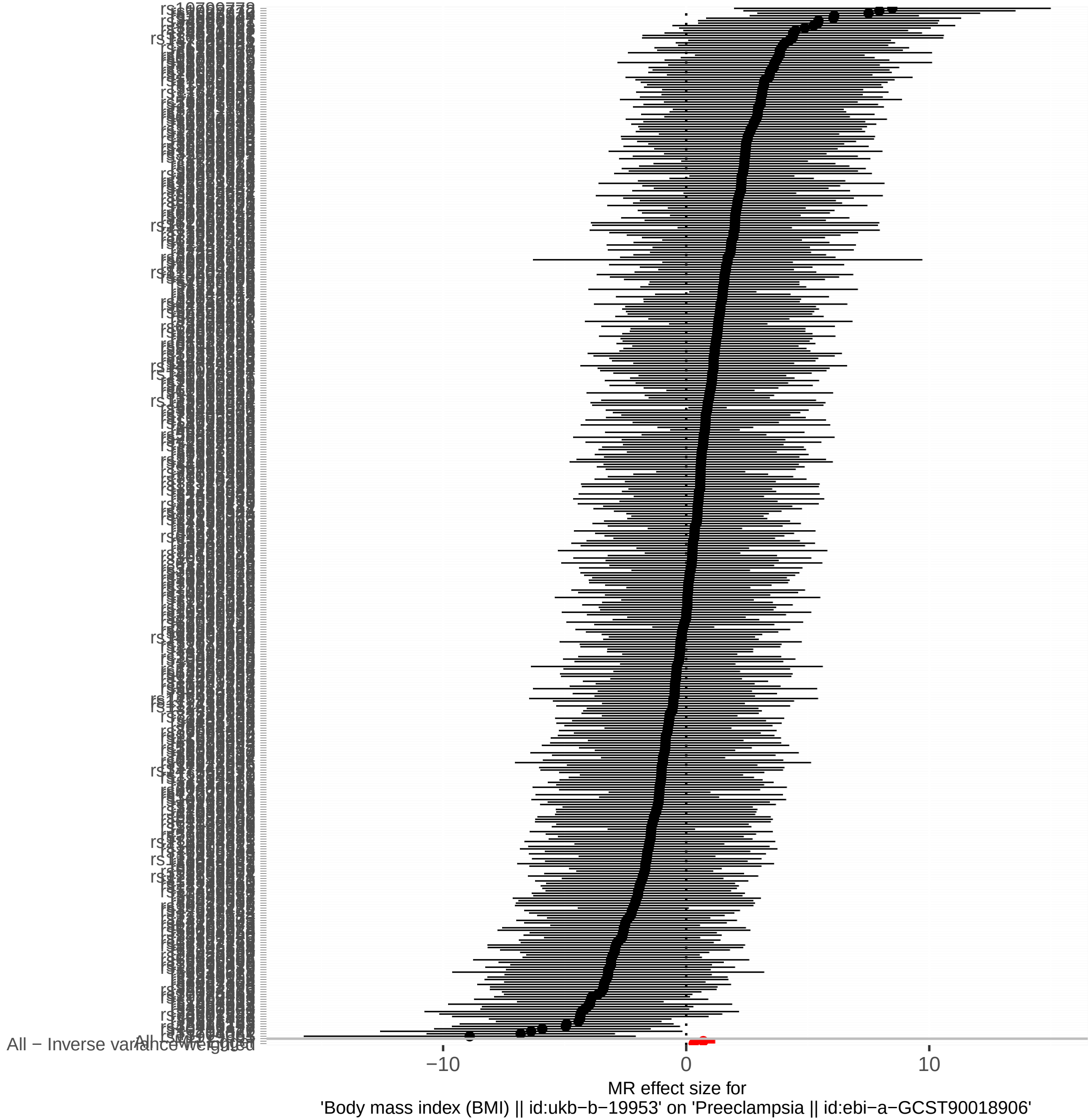
All − Inverse variance weighted
−10
0
10
MR effect size for
'Body mass index (BMI) || id:ukb−b−19953' on 'Preeclampsia || id:ebi−a−GCST90018906'

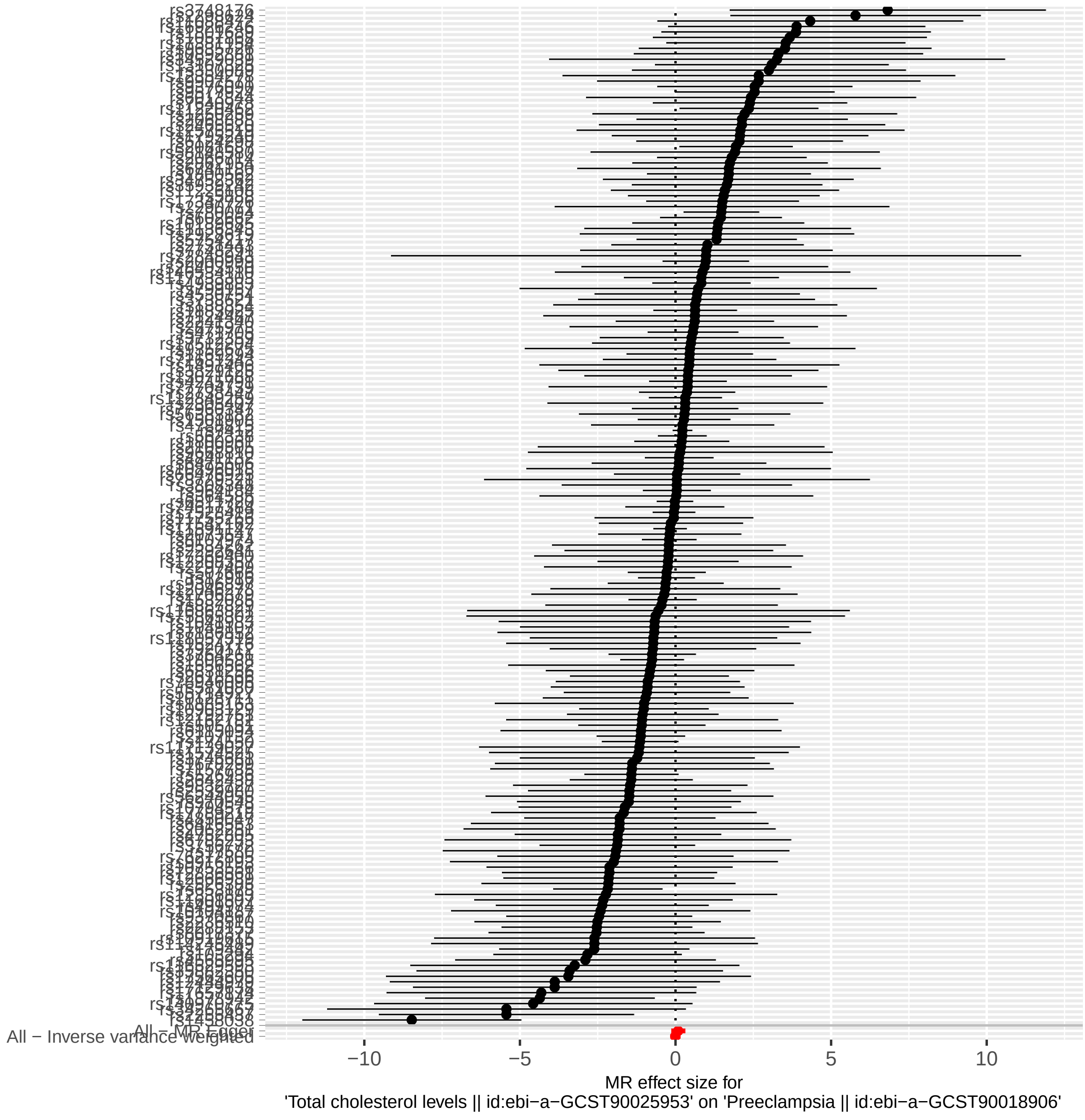
rs3748176
rs1453038
All − MR Egger
All − Inverse variance weighted
−10
−5
0
5
10
MR effect size for
'Total cholesterol levels || id:ebi−a−GCST90025953' on 'Preeclampsia || id:ebi−a−GCST90018906'

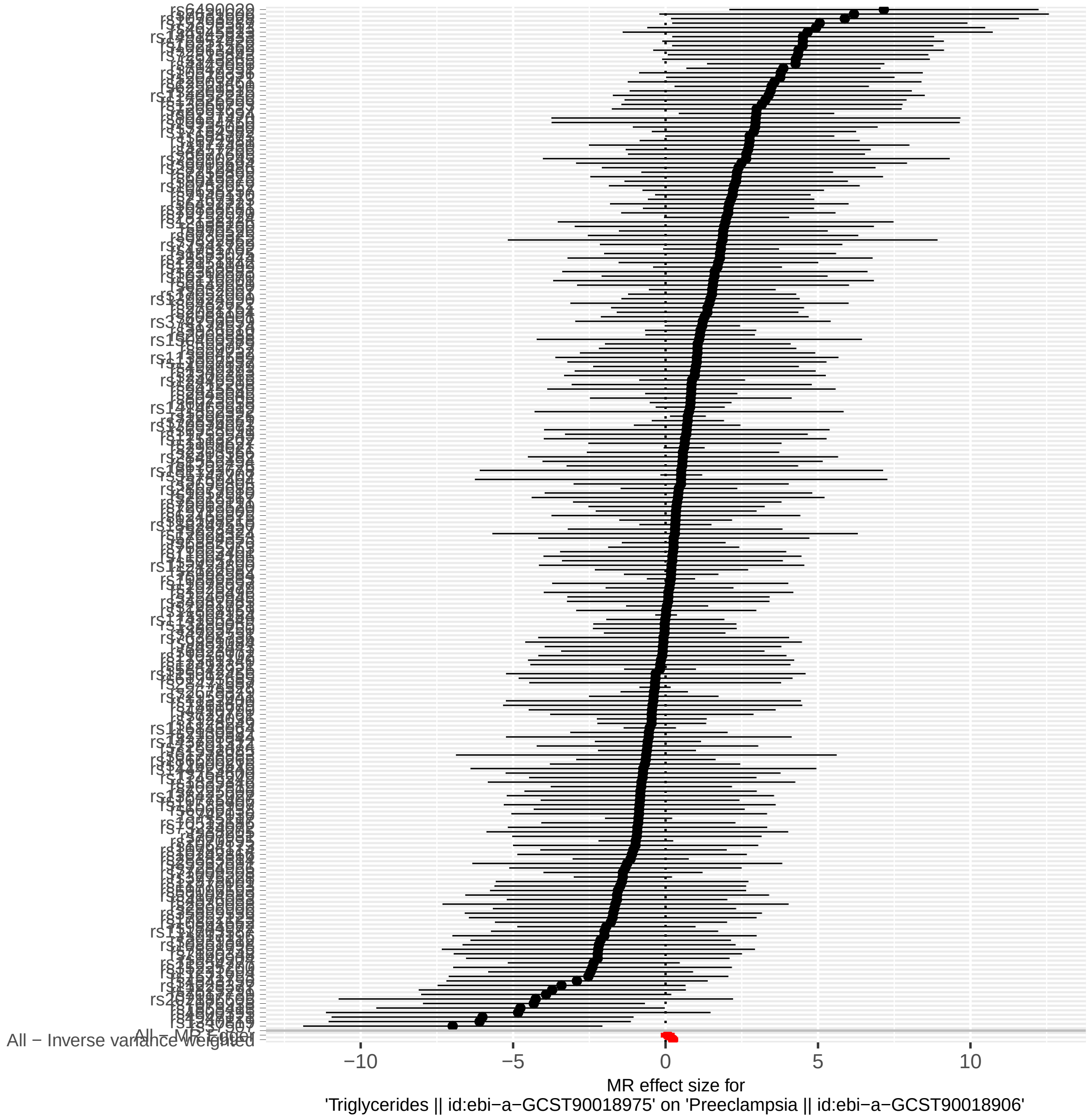
rs6490020
rs1840507
All − MR Egger
All − Inverse variance weighted
−10
−5
0
5
10
MR effect size for
'Triglycerides || id:ebi−a−GCST90018975' on 'Preeclampsia || id:ebi−a−GCST90018906'

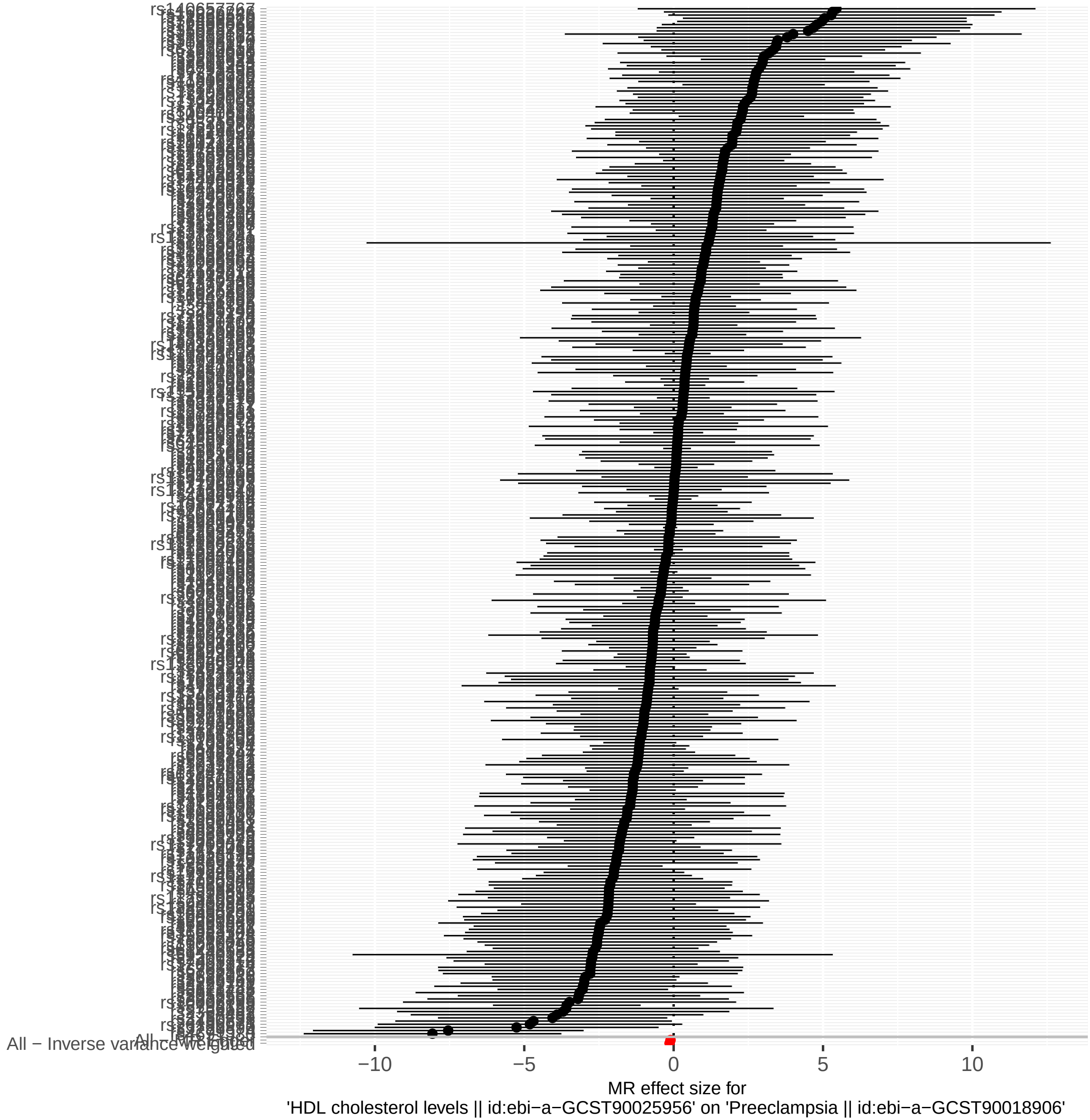

All − Inverse variance weighted
−10
−5
0
5
10
MR effect size for
'HDL cholesterol levels || id:ebi−a−GCST90025956' on 'Preeclampsia || id:ebi−a−GCST90018906'

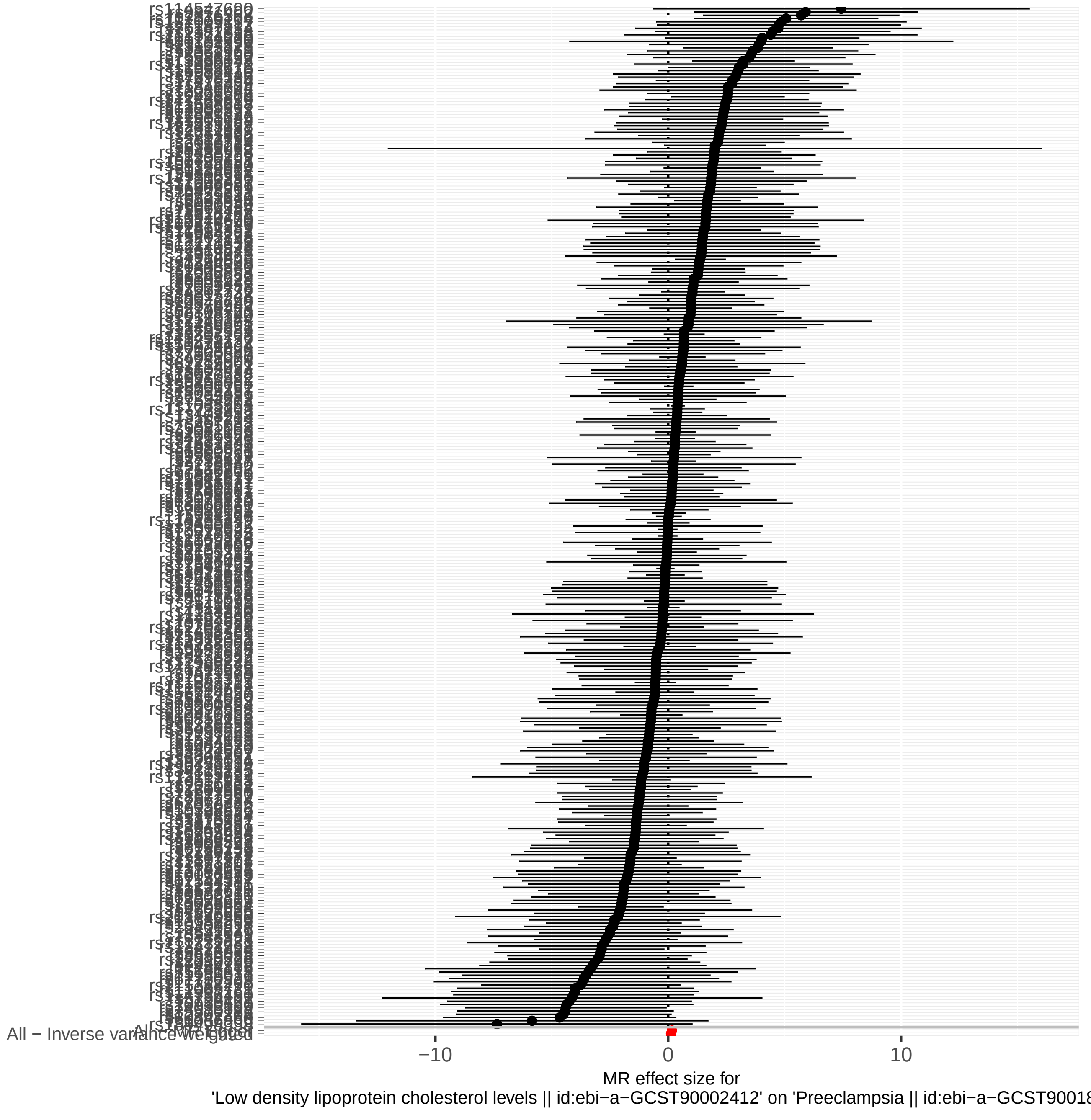

All − Inverse variance weighted
−10
0
10
MR effect size for
'Low density lipoprotein cholesterol levels || id:ebi−a−GCST90002412' on 'Preeclampsia || id:ebi−a−GCST90018

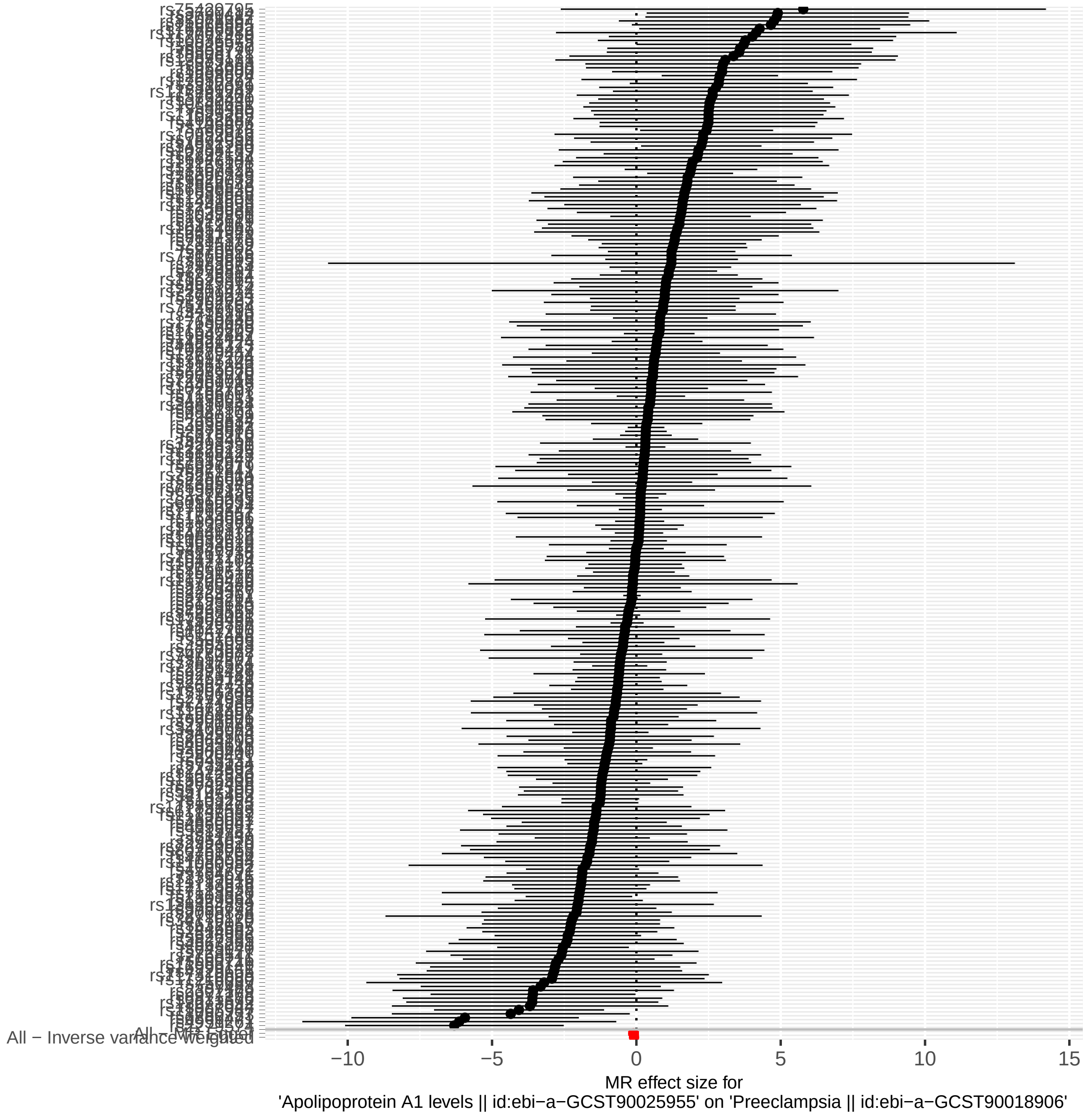
rs75430795
All − Inverse variance weighted
−10
−5
0
5
10
15
MR effect size for
'Apolipoprotein A1 levels || id:ebi−a−GCST90025955' on 'Preeclampsia || id:ebi−a−GCST90018906'

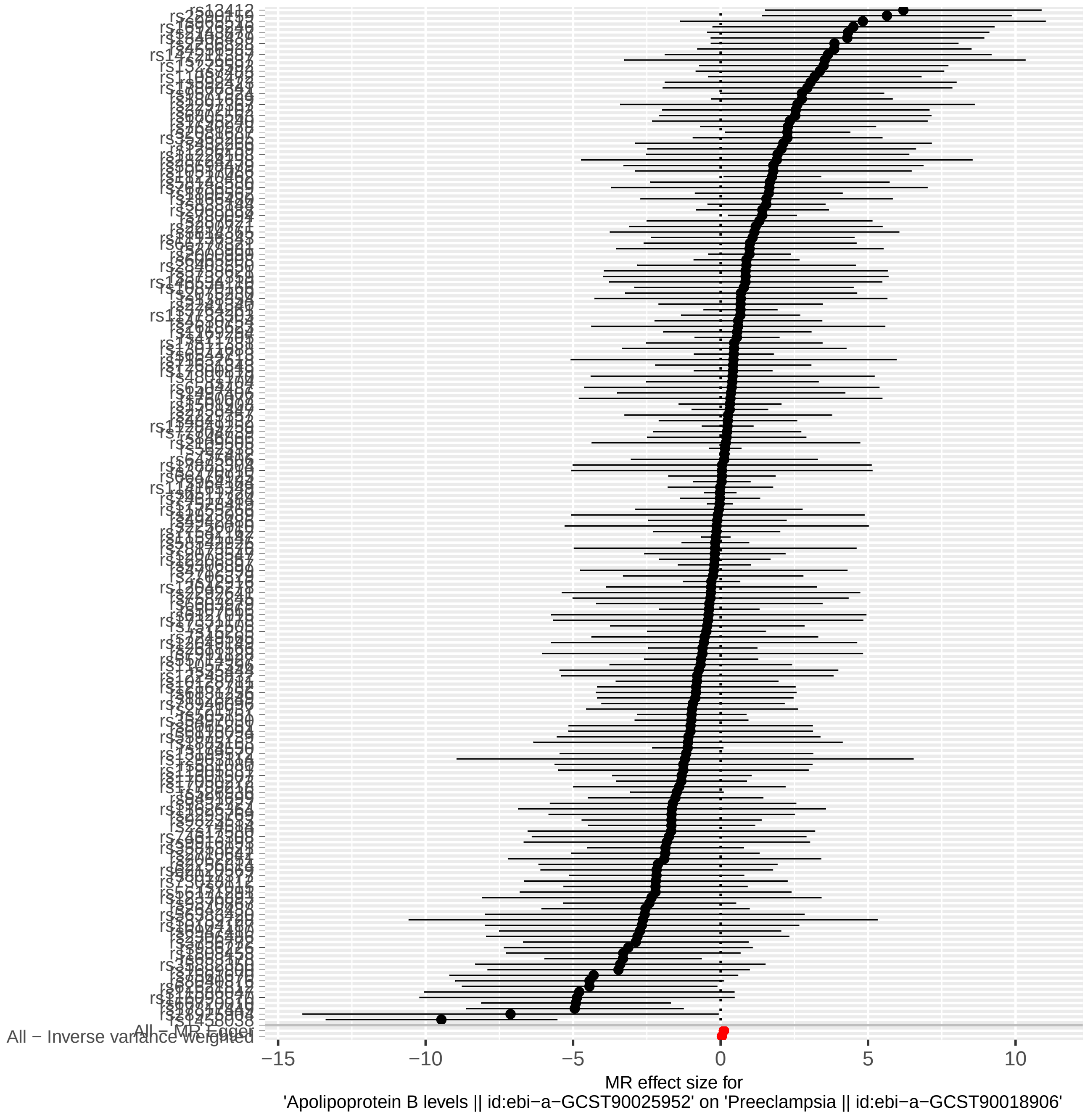

All − MR Egger
All − Inverse variance weighted
−15
−10
−5
0
5
10
MR effect size for
'Apolipoprotein B levels || id:ebi−a−GCST90025952' on 'Preeclampsia || id:ebi−a−GCST90018906'

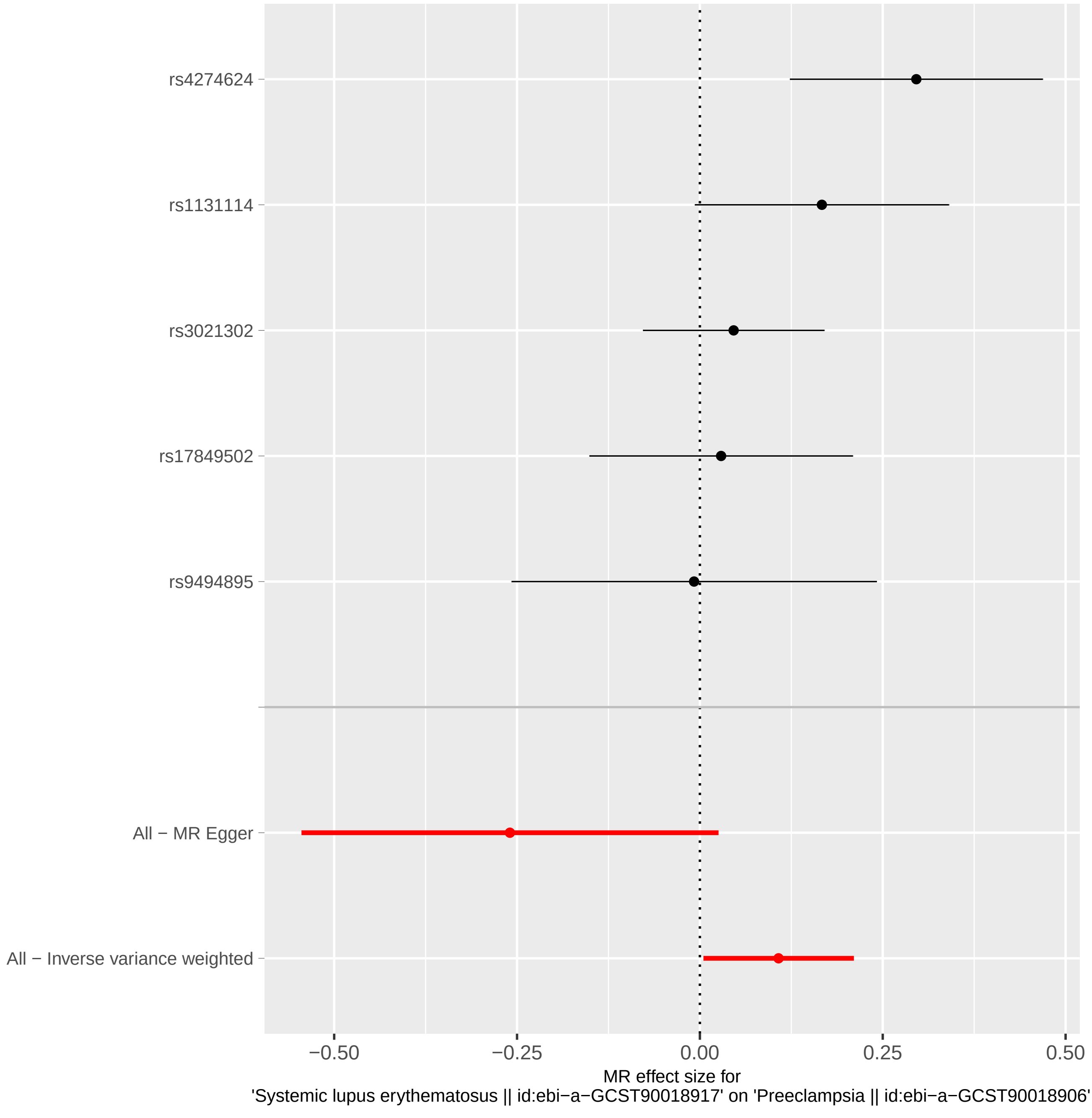
rs4274624
rs1131114
rs3021302
rs17849502
rs9494895
All − MR Egger
All − Inverse variance weighted
−0.50
−0.25
0.00
0.25
0.50
MR effect size for
'Systemic lupus erythematosus || id:ebi−a−GCST90018917' on 'Preeclampsia || id:ebi−a−GCST90018906'

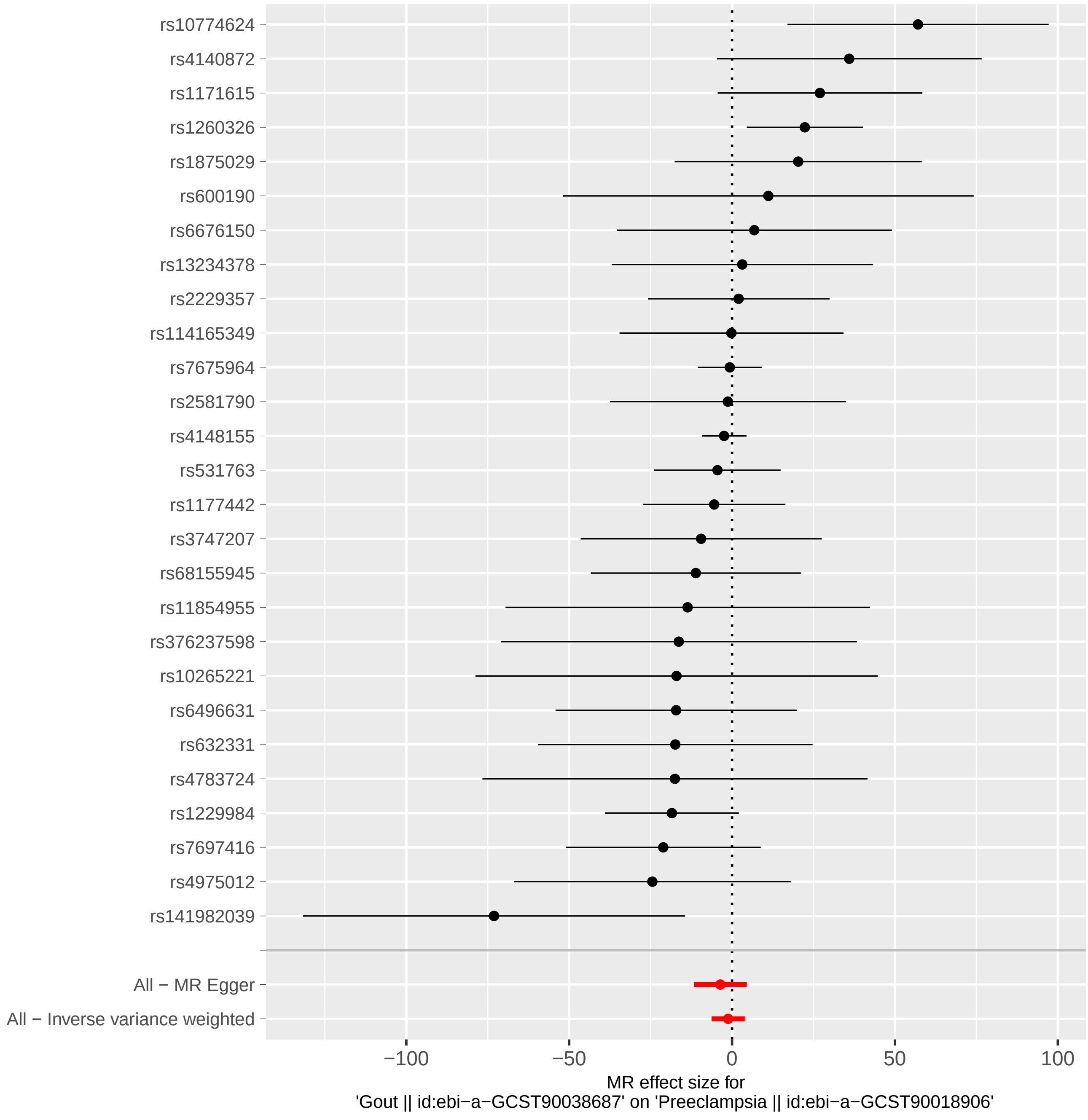

rs10774624
rs4140872
rs1171615
rs1260326
rs1875029
rs600190
rs6676150
rs13234378
rs2229357
rs114165349
rs7675964
rs2581790
rs4148155
rs531763
rs1177442
rs3747207
rs68155945
rs11854955
rs376237598
rs10265221
rs6496631
rs632331
rs4783724
rs1229984
rs7697416
rs4975012
rs141982039
All − MR Egger
All − Inverse variance weighted
−100
−50
0
50
100
MR effect size for
'Gout || id:ebi−a−GCST90038687' on 'Preeclampsia || id:ebi−a−GCST90018906'

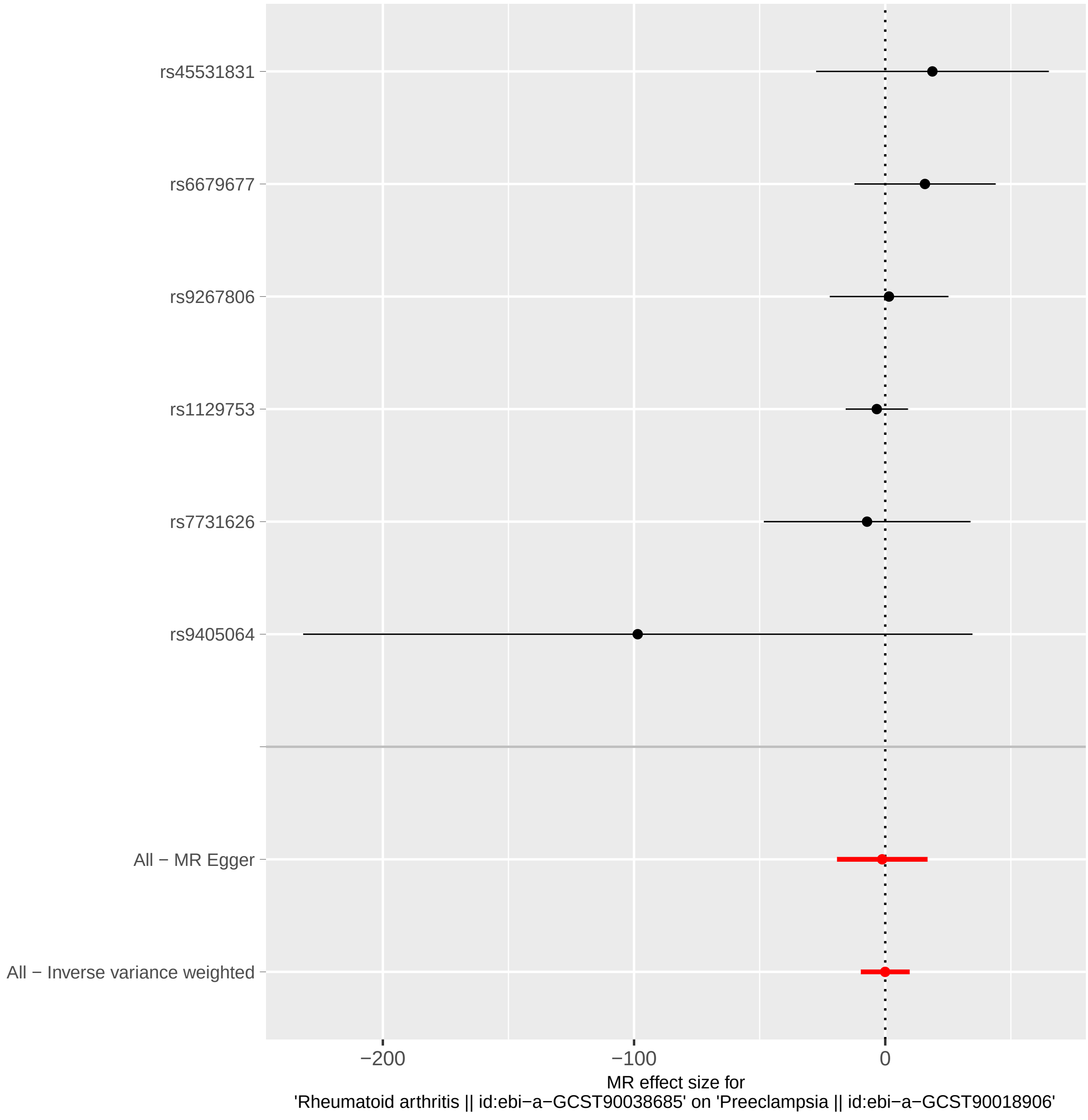

rs45531831
rs6679677
rs9267806
rs1129753
rs7731626
rs9405064
All − MR Egger
All − Inverse variance weighted
−200
−100
0
MR effect size for
'Rheumatoid arthritis || id:ebi−a−GCST90038685' on 'Preeclampsia || id:ebi−a−GCST90018906'

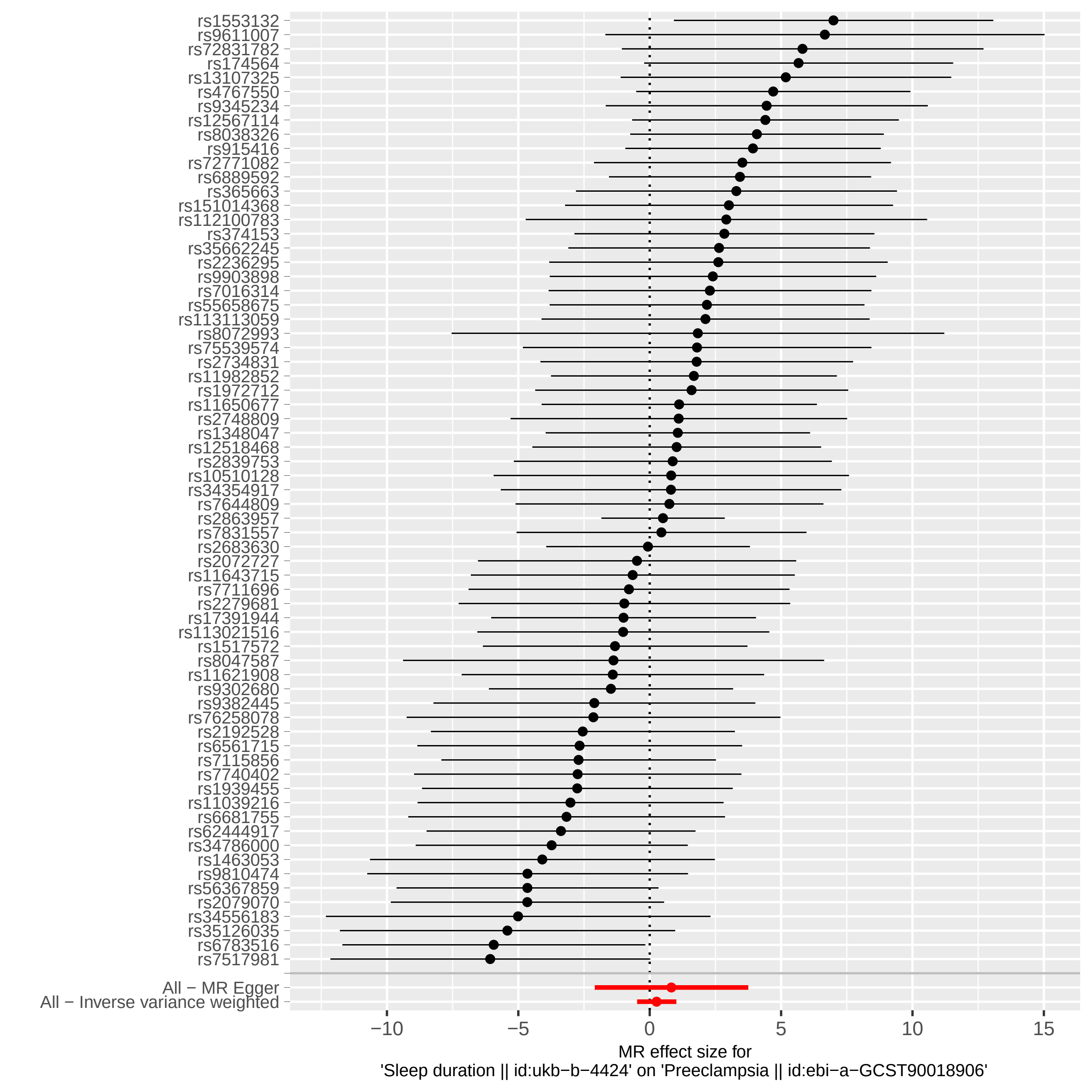

rs1553132
rs9611007
rs72831782
rs174564
rs13107325
rs4767550
rs9345234
rs12567114
rs8038326
rs915416
rs72771082
rs6889592
rs365663
rs151014368
rs112100783
rs374153
rs35662245
rs2236295
rs9903898
rs7016314
rs55658675
rs113113059
rs8072993
rs75539574
rs2734831
rs11982852
rs1972712
rs11650677
rs2748809
rs1348047
rs12518468
rs2839753
rs10510128
rs34354917
rs7644809
rs2863957
rs7831557
rs2683630
rs2072727
rs11643715
rs7711696
rs2279681
rs17391944
rs113021516
rs1517572
rs8047587
rs11621908
rs9302680
rs9382445
rs76258078
rs2192528
rs6561715
rs7115856
rs7740402
rs1939455
rs11039216
rs6681755
rs62444917
rs34786000
rs1463053
rs9810474
rs56367859
rs2079070
rs34556183
rs35126035
rs6783516
rs7517981
All − MR Egger
All − Inverse variance weighted
−10
−5
0
5
10
15
MR effect size for
'Sleep duration || id:ukb-b−4424' on 'Preeclampsia || id:ebi−a−GCST90018906'

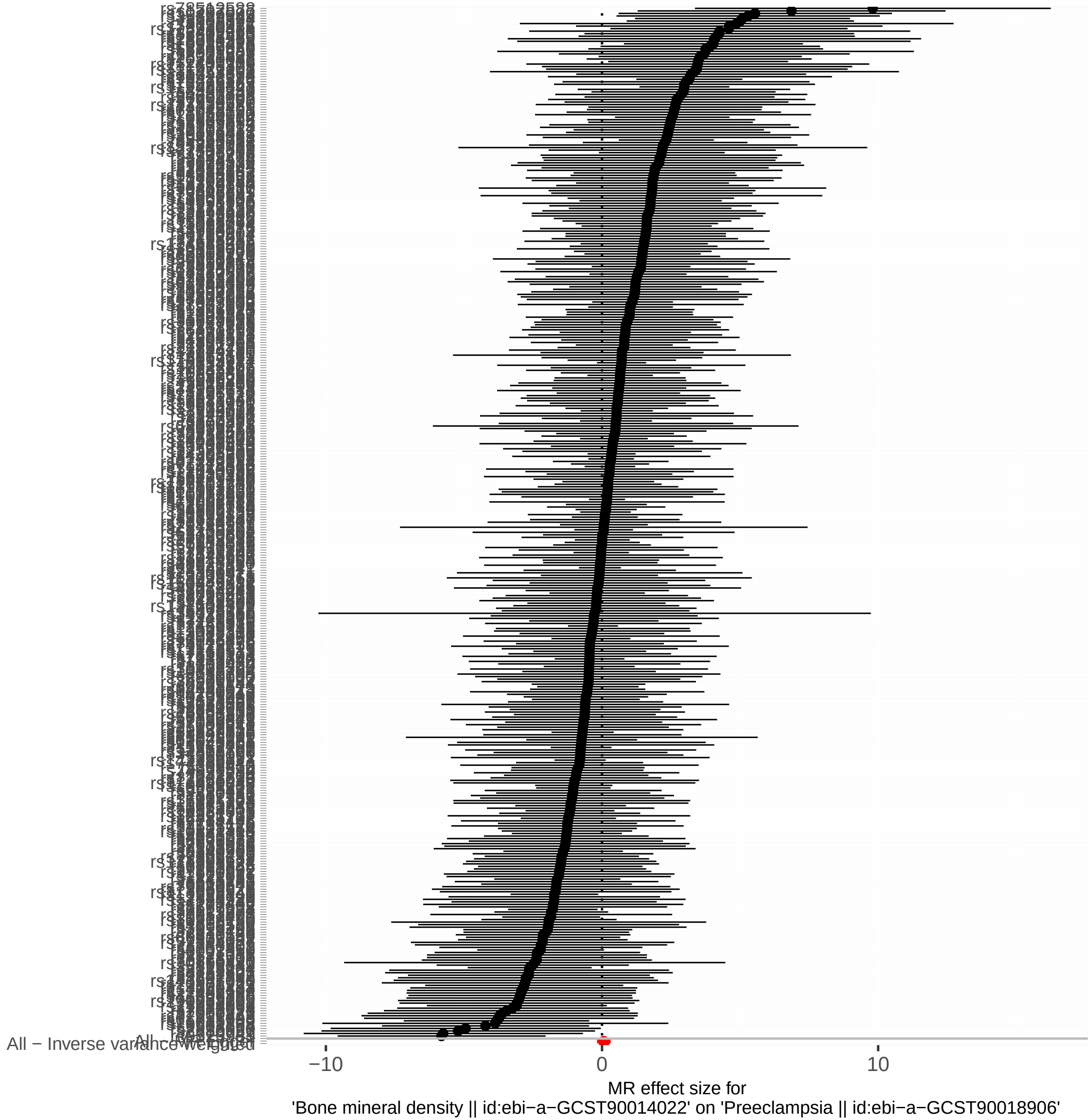
All − Inverse variance weighted
−10
0
10
MR effect size for
'Bone mineral density || id:ebi−a−GCST90014022' on 'Preeclampsia || id:ebi−a−GCST90018906'

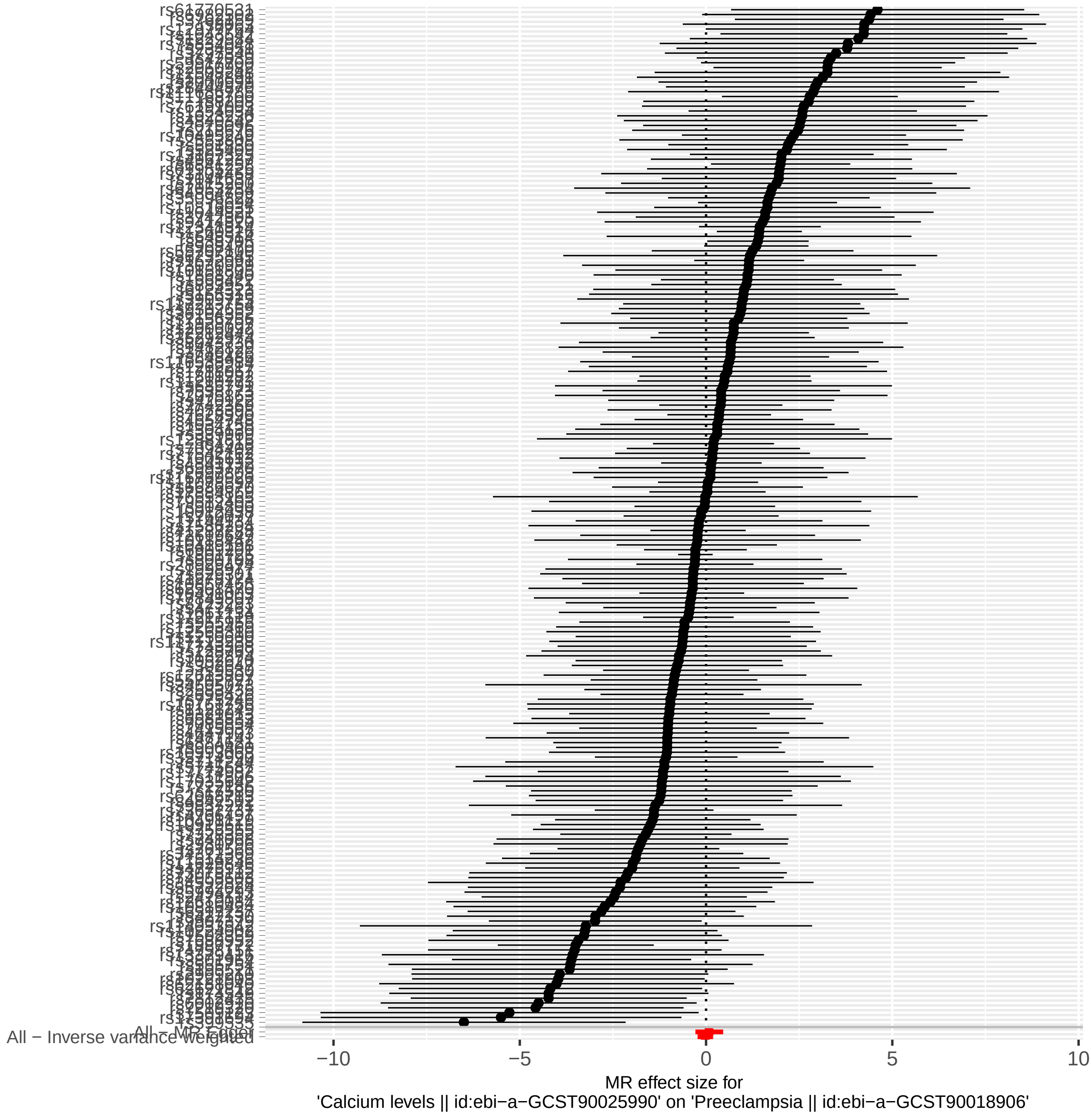

rs61770521
All − MR Egger
All − Inverse variance weighted
−10
−5
0
5
10
MR effect size for
'Calcium levels || id:ebi−a−GCST90025990' on 'Preeclampsia || id:ebi−a−GCST90018906'

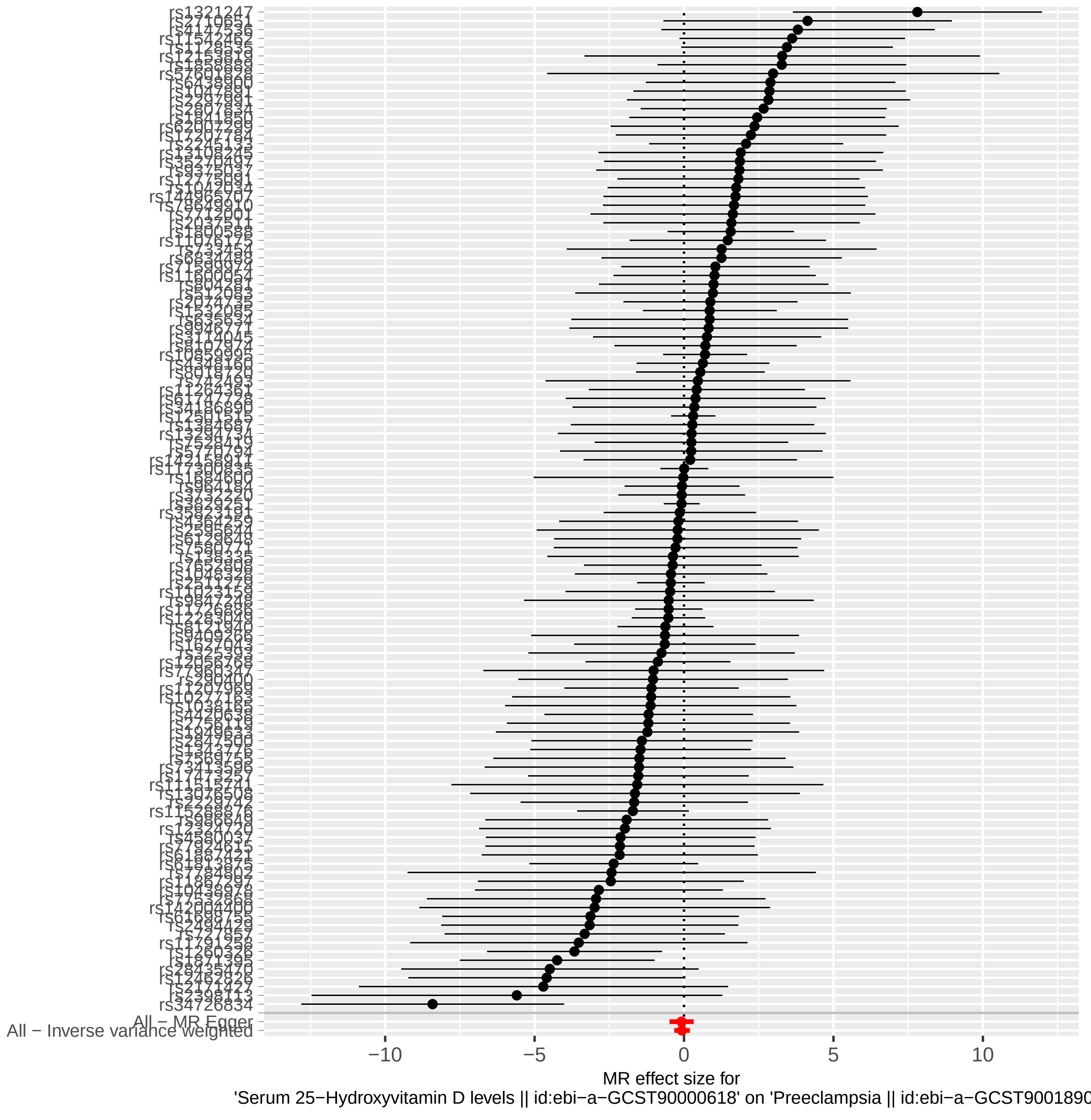

rs1321247
rs34726834
All − MR Egger
All − Inverse variance weighted
−10
−5
0
5
10
MR effect size for
'Serum 25−Hydroxyvitamin D levels || id:ebi−a−GCST90000618' on 'Preeclampsia || id:ebi−a−GCST9001890

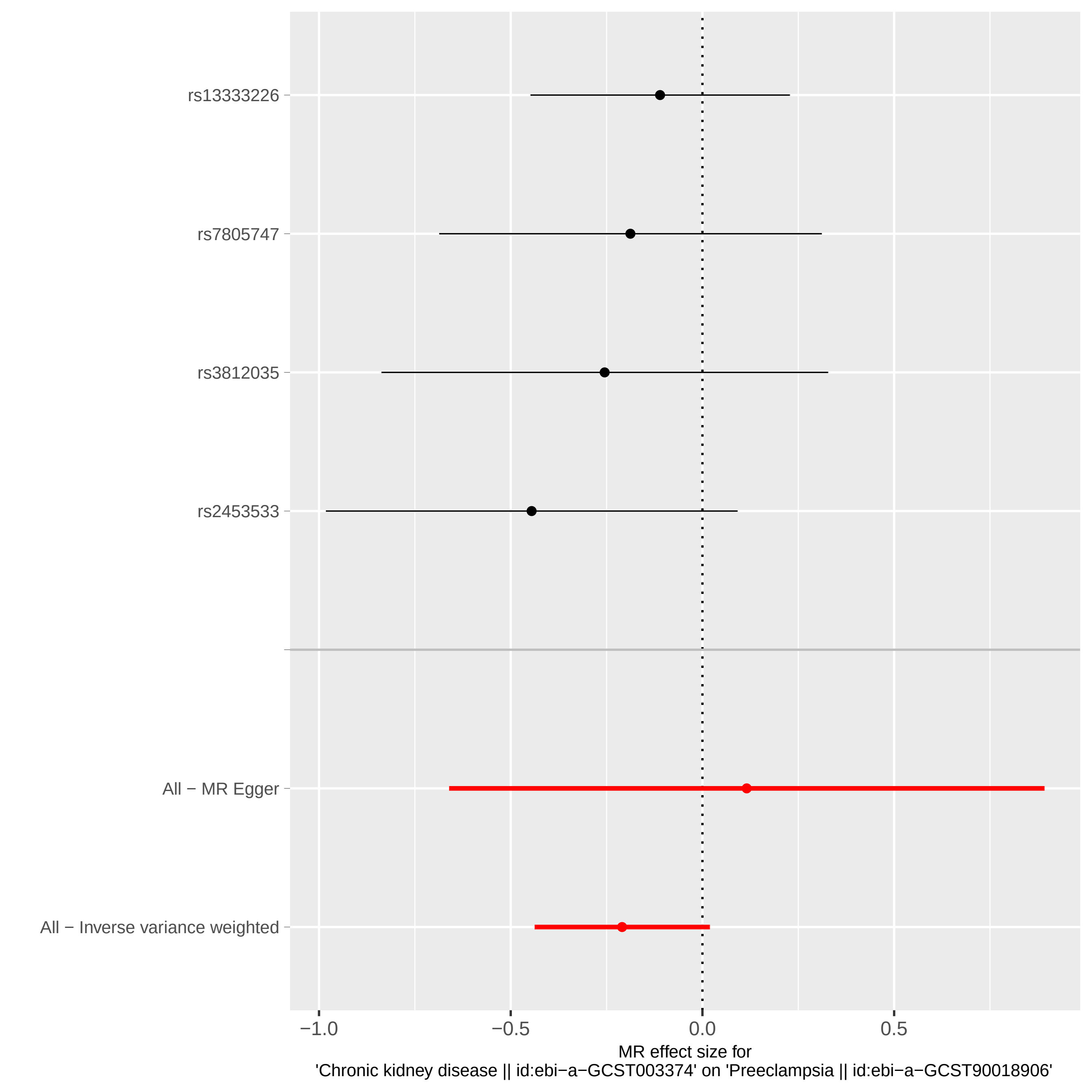
rs13333226
rs7805747
rs3812035
rs2453533
All − MR Egger
All − Inverse variance weighted
−1.0
−0.5
0.0
0.5
MR effect size for
'Chronic kidney disease || id:ebi−a−GCST003374' on 'Preeclampsia || id:ebi−a−GCST90018906'

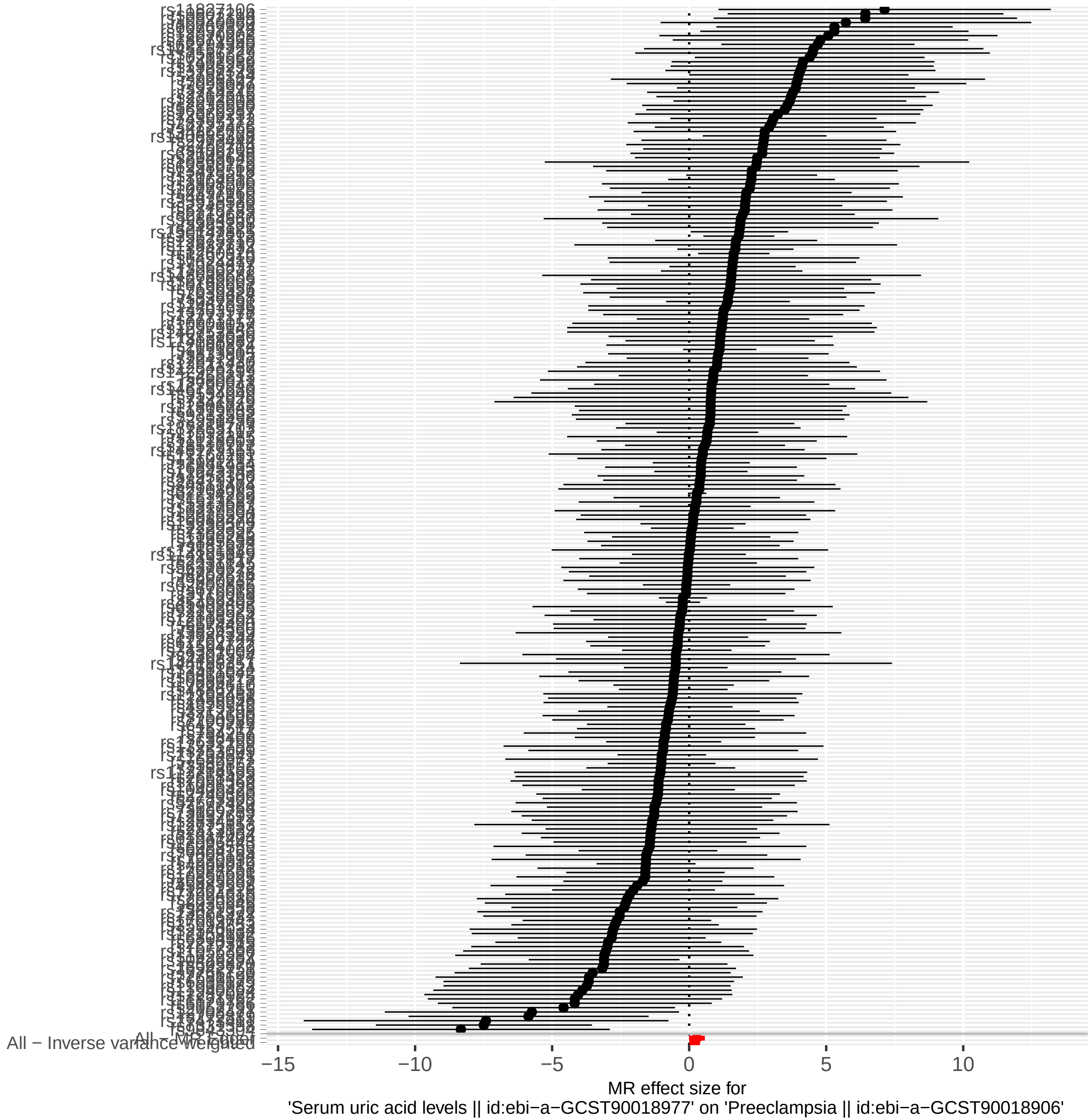

All − Inverse variance weighted
−15
−10
−5
0
5
10
MR effect size for
'Serum uric acid levels || id:ebi−a−GCST90018977' on 'Preeclampsia || id:ebi−a−GCST90018906'

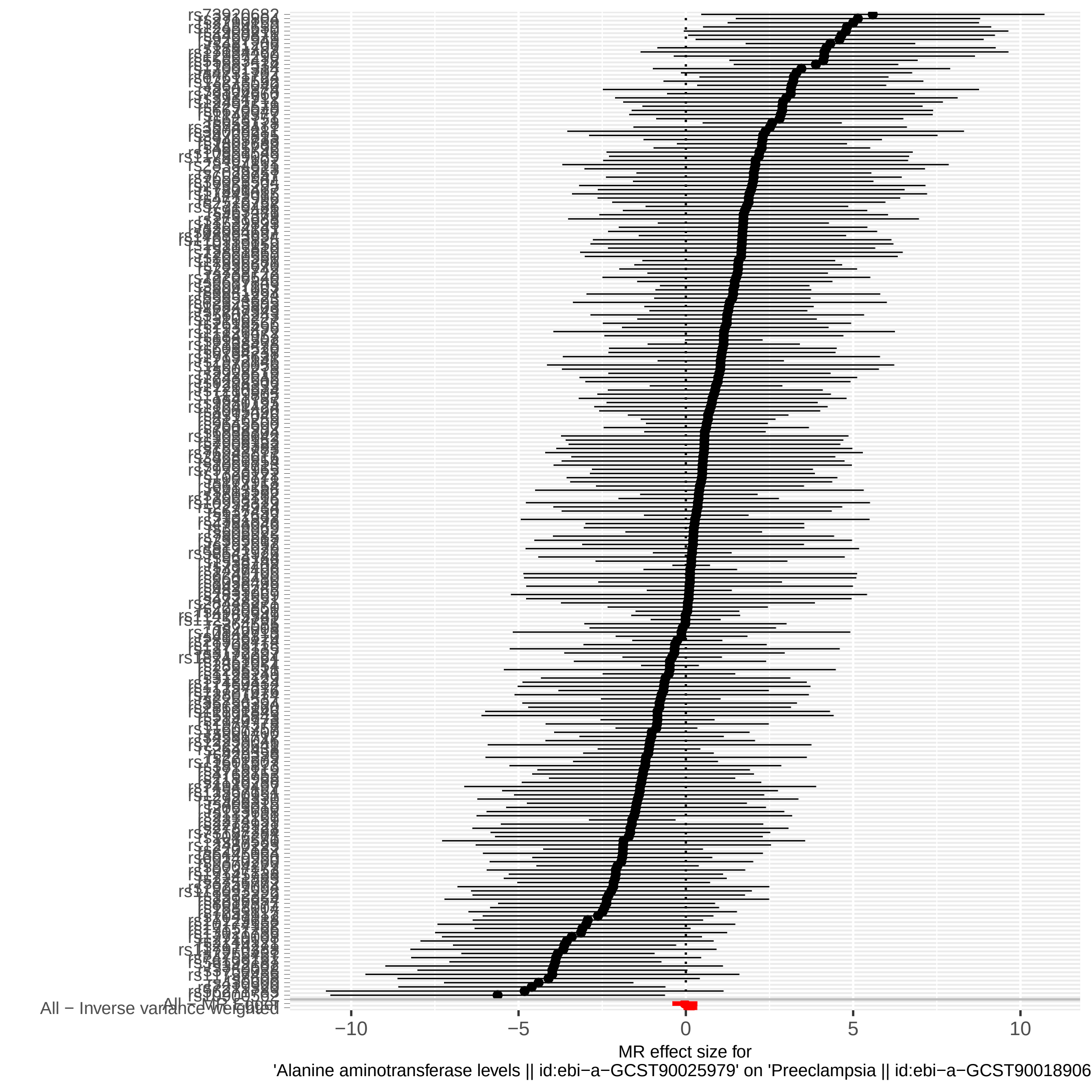

All − Inverse variance weighted
−10
−5
0
5
10
MR effect size for
'Alanine aminotransferase levels || id:ebi−a−GCST90025979' on 'Preeclampsia || id:ebi−a−GCST90018906'

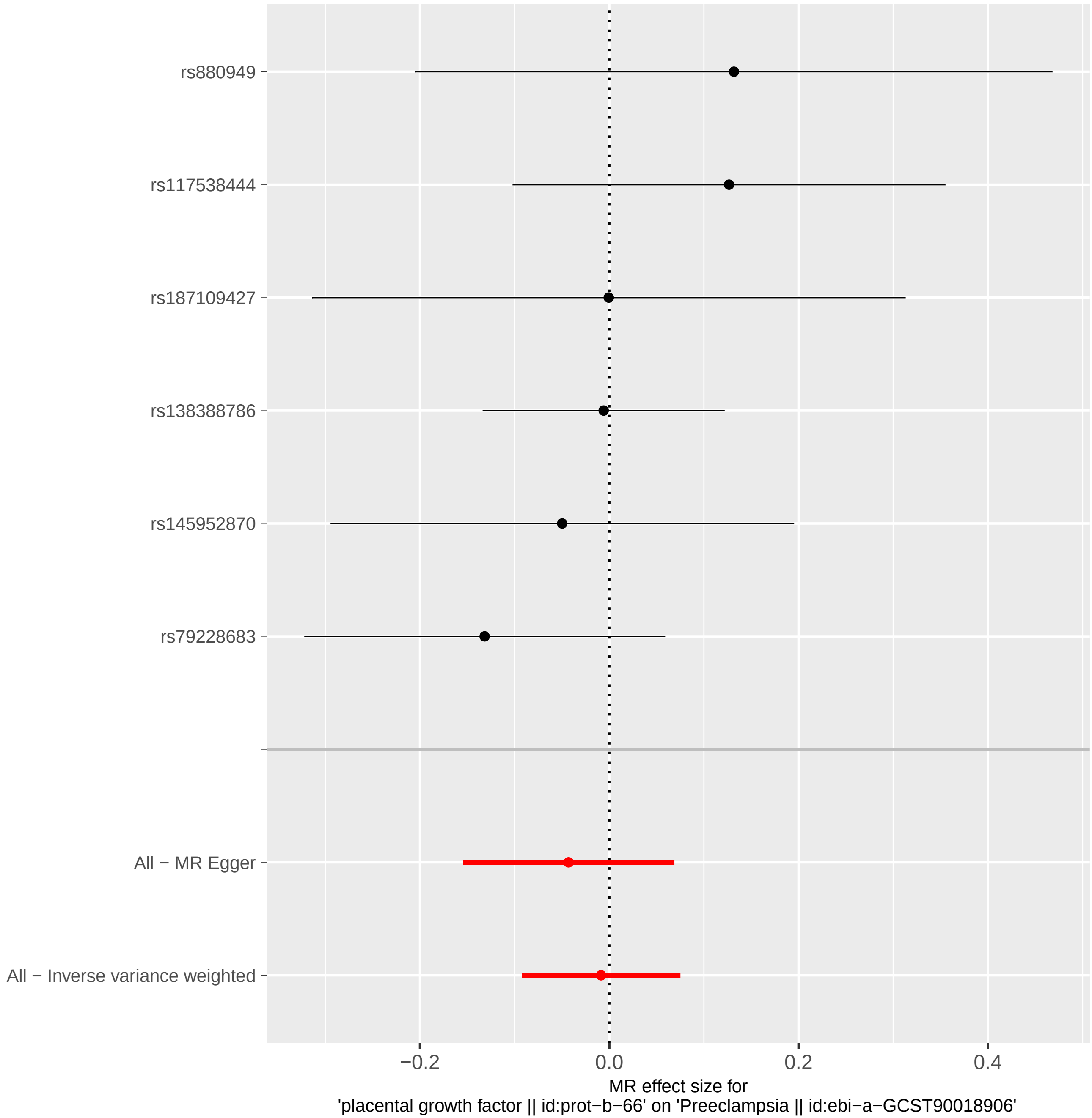
rs880949
rs117538444
rs187109427
rs138388786
rs145952870
rs79228683
All − MR Egger
All − Inverse variance weighted
−0.2
0.0
0.2
0.4
MR effect size for
'placental growth factor || id:prot−b−66' on 'Preeclampsia || id:ebi−a−GCST90018906'

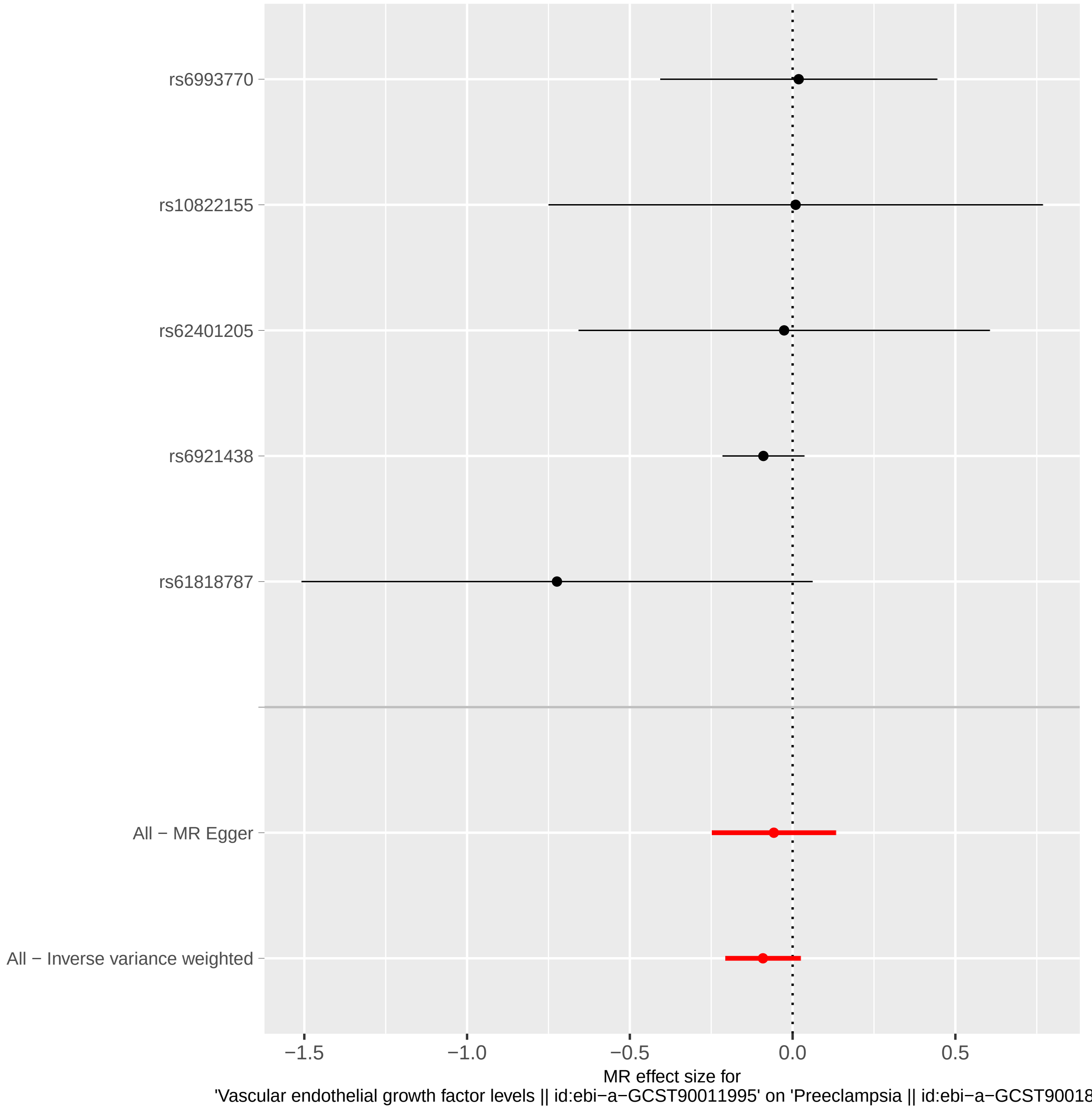
rs6993770
rs10822155
rs62401205
rs6921438
rs61818787
All − MR Egger
All − Inverse variance weighted
−1.5
−1.0
−0.5
0.0
0.5
MR effect size for
'Vascular endothelial growth factor levels || id:ebi−a−GCST90011995' on 'Preeclampsia || id:ebi−a−GCST90018

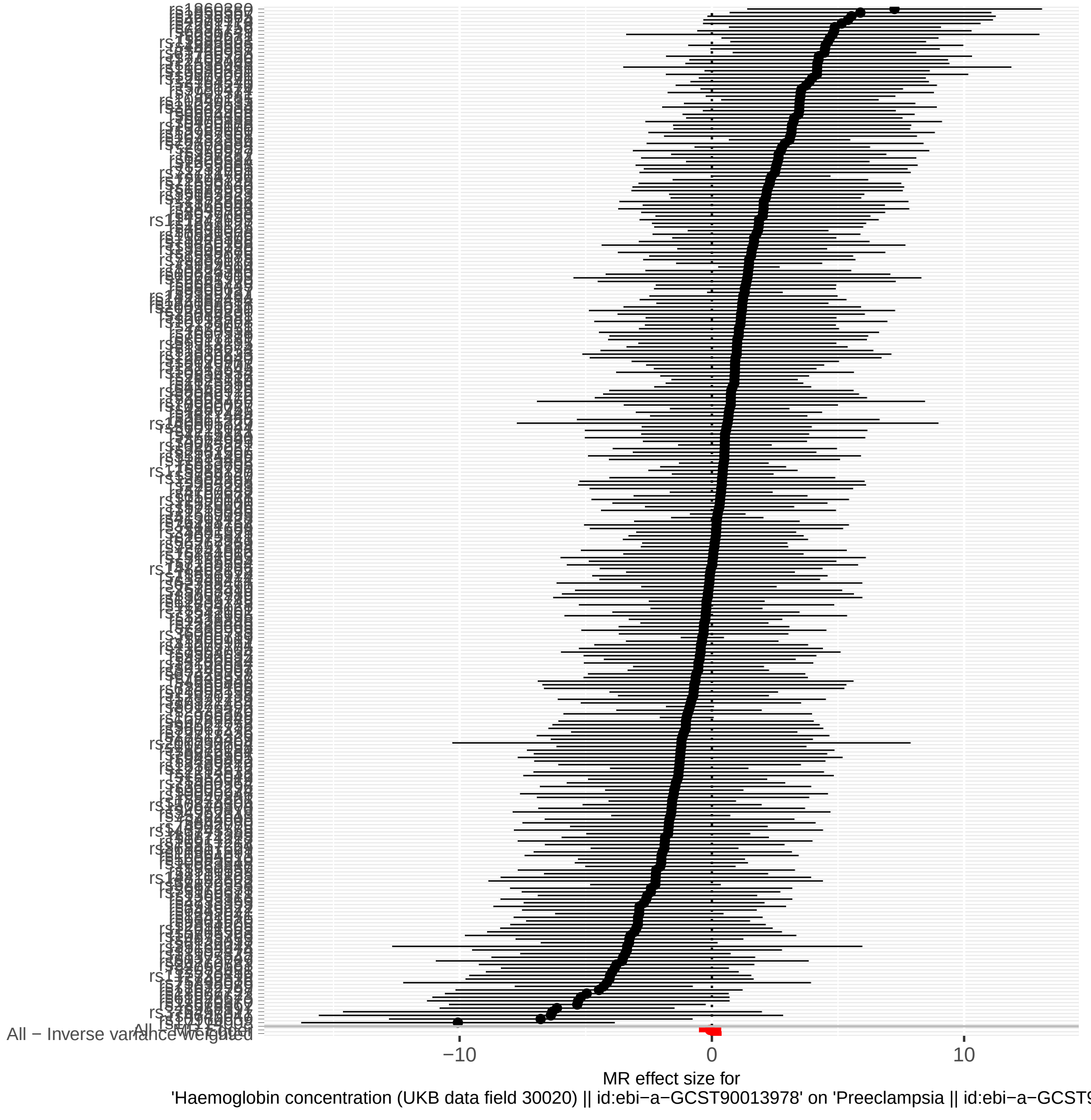

All − Inverse variance weighted
−10
0
10
MR effect size for
'Haemoglobin concentration (UKB data field 30020) || id:ebi−a−GCST90013978' on 'Preeclampsia || id:ebi−a−GCST9

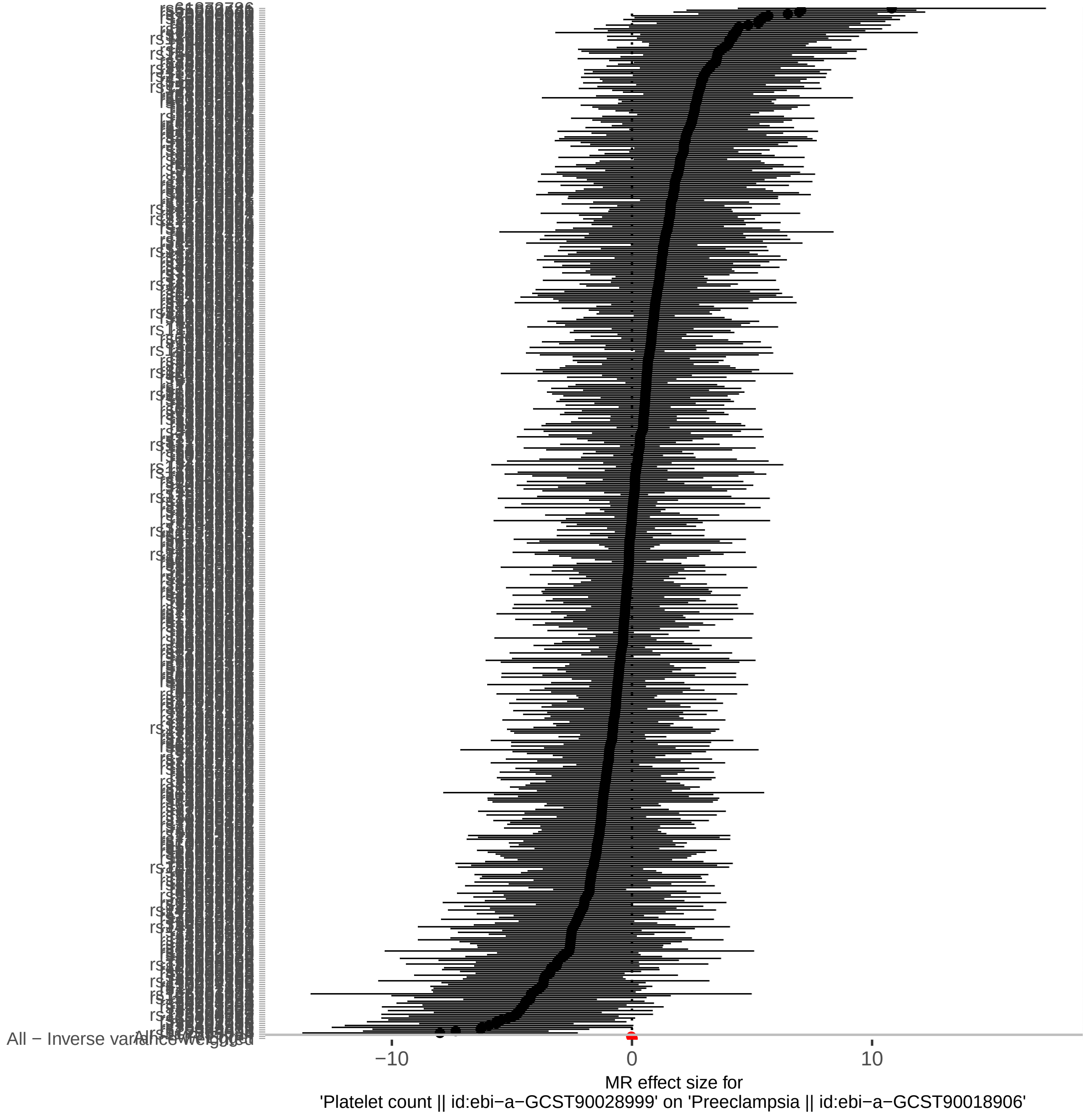

All − Inverse variance weighted
−10
0
10
MR effect size for
'Platelet count || id:ebi−a−GCST90028999' on 'Preeclampsia || id:ebi−a−GCST90018906'
