# Supplementary material for: Causal pathways in preeclampsia: a Mendelian randomization study in European populations
Source: Front Endocrinol (Lausanne). 2024 Sep 2;15:1453277. doi: 10.3389/fendo.2024.1453277 (PMC11402816; doi:10.3389/fendo.2024.1453277)
Supplement: Supplementary file 2 [file DataSheet2.pdf]

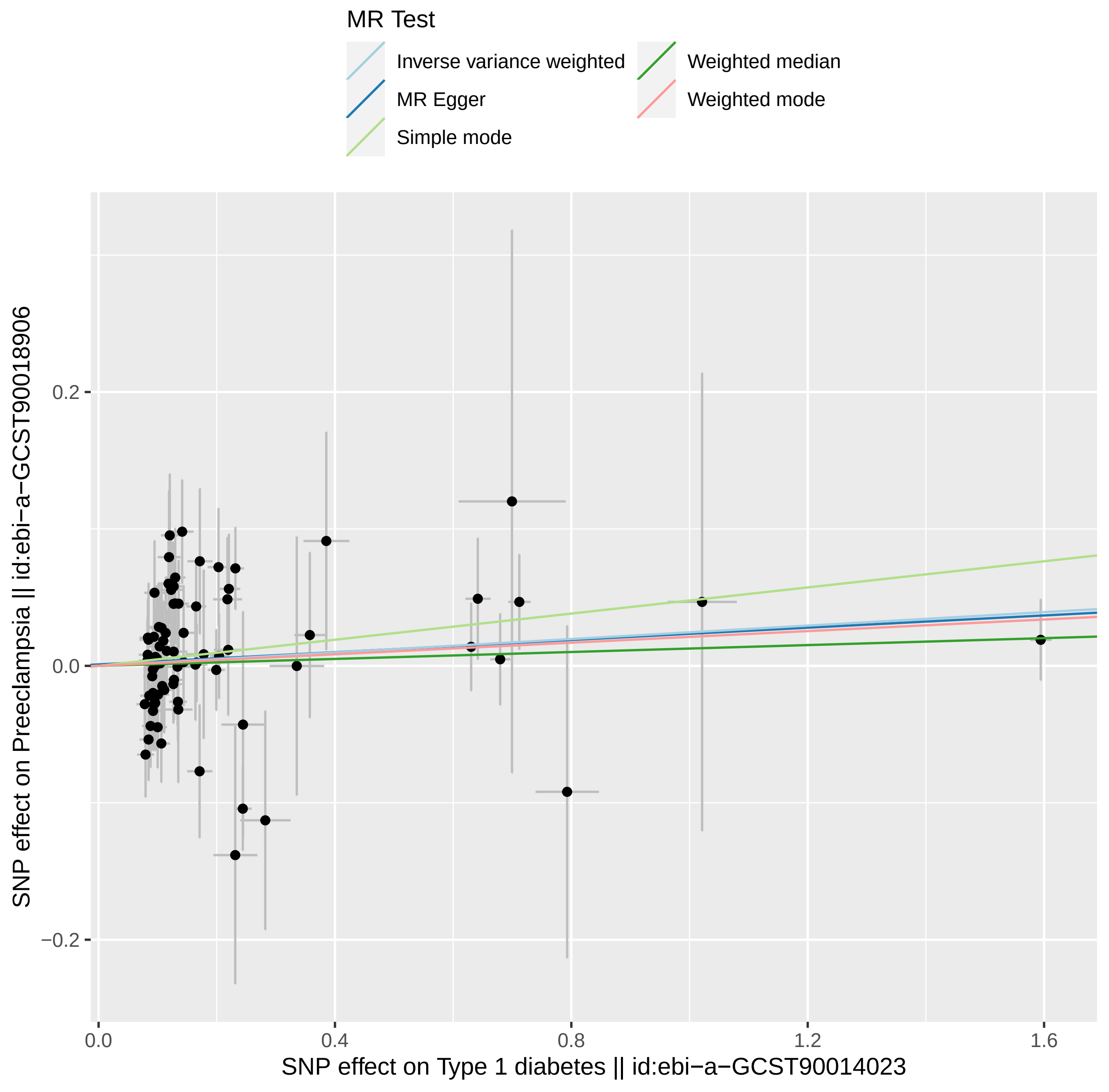
MR Test
Inverse variance weighted
MR Egger
Simple mode
Weighted median
Weighted mode
SNP effect on Preeclampsia || id:ebi−a−GCST90018906
0.2
0.0
−0.2
0.0
0.4
0.8
1.2
1.6
SNP effect on Type 1 diabetes || id:ebi−a−GCST90014023

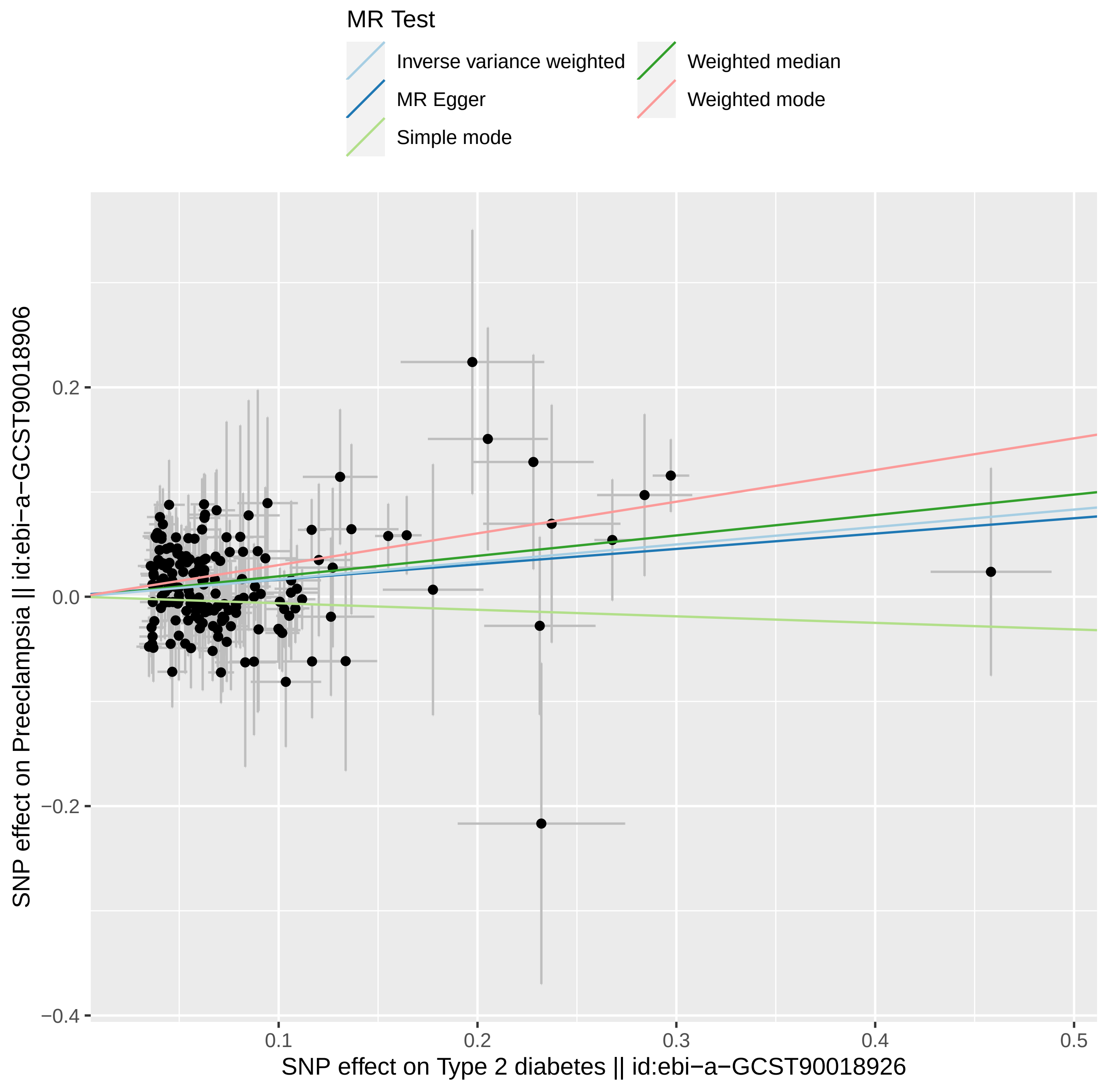

MR Test
Inverse variance weighted
MR Egger
Simple mode
Weighted median
Weighted mode
0.2
0.0
−0.2
−0.4
0.1
0.2
0.3
0.4
0.5
SNP effect on Preeclampsia || id:ebi−a−GCST90018906
SNP effect on Type 2 diabetes || id:ebi−a−GCST90018926

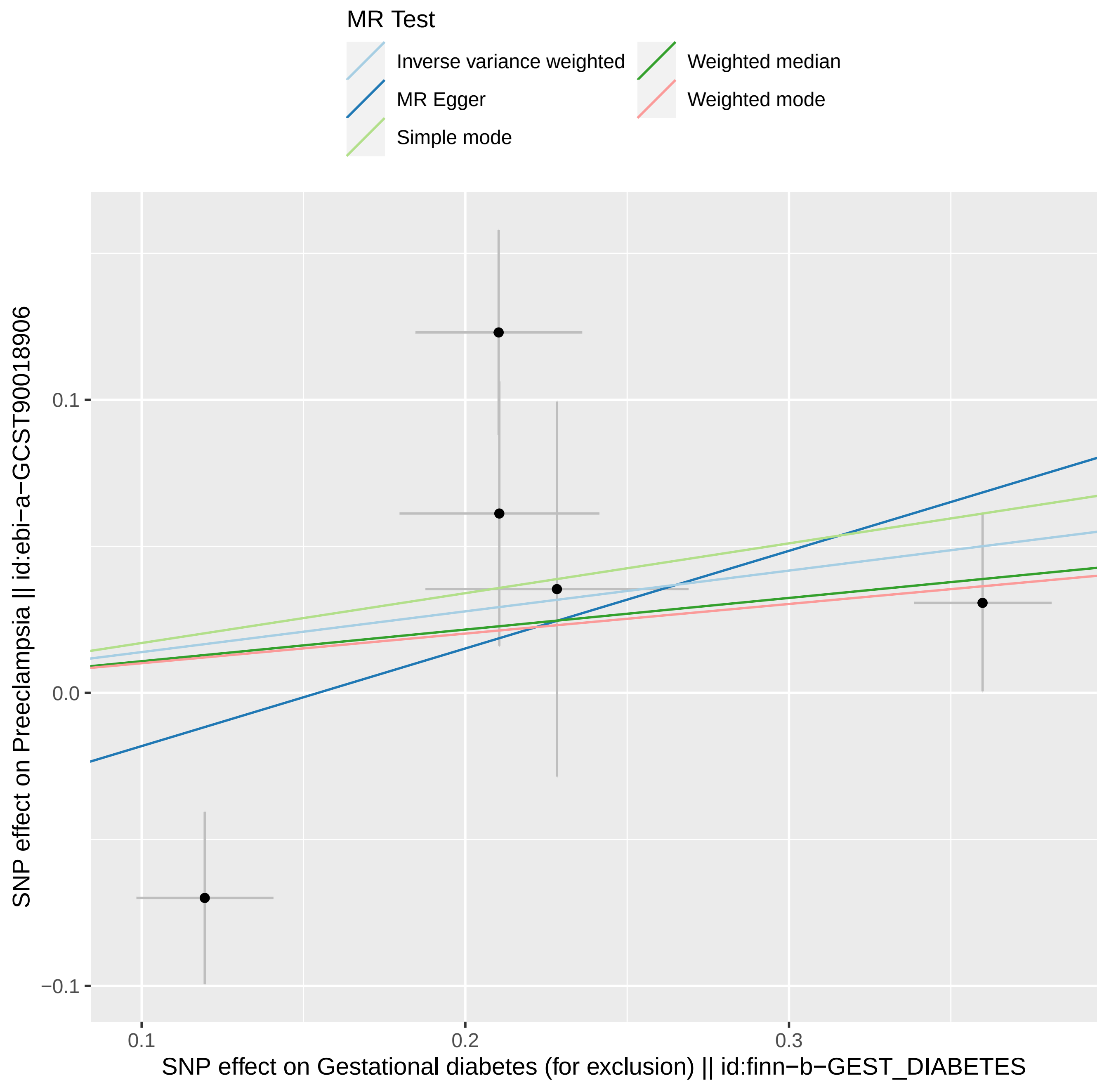
MR Test
Inverse variance weighted
MR Egger
Simple mode
Weighted median
Weighted mode
SNP effect on Preeclampsia || id:ebi−a−GCST90018906
0.1
0.0
−0.1
0.1
0.2
0.3
SNP effect on Gestational diabetes (for exclusion) || id:finn−b−GEST_DIABETES

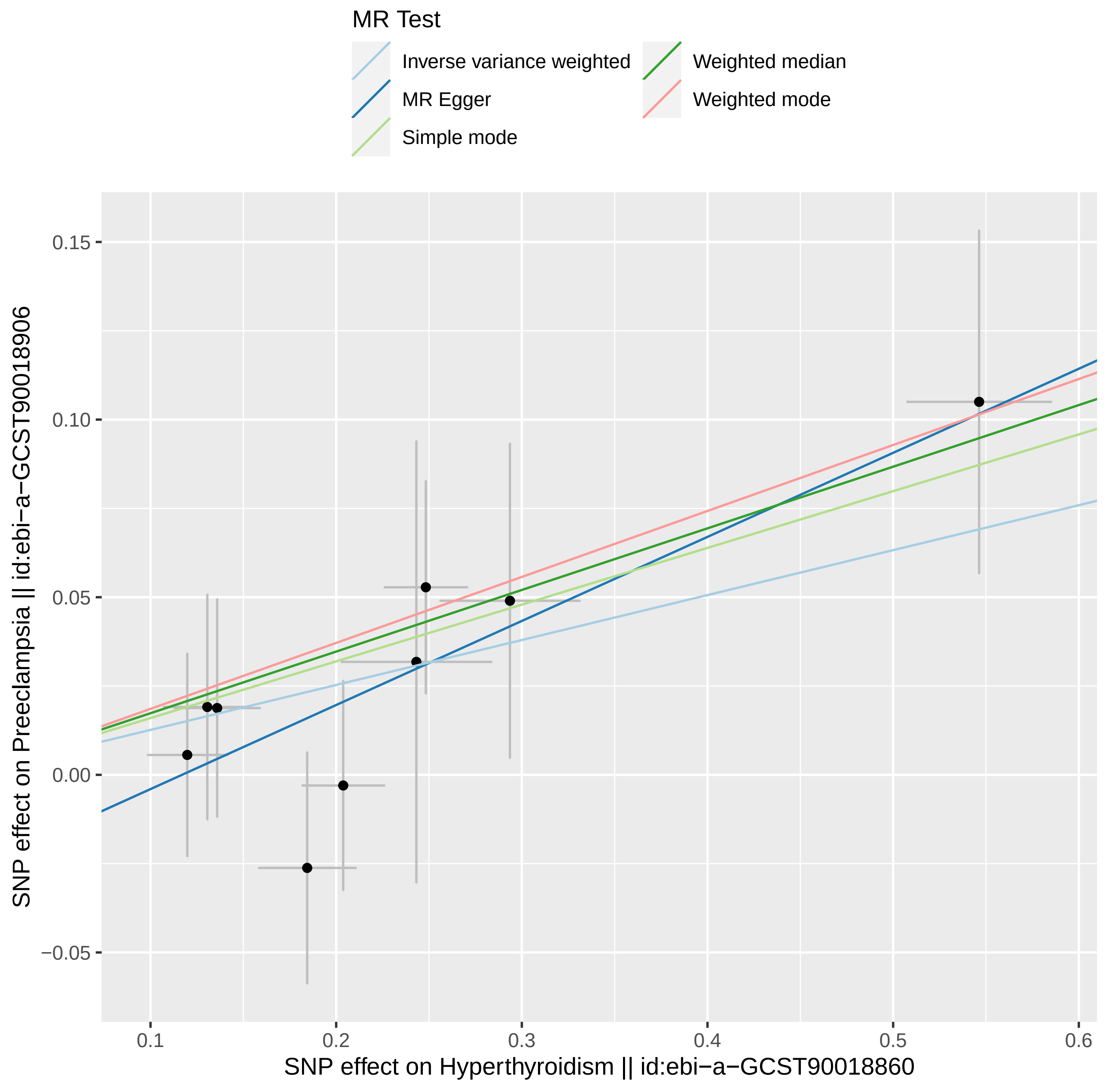
MR Test
Inverse variance weighted
MR Egger
Simple mode
Weighted median
Weighted mode
SNP effect on Preeclampsia || id:ebi−a−GCST90018906
SNP effect on Hyperthyroidism || id:ebi−a−GCST90018860
0.15
0.10
0.05
0.00
−0.05
0.1
0.2
0.3
0.4
0.5
0.6

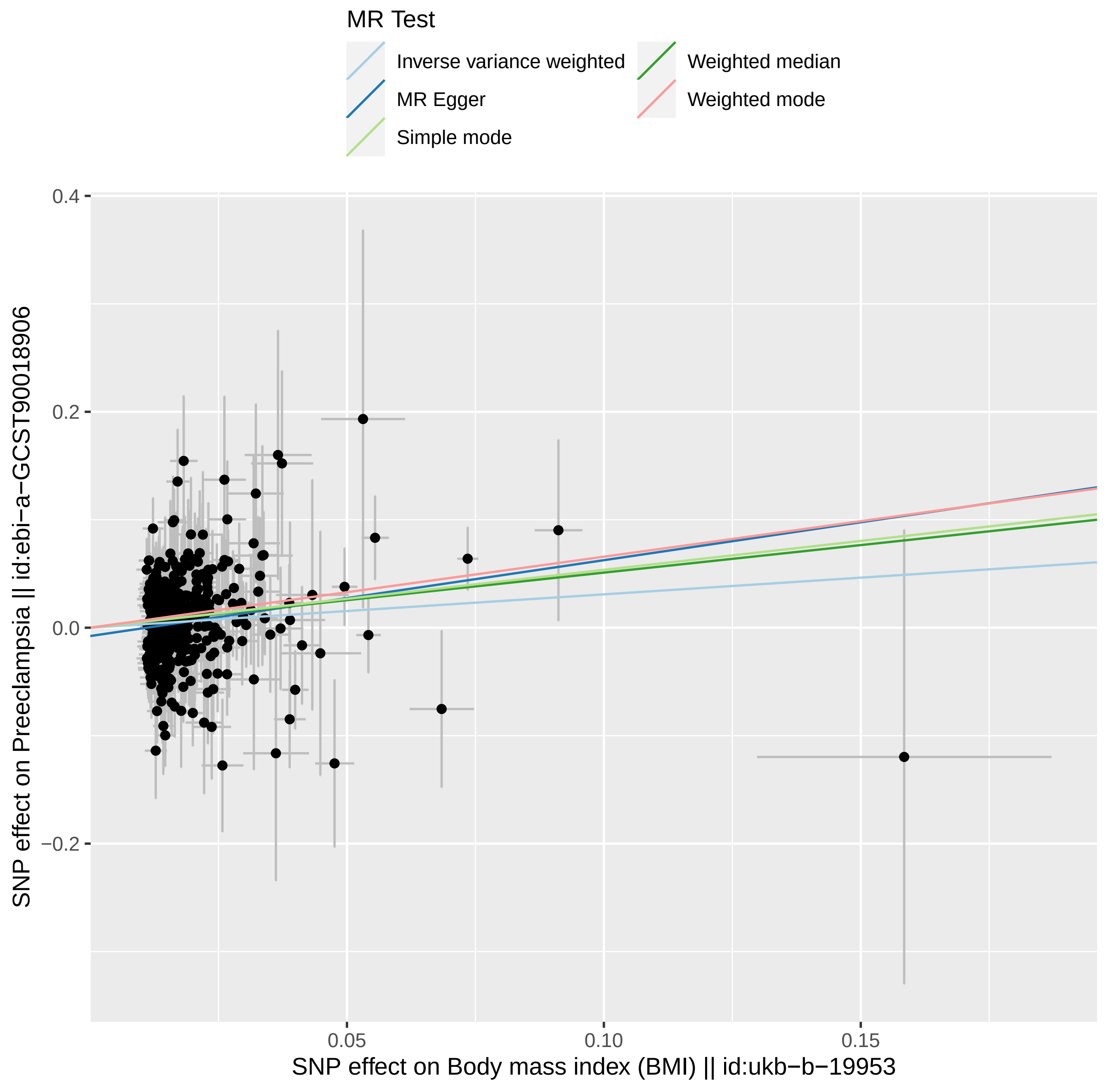
MR Test
Inverse variance weighted
MR Egger
Simple mode
Weighted median
Weighted mode
0.4
0.2
0.0
−0.2
0.05
0.10
0.15
SNP effect on Preeclampsia || id:ebi−a−GCST90018906
SNP effect on Body mass index (BMI) || id:ukb−b−19953

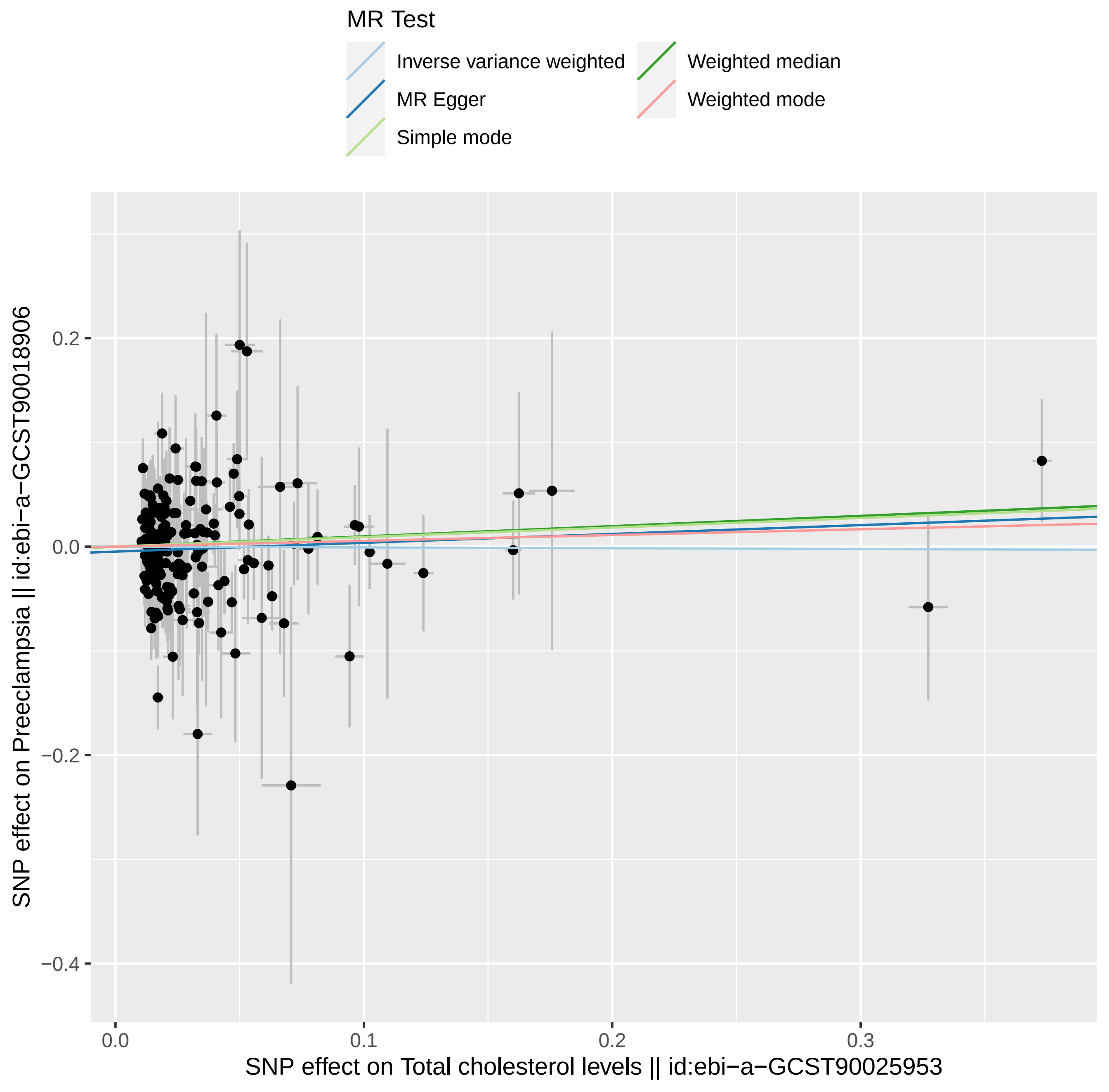
MR Test
Inverse variance weighted
MR Egger
Simple mode
Weighted median
Weighted mode
0.2
0.0
−0.2
−0.4
0.0
0.1
0.2
0.3
SNP effect on Preeclampsia || id:ebi−a−GCST90018906
SNP effect on Total cholesterol levels || id:ebi−a−GCST90025953

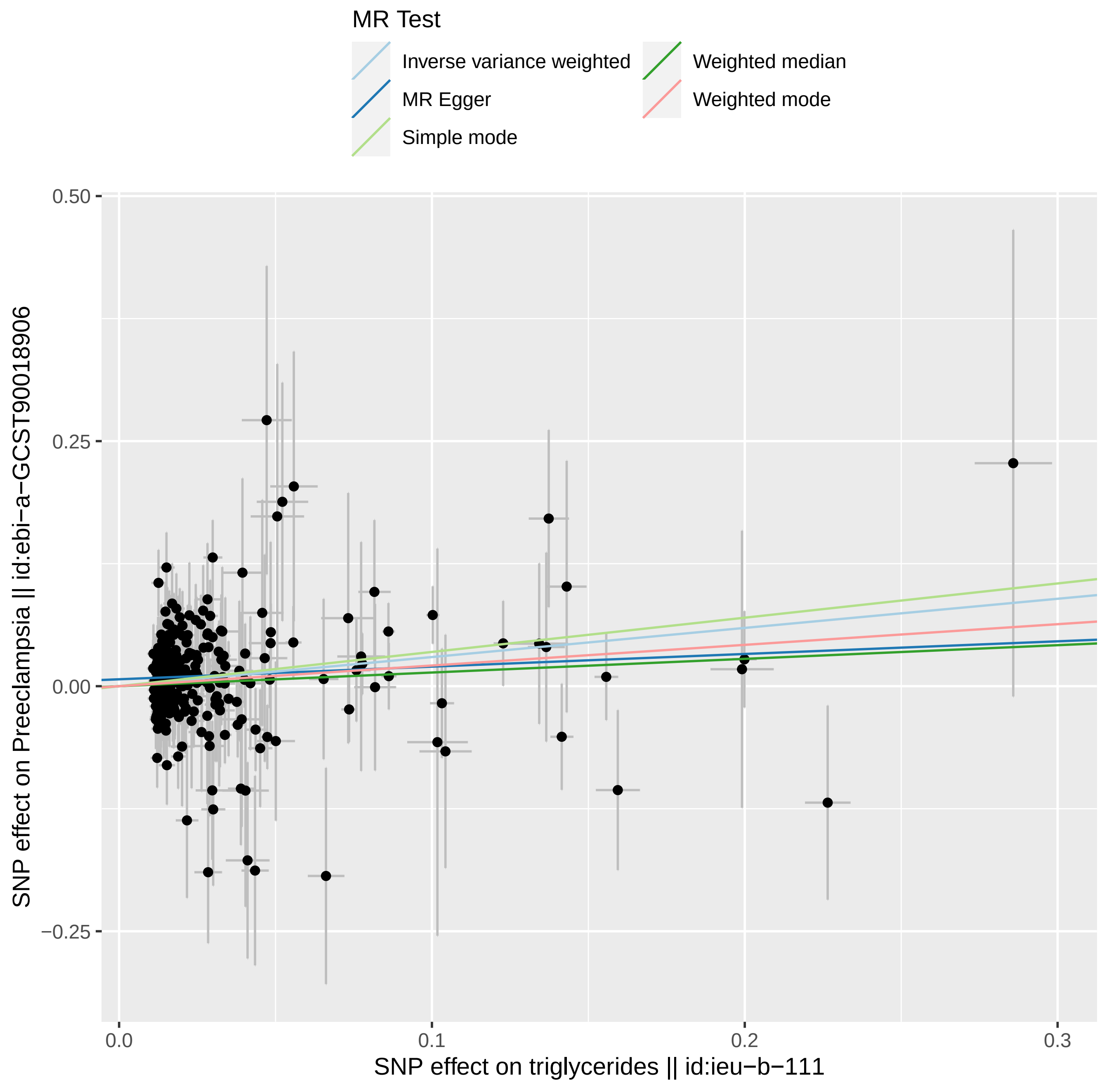
MR Test
Inverse variance weighted
MR Egger
Simple mode
Weighted median
Weighted mode
0.50
0.25
0.00
−0.25
SNP effect on Preeclampsia || id:ebi−a−GCST90018906
0.0
0.1
0.2
0.3
SNP effect on triglycerides || id:ieu−b−111

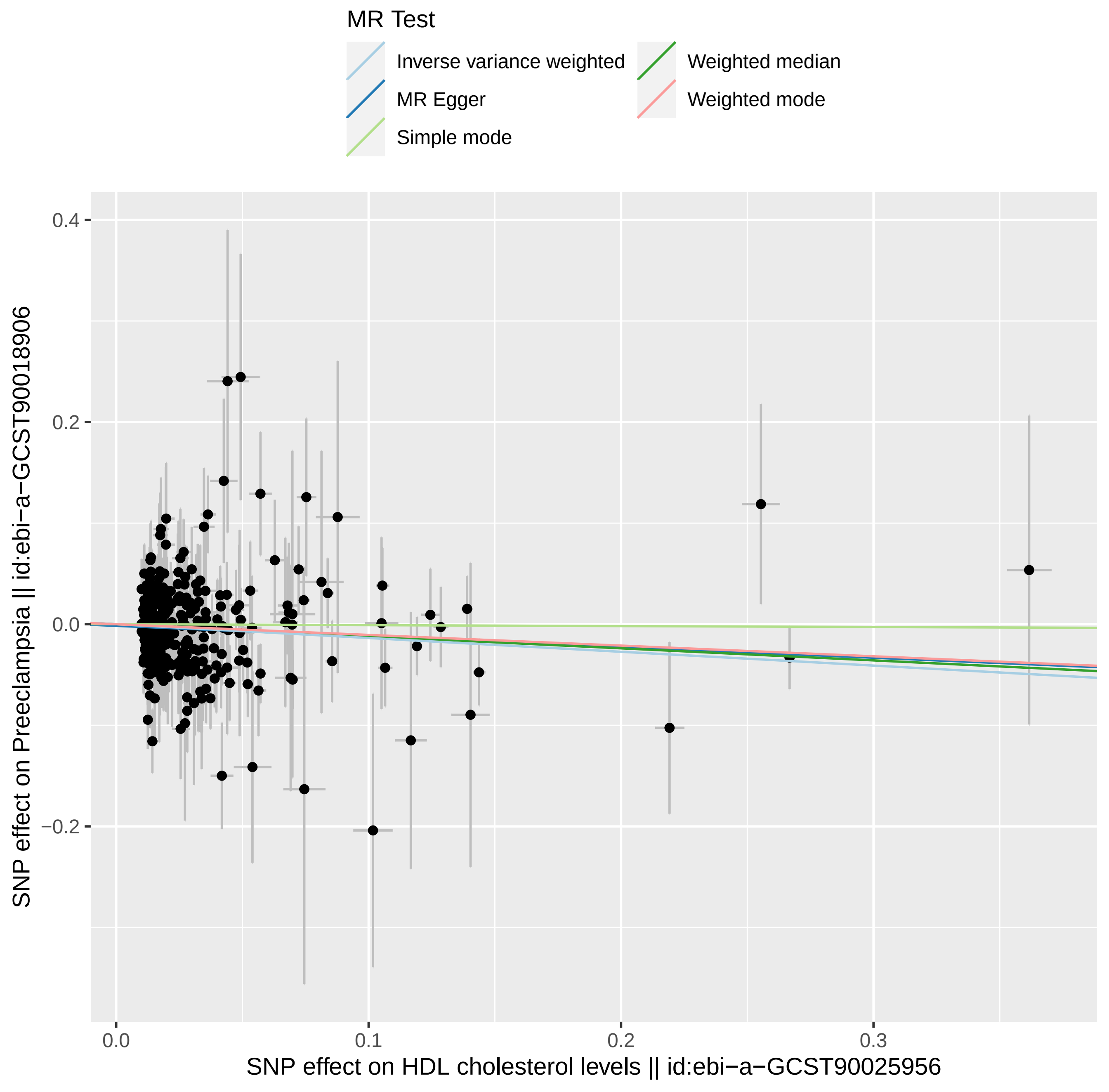

MR Test
Inverse variance weighted
MR Egger
Simple mode
Weighted median
Weighted mode
0.4
0.2
0.0
−0.2
0.0
0.1
0.2
0.3
SNP effect on Preeclampsia || id:ebi−a−GCST90018906
SNP effect on HDL cholesterol levels || id:ebi−a−GCST90025956

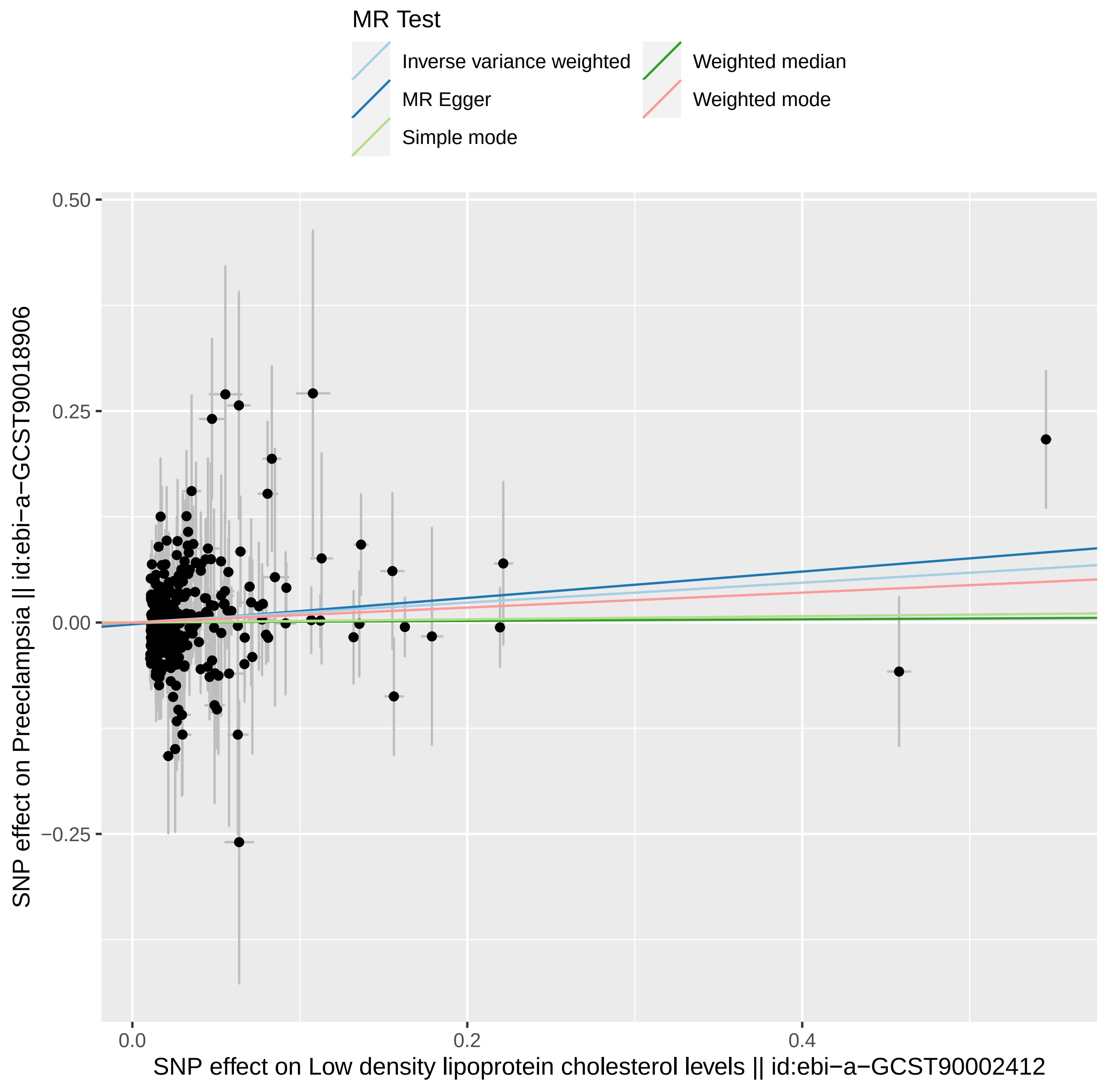
MR Test
Inverse variance weighted
MR Egger
Simple mode
Weighted median
Weighted mode
0.50
0.25
0.00
−0.25
SNP effect on Preeclampsia || id:ebi−a−GCST90018906
0.0
0.2
0.4
SNP effect on Low density lipoprotein cholesterol levels || id:ebi−a−GCST90002412

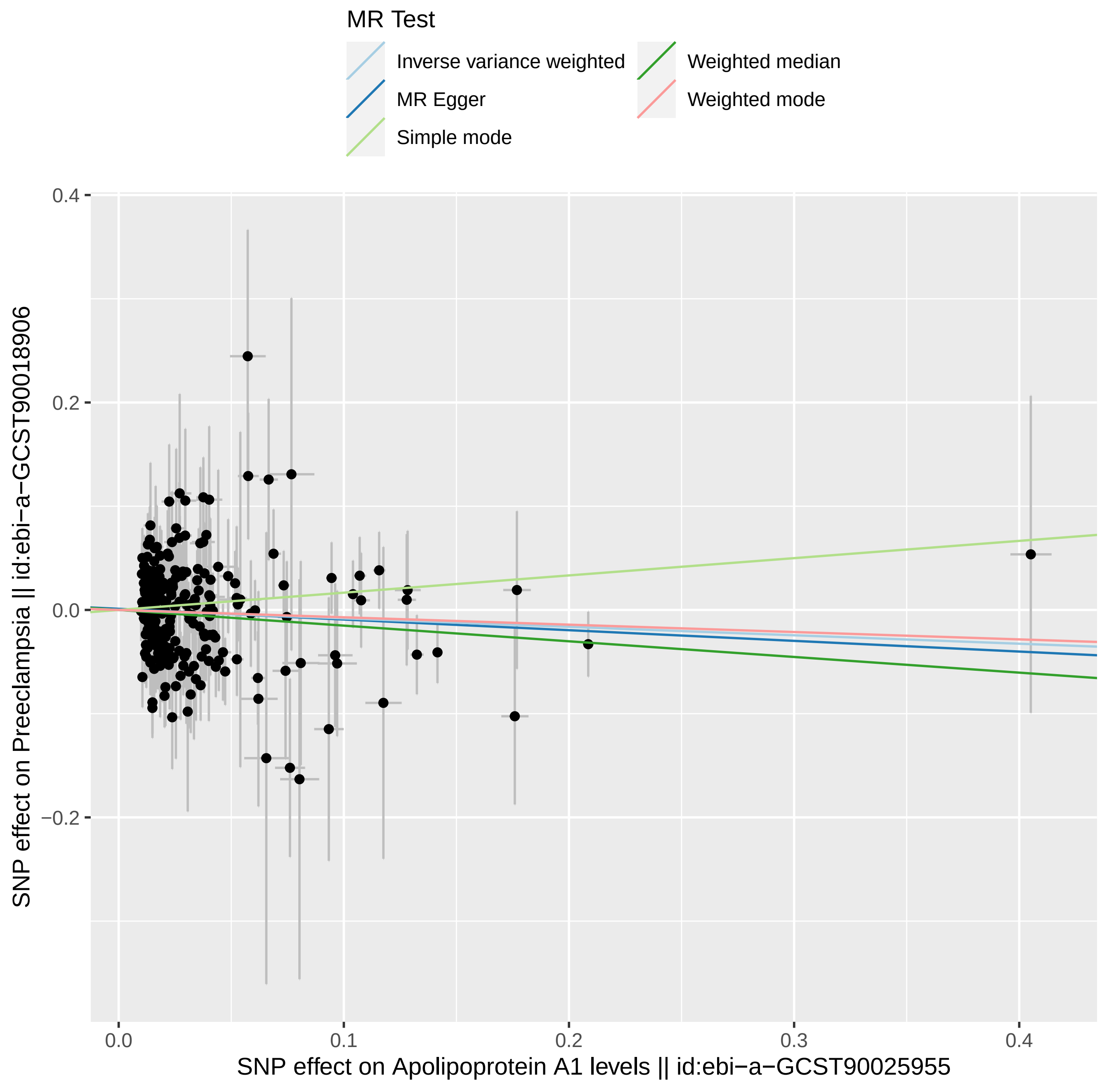

MR Test
Inverse variance weighted
MR Egger
Simple mode
Weighted median
Weighted mode
0.4
0.2
0.0
−0.2
SNP effect on Preeclampsia || id:ebi−a−GCST90018906
0.0
0.1
0.2
0.3
0.4
SNP effect on Apolipoprotein A1 levels || id:ebi−a−GCST90025955

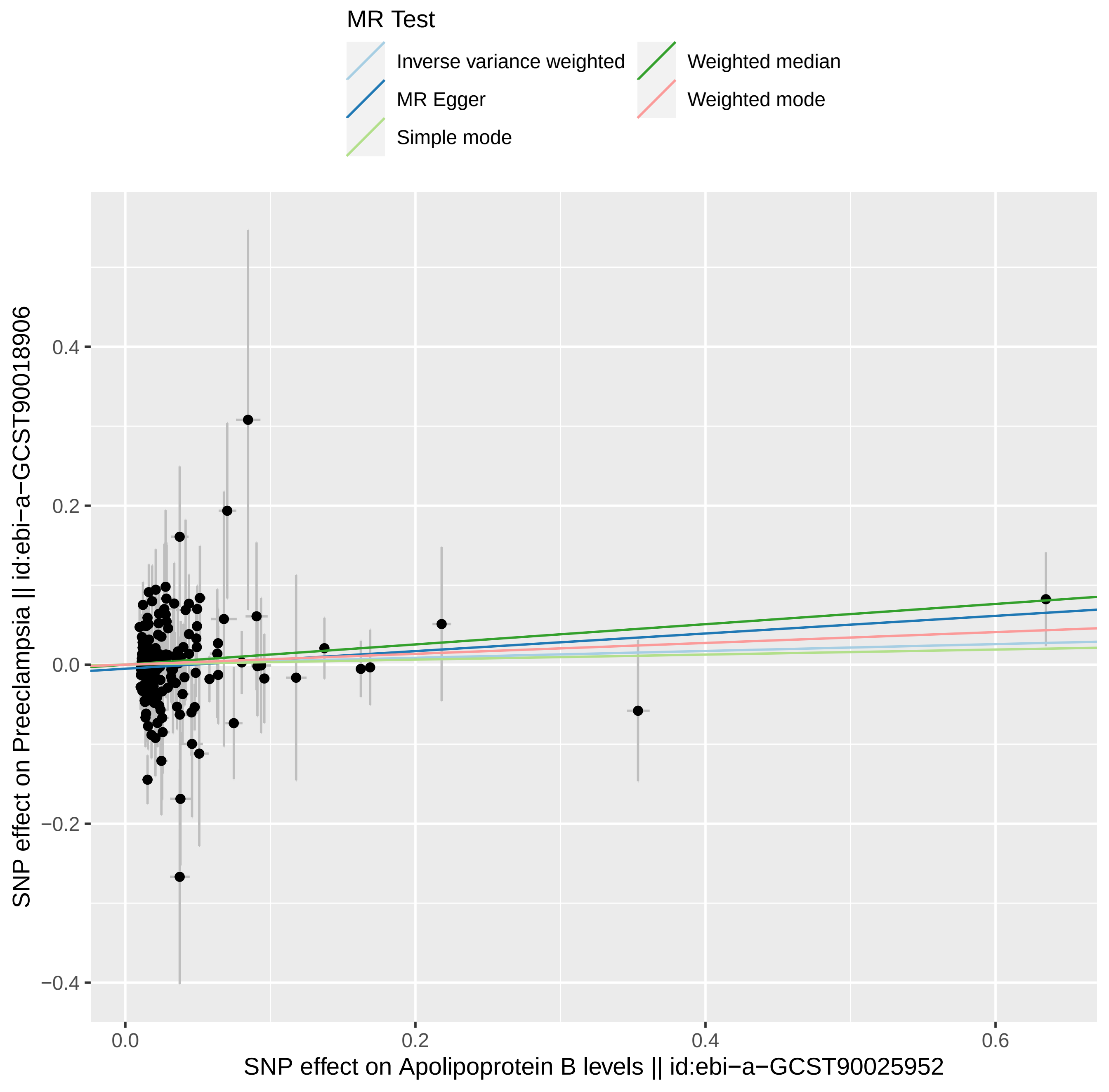

MR Test
Inverse variance weighted
MR Egger
Simple mode
Weighted median
Weighted mode
SNP effect on Preeclampsia || id:ebi−a−GCST90018906
SNP effect on Apolipoprotein B levels || id:ebi−a−GCST90025952
0.4
0.2
0.0
−0.2
−0.4
0.0
0.2
0.4
0.6

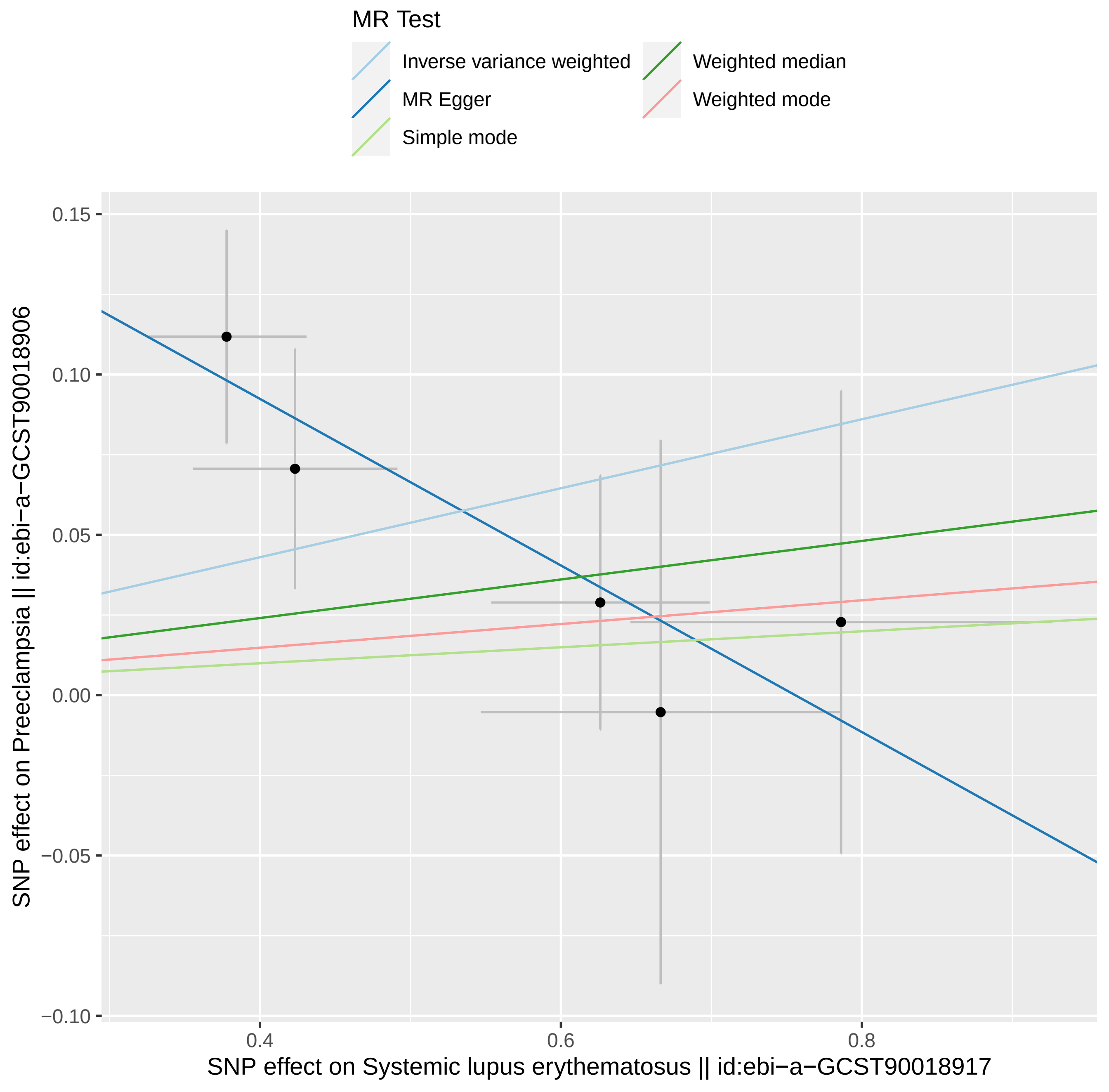

MR Test
Inverse variance weighted
MR Egger
Simple mode
Weighted median
Weighted mode
0.15
0.10
0.05
0.00
−0.05
−0.10
0.4
0.6
0.8
SNP effect on Preeclampsia || id:ebi−a−GCST90018906
SNP effect on Systemic lupus erythematosus || id:ebi−a−GCST90018917

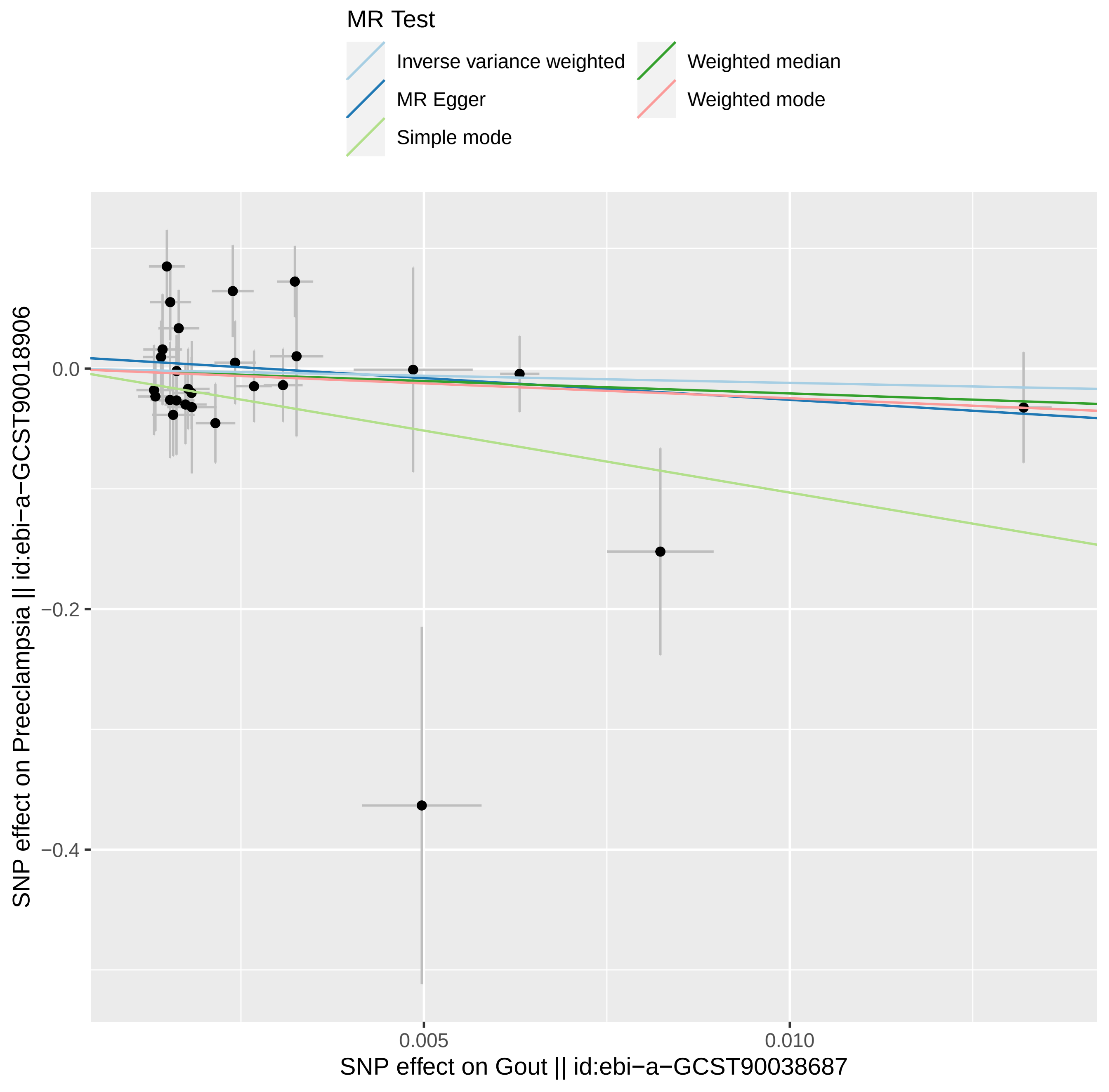

MR Test
Inverse variance weighted
MR Egger
Simple mode
Weighted median
Weighted mode
0.0
−0.2
−0.4
0.005
0.010
SNP effect on Gout || id:ebi−a−GCST90038687
SNP effect on Preeclampsia || id:ebi−a−GCST90018906

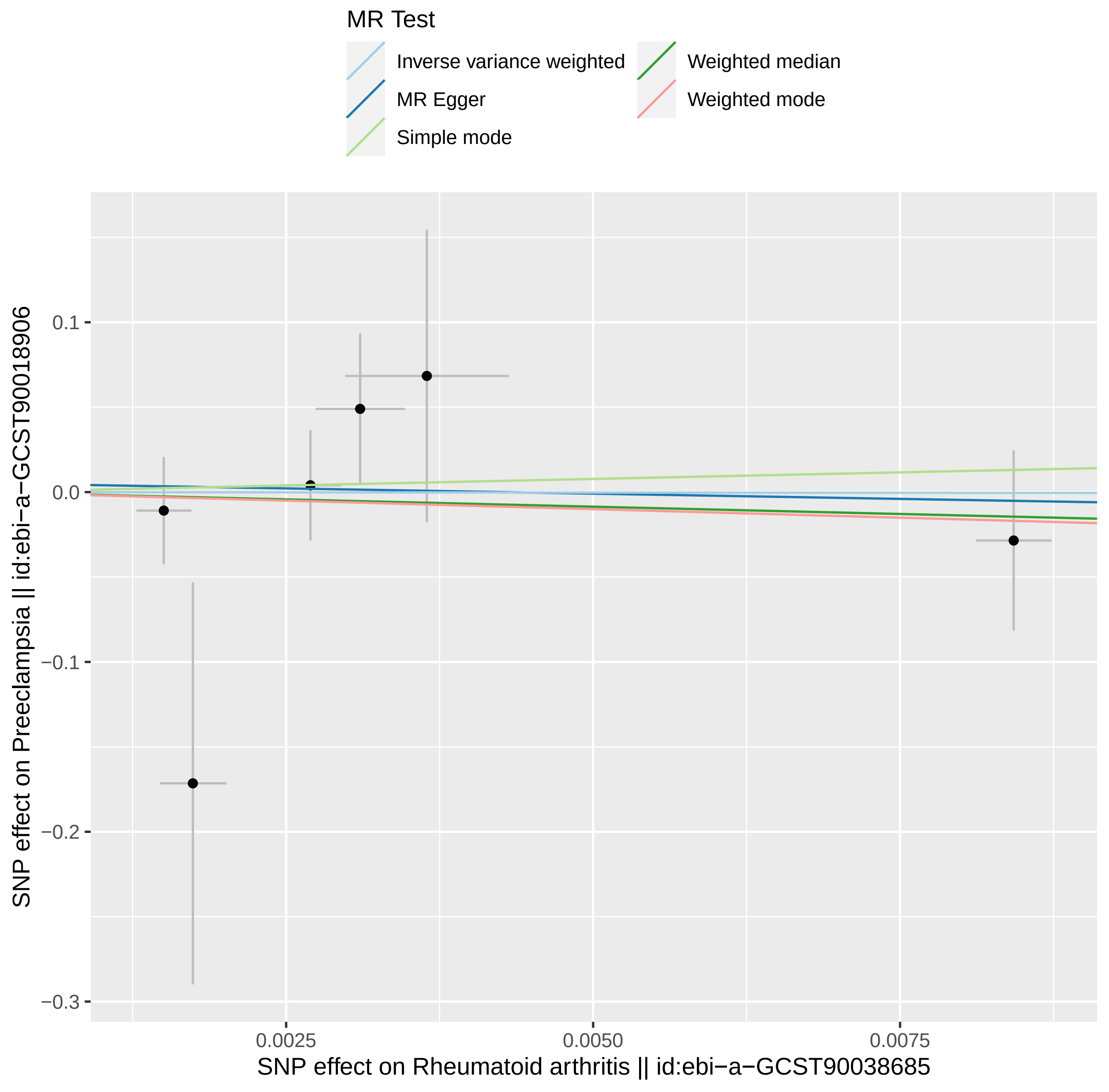
MR Test
Inverse variance weighted
MR Egger
Simple mode
Weighted median
Weighted mode
SNP effect on Preeclampsia || id:ebi−a−GCST90018906
0.1
0.0
−0.1
−0.2
−0.3
0.0025
0.0050
0.0075
SNP effect on Rheumatoid arthritis || id:ebi−a−GCST90038685

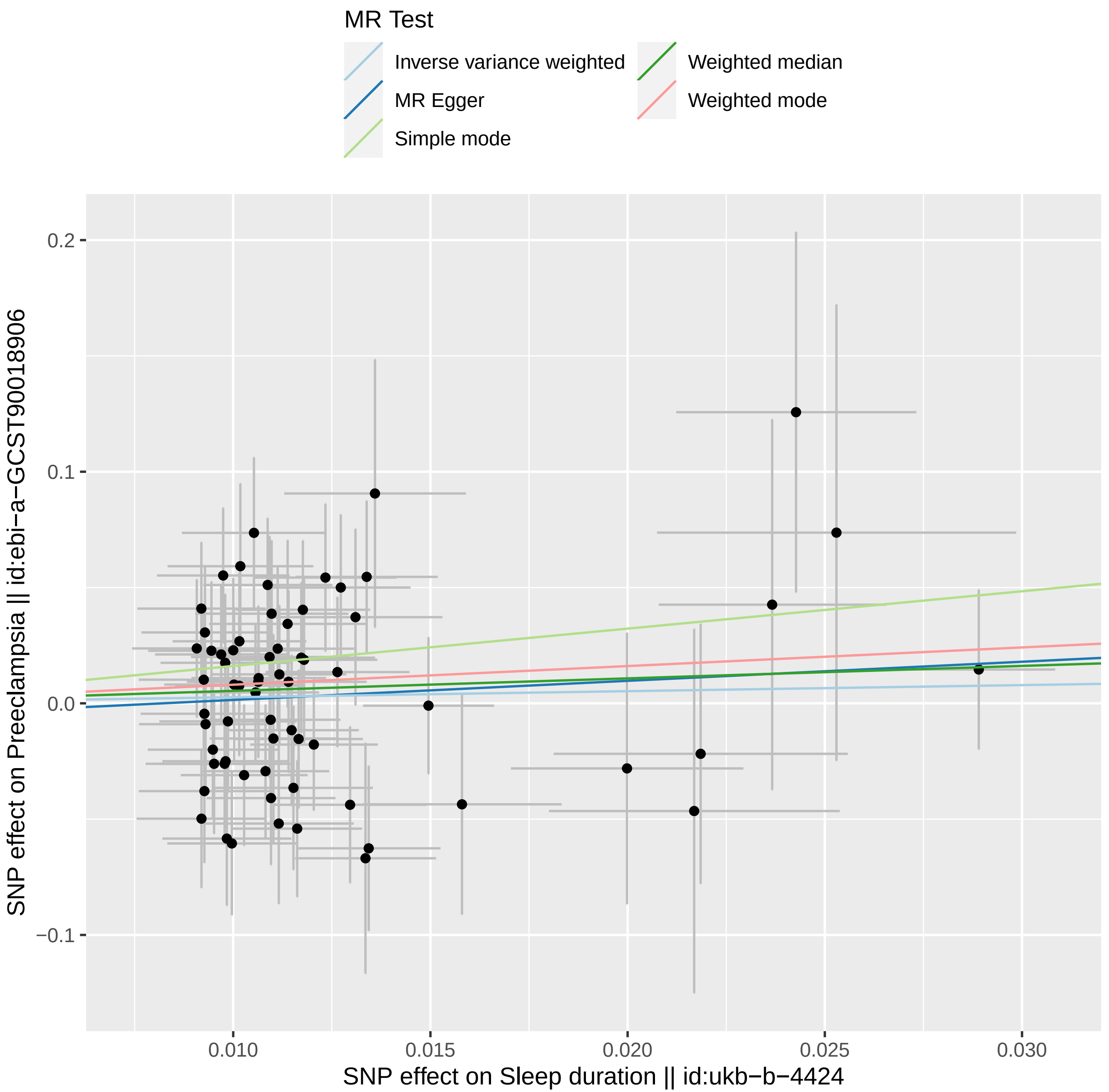
MR Test
Inverse variance weighted
MR Egger
Simple mode
Weighted median
Weighted mode
SNP effect on Preeclampsia || id:ebi−a−GCST90018906
SNP effect on Sleep duration || id:ukb−b−4424
0.2
0.1
0.0
−0.1
0.010
0.015
0.020
0.025
0.030

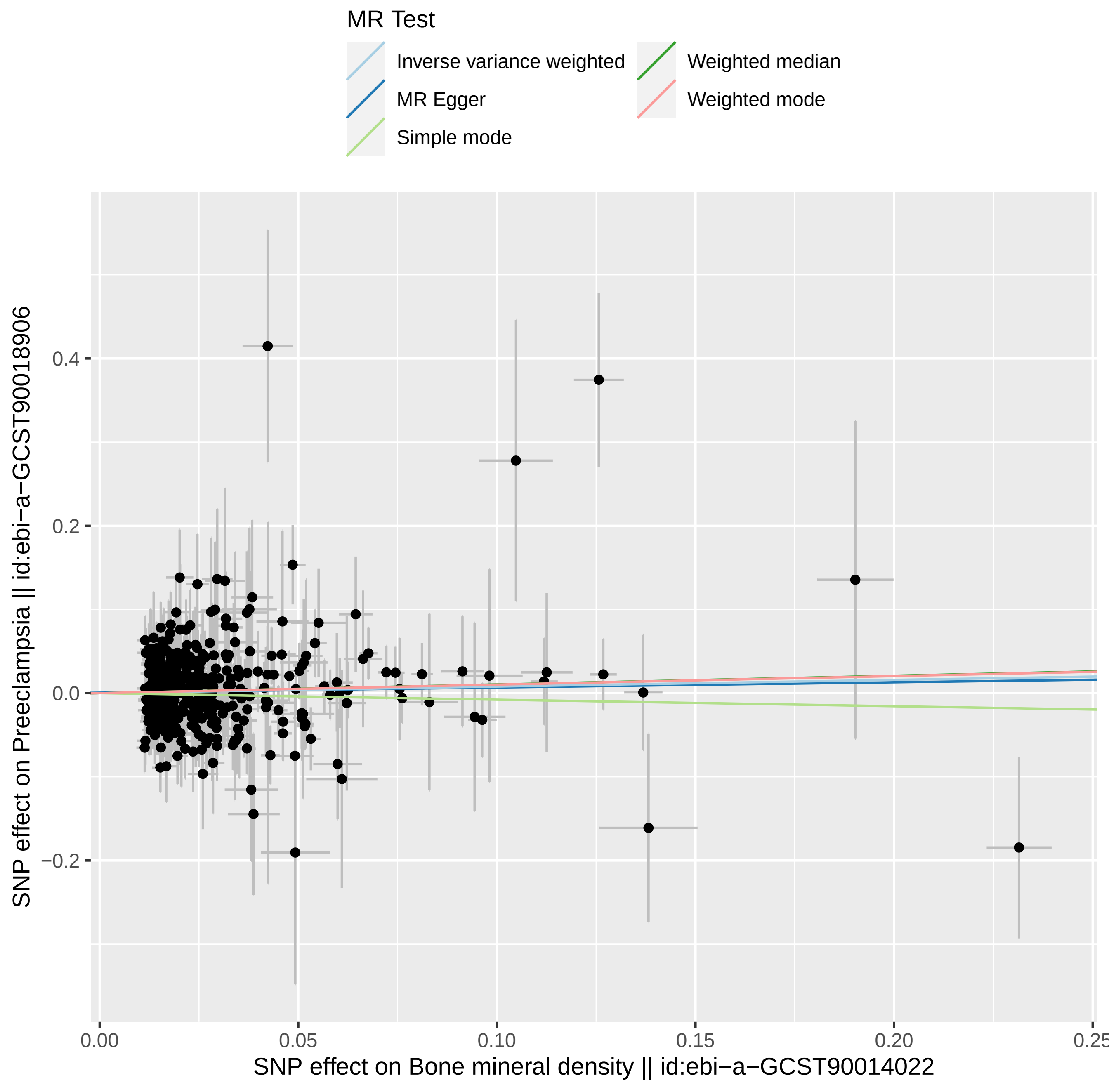

MR Test
Inverse variance weighted
MR Egger
Simple mode
Weighted median
Weighted mode
SNP effect on Preeclampsia || id:ebi−a−GCST90018906
0.4
0.2
0.0
−0.2
0.00
0.05
0.10
0.15
0.20
SNP effect on Bone mineral density || id:ebi−a−GCST90014022

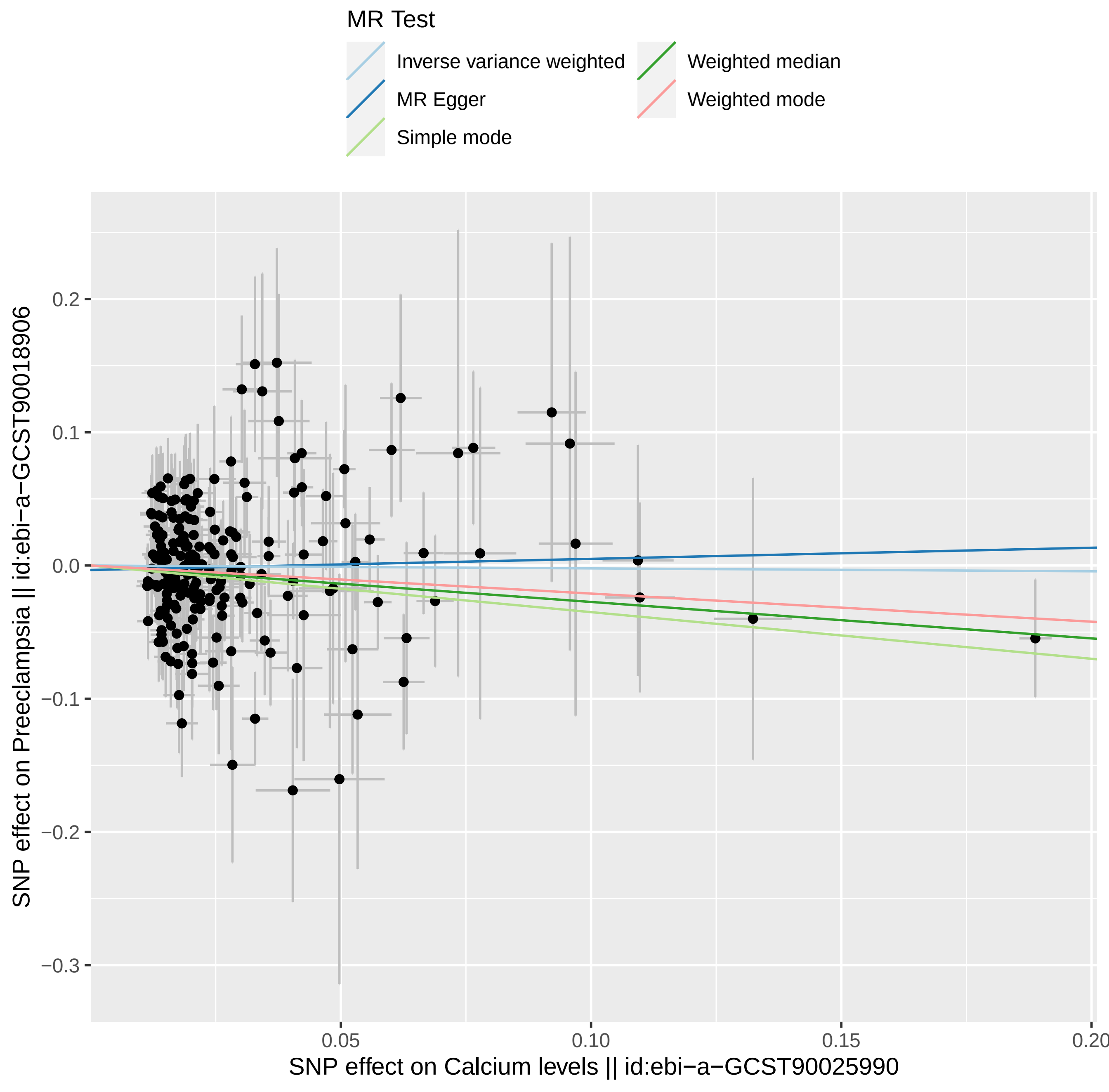

MR Test
Inverse variance weighted
MR Egger
Simple mode
Weighted median
Weighted mode
0.2
0.1
0.0
−0.1
−0.2
−0.3
0.05
0.10
0.15
0.20
SNP effect on Preeclampsia || id:ebi−a−GCST90018906
SNP effect on Calcium levels || id:ebi−a−GCST90025990

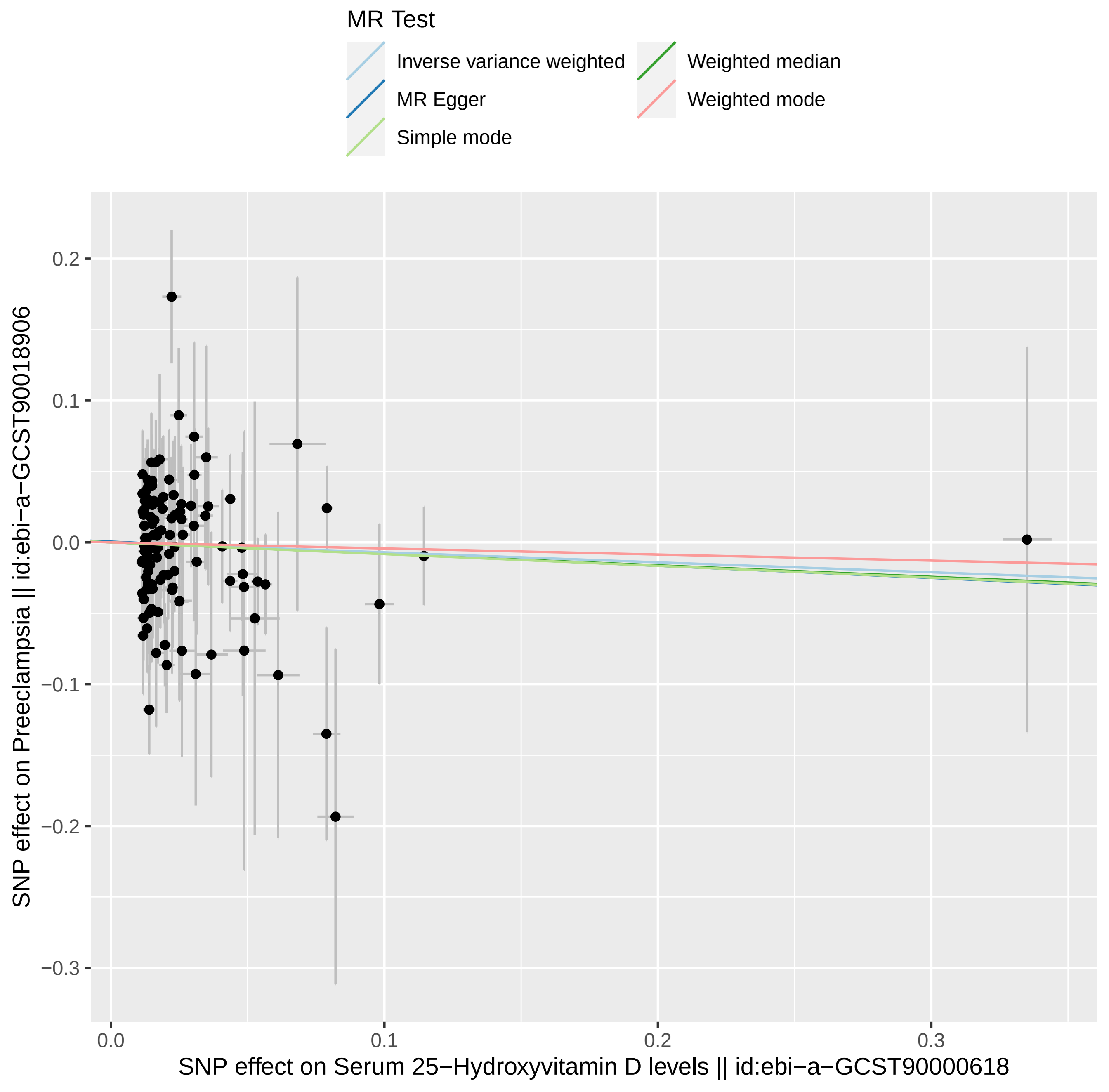

MR Test
Inverse variance weighted
MR Egger
Simple mode
Weighted median
Weighted mode
SNP effect on Preeclampsia || id:ebi−a−GCST90018906
SNP effect on Serum 25−Hydroxyvitamin D levels || id:ebi−a−GCST90000618
0.2
0.1
0.0
−0.1
−0.2
−0.3
0.0
0.1
0.2
0.3

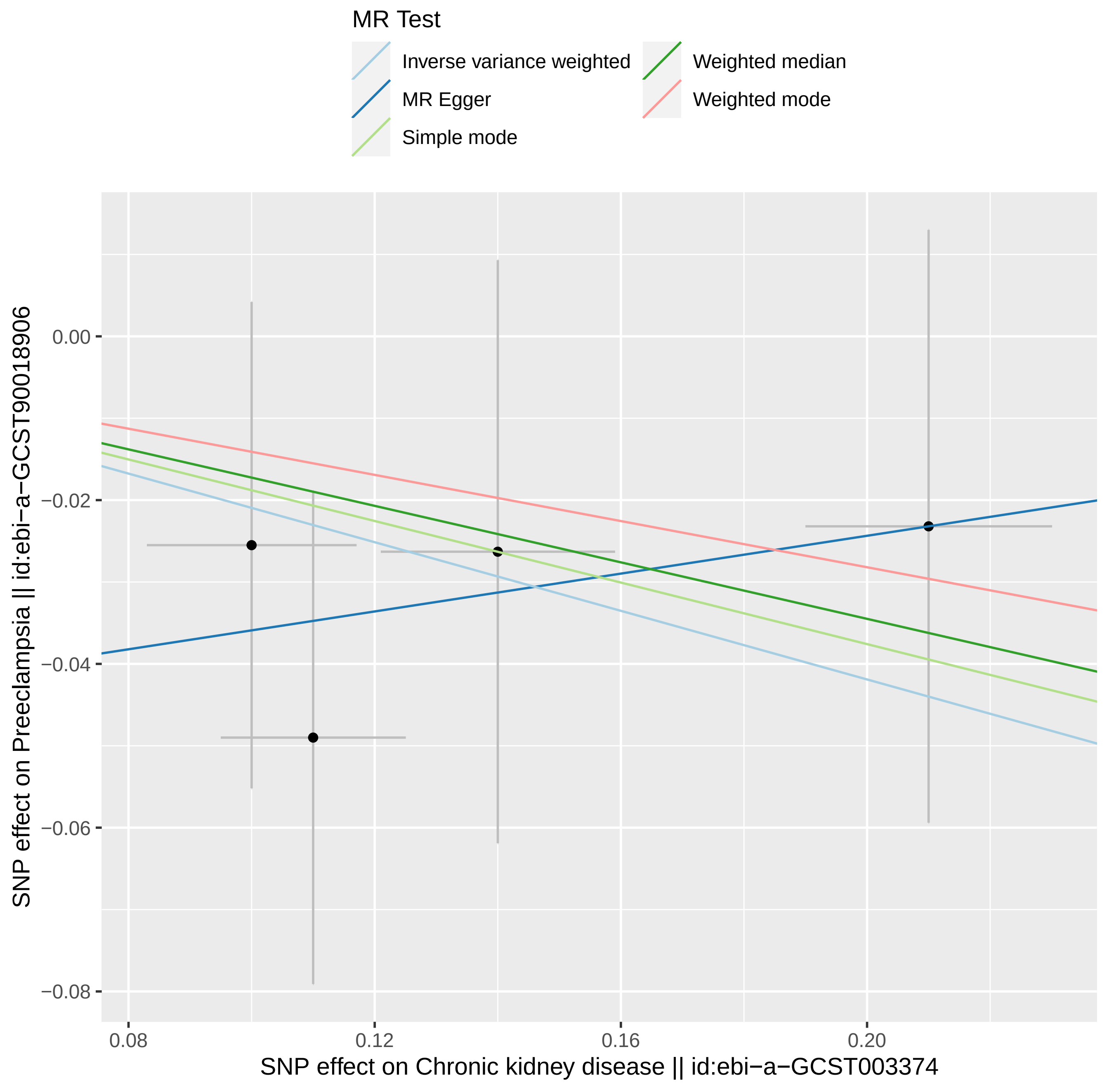

MR Test
Inverse variance weighted
MR Egger
Simple mode
Weighted median
Weighted mode
0.00
−0.02
−0.04
−0.06
−0.08
SNP effect on Preeclampsia || id:ebi−a−GCST90018906
0.08
0.12
0.16
0.20
SNP effect on Chronic kidney disease || id:ebi−a−GCST003374

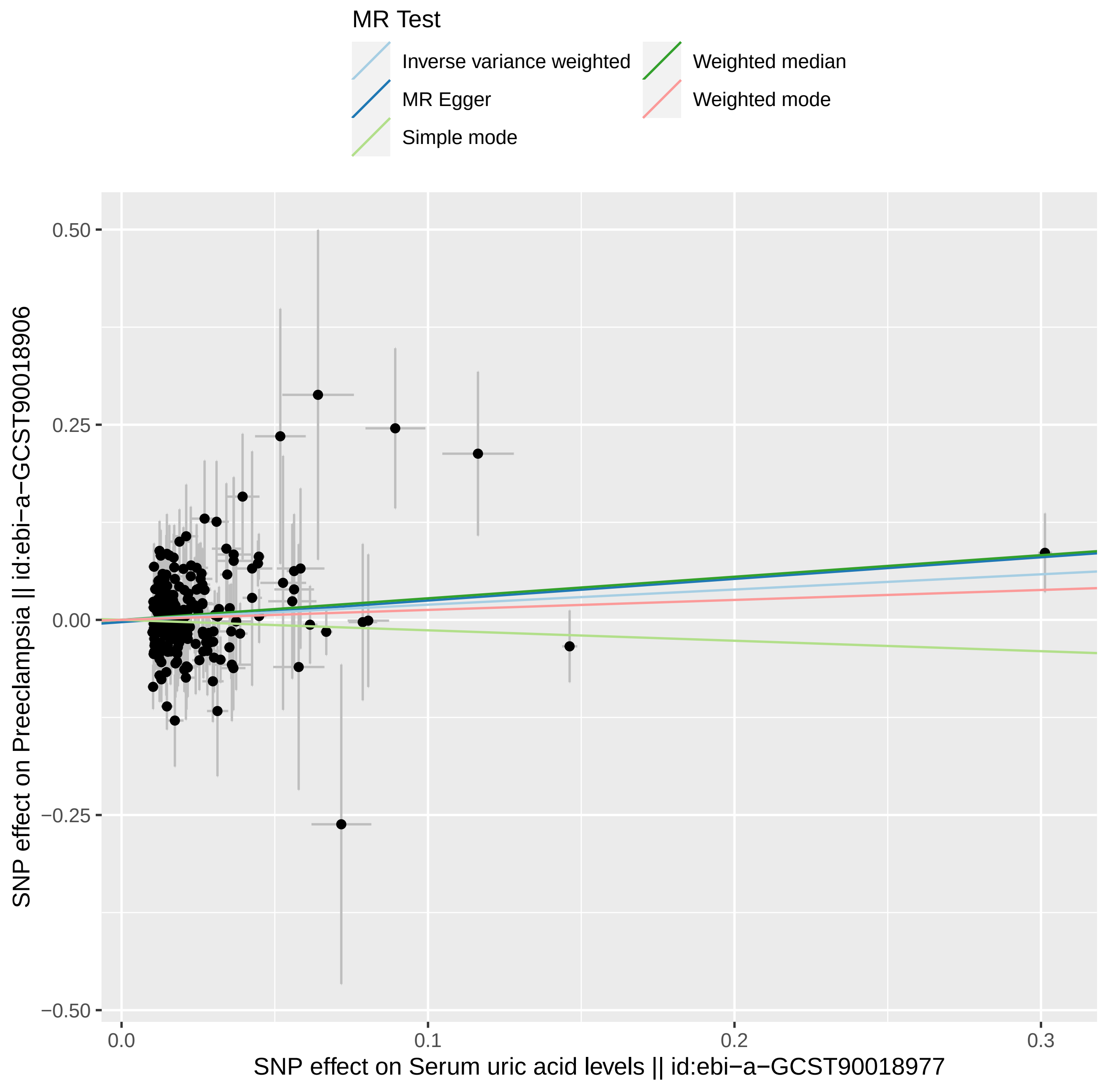
MR Test
Inverse variance weighted
MR Egger
Simple mode
Weighted median
Weighted mode
0.50
0.25
0.00
−0.25
−0.50
SNP effect on Preeclampsia || id:ebi−a−GCST90018906
0.0
0.1
0.2
0.3
SNP effect on Serum uric acid levels || id:ebi−a−GCST90018977

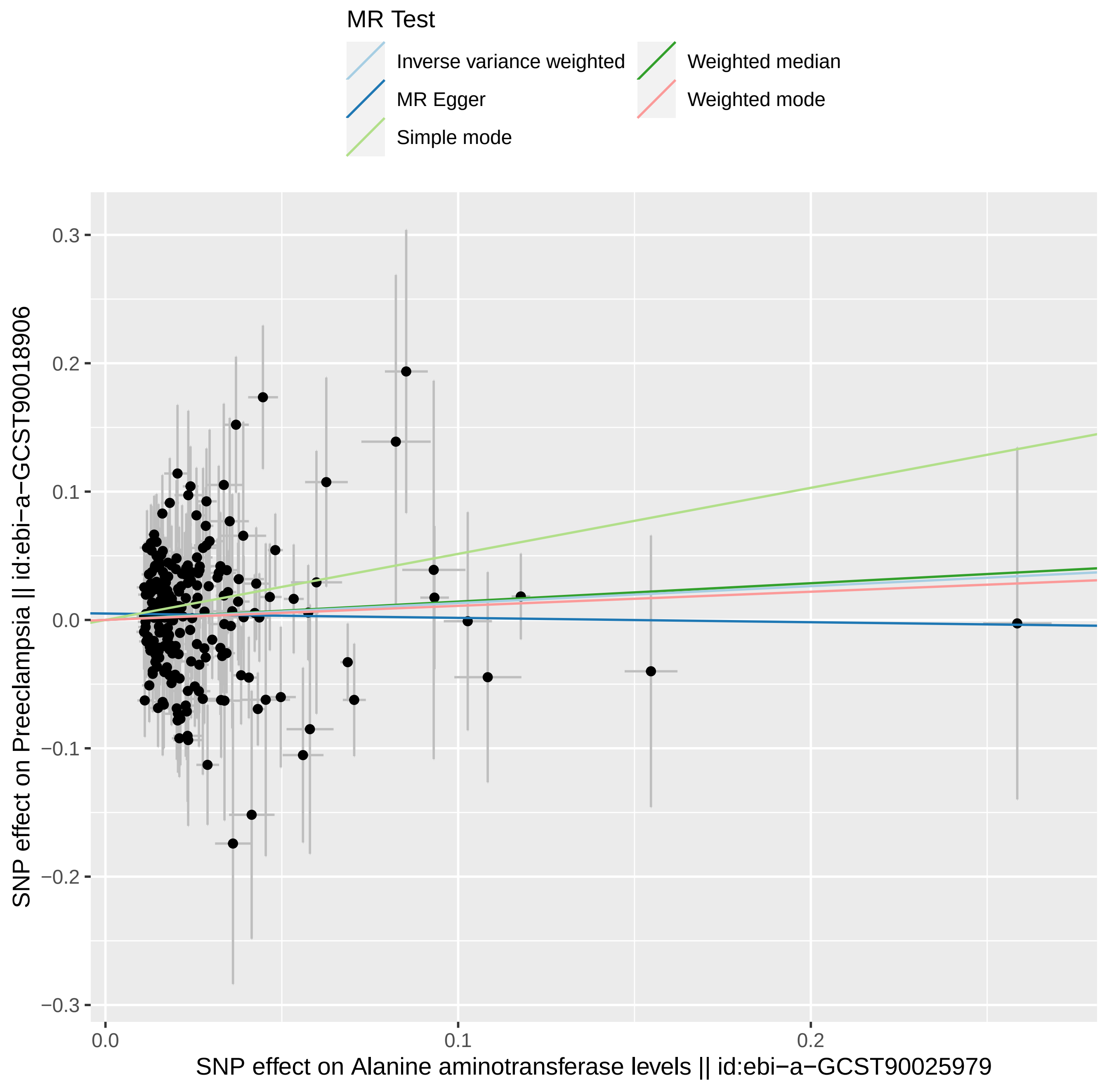
MR Test
Inverse variance weighted
MR Egger
Simple mode
Weighted median
Weighted mode
SNP effect on Preeclampsia || id:ebi−a−GCST90018906
SNP effect on Alanine aminotransferase levels || id:ebi−a−GCST90025979
0.3
0.2
0.1
0.0
−0.1
−0.2
−0.3
0.0
0.1
0.2

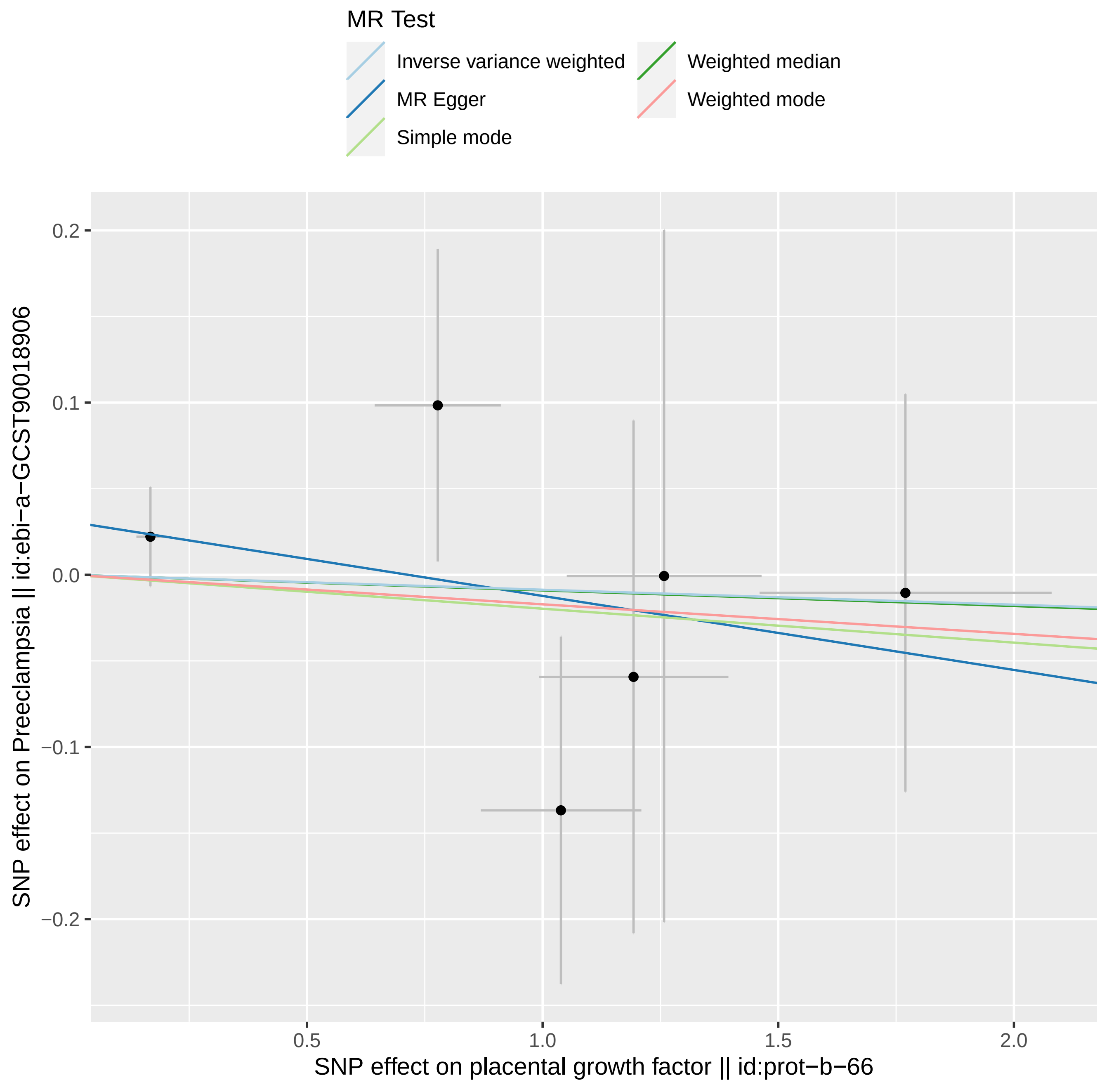
MR Test
Inverse variance weighted
MR Egger
Simple mode
Weighted median
Weighted mode
0.2
0.1
0.0
−0.1
−0.2
0.5
1.0
1.5
2.0
SNP effect on Preeclampsia || id:ebi−a−GCST90018906
SNP effect on placental growth factor || id:prot−b−66

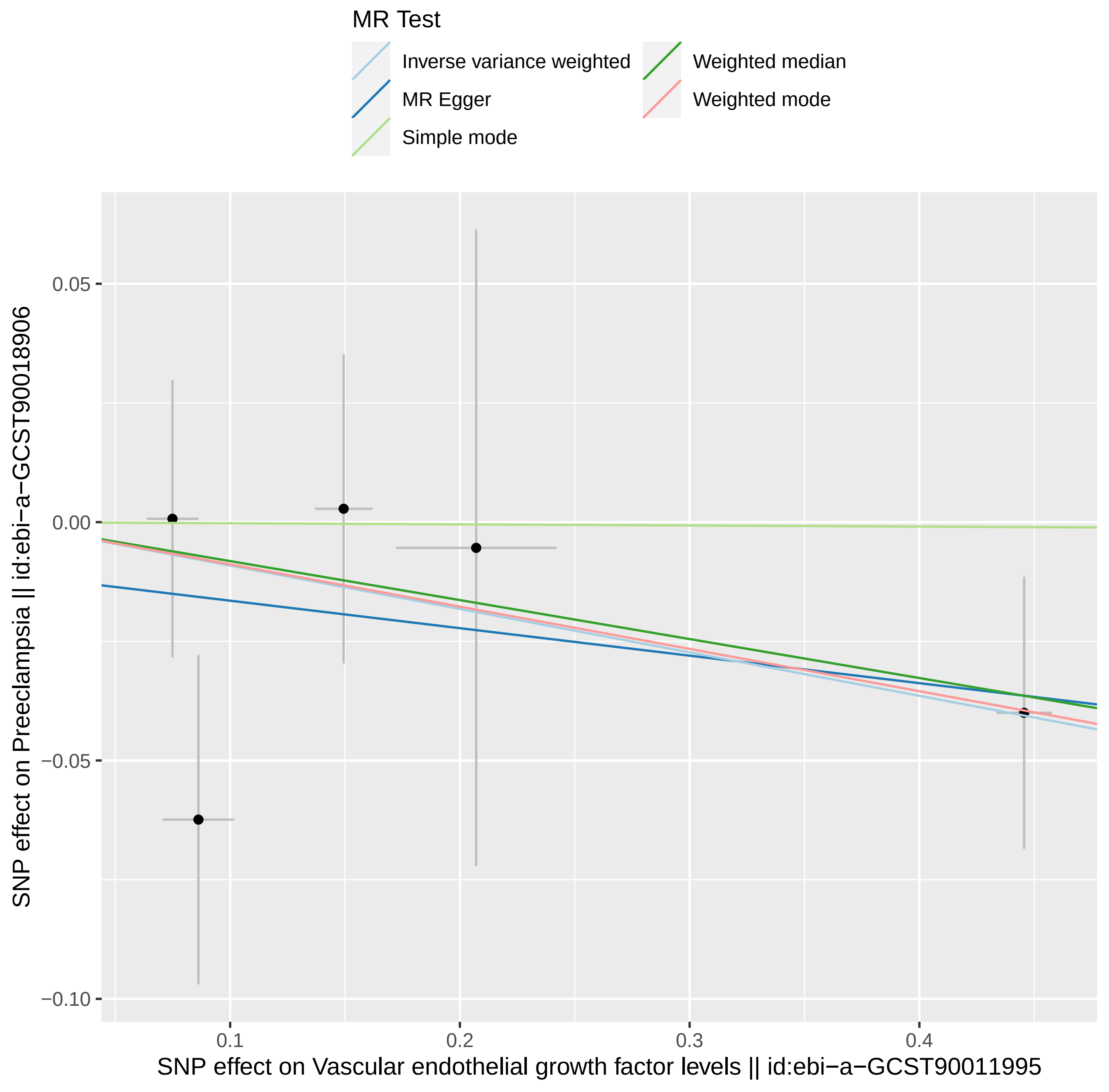
MR Test
Inverse variance weighted
MR Egger
Simple mode
Weighted median
Weighted mode
0.05
0.00
−0.05
−0.10
0.1
0.2
0.3
0.4
SNP effect on Preeclampsia || id:ebi−a−GCST90018906
SNP effect on Vascular endothelial growth factor levels || id:ebi−a−GCST90011995

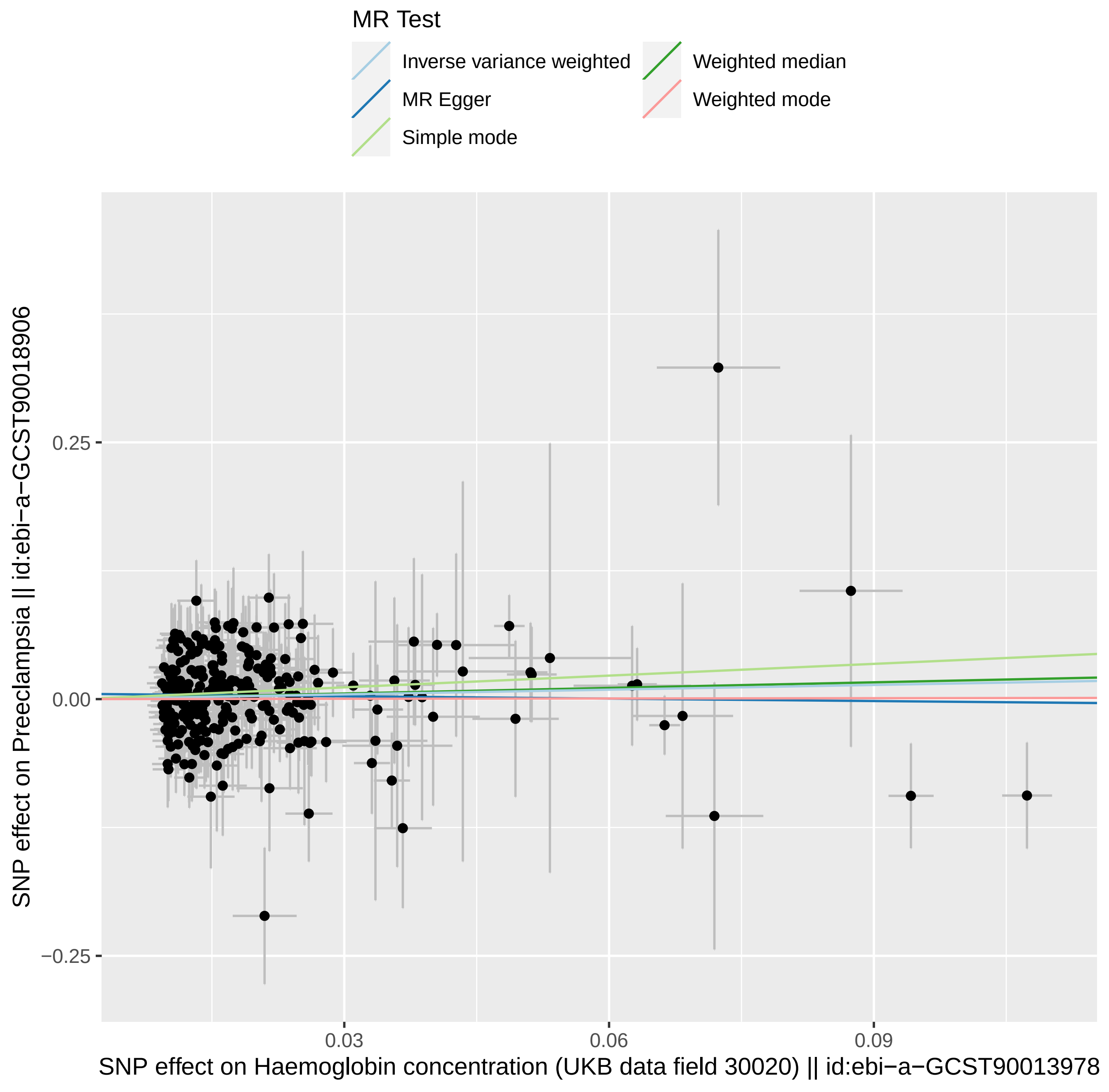
MR Test
Inverse variance weighted
MR Egger
Simple mode
Weighted median
Weighted mode
0.25
0.00
−0.25
0.03
0.06
0.09
SNP effect on Preeclampsia || id:ebi−a−GCST90018906
SNP effect on Haemoglobin concentration (UKB data field 30020) || id:ebi−a−GCST90013978

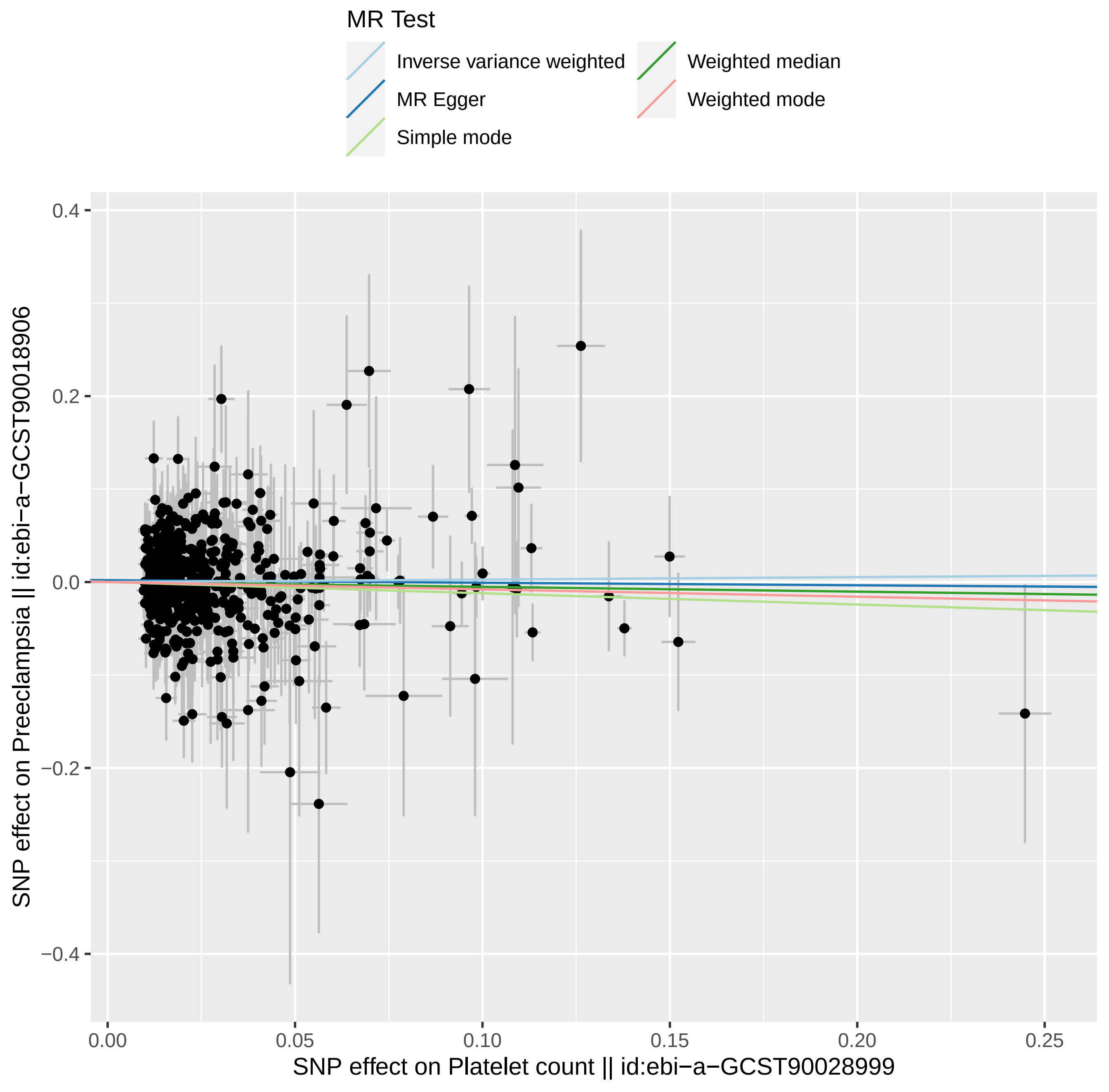

MR Test
Inverse variance weighted
MR Egger
Simple mode
Weighted median
Weighted mode
0.4
0.2
0.0
−0.2
−0.4
0.00
0.05
0.10
0.15
0.20
0.25
SNP effect on Preeclampsia || id:ebi−a−GCST90018906
SNP effect on Platelet count || id:ebi−a−GCST90028999
