# Supplementary material for: Causal pathways in preeclampsia: a Mendelian randomization study in European populations
Source: Front Endocrinol (Lausanne). 2024 Sep 2;15:1453277. doi: 10.3389/fendo.2024.1453277 (PMC11402816; doi:10.3389/fendo.2024.1453277)
Supplement: Supplementary file 3 [file DataSheet3.pdf]

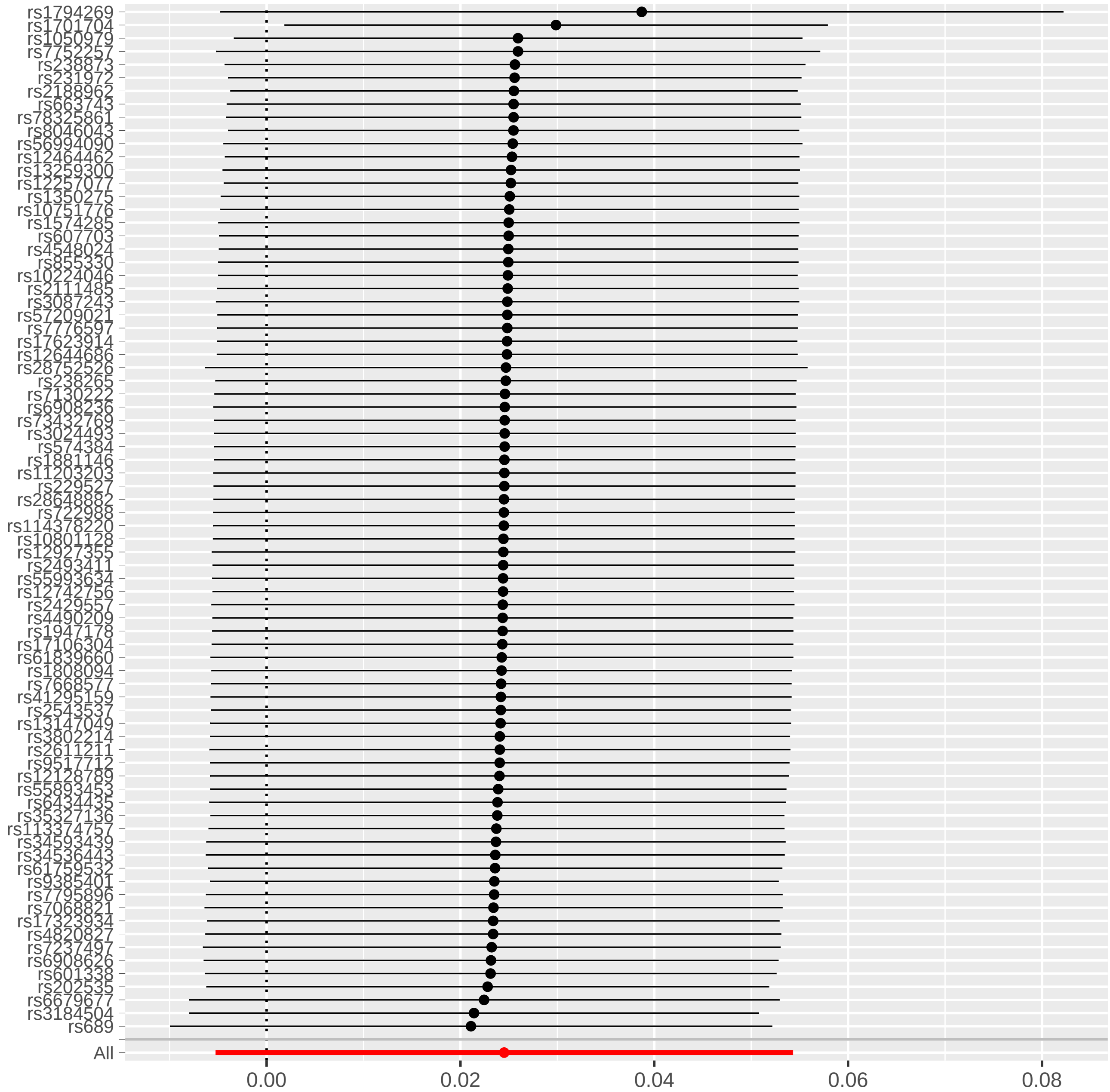

MR leave−one−out sensitivity analysis for
'Type 1 diabetes || id:ebi−a−GCST90014023' on 'Preeclampsia || id:ebi−a−GCST90018906'

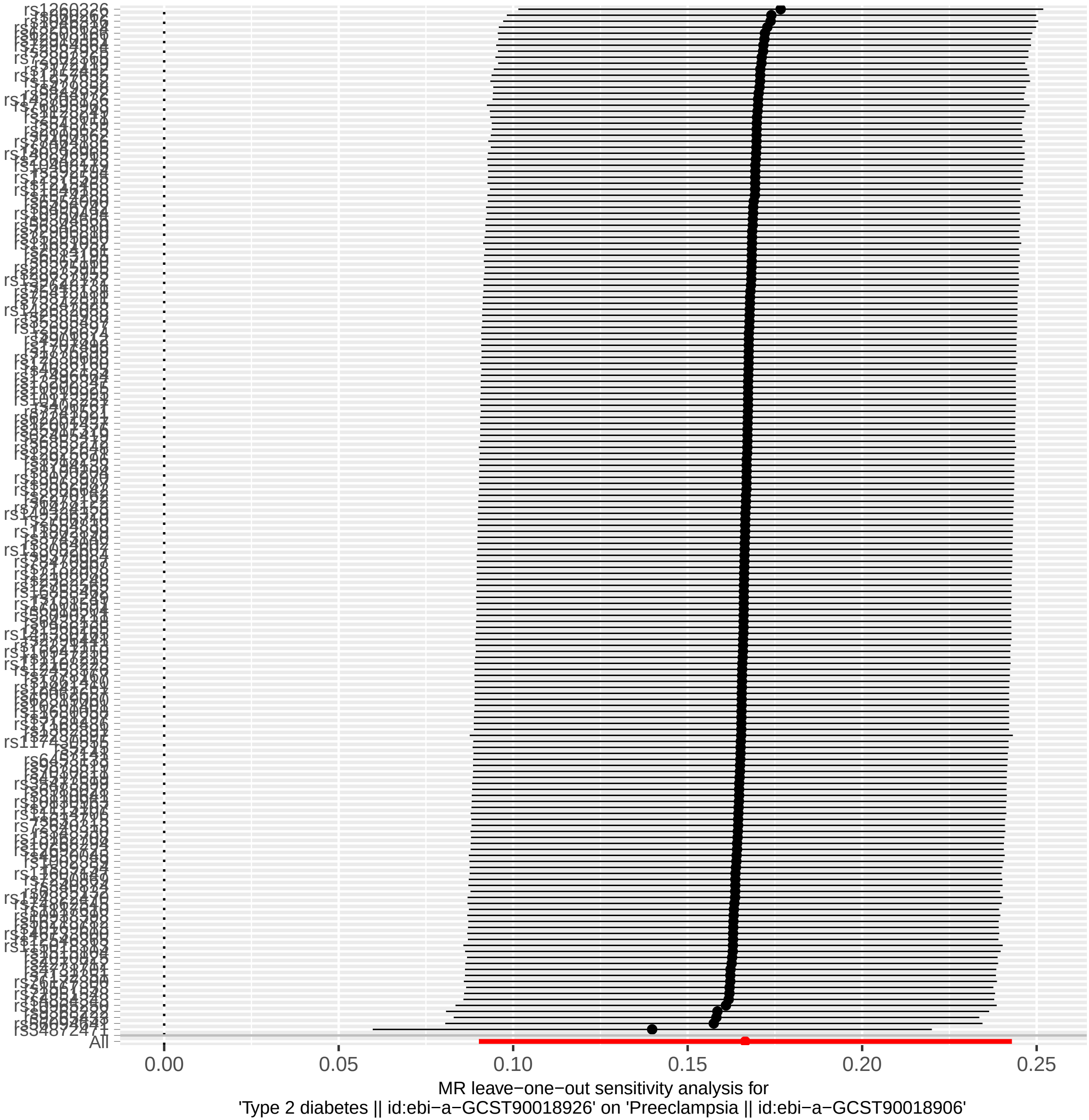
rs1260326
rs34872471
All
0.00
0.05
0.10
0.15
0.20
0.25
MR leave−one−out sensitivity analysis for
'Type 2 diabetes || id:ebi−a−GCST90018926' on 'Preeclampsia || id:ebi−a−GCST90018906'

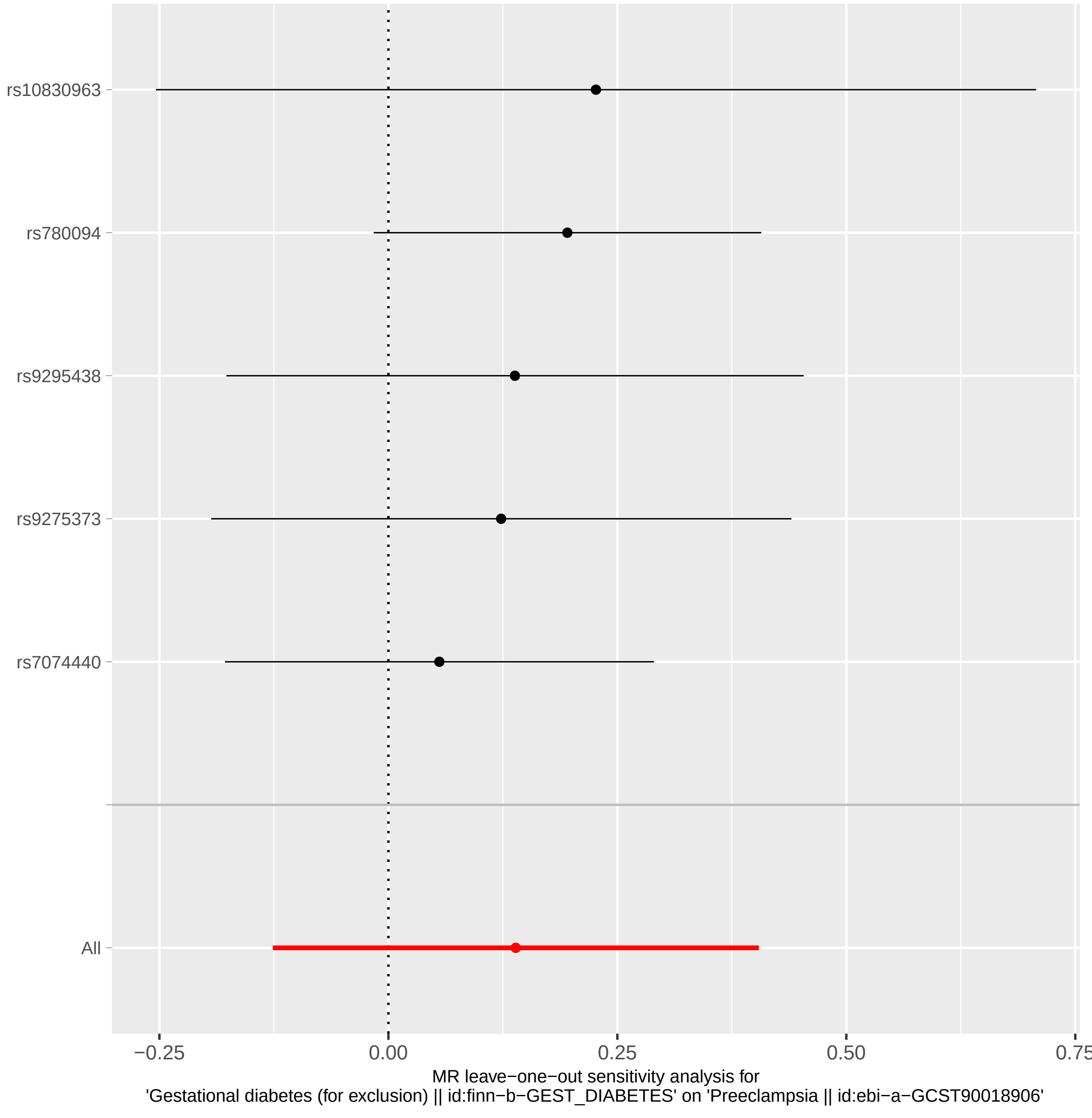
rs10830963
rs780094
rs9295438
rs9275373
rs7074440
All
−0.25
0.00
0.25
0.50
0.75
MR leave−one−out sensitivity analysis for
'Gestational diabetes (for exclusion) || id:finn−b−GEST_DIABETES' on 'Preeclampsia || id:ebi−a−GCST90018906'

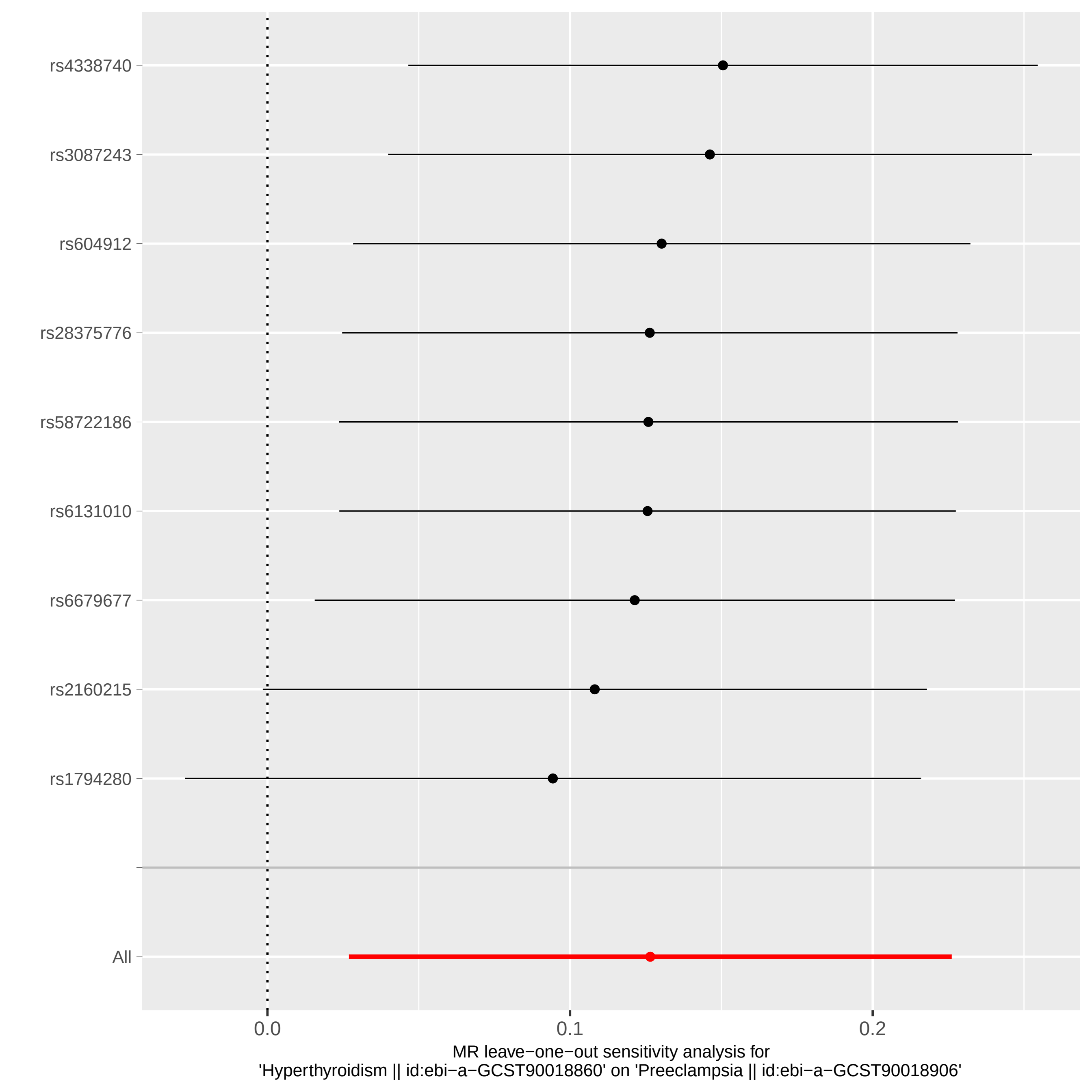

rs4338740
rs3087243
rs604912
rs28375776
rs58722186
rs6131010
rs6679677
rs2160215
rs1794280
All
0.0
0.1
0.2
MR leave−one−out sensitivity analysis for
'Hyperthyroidism || id:ebi−a−GCST90018860' on 'Preeclampsia || id:ebi−a−GCST90018906'

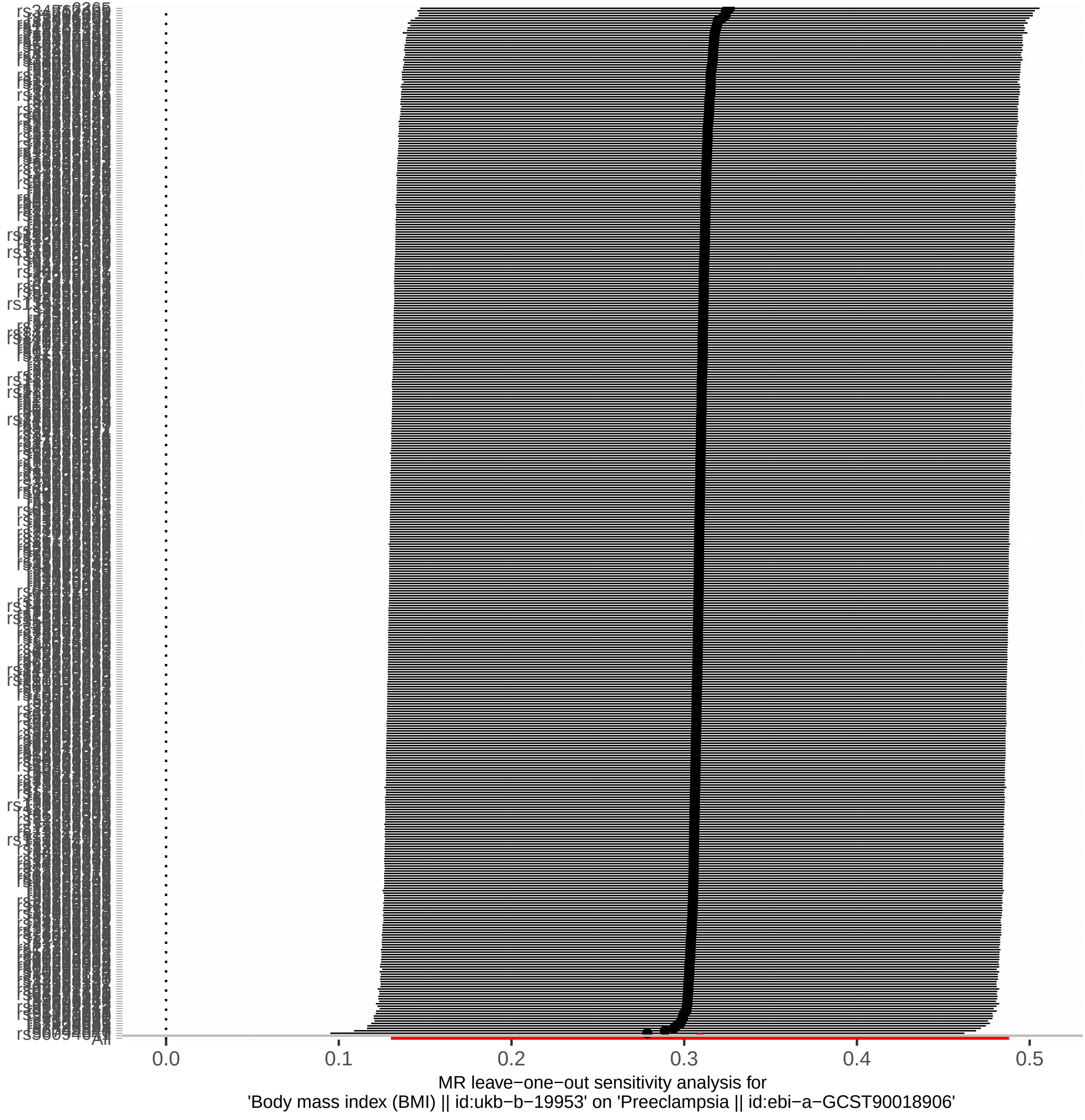
All
0.0
0.1
0.2
0.3
0.4
0.5
MR leave−one−out sensitivity analysis for
'Body mass index (BMI) || id:ukb−b−19953' on 'Preeclampsia || id:ebi−a−GCST90018906'

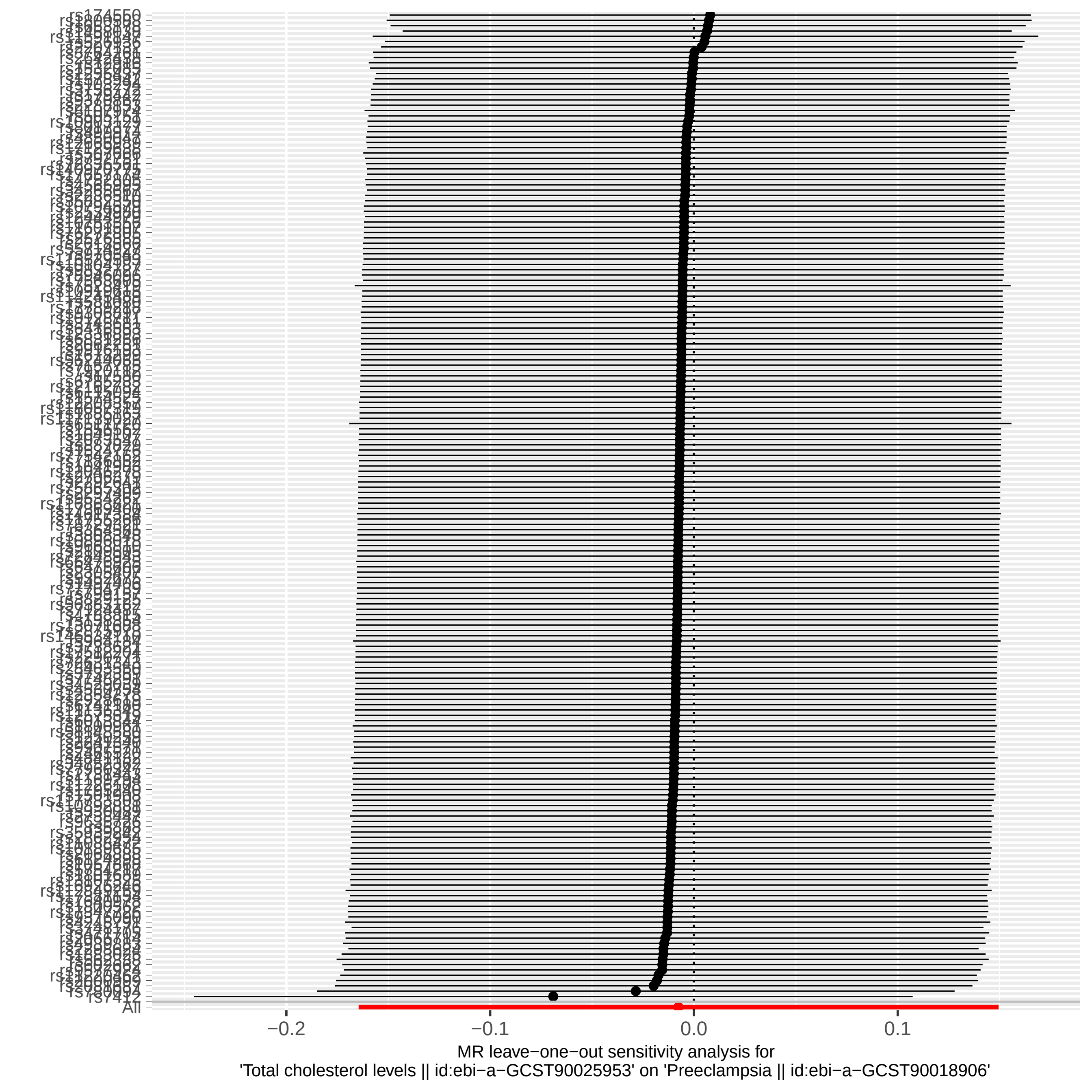
rs174550
rs7412
All
−0.2
−0.1
0.0
0.1
MR leave−one−out sensitivity analysis for
'Total cholesterol levels || id:ebi−a−GCST90025953' on 'Preeclampsia || id:ebi−a−GCST90018906'

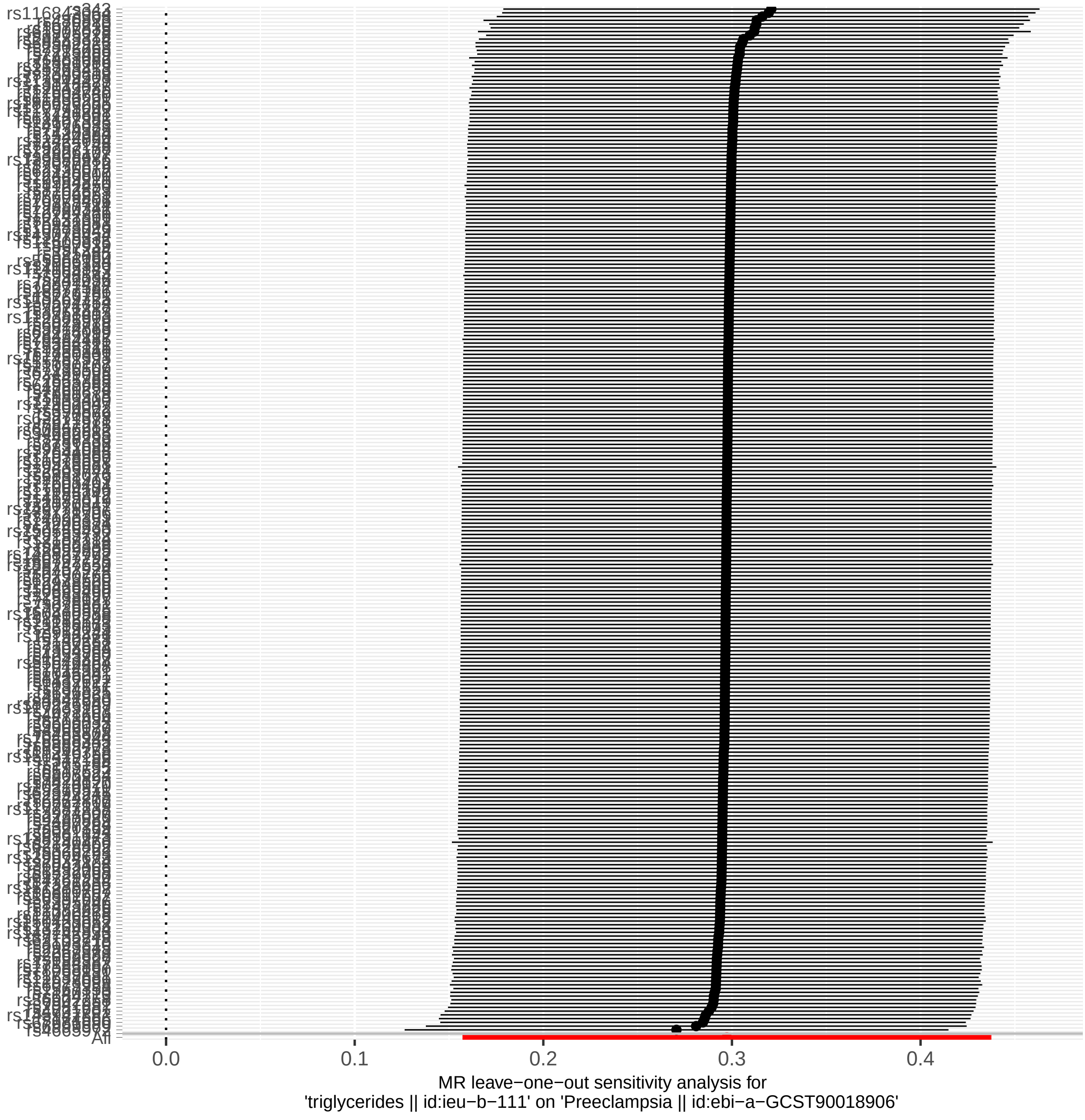
All
0.0
0.1
0.2
0.3
0.4
MR leave−one−out sensitivity analysis for
'triglycerides || id:ieu−b−111' on 'Preeclampsia || id:ebi−a−GCST90018906'

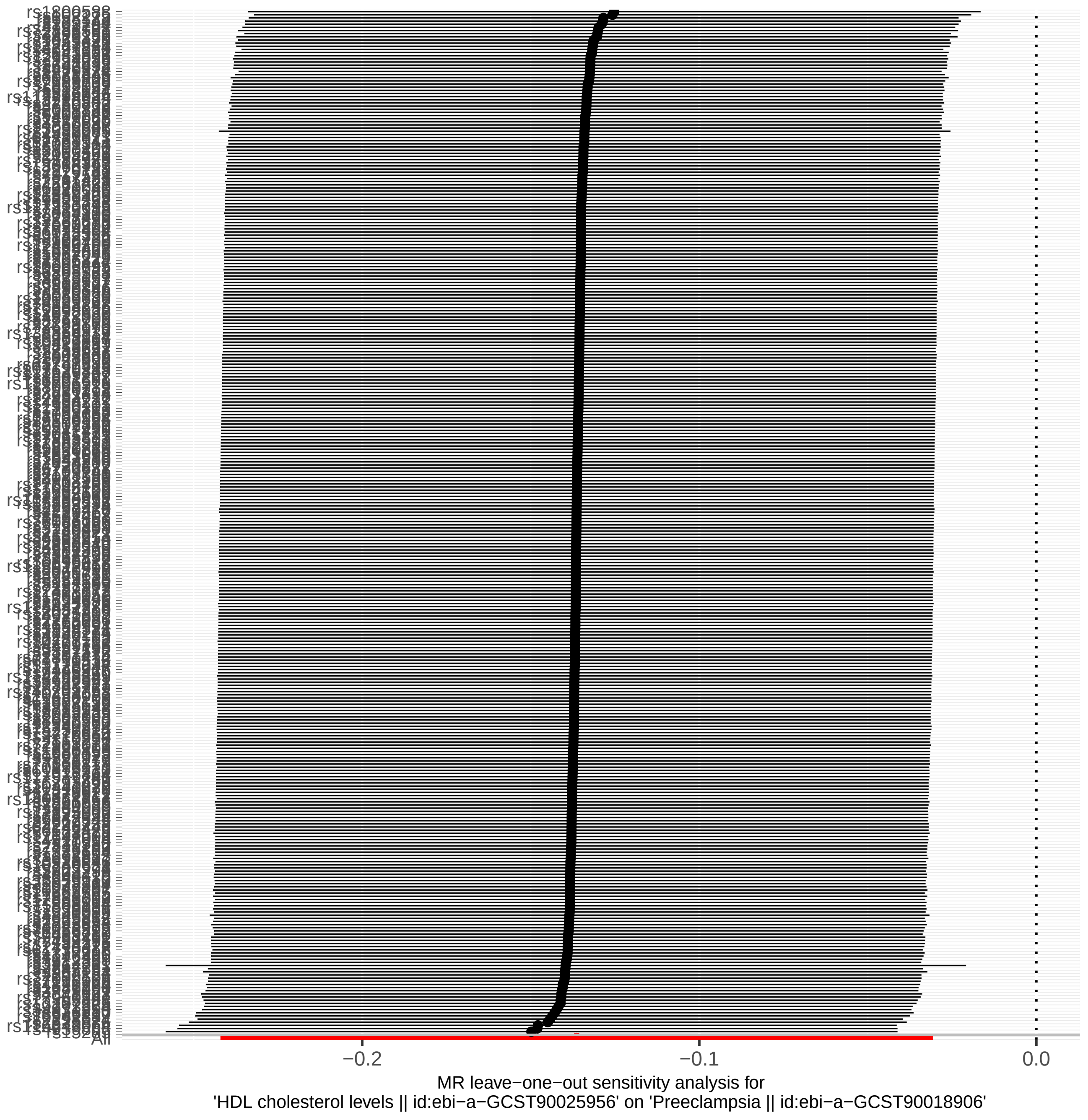
All
−0.2
−0.1
0.0
MR leave−one−out sensitivity analysis for
'HDL cholesterol levels || id:ebi−a−GCST90025956' on 'Preeclampsia || id:ebi−a−GCST90018906'

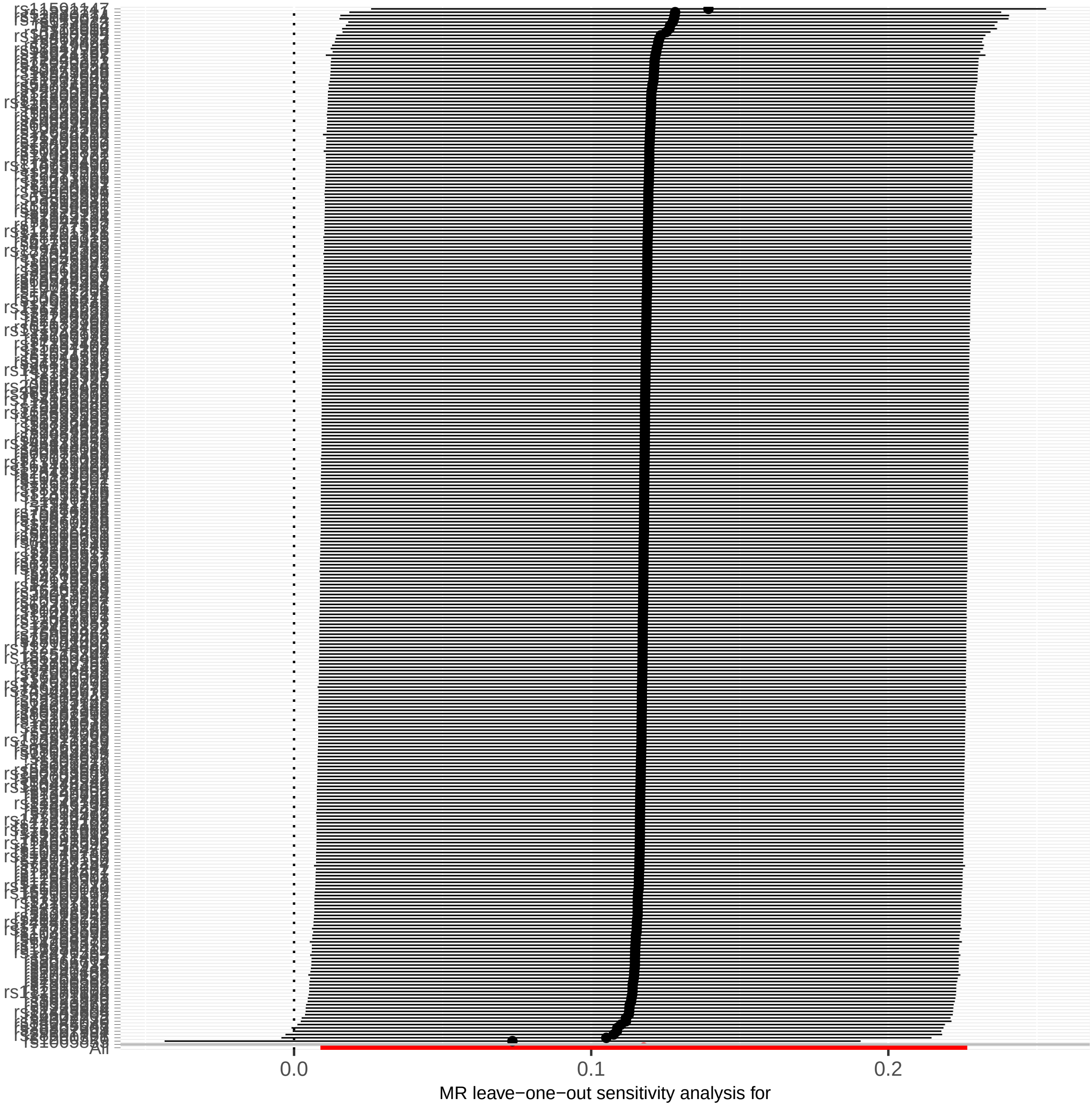

MR leave−one−out sensitivity analysis for
'Low density lipoprotein cholesterol levels || id:ebi−a−GCST90002412' on 'Preeclampsia || id:ebi−a−GCST90018906'

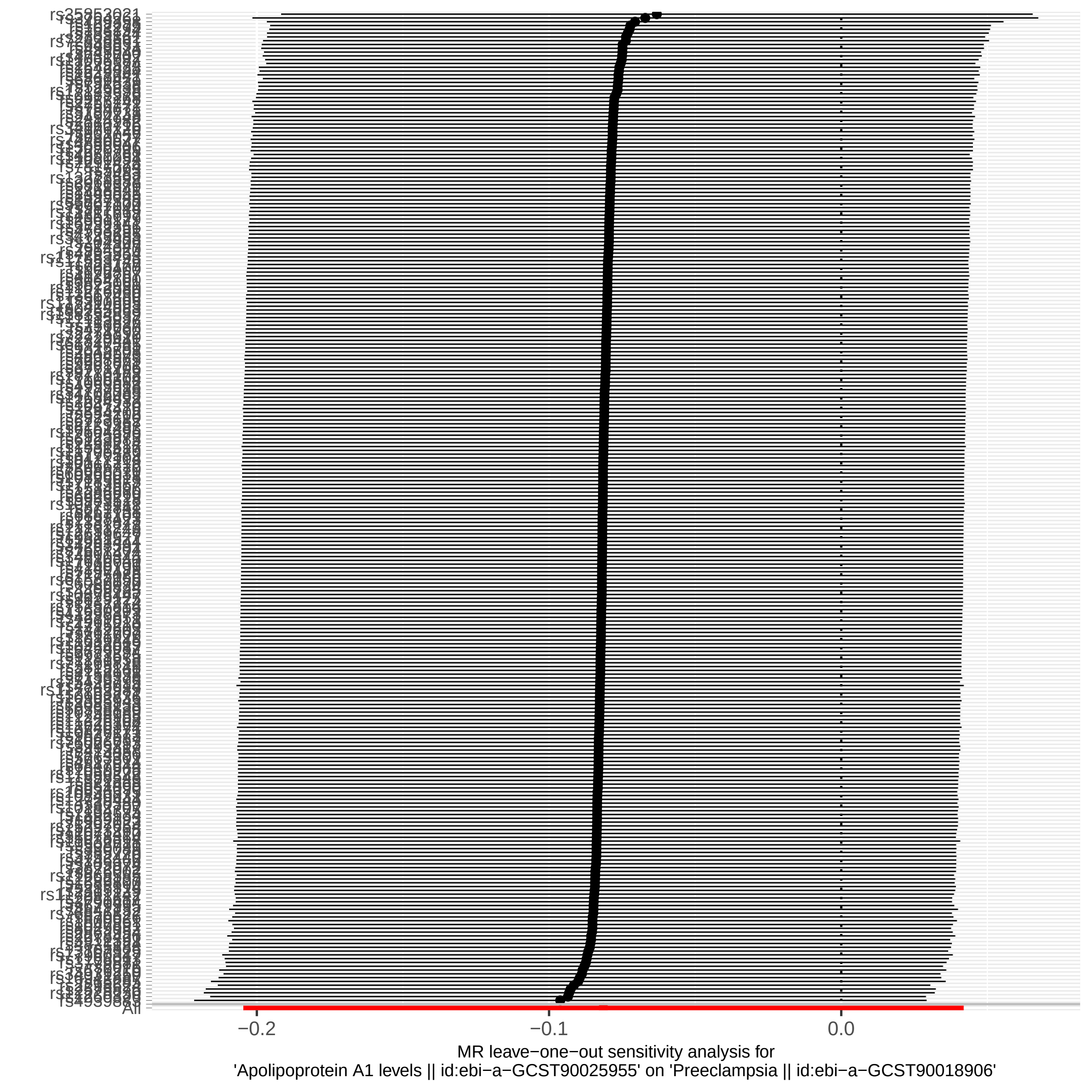
All
−0.2
−0.1
0.0
MR leave−one−out sensitivity analysis for
'Apolipoprotein A1 levels || id:ebi−a−GCST90025955' on 'Preeclampsia || id:ebi−a−GCST90018906'

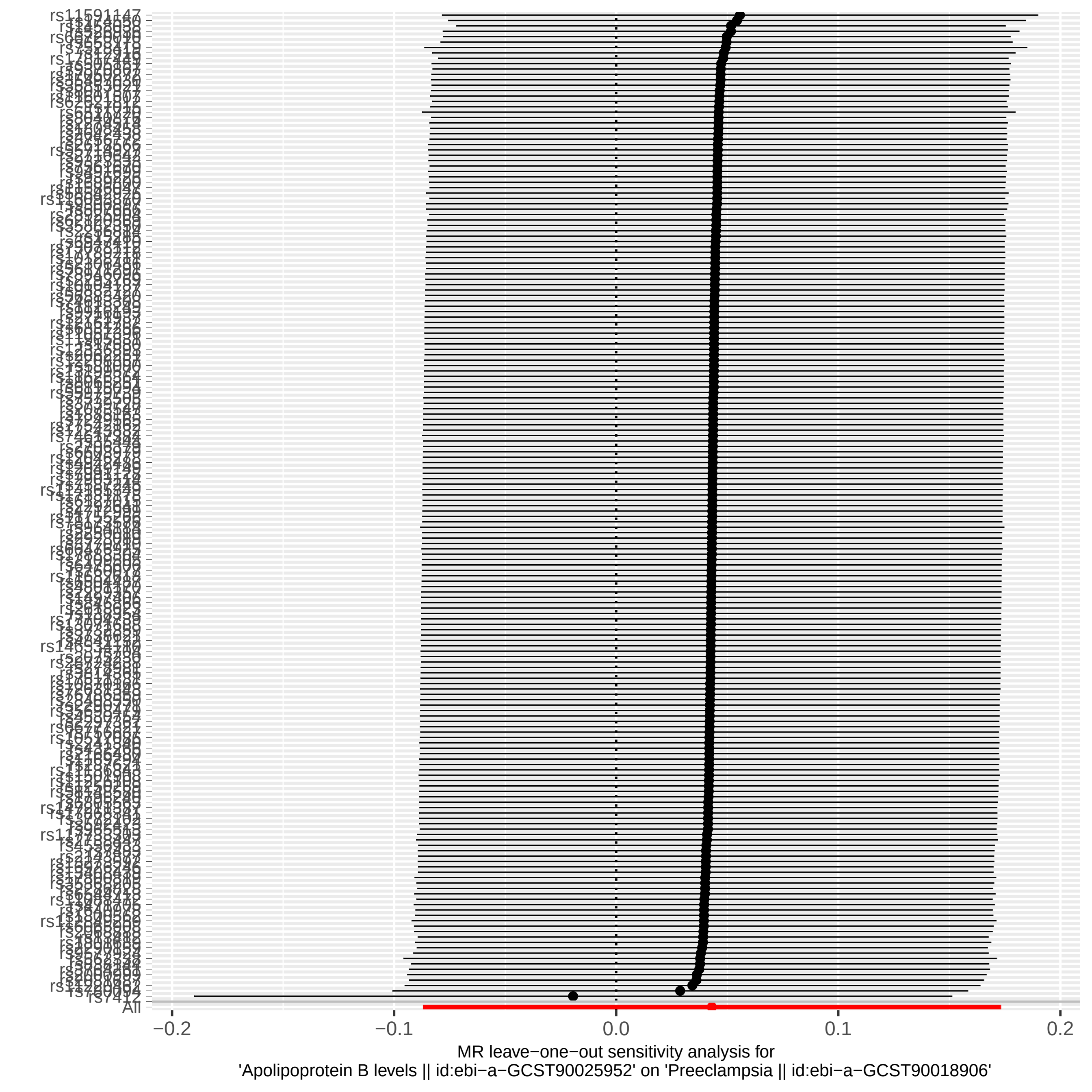
rs11591147
rs7412
All
−0.2
−0.1
0.0
0.1
0.2
MR leave−one−out sensitivity analysis for
'Apolipoprotein B levels || id:ebi−a−GCST90025952' on 'Preeclampsia || id:ebi−a−GCST90018906'

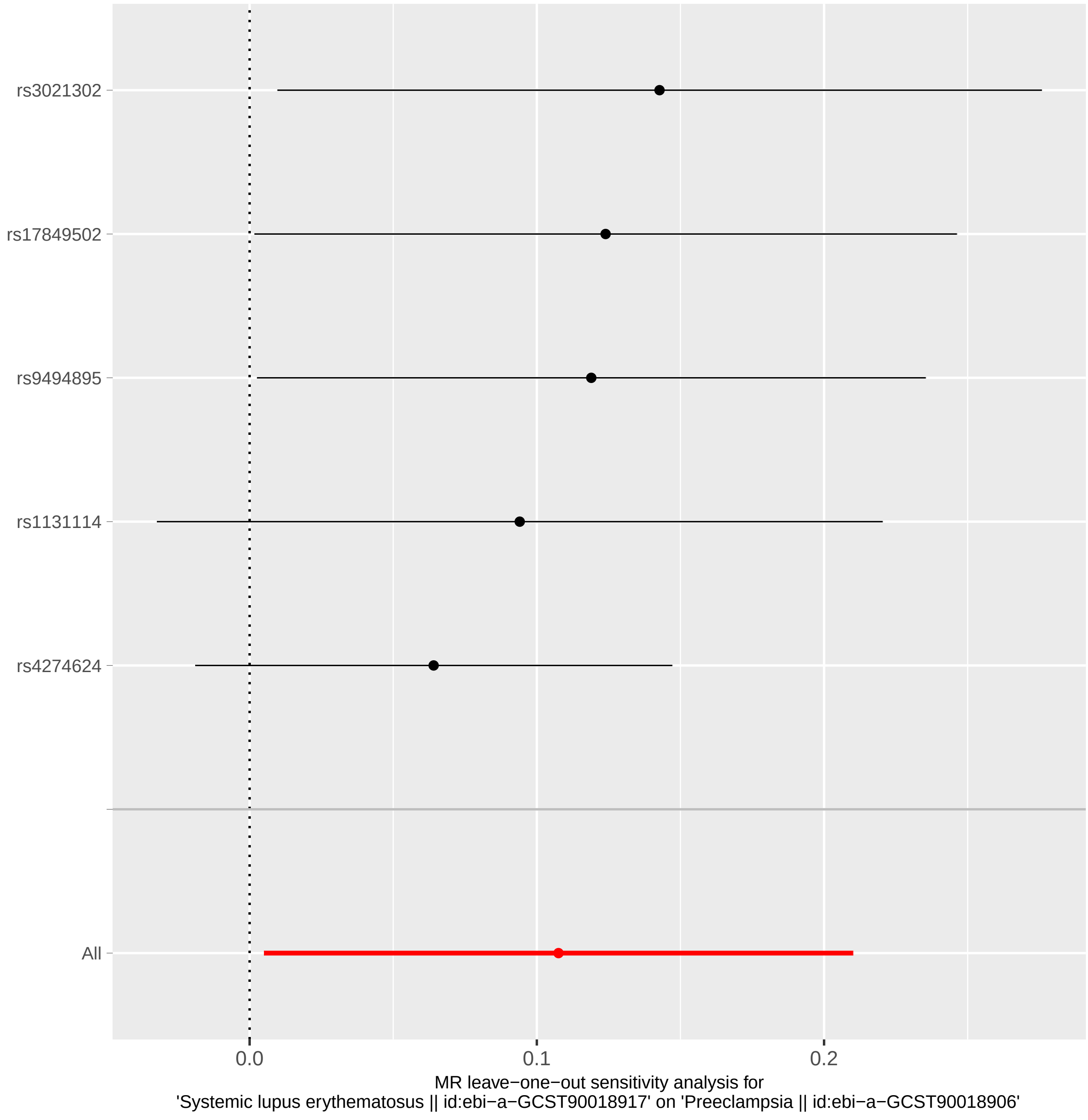
rs3021302
rs17849502
rs9494895
rs1131114
rs4274624
All
0.0
0.1
0.2
MR leave−one−out sensitivity analysis for
'Systemic lupus erythematosus || id:ebi−a−GCST90018917' on 'Preeclampsia || id:ebi−a−GCST90018906'

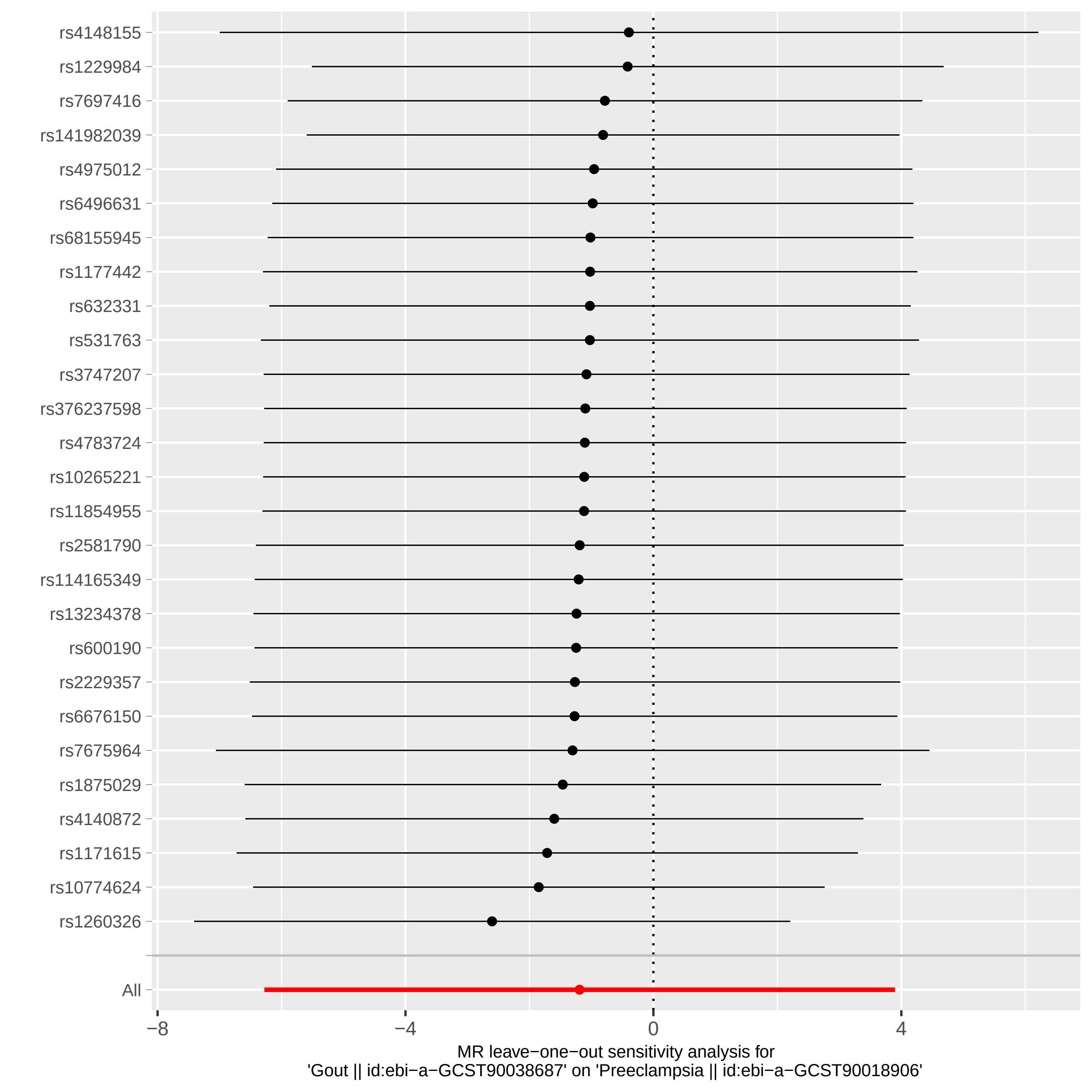
rs4148155
rs1229984
rs7697416
rs141982039
rs4975012
rs6496631
rs68155945
rs1177442
rs632331
rs531763
rs3747207
rs376237598
rs4783724
rs10265221
rs11854955
rs2581790
rs114165349
rs13234378
rs600190
rs2229357
rs6676150
rs7675964
rs1875029
rs4140872
rs1171615
rs10774624
rs1260326
All
−8
−4
0
4
MR leave−one−out sensitivity analysis for
'Gout || id:ebi−a−GCST90038687' on 'Preeclampsia || id:ebi−a−GCST90018906'

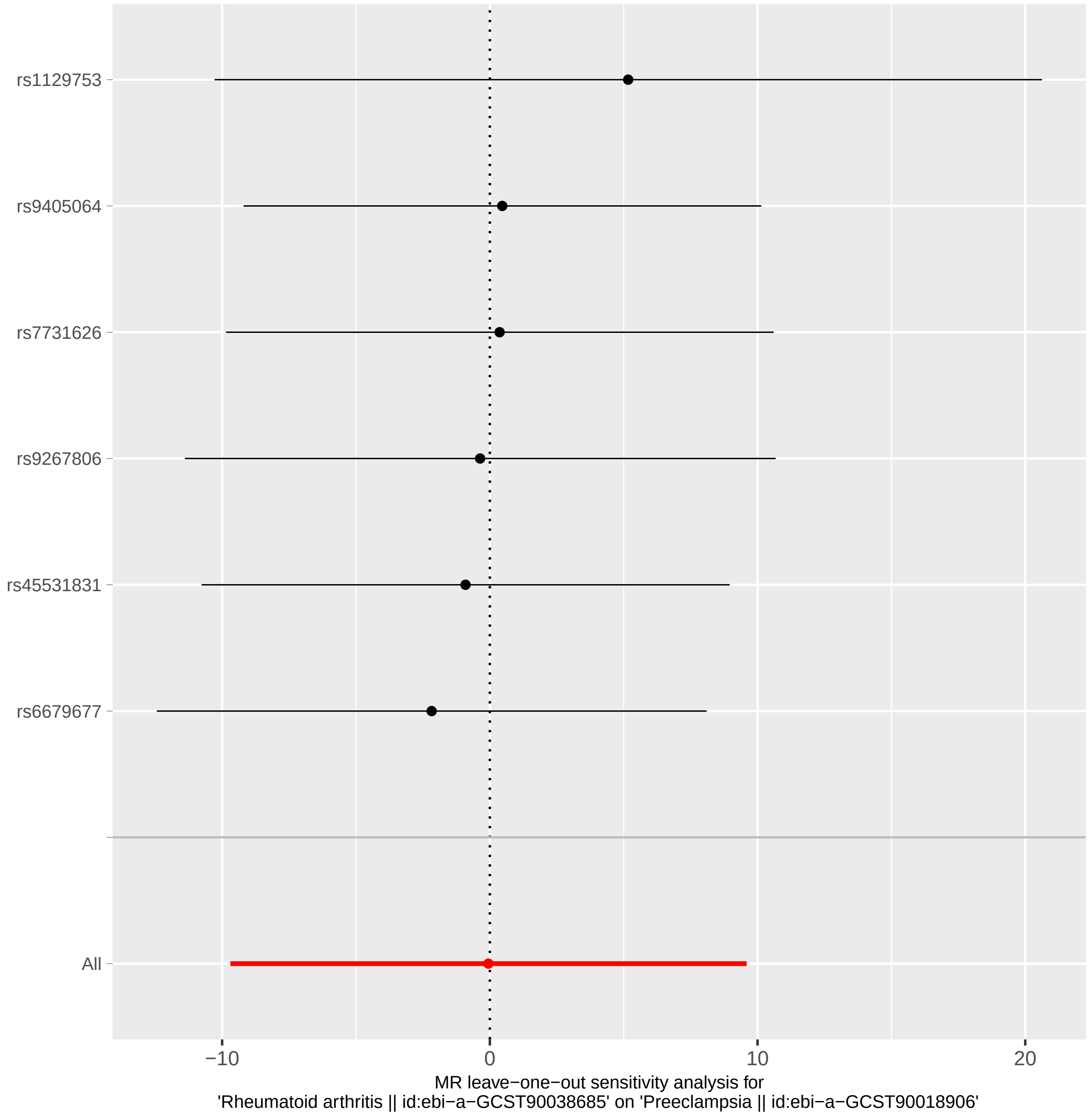
rs1129753
rs9405064
rs7731626
rs9267806
rs45531831
rs6679677
All
−10
0
10
20
MR leave−one−out sensitivity analysis for
'Rheumatoid arthritis || id:ebi−a−GCST90038685' on 'Preeclampsia || id:ebi−a−GCST90018906'

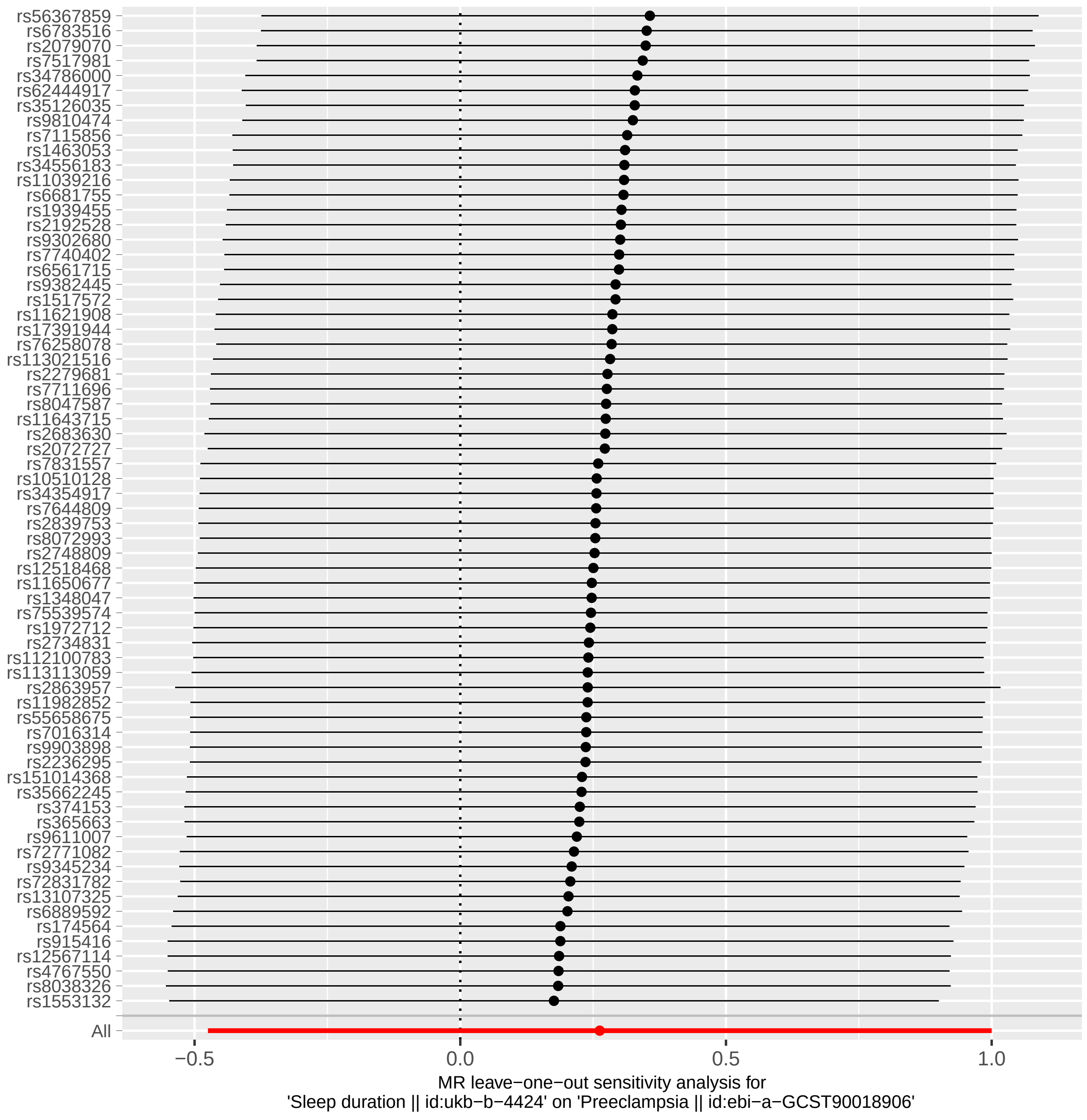
rs56367859
rs6783516
rs2079070
rs7517981
rs34786000
rs62444917
rs35126035
rs9810474
rs7115856
rs1463053
rs34556183
rs11039216
rs6681755
rs1939455
rs2192528
rs9302680
rs7740402
rs6561715
rs9382445
rs1517572
rs11621908
rs17391944
rs76258078
rs113021516
rs2279681
rs7711696
rs8047587
rs11643715
rs2683630
rs2072727
rs7831557
rs10510128
rs34354917
rs7644809
rs2839753
rs8072993
rs2748809
rs12518468
rs11650677
rs1348047
rs75539574
rs1972712
rs2734831
rs112100783
rs113113059
rs2863957
rs11982852
rs55658675
rs7016314
rs9903898
rs2236295
rs151014368
rs35662245
rs374153
rs365663
rs9611007
rs72771082
rs9345234
rs72831782
rs13107325
rs6889592
rs174564
rs915416
rs12567114
rs4767550
rs8038326
rs1553132
All
−0.5
0.0
0.5
1.0
MR leave−one−out sensitivity analysis for
'Sleep duration || id:ukb−b−4424' on 'Preeclampsia || id:ebi−a−GCST90018906'

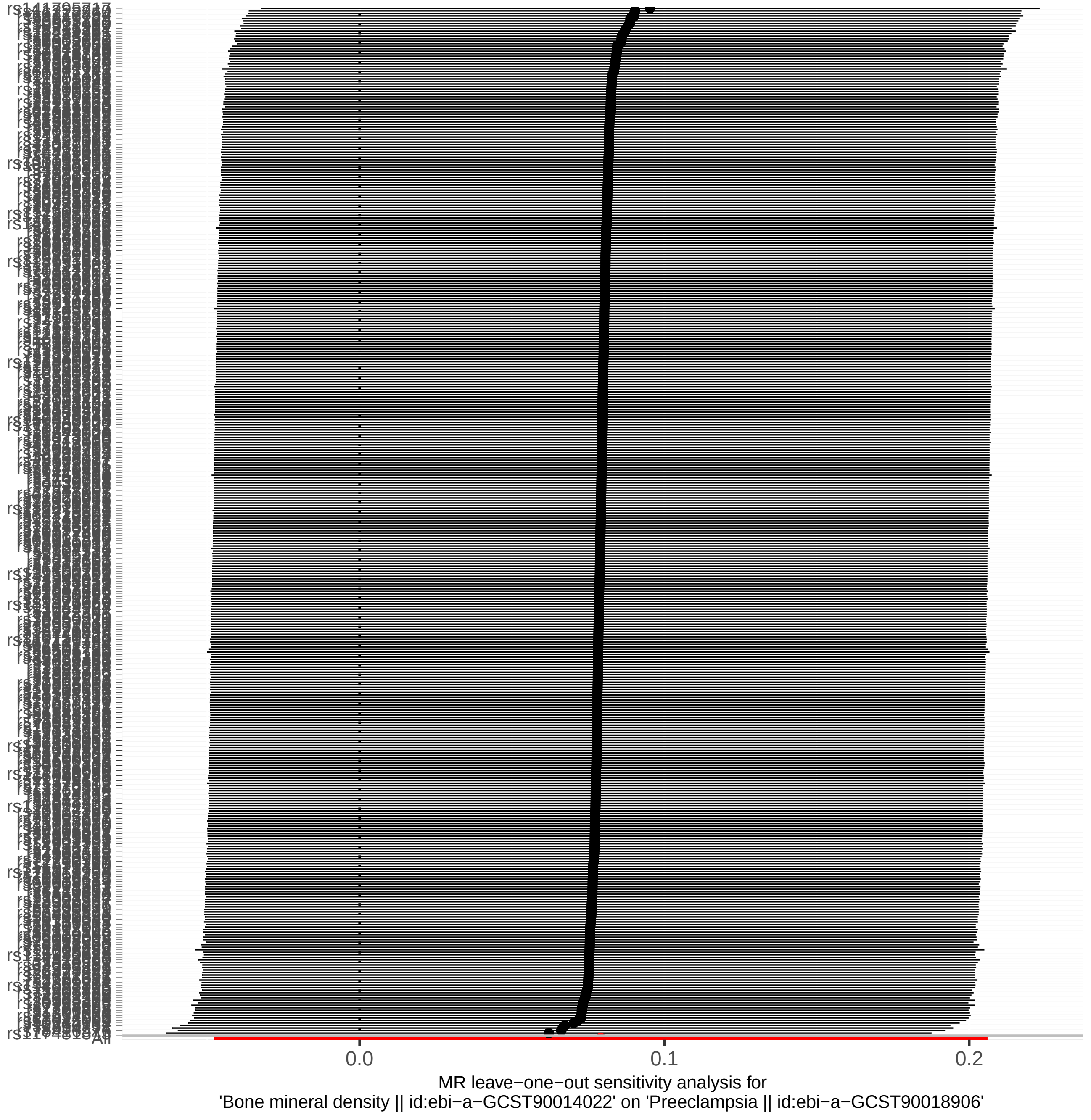
All
0.0
0.1
0.2
MR leave−one−out sensitivity analysis for
'Bone mineral density || id:ebi−a−GCST90014022' on 'Preeclampsia || id:ebi−a−GCST90018906'

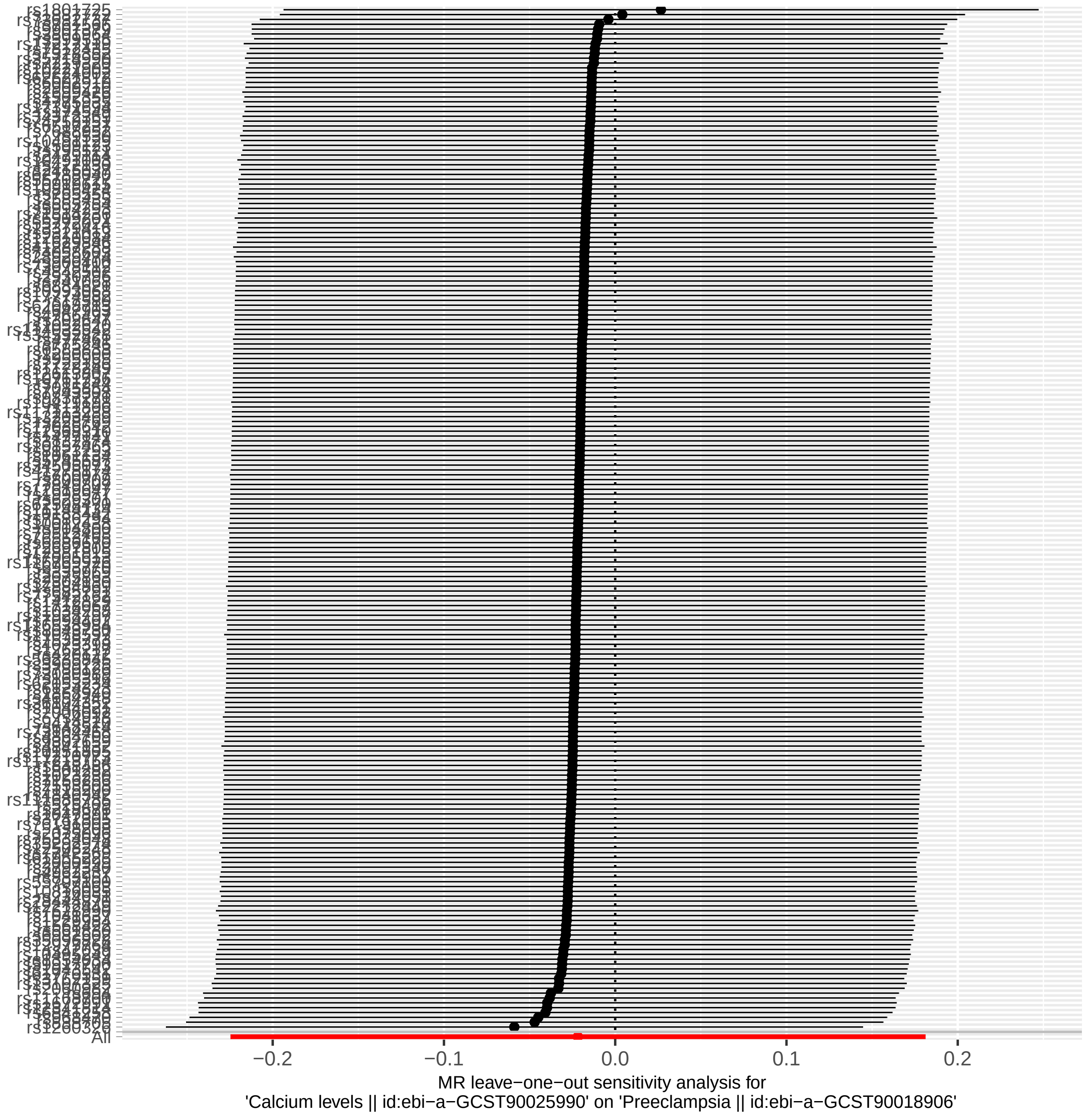
rs1801725
rs1260326
All
−0.2
−0.1
0.0
0.1
0.2
MR leave−one−out sensitivity analysis for
'Calcium levels || id:ebi−a−GCST90025990' on 'Preeclampsia || id:ebi−a−GCST90018906'

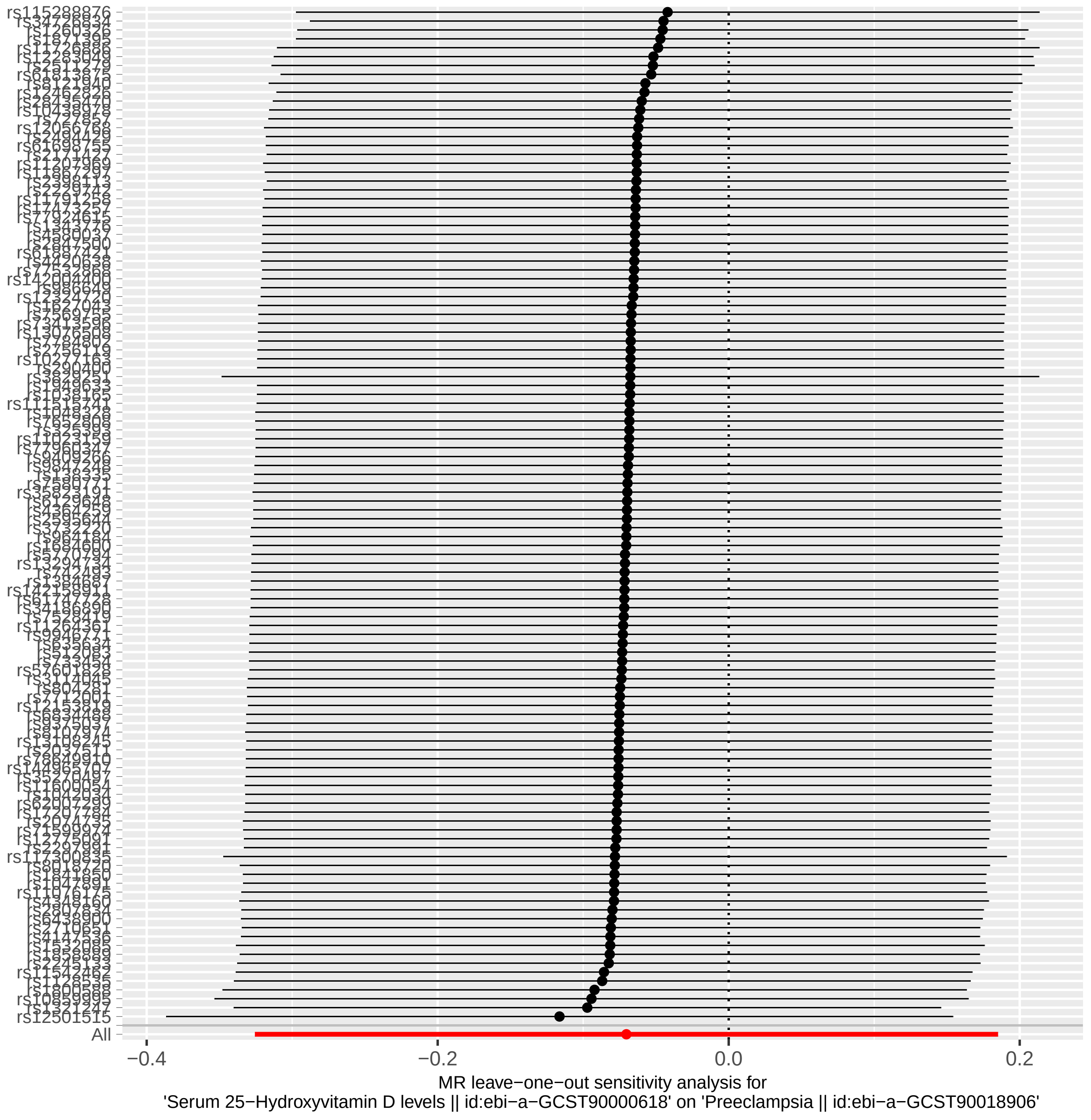
rs115288876
rs12501515
All
−0.4
−0.2
0.0
0.2
MR leave−one−out sensitivity analysis for
'Serum 25−Hydroxyvitamin D levels || id:ebi−a−GCST90000618' on 'Preeclampsia || id:ebi−a−GCST90018906'

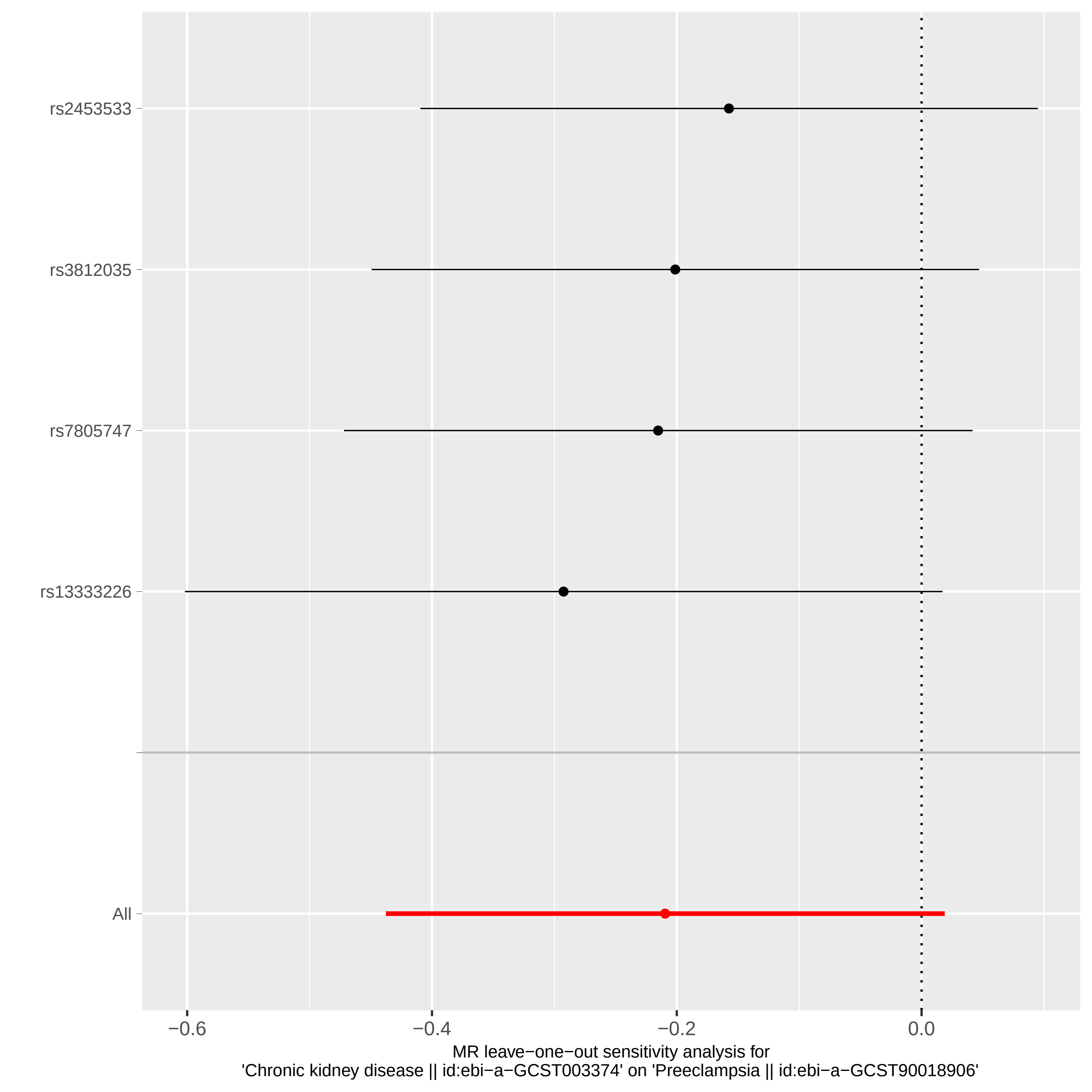
rs2453533
rs3812035
rs7805747
rs13333226
All
−0.6
−0.4
−0.2
0.0
MR leave−one−out sensitivity analysis for
'Chronic kidney disease || id:ebi−a−GCST003374' on 'Preeclampsia || id:ebi−a−GCST90018906'

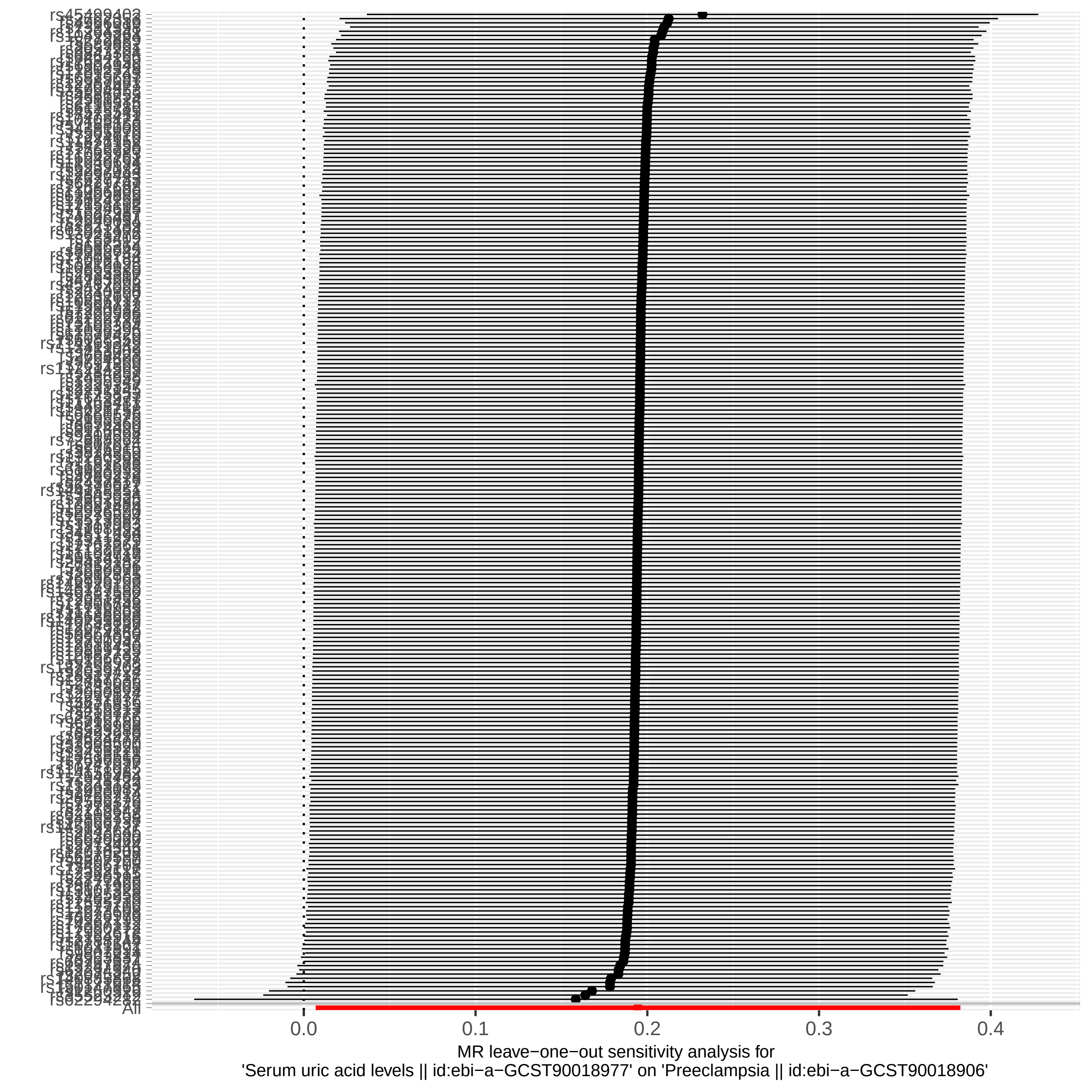
rs45409402
All
0.0
0.1
0.2
0.3
0.4
MR leave−one−out sensitivity analysis for
'Serum uric acid levels || id:ebi−a−GCST90018977' on 'Preeclampsia || id:ebi−a−GCST90018906'

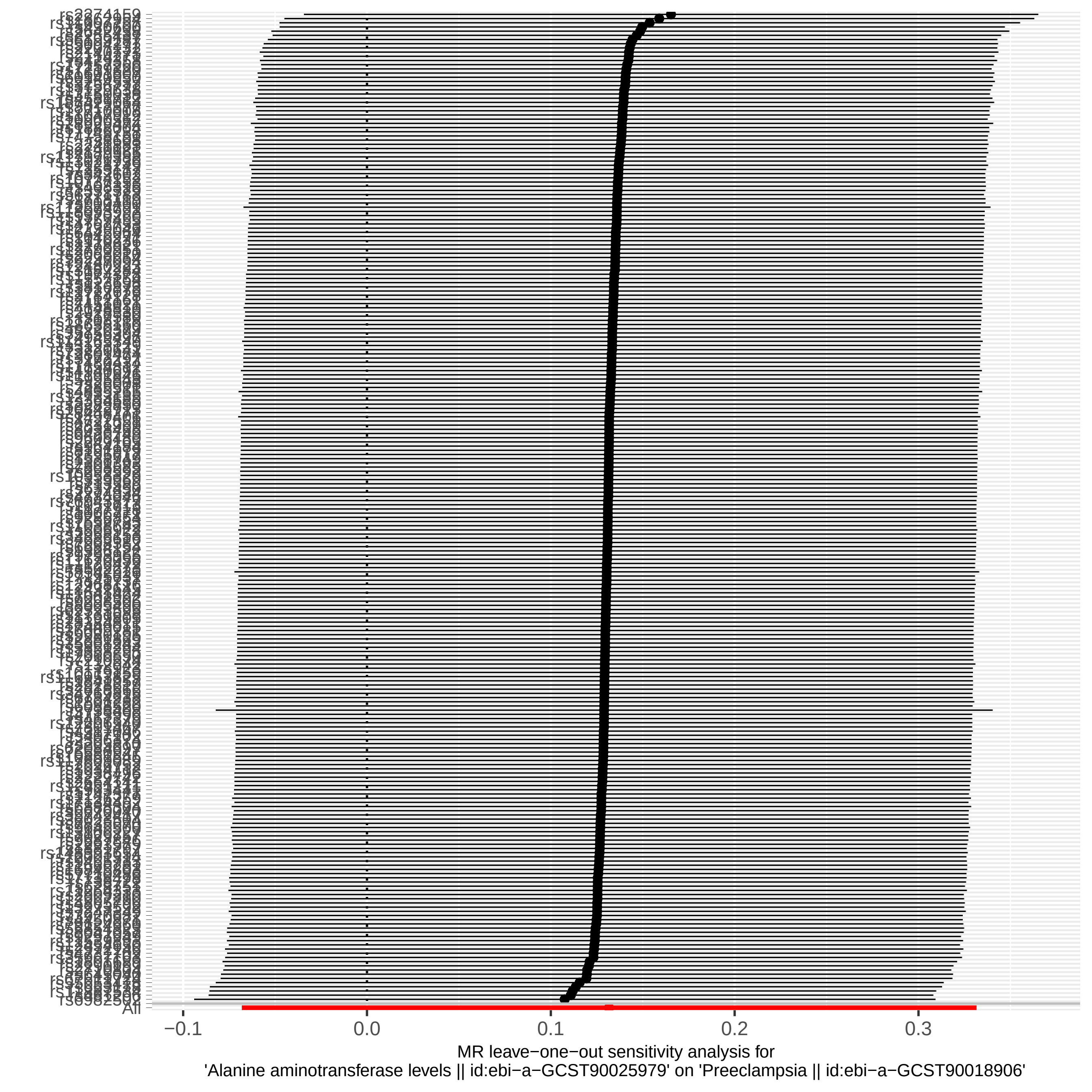
rs2274159
All
−0.1
0.0
0.1
0.2
0.3
MR leave−one−out sensitivity analysis for
'Alanine aminotransferase levels || id:ebi−a−GCST90025979' on 'Preeclampsia || id:ebi−a−GCST90018906'

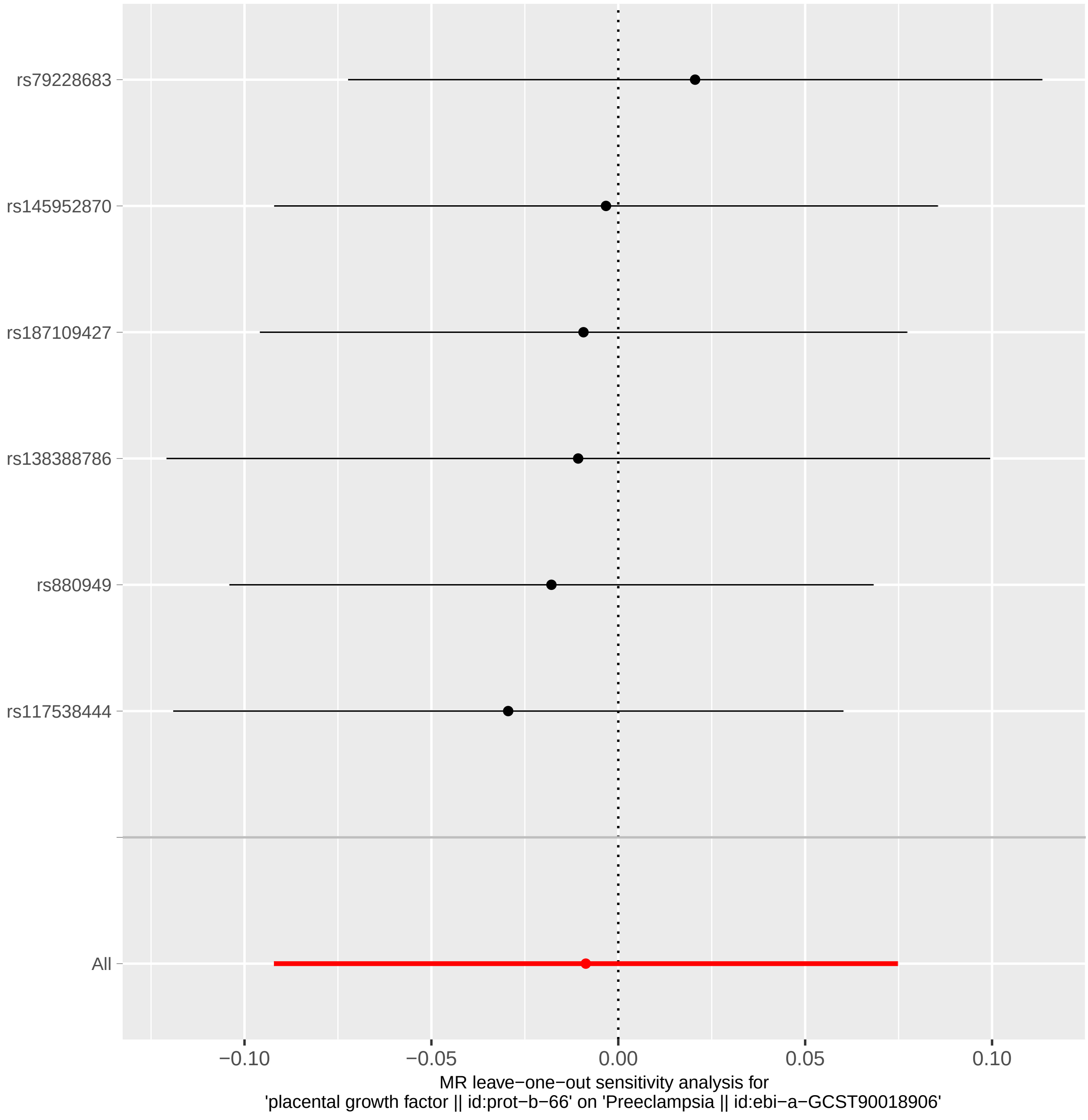
rs79228683
rs145952870
rs187109427
rs138388786
rs880949
rs117538444
All
−0.10
−0.05
0.00
0.05
0.10
MR leave−one−out sensitivity analysis for
'placental growth factor || id:prot−b−66' on 'Preeclampsia || id:ebi−a−GCST90018906'

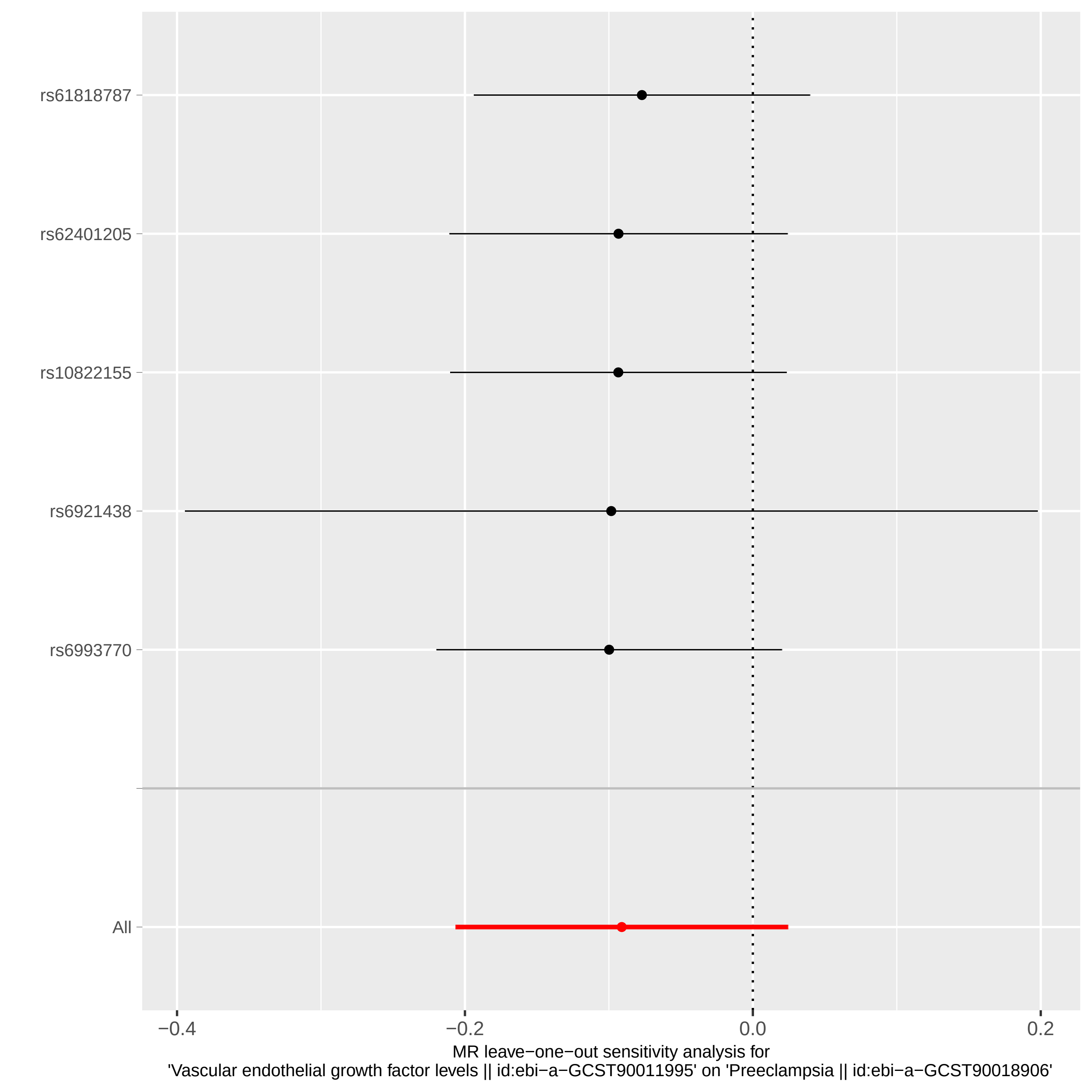
rs61818787
rs62401205
rs10822155
rs6921438
rs6993770
All
−0.4
−0.2
0.0
0.2
MR leave−one−out sensitivity analysis for
'Vascular endothelial growth factor levels || id:ebi−a−GCST90011995' on 'Preeclampsia || id:ebi−a−GCST90018906'

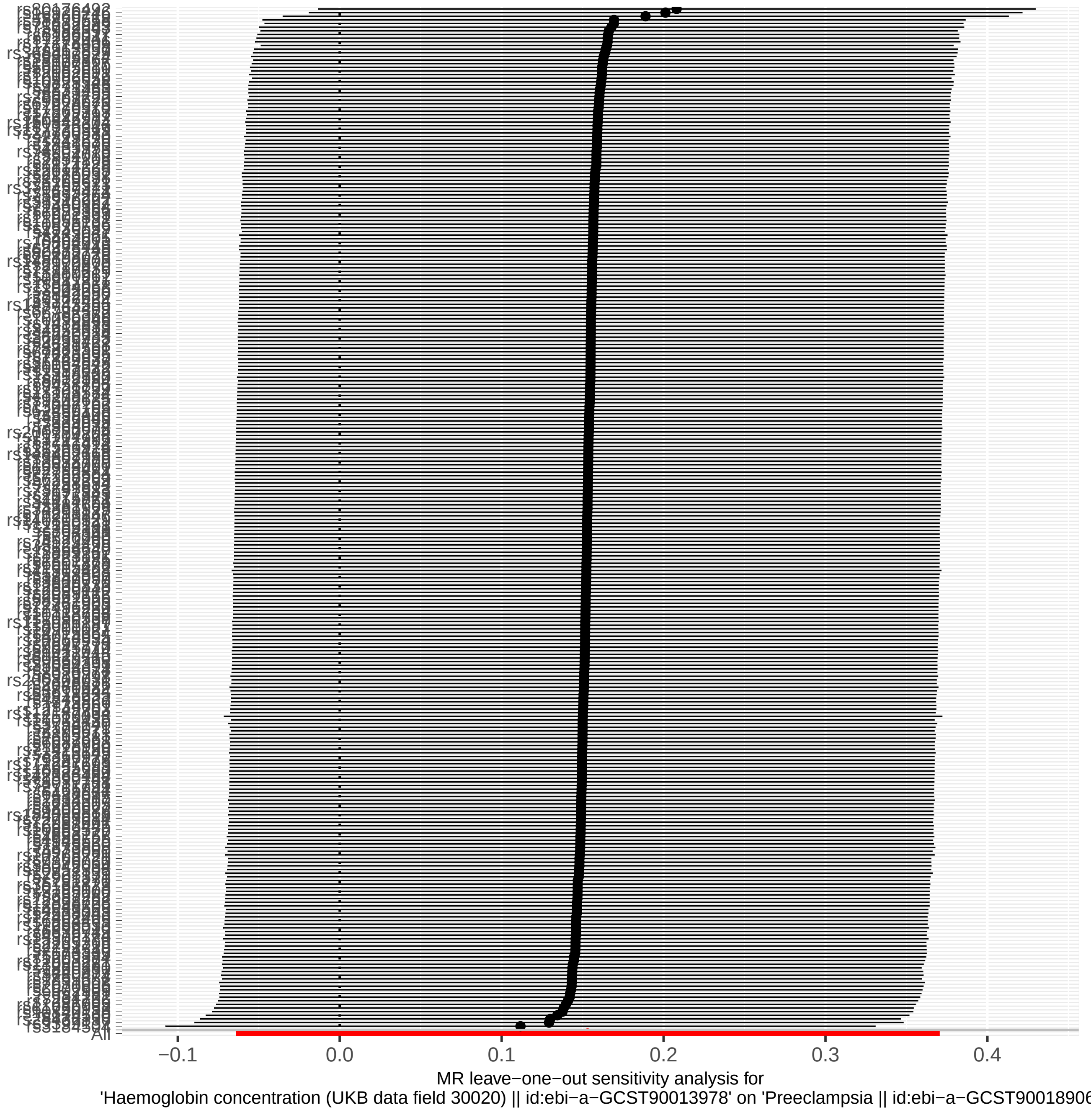
All
−0.1
0.0
0.1
0.2
0.3
0.4
MR leave−one−out sensitivity analysis for
'Haemoglobin concentration (UKB data field 30020) || id:ebi−a−GCST90013978' on 'Preeclampsia || id:ebi−a−GCST9001890

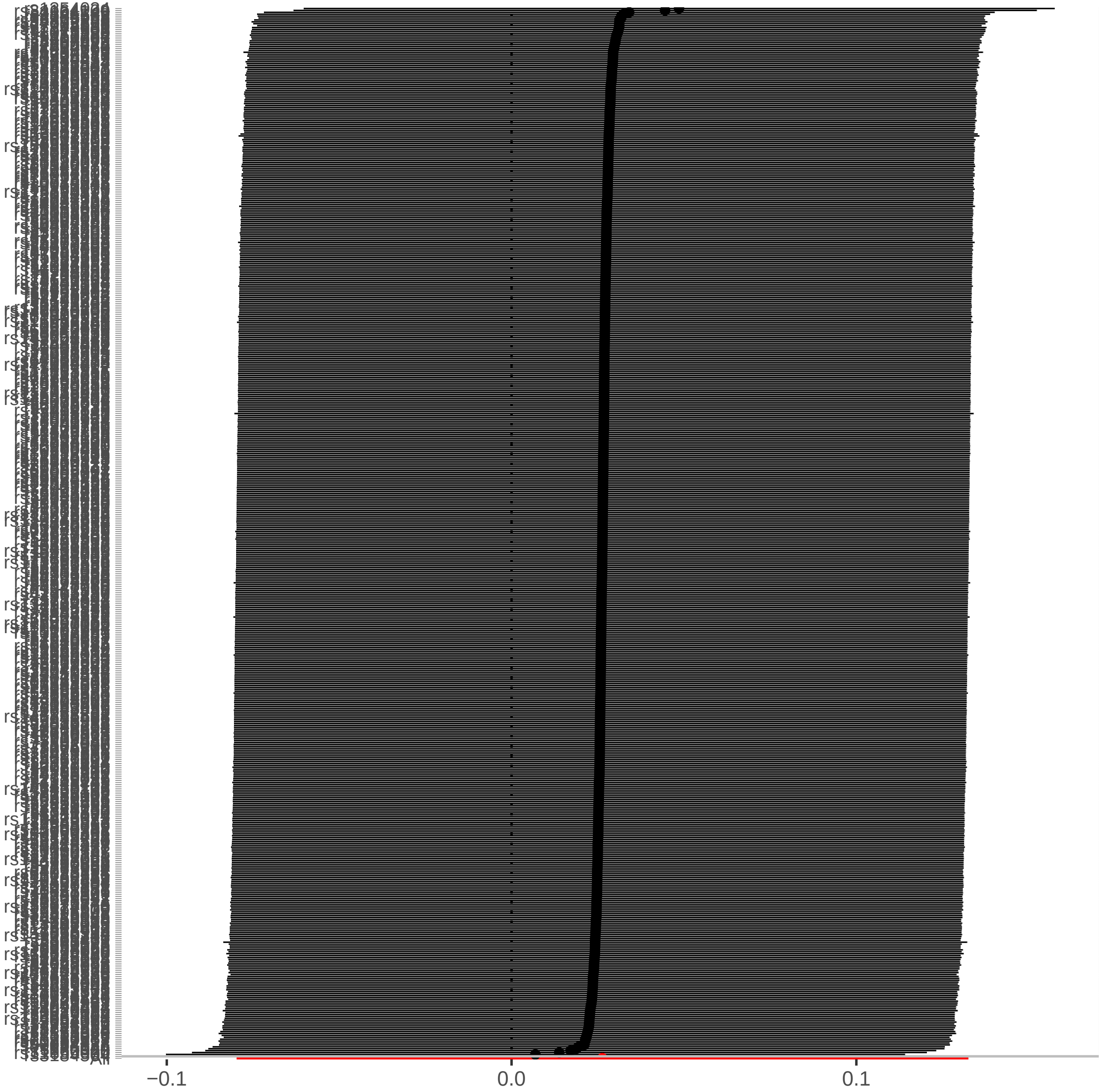

MR leave−one−out sensitivity analysis for
'Platelet count || id:ebi−a−GCST90028999' on 'Preeclampsia || id:ebi−a−GCST90018906'
