# Supplementary material for: Causal pathways in preeclampsia: a Mendelian randomization study in European populations
Source: Front Endocrinol (Lausanne). 2024 Sep 2;15:1453277. doi: 10.3389/fendo.2024.1453277 (PMC11402816; doi:10.3389/fendo.2024.1453277)
Supplement: Supplementary file 4 [file DataSheet4.pdf]

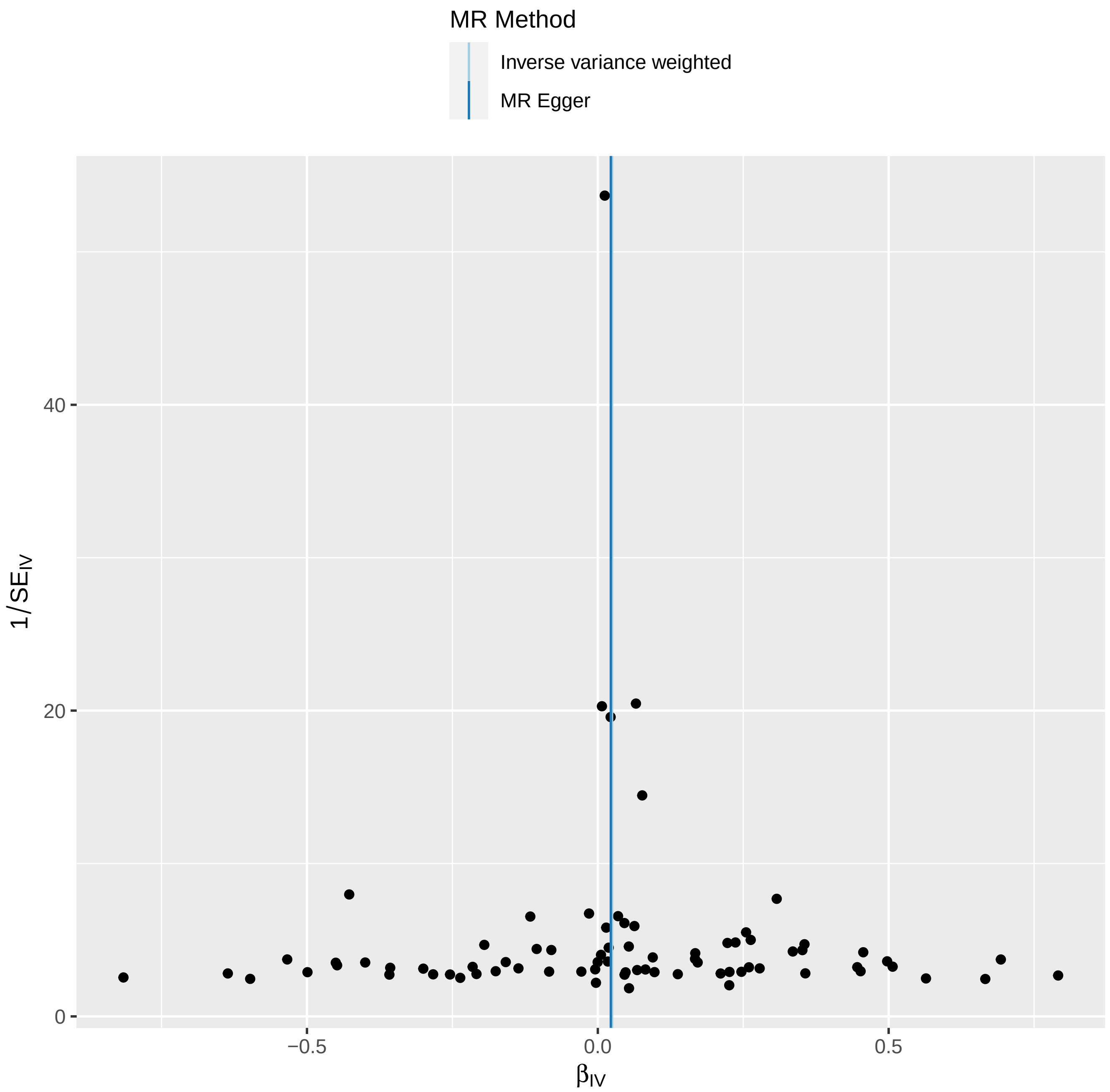

MR Method
Inverse variance weighted
MR Egger
40
20
0
1/SE$_{IV}$
−0.5
0.0
0.5
β_{IV}

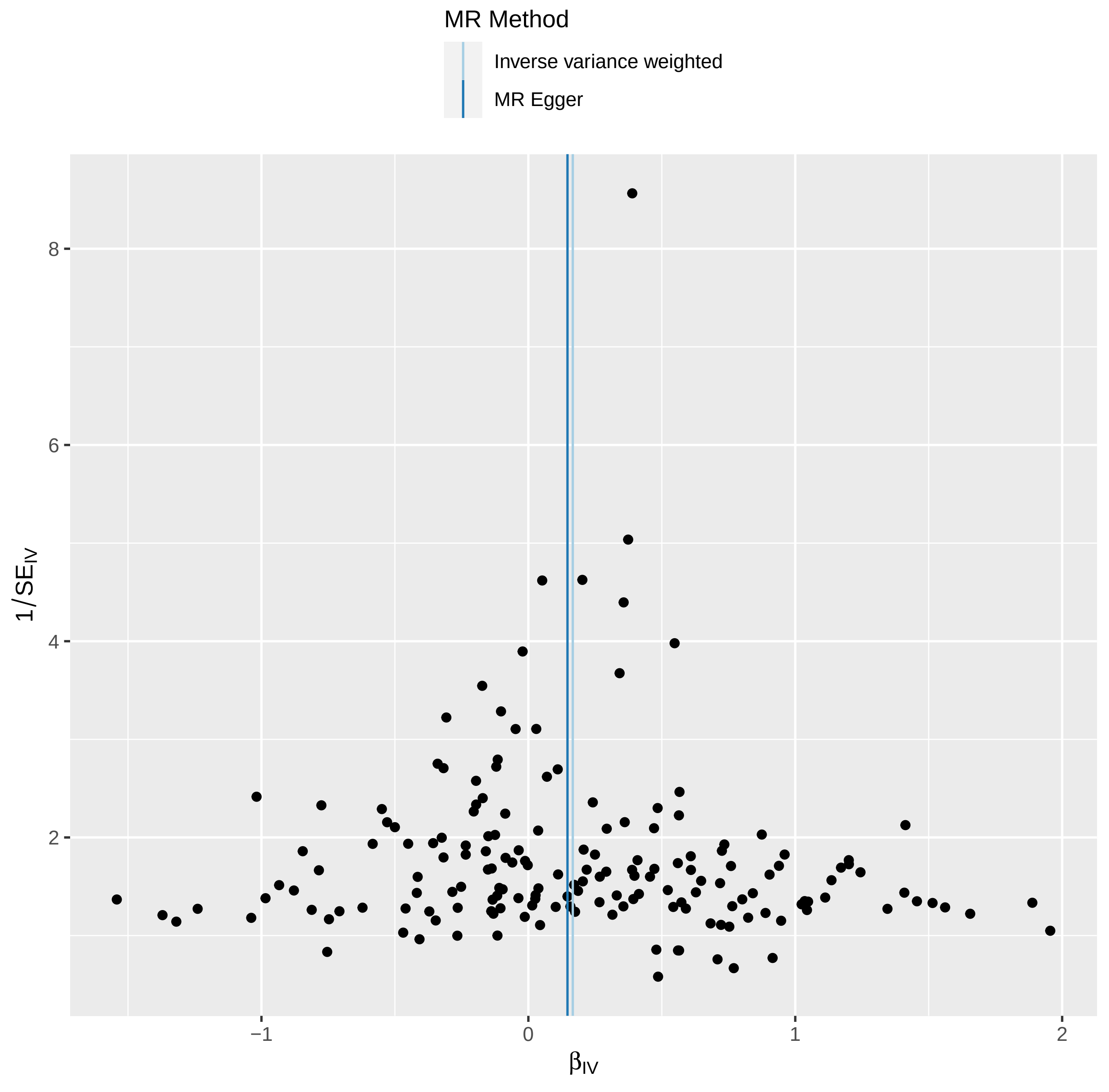

MR Method
Inverse variance weighted
MR Egger
8
6
4
2
1/SE_IV
−1
0
1
2
β_IV

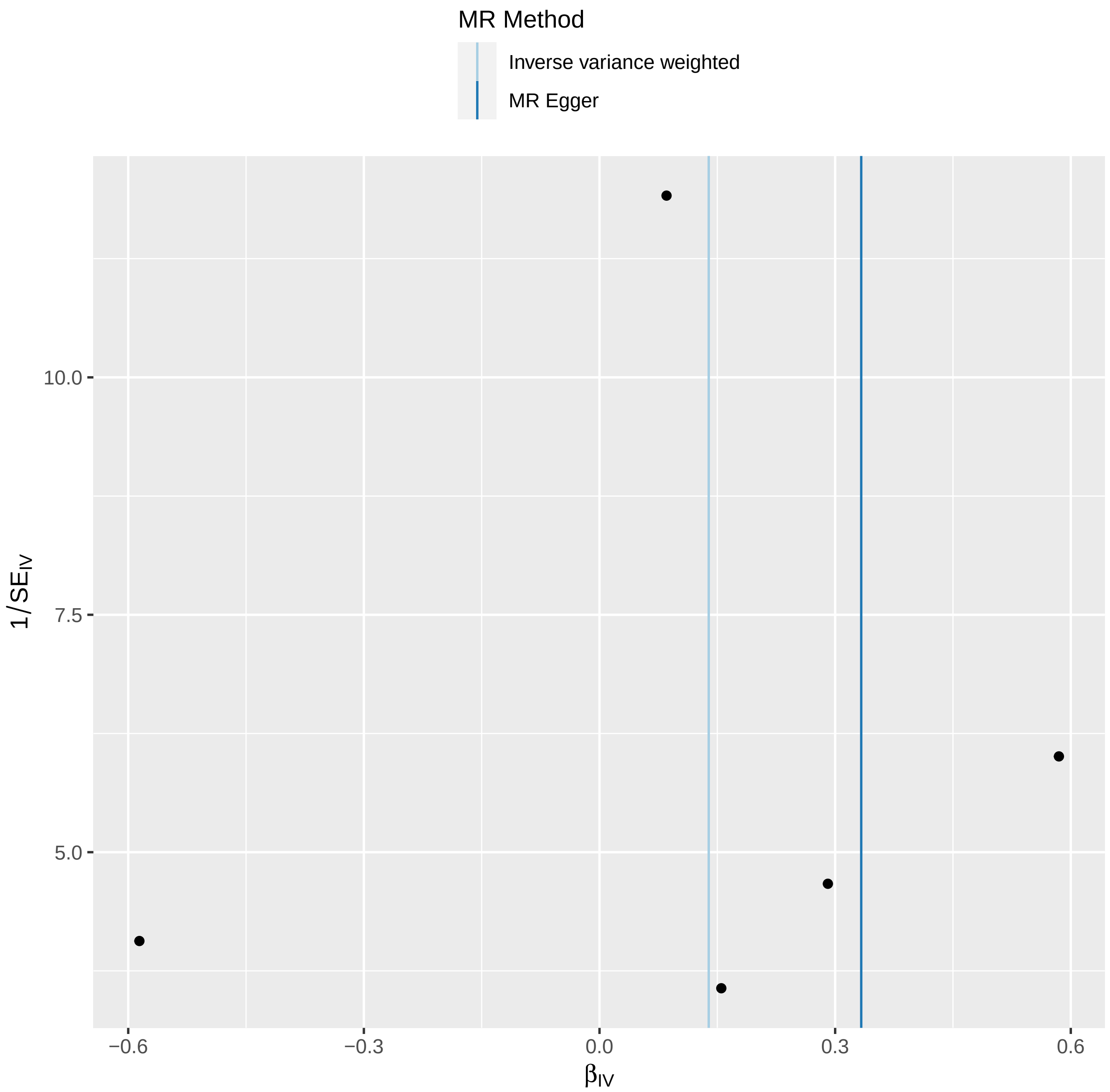

MR Method
Inverse variance weighted
MR Egger
10.0
7.5
5.0
−0.6
−0.3
0.0
0.3
0.6
$1/SE_{IV}$
β_{IV}

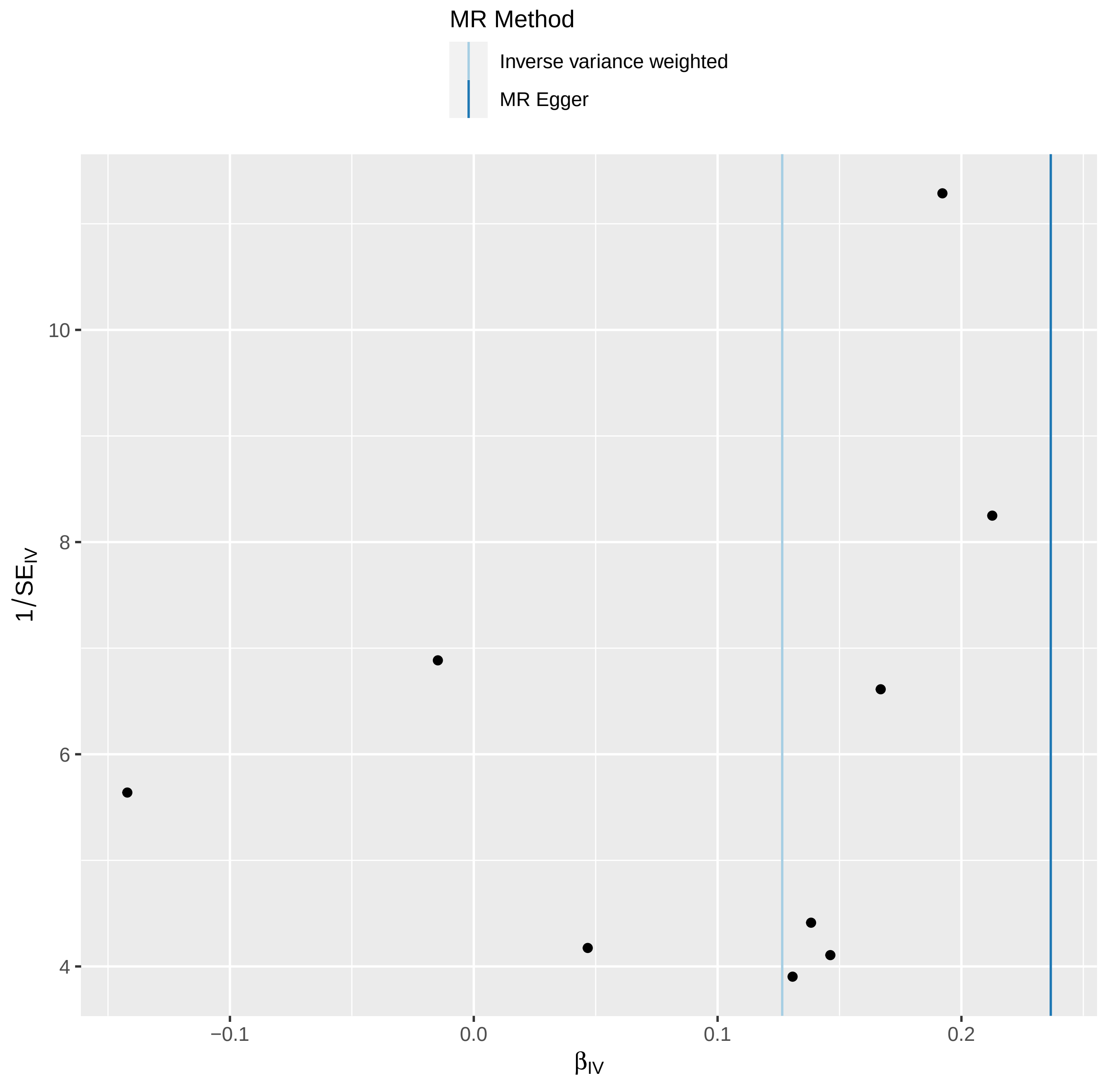

MR Method
Inverse variance weighted
MR Egger
10
8
6
4
1/SE_IV
−0.1
0.0
0.1
0.2
β_IV

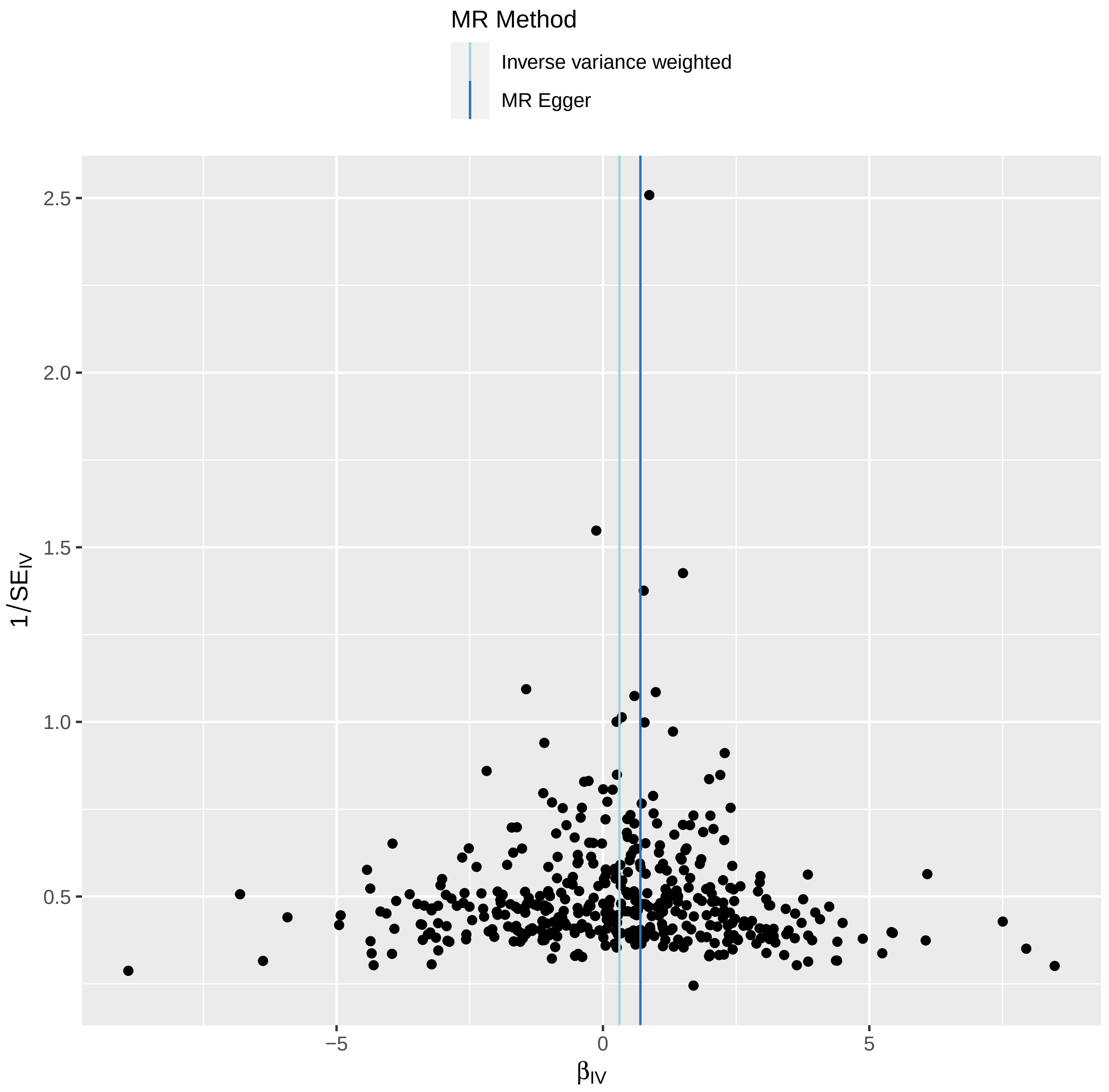

MR Method
Inverse variance weighted
MR Egger
1/SE_IV
2.5
2.0
1.5
1.0
0.5
−5
0
5
β_IV

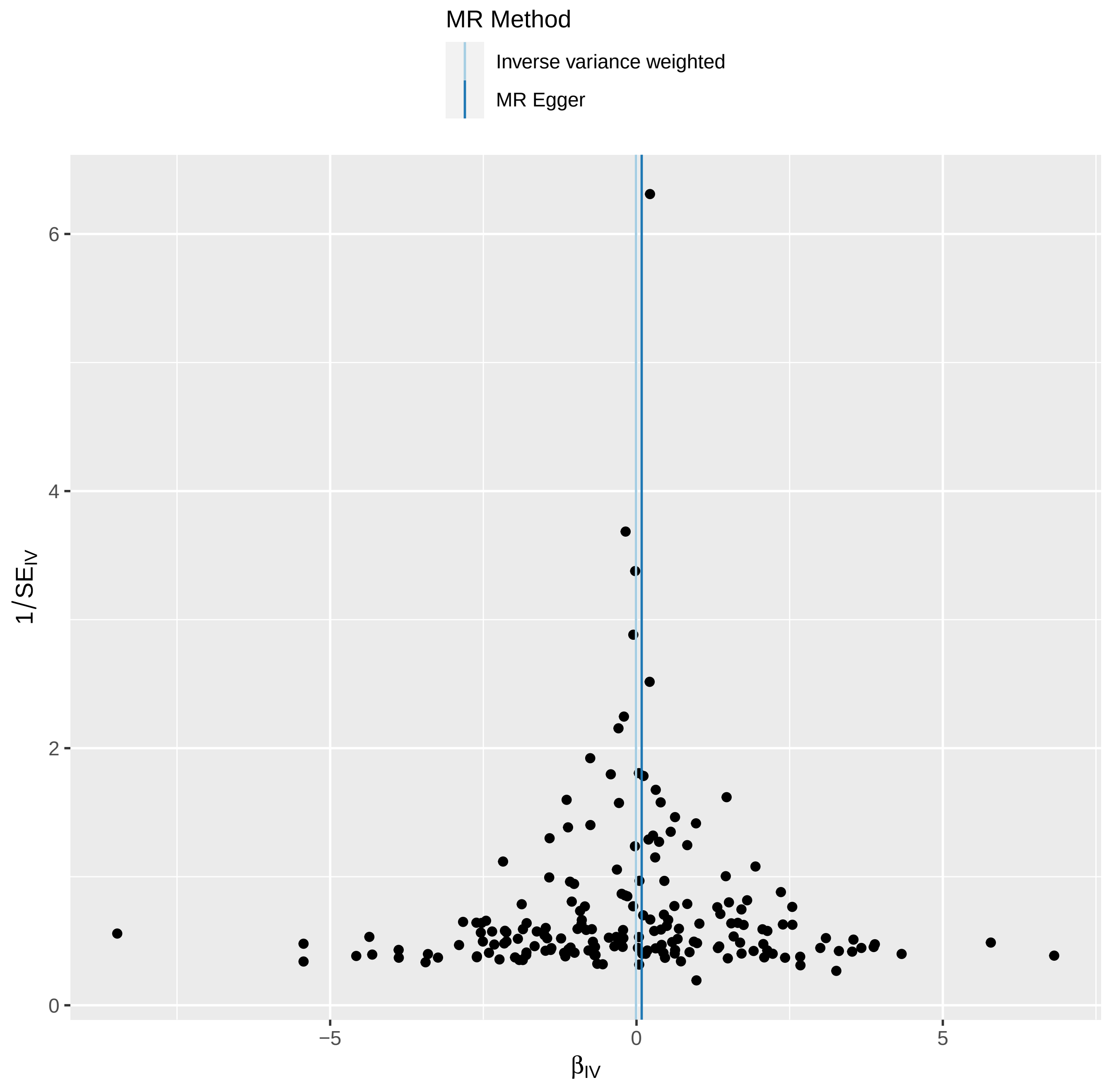
MR Method
Inverse variance weighted
MR Egger
6
4
2
0
−5
0
5
1/SE$_{IV}$
β$_{IV}$

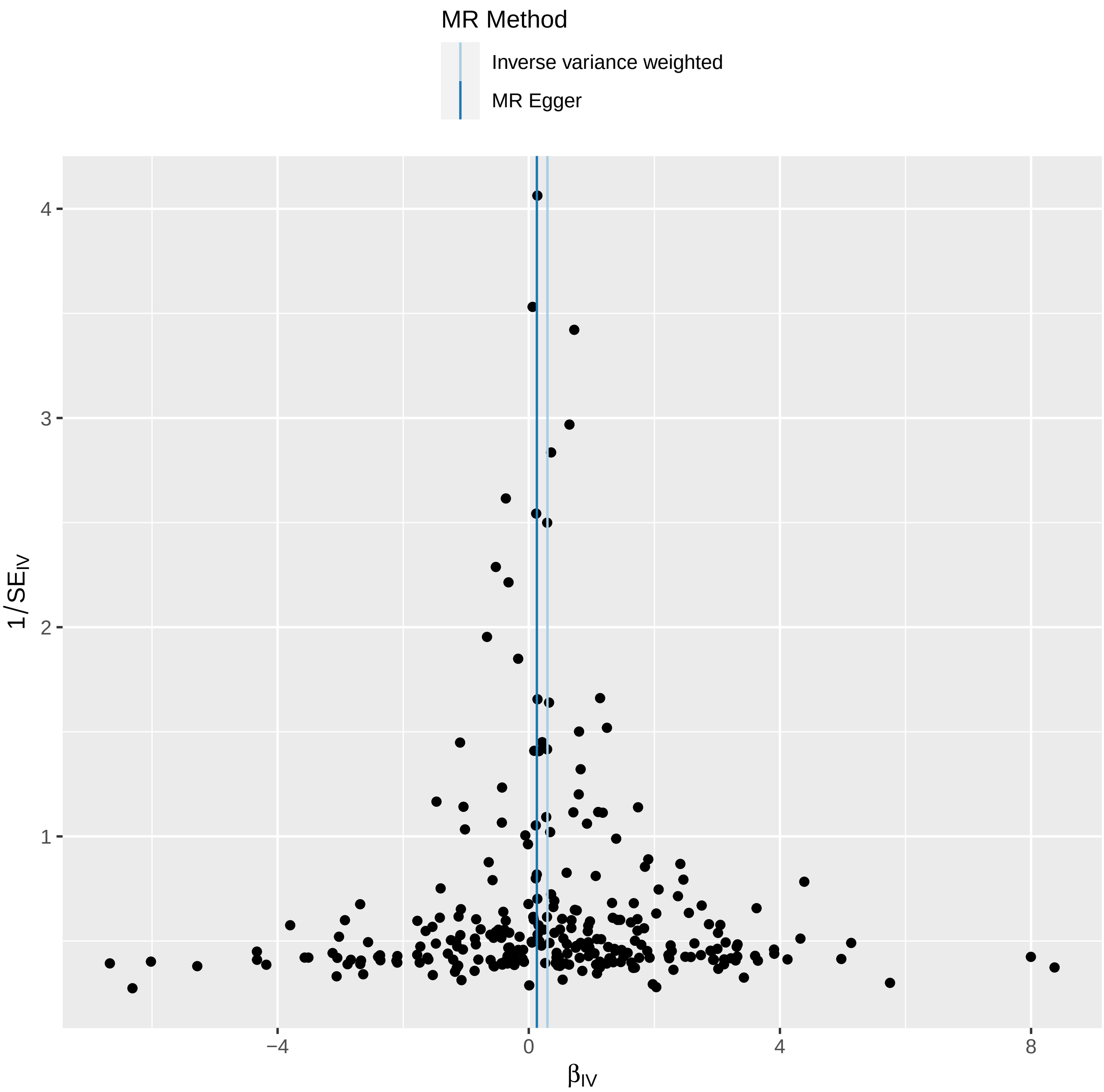

MR Method
Inverse variance weighted
MR Egger
4
3
2
1
1/SE_IV
−4
0
4
8
β_IV

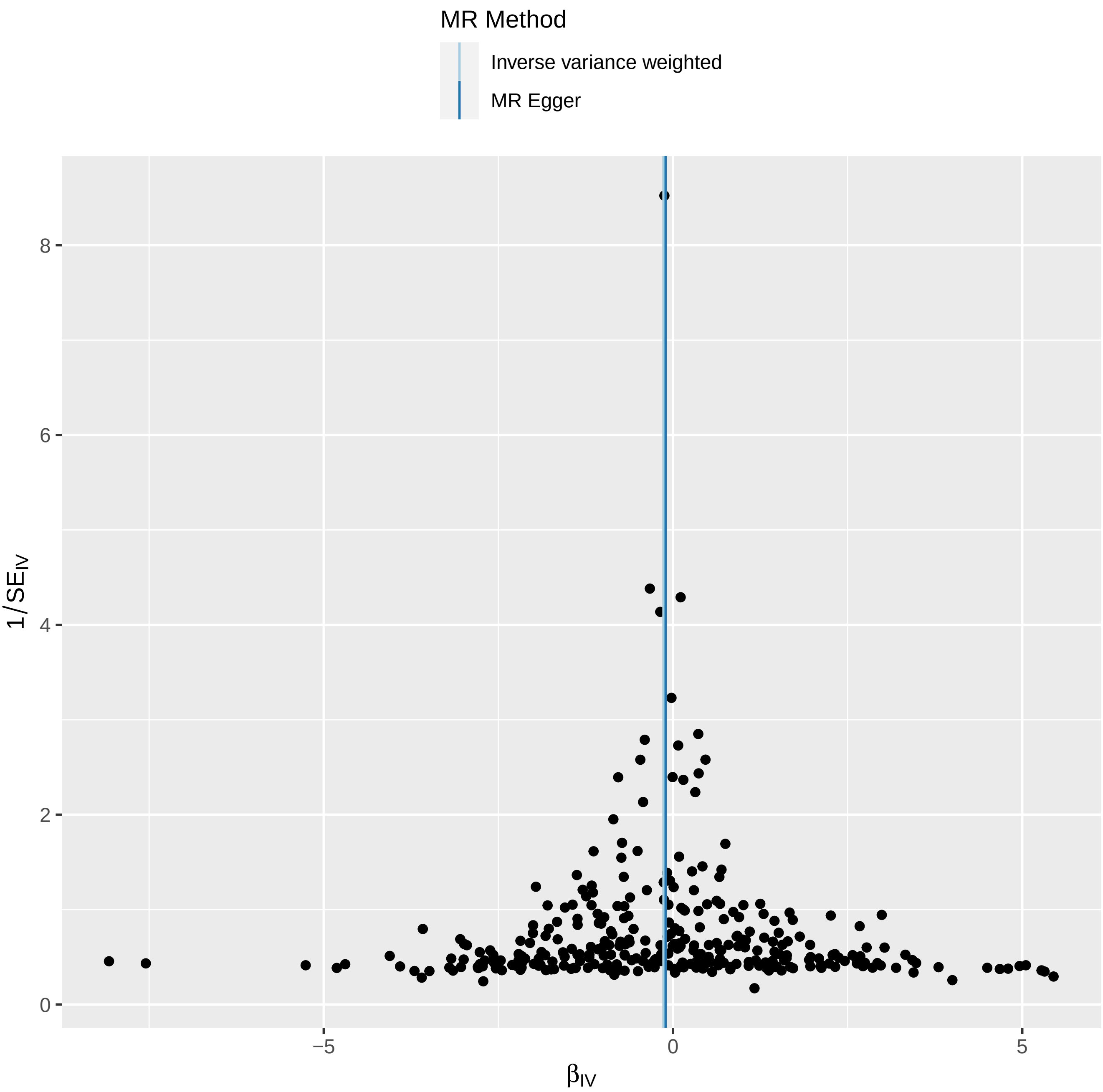
MR Method
Inverse variance weighted
MR Egger
1/SE_IV
8
6
4
2
0
−5
0
5
β_IV

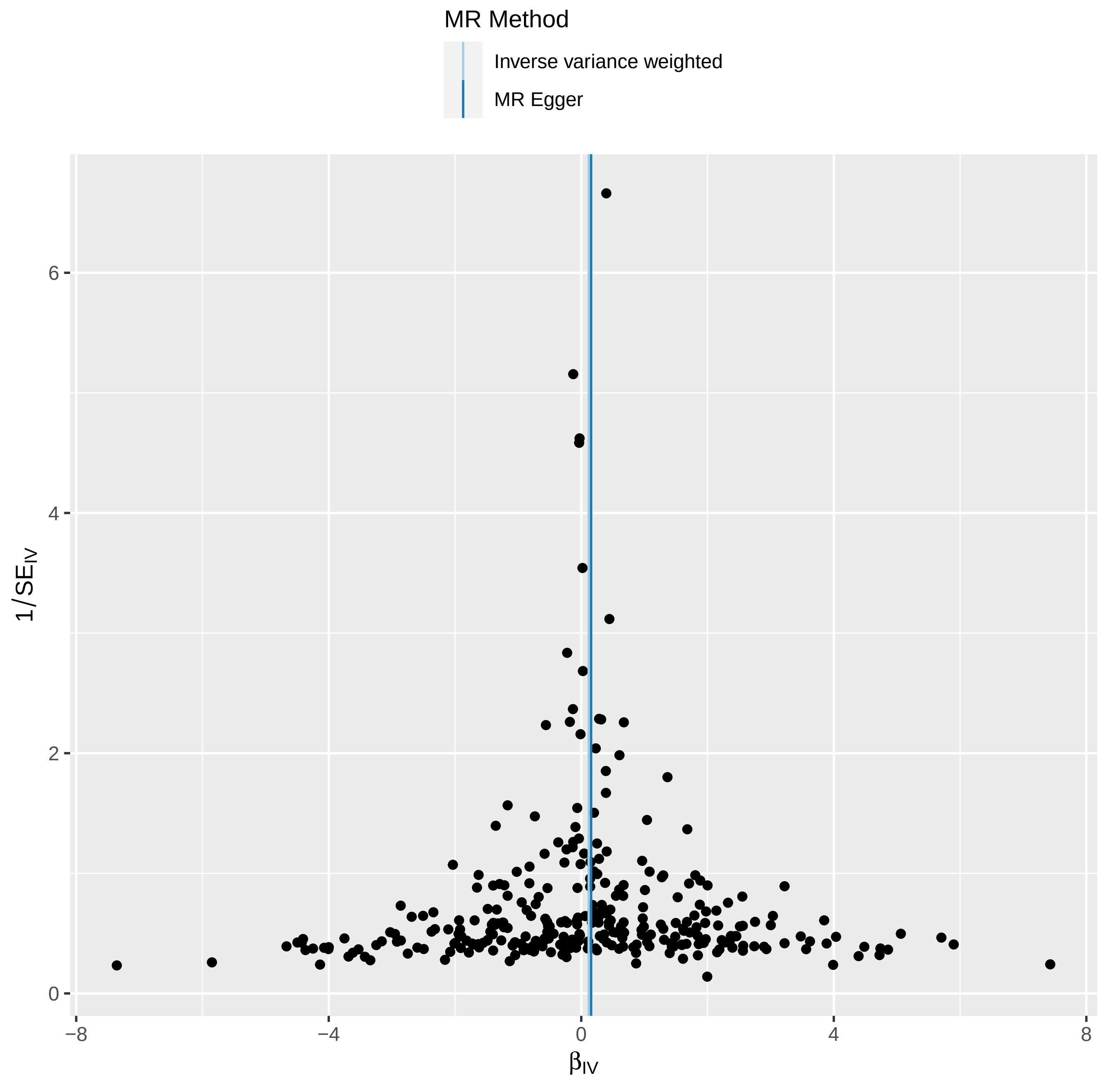

MR Method
Inverse variance weighted
MR Egger
6
4
2
0
$1/SE_{IV}$
−8
−4
0
4
8
β_{IV}

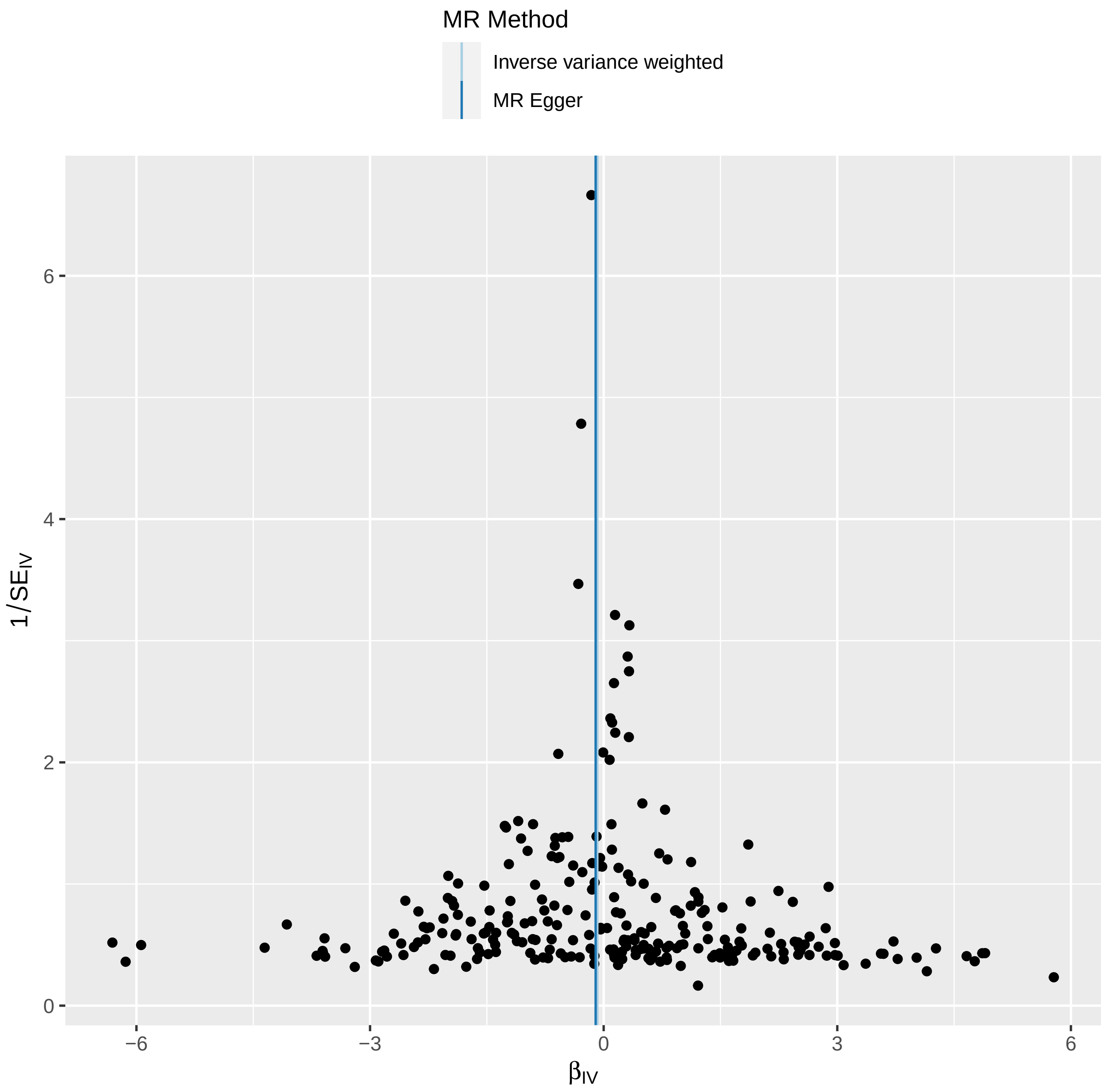
MR Method
Inverse variance weighted
MR Egger
6
4
2
0
−6
−3
0
3
6
1/SE_IV
β_IV

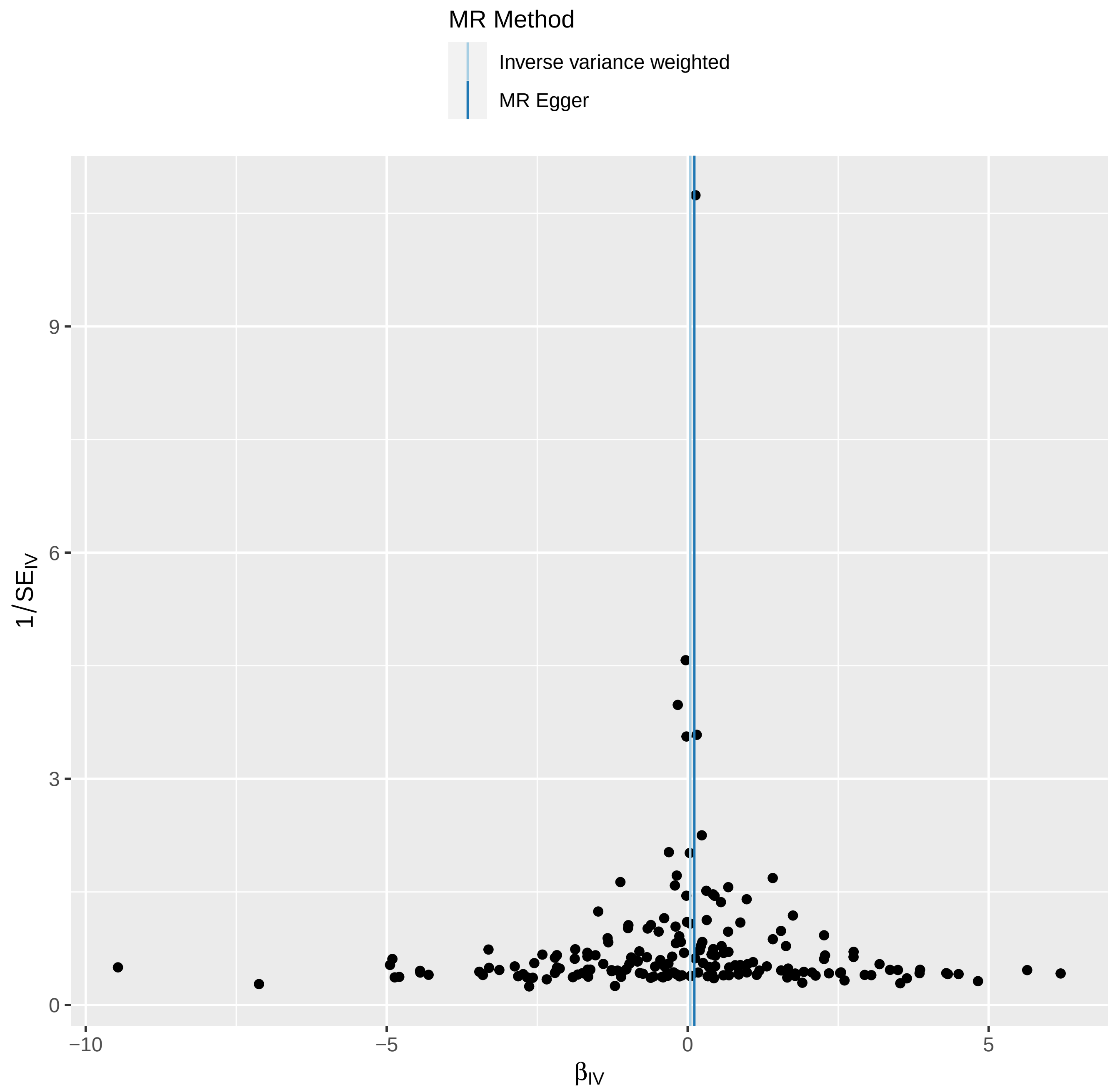
MR Method
Inverse variance weighted
MR Egger
1/SE_IV
β_IV
−10
−5
0
5
0
3
6
9

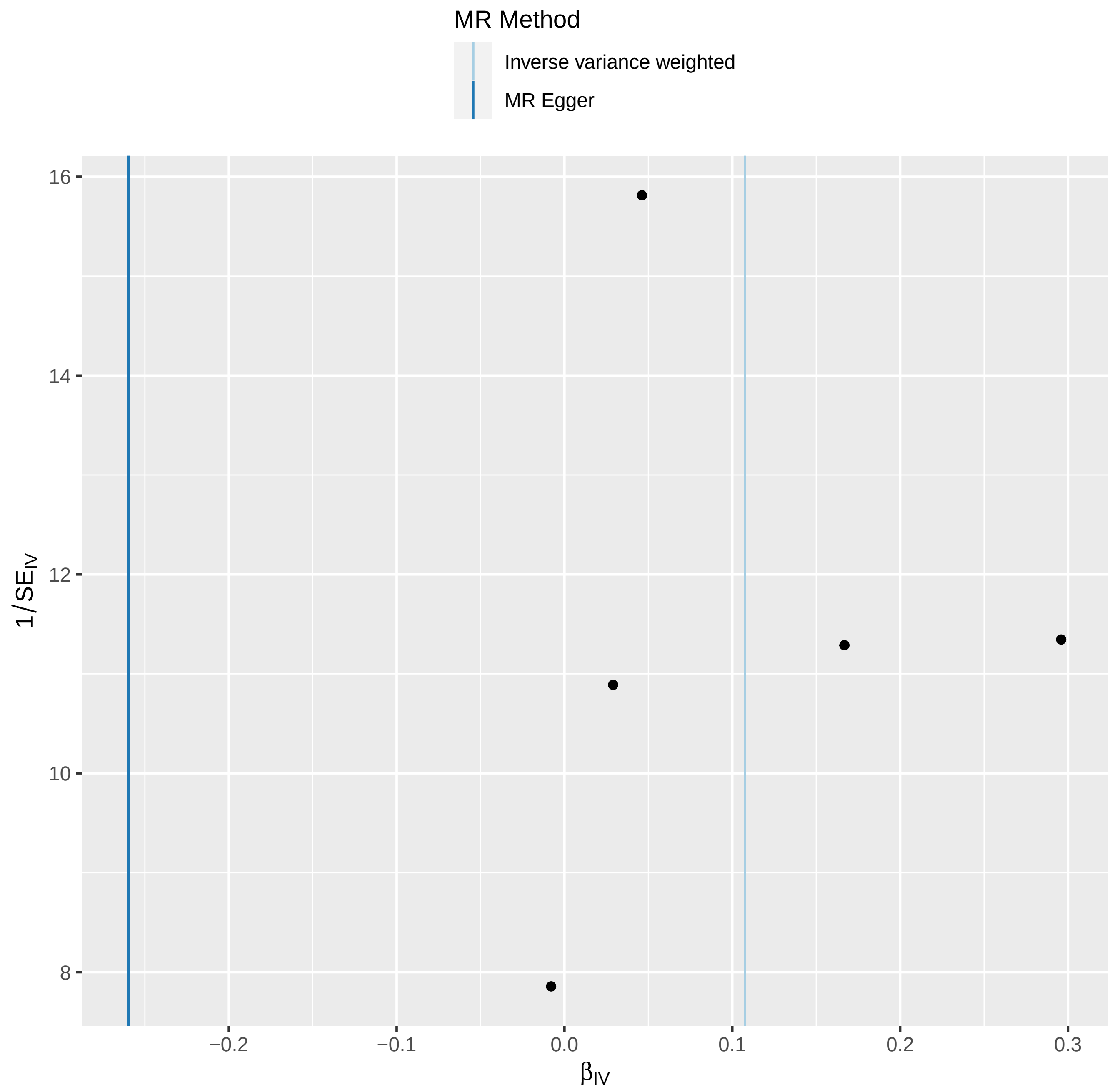
MR Method
Inverse variance weighted
MR Egger
16
14
12
10
8
$1/SE_{IV}$
−0.2
−0.1
0.0
0.1
0.2
0.3
β_{IV}

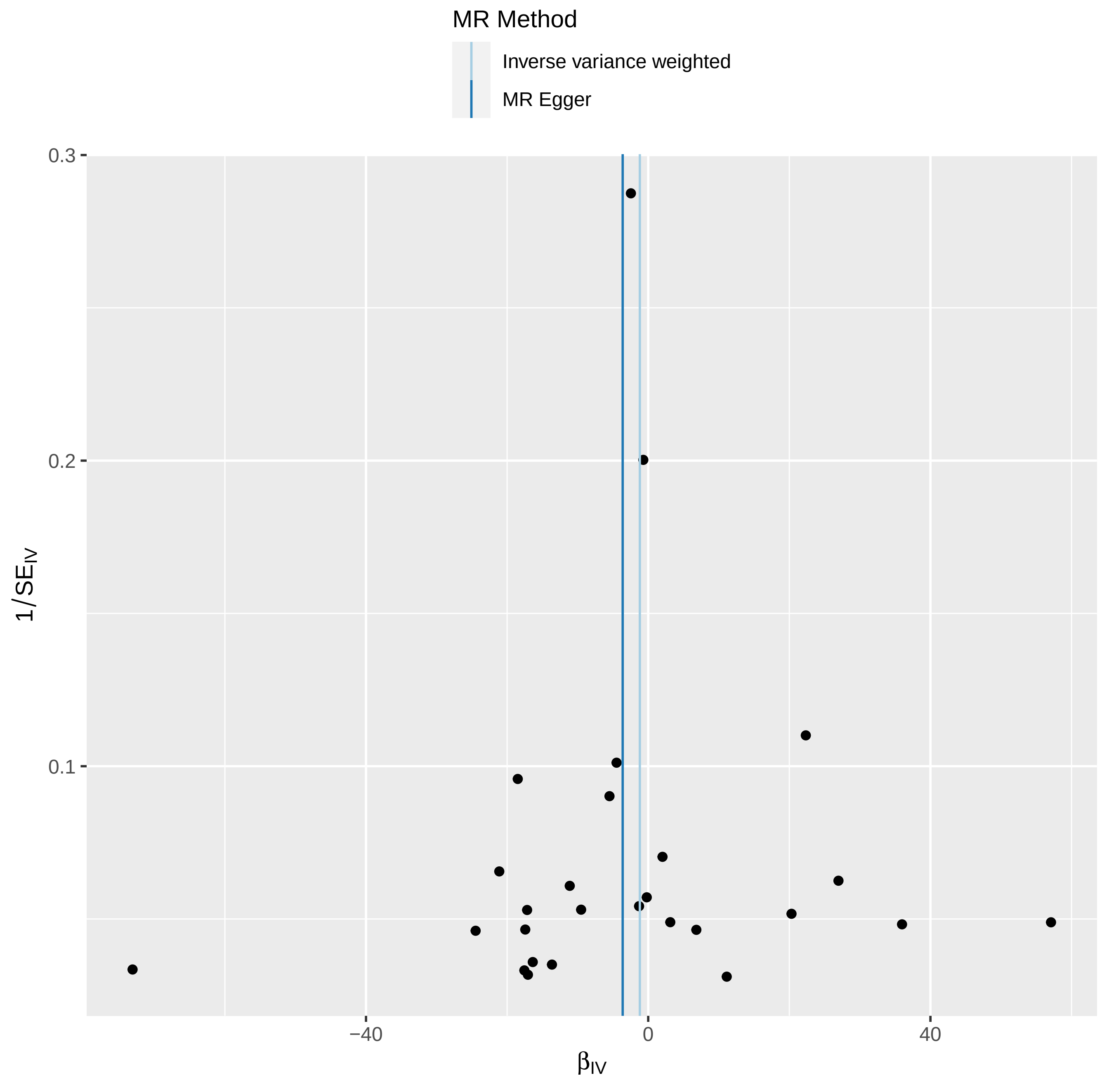

MR Method
Inverse variance weighted
MR Egger
0.3
0.2
0.1
1/SE_IV
−40
0
40
β_IV

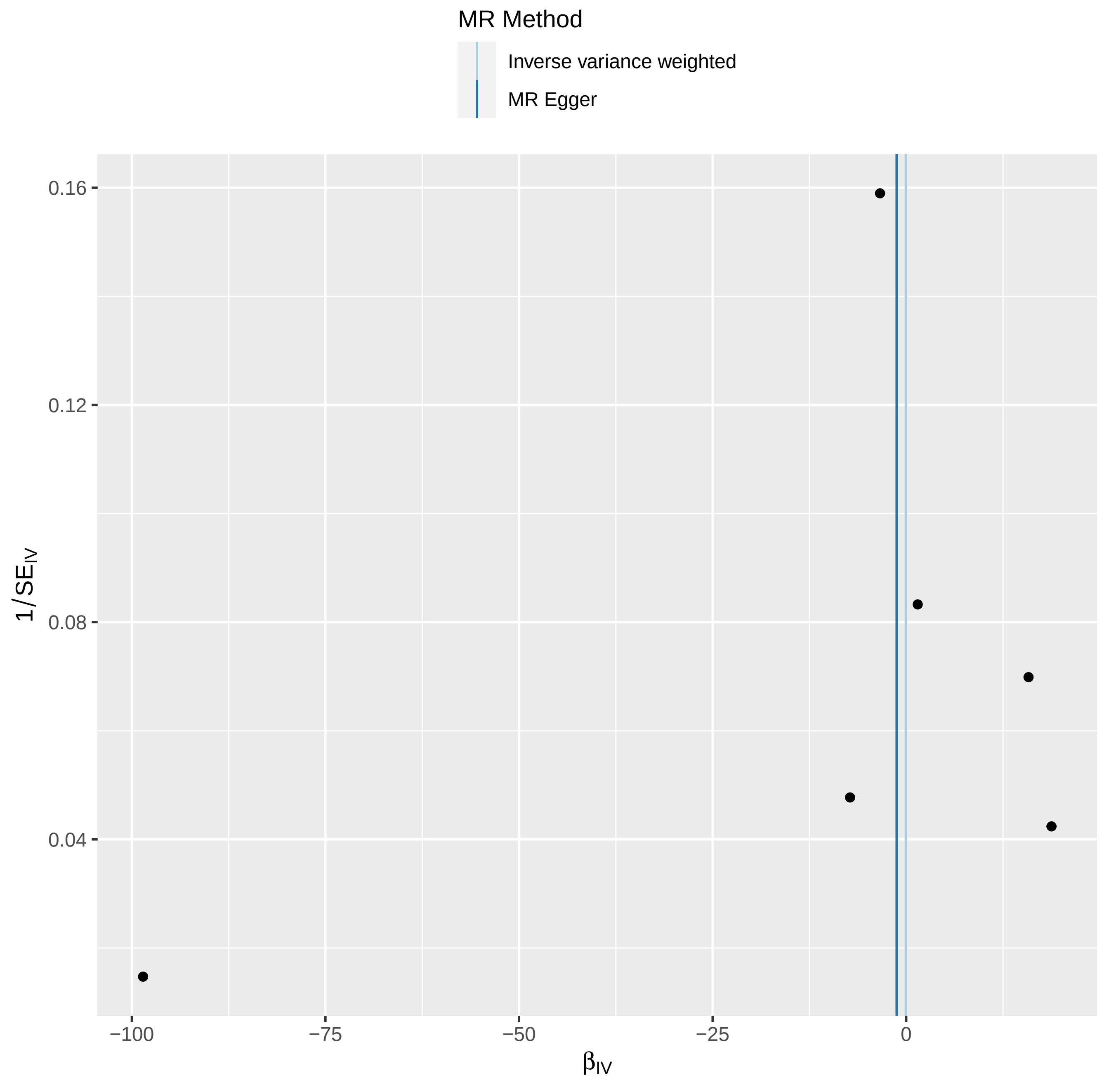

MR Method
Inverse variance weighted
MR Egger
0.16
0.12
0.08
0.04
1/SE_IV
−100
−75
−50
−25
0
β_IV

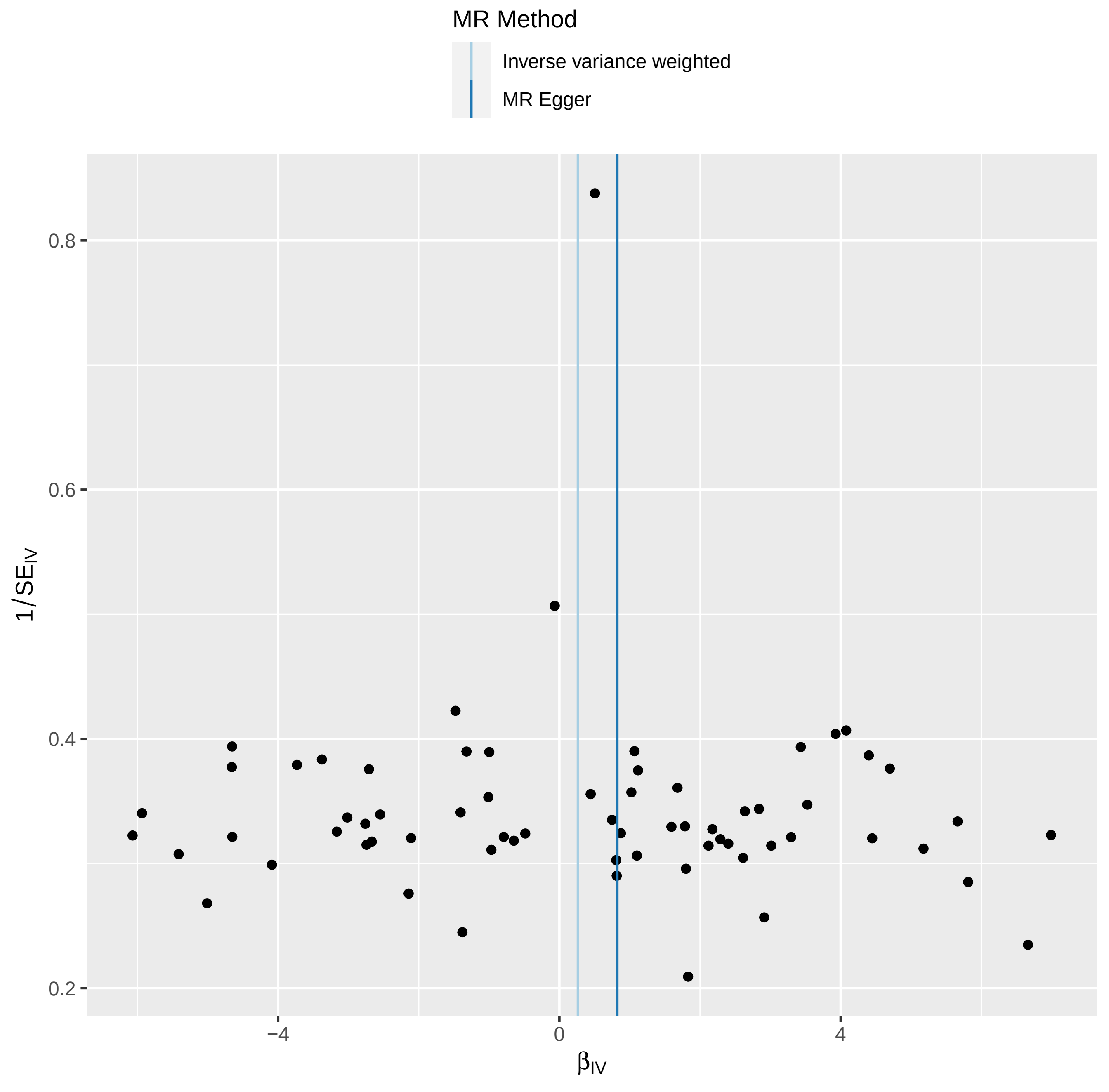

MR Method
Inverse variance weighted
MR Egger
0.8
0.6
0.4
0.2
1/SE$_{IV}$
−4
0
4
β$_{IV}$

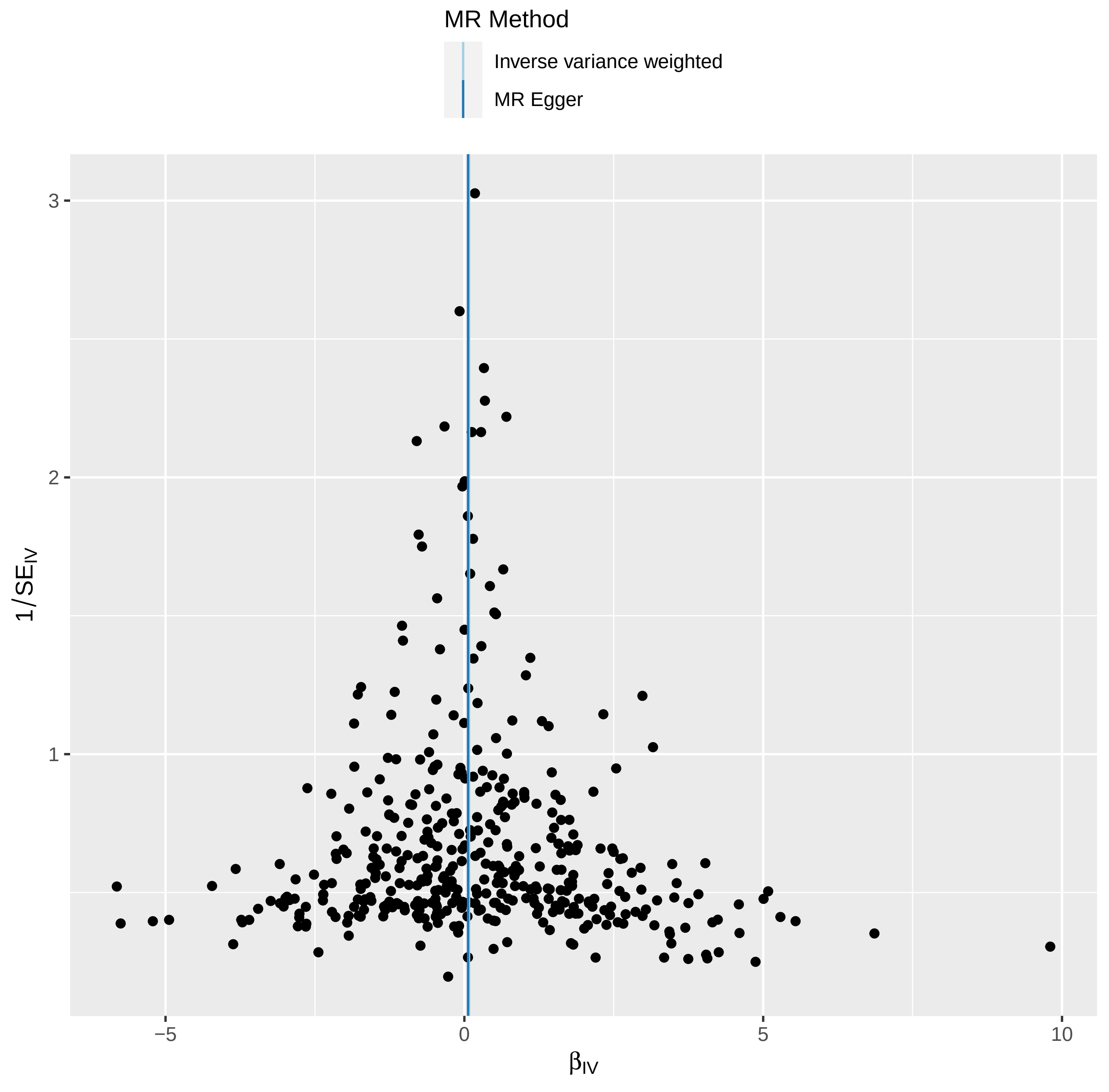
MR Method
Inverse variance weighted
MR Egger
3
2
1
1/SE$_{IV}$
−5
0
5
10
β$_{IV}$

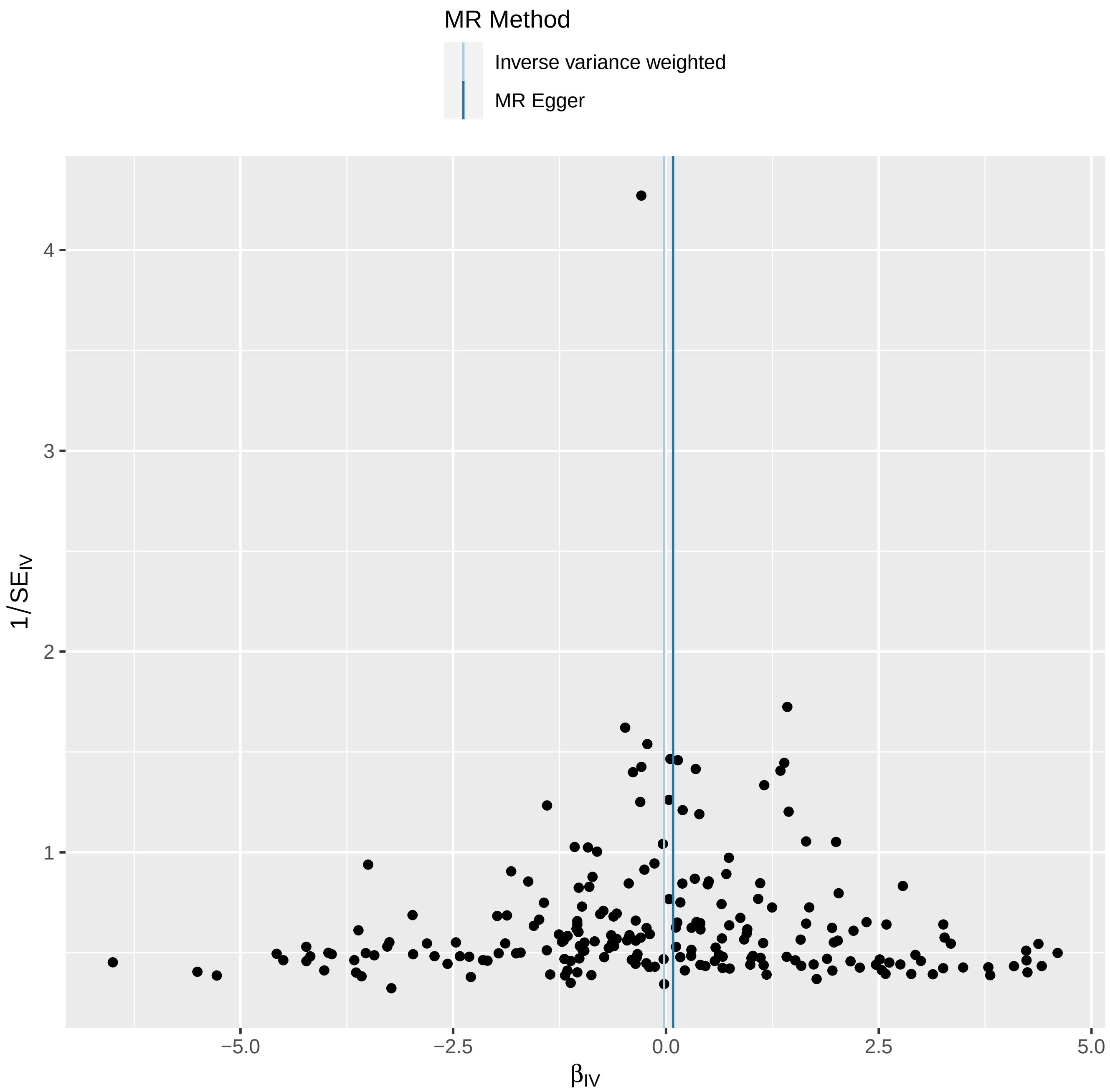

MR Method
Inverse variance weighted
MR Egger
$1/SE_{IV}$
4
3
2
1
−5.0
−2.5
0.0
2.5
5.0
β_{IV}

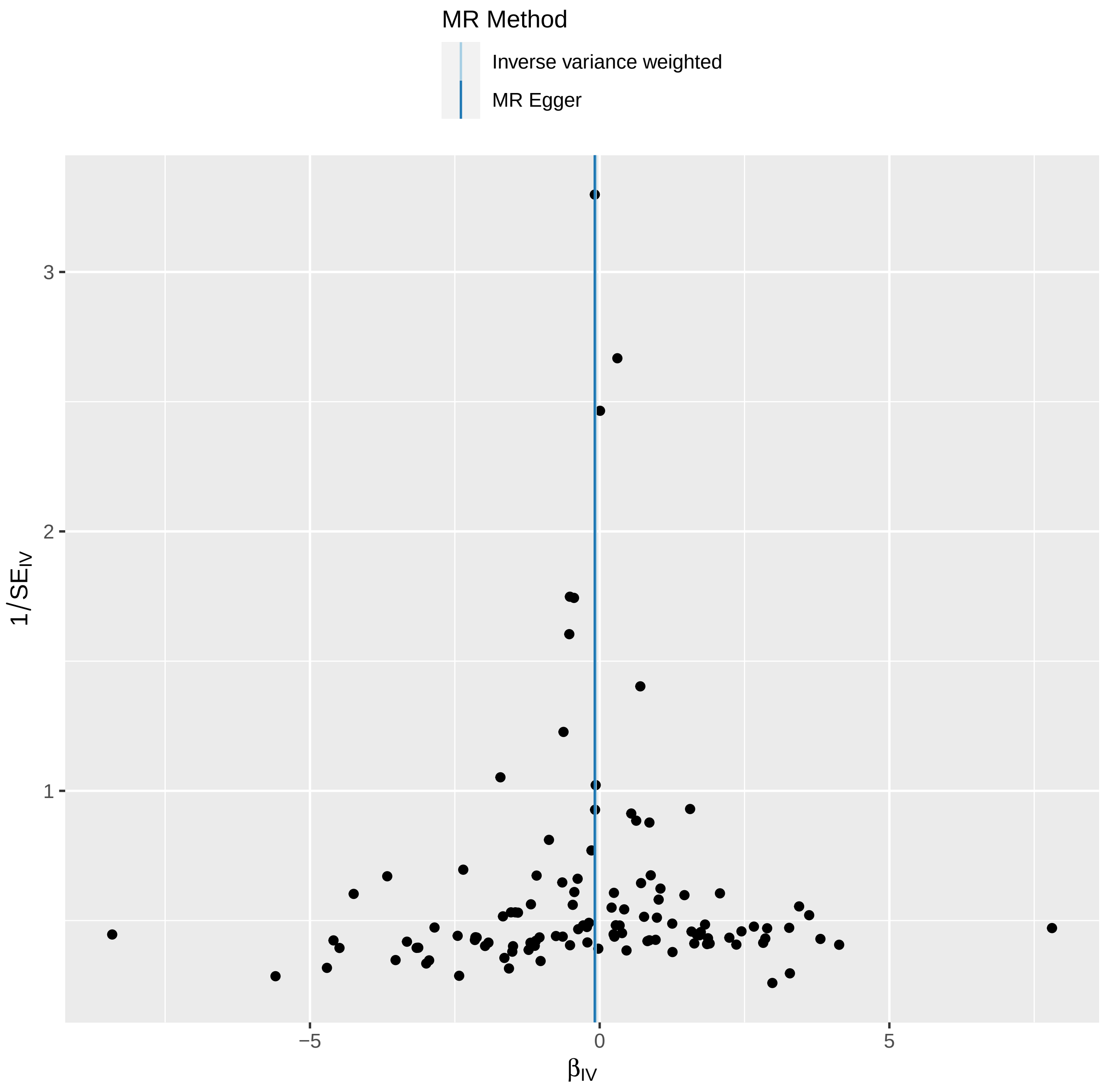
MR Method
Inverse variance weighted
MR Egger
3
2
1
1/SE_IV
−5
0
5
β_IV

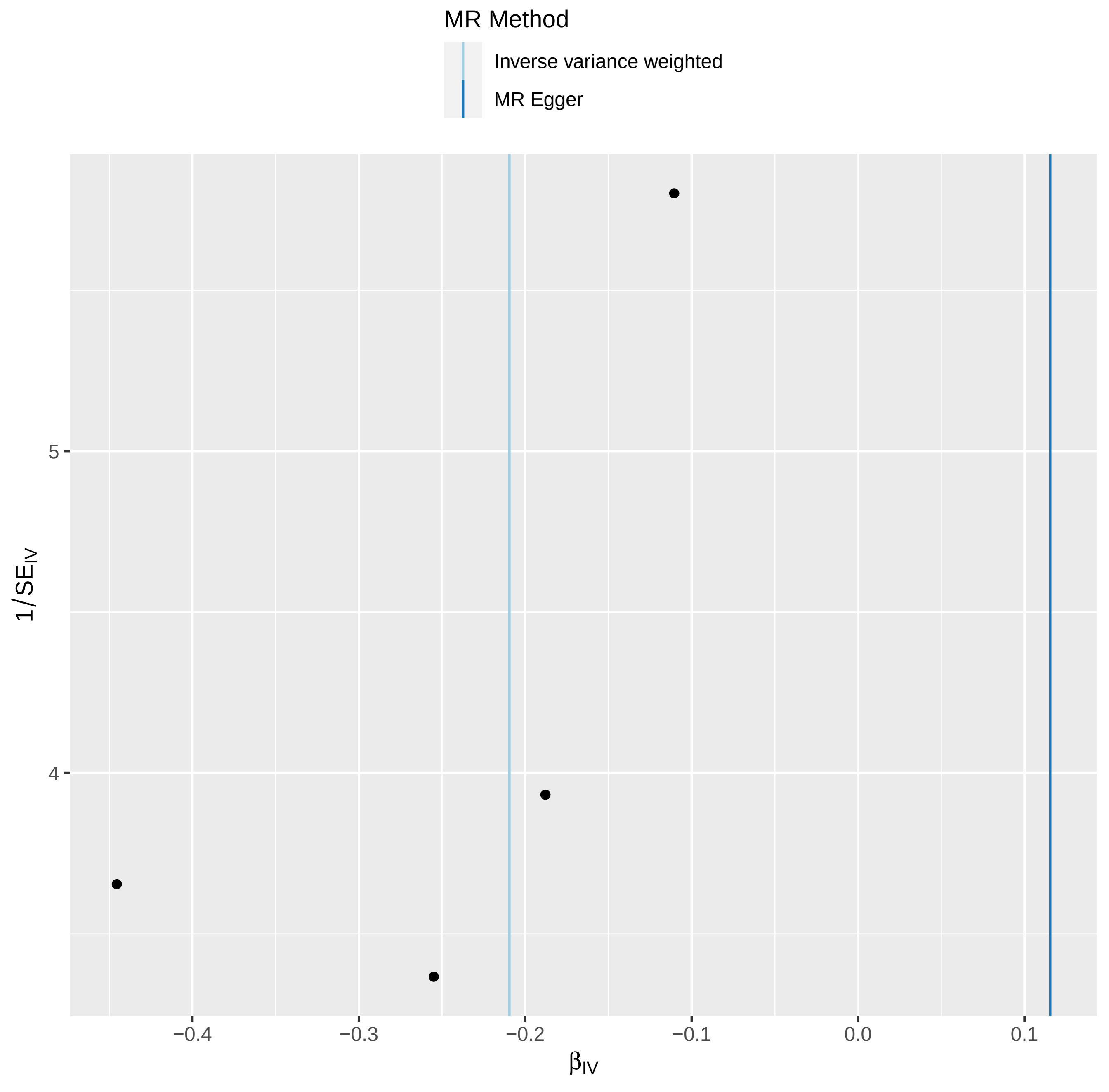

MR Method
Inverse variance weighted
MR Egger
5
4
1/SE_IV
−0.4
−0.3
−0.2
−0.1
0.0
0.1
β_IV

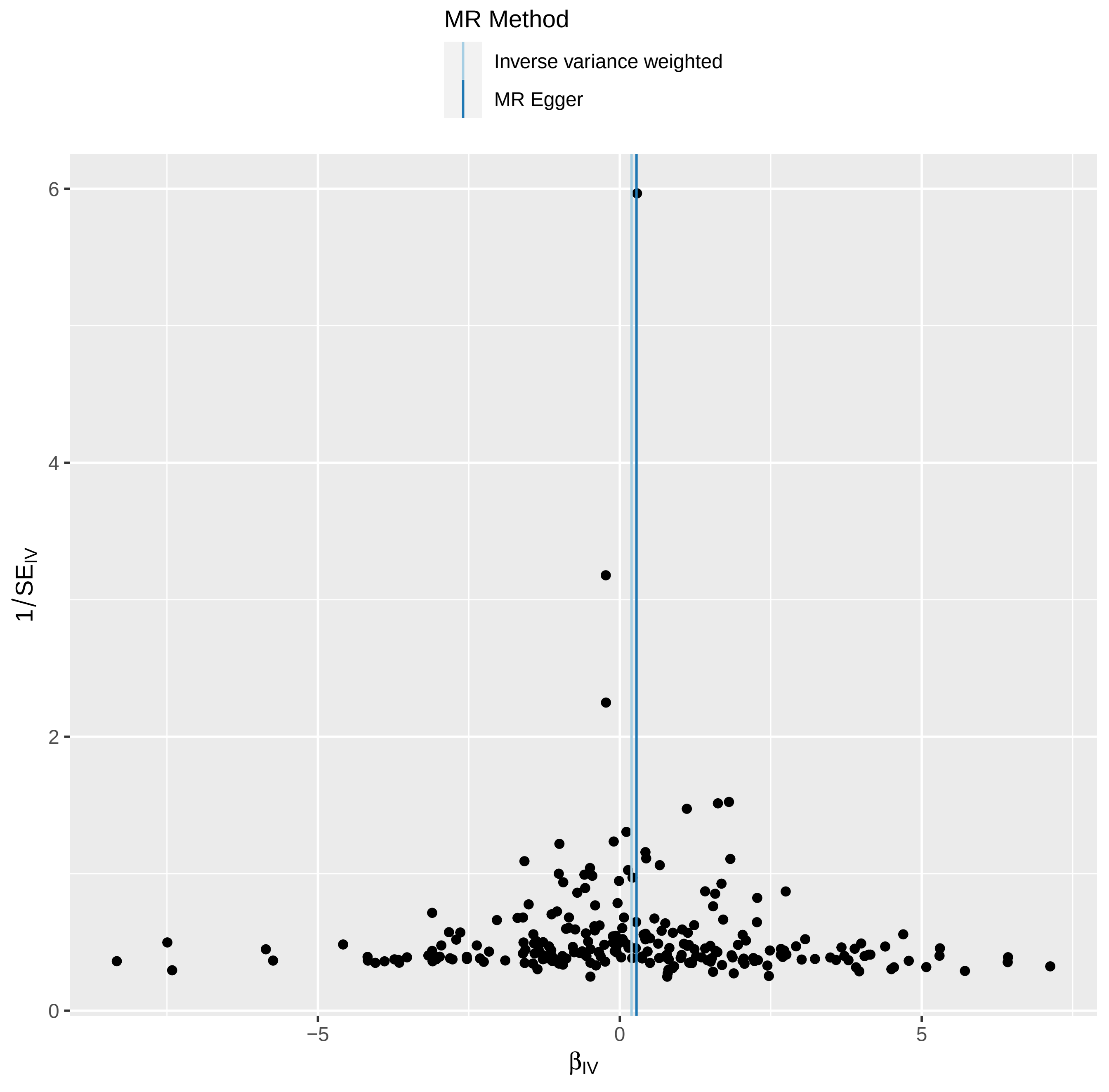

MR Method
Inverse variance weighted
MR Egger
6
4
2
0
−5
0
5
1/SE_IV
β_IV

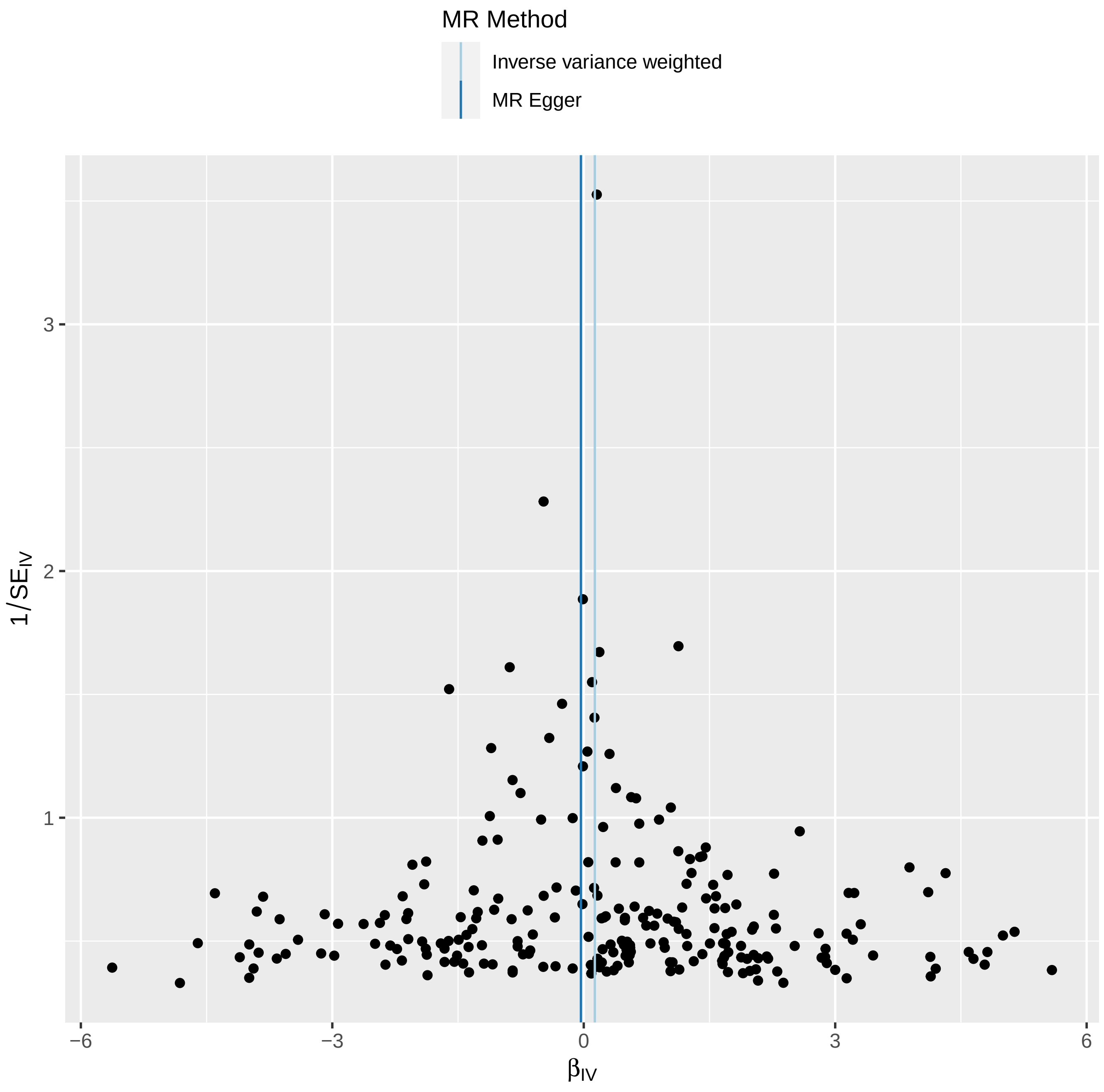

MR Method
Inverse variance weighted
MR Egger
1/SE$_{IV}$
3
2
1
−6
−3
0
3
6
β$_{IV}$

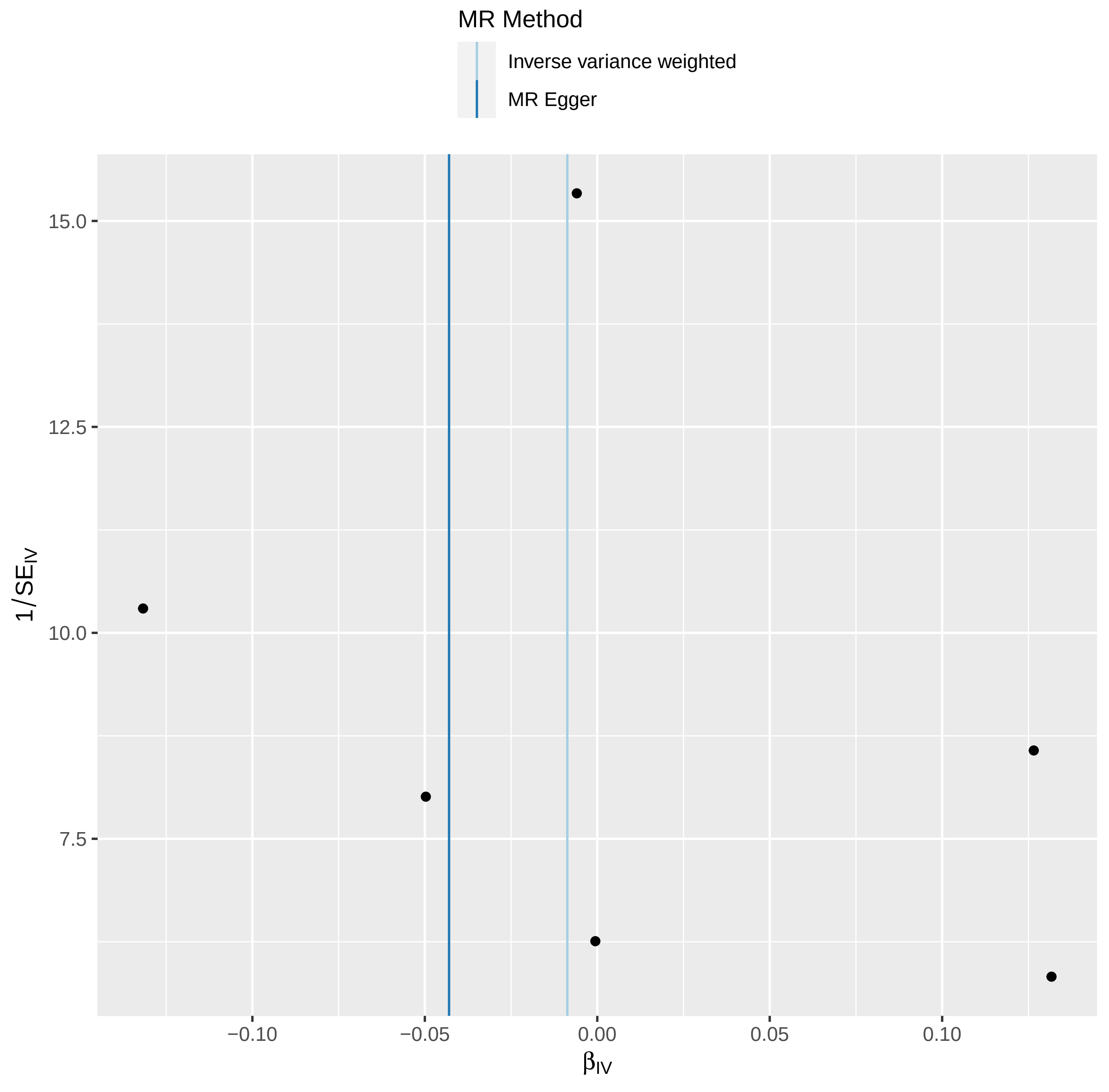

MR Method
Inverse variance weighted
MR Egger
15.0
12.5
10.0
7.5
$1/SE_{IV}$
−0.10
−0.05
0.00
0.05
0.10
β_{IV}

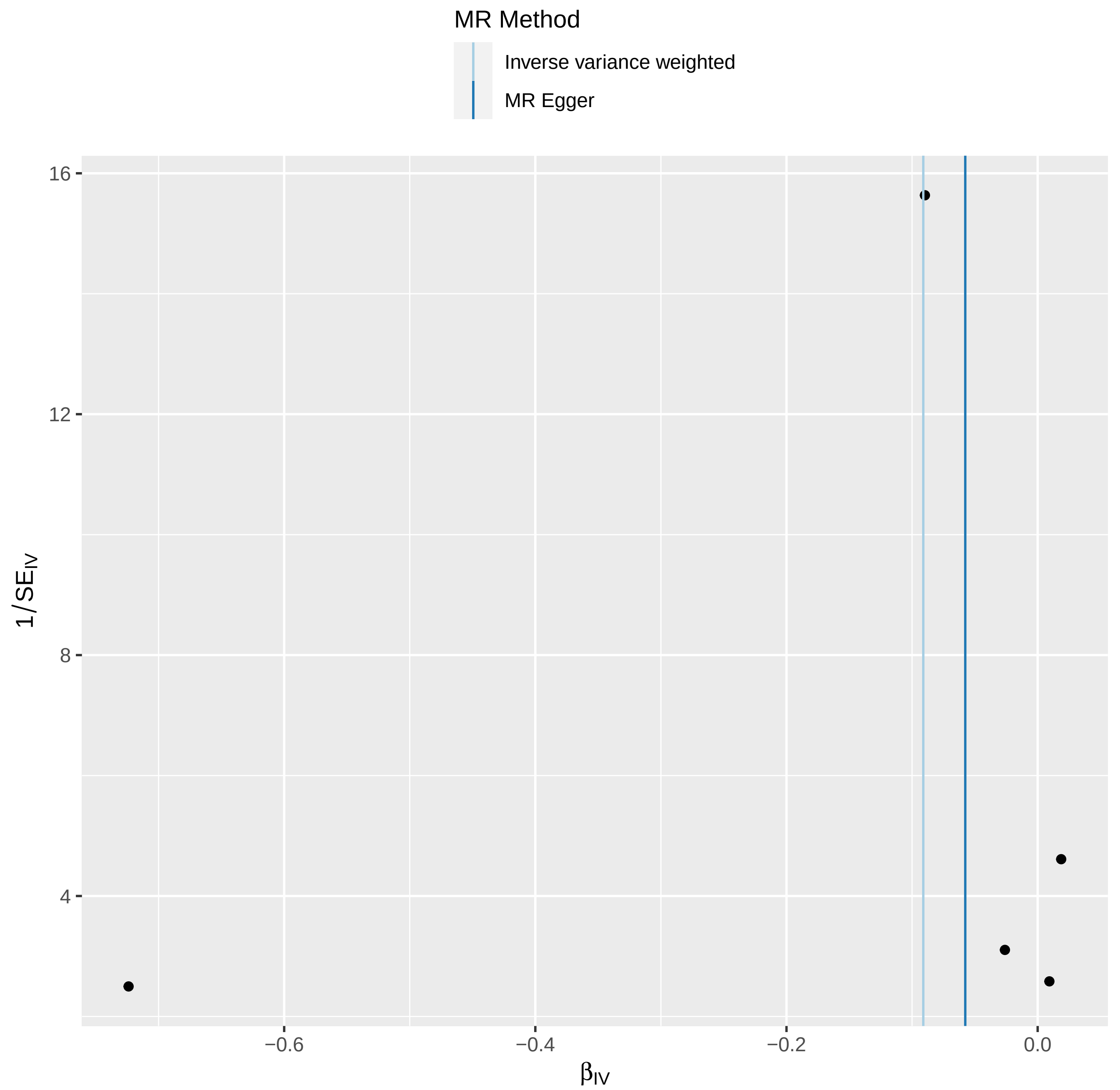

MR Method
Inverse variance weighted
MR Egger
16
12
8
4
1/SE$_{IV}$
−0.6
−0.4
−0.2
0.0
β$_{IV}$

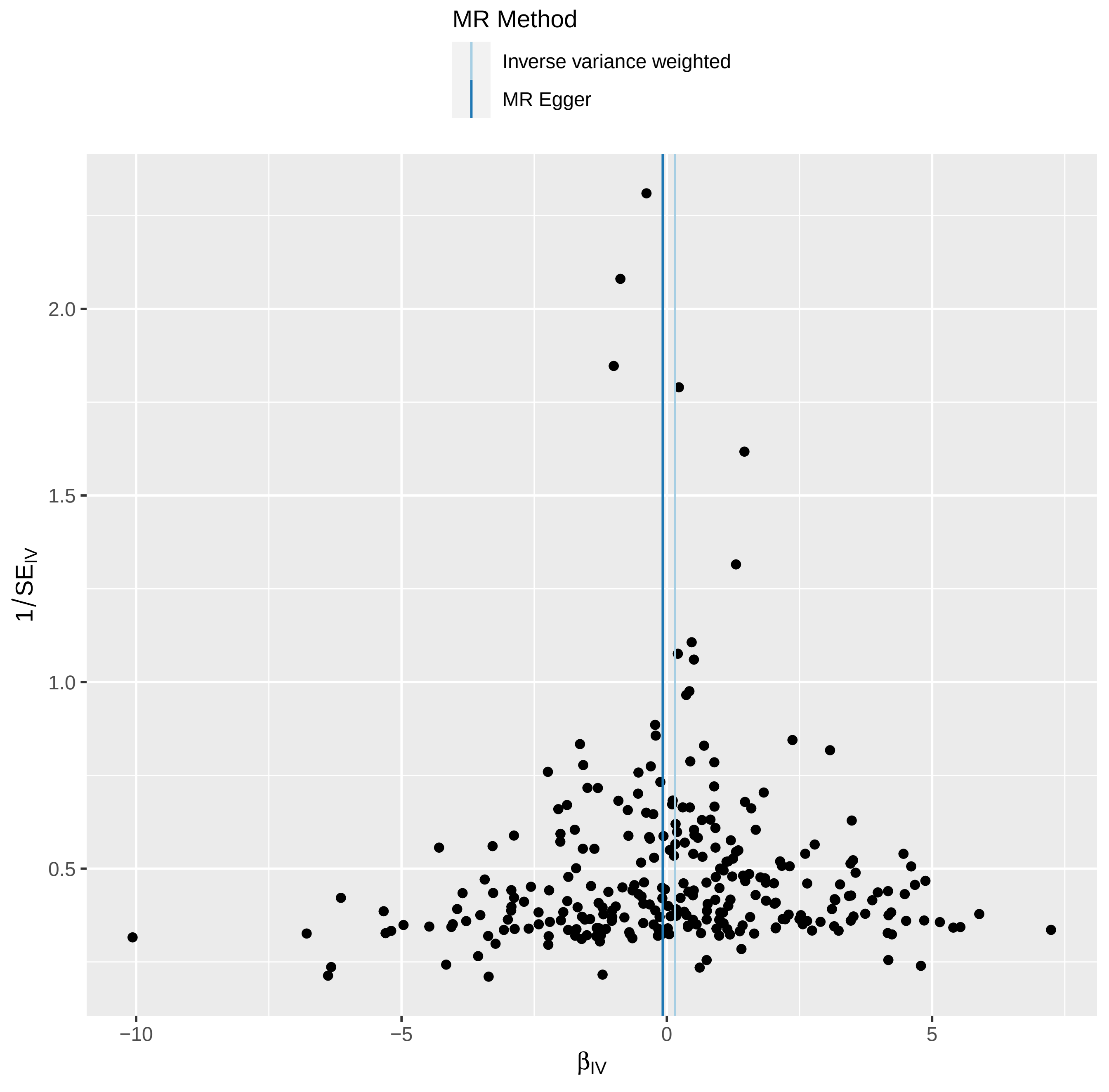

MR Method
Inverse variance weighted
MR Egger
2.0
1.5
1.0
0.5
$1/SE_{IV}$
−10
−5
0
5
β_{IV}

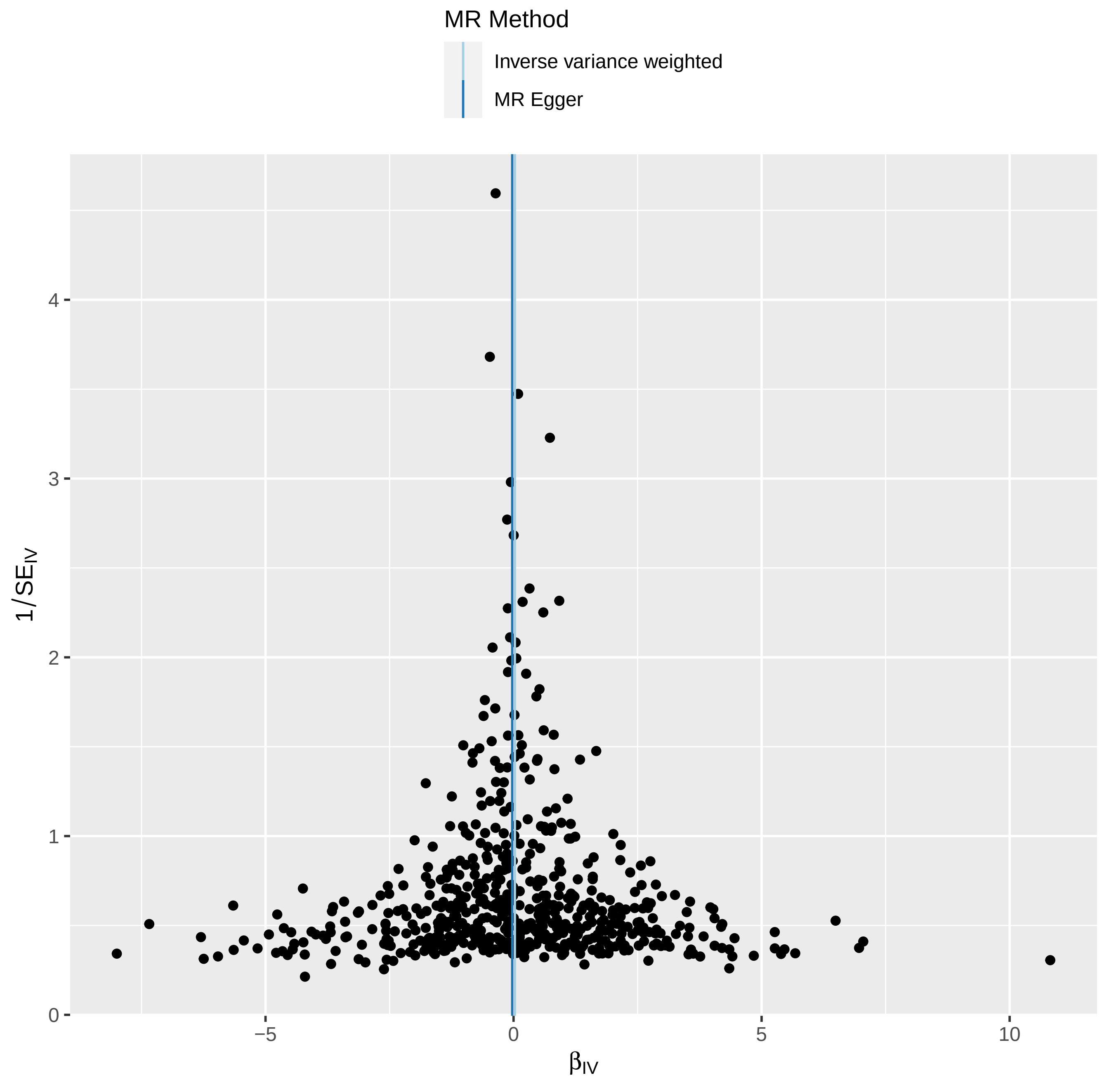

MR Method
Inverse variance weighted
MR Egger
1/SE$_{IV}$
4
3
2
1
0
−5
0
5
10
β$_{IV}$
