# Supplementary material for: Causal pathways in preeclampsia: a Mendelian randomization study in European populations
Source: Front Endocrinol (Lausanne). 2024 Sep 2;15:1453277. doi: 10.3389/fendo.2024.1453277 (PMC11402816; doi:10.3389/fendo.2024.1453277)
Supplement: Supplementary file 6 [file Table2.doc]

**Table S26. The Association of Type 1 diabetes with Preeclampsia risk using five MR methods.**

| **Method** | **Beta** | **SE** | **OR** | **95%CI** | **P Value** |
| --- | --- | --- | --- | --- | --- |
| **IVW** | 0.025 | 0.015 | 1.025 | 0.995-1.056 | 0.107 |
| **MR Egger** | 0.022 | 0.019 | 1.023 | 0.984-1.062 | 0.252 |
| **Weighted median** | 0.013 | 0.018 | 1.013 | 0.978-1.048 | 0.473 |
| **Simple mode** | 0.048 | 0.054 | 1.049 | 0.943-1.166 | 0.380 |
| **Weighted mode** | 0.021 | 0.017 | 1.021 | 0.989-1.055 | 0.206 |

CI, confidence interval; IVW, inverse variance-weighted; MR,

Mendelian Randomization; OR, odds ratio.

**Table S27. The Association of Type 2 diabetes with Preeclampsia risk using five MR methods.**

| **Method** | **Beta** | **SE** | **OR** | **95%CI** | **P Value** |
| --- | --- | --- | --- | --- | --- |
| **IVW** | 0.166 | 0.039 | 1.181 | 1.094-1.275 | 1.899e-05 |
| **MR Egger** | 0.147 | 0.079 | 1.158 | 0.992-1.352 | 6.532e-02 |
| **Weighted median** | 0.195 | 0.063 | 1.215 | 1.074-1.375 | 1.973e-03 |
| **Simple mode** | -0.062 | 0.159 | 0.939 | 0.687-1.284 | 6.956e-01 |
| **Weighted mode** | 0.303 | 0.091 | 1.353 | 1.133-1.617 | 1.060e-03 |

**Table S28. The Association of Gestational diabetes with Preeclampsia risk using five MR methods.**

| **Method** | **Beta** | **SE** | **OR** | **95%CI** | **P Value** |
| --- | --- | --- | --- | --- | --- |
| **IVW** | 0.139 | 0.135 | 1.149 | 0.882-1.498 | 0.304 |
| **MR Egger** | 0.333 | 0.393 | 1.395 | 0.646-3.014 | 0.459 |
| **Weighted median** | 0.108 | 0.078 | 1.114 | 0.956-1.298 | 0.166 |
| **Simple mode** | 0.170 | 0.167 | 1.185 | 0.855-1.643 | 0.365 |
| **Weighted mode** | 0.101 | 0.082 | 1.106 | 0.942-1.299 | 0.284 |

**Table S29. The Association of Hyperthyroidism with Preeclampsia risk using five MR methods.**

| **Method** | **Beta** | **SE** | **OR** | **95%CI** | **P Value** |
| --- | --- | --- | --- | --- | --- |
| **IVW** | 0.127 | 0.051 | 1.135 | 1.027-1.254 | 0.013 |
| **MR Egger** | 0.237 | 0.115 | 1.267 | 1.012-1.587 | 0.078 |
| **Weighted median** | 0.173 | 0.065 | 1.189 | 1.047-1.352 | 7.825e-3 |
| **Simple mode** | 0.160 | 0.102 | 1.173 | 0.961-1.431 | 0.154 |
| **Weighted mode** | 0.186 | 0.080 | 1.204 | 1.030-1.408 | 0.048 |

**Table S30. The Association of Body mass index (BMI) with Preeclampsia risk using five MR methods.**

| **Method** | **Beta** | **SE** | **OR** | **95%CI** | **P Value** |
| --- | --- | --- | --- | --- | --- |
| **IVW** | 0.309 | 0.091 | 1.362 | 1.241-2.235 | 7.066e-04 |
| **MR Egger** | 0.703 | 0.243 | 2.019 | 1.254-3.251 | 4.020e-03 |
| **Weighted median** | 0.510 | 0.150 | 1.666 | 1.242-2.235 | 6.680e-04 |
| **Simple mode** | 0.536 | 0.429 | 1.709 | 0.737-3.963 | 0.212 |
| **Weighted mode** | 0.658 | 0.279 | 1.931 | 1.117-3.337 | 0.019 |

**Table S31. The Association of Total cholesterol levels with Preeclampsia risk using five MR methods.**

| **Method** | **Beta** | **SE** | **OR** | **95%CI** | **P Value** |
| --- | --- | --- | --- | --- | --- |
| **IVW** | -0.008 | 0.080 | 0.992 | 0.848-1.161 | 0.924 |
| **MR Egger** | 0.085 | 0.112 | 1.088 | 0.873-1.357 | 0.452 |
| **Weighted median** | 0.098 | 0.117 | 1.103 | 0.878-1.387 | 0.398 |
| **Simple mode** | 0.092 | 0.257 | 1.096 | 0.662-1.813 | 0.722 |
| **Weighted mode** | 0.055 | 0.099 | 1.057 | 0.871-1.284 | 0.576 |

**Table S32. The Association of Triglycerides with Preeclampsia risk using five MR methods.**

| **Method** | **Beta** | **SE** | **OR** | **95%CI** | **P Value** |
| --- | --- | --- | --- | --- | --- |
| **IVW** | 0.297 | 0.071 | 1.346 | 1.170-1.549 | 3.184e-05 |
| **MR Egger** | 0.129 | 0.105 | 1.138 | 0.926-1.398 | 2.198e-01 |
| **Weighted median** | 0.139 | 0.127 | 1.150 | 0.897-1.473 | 2.703e-01 |
| **Simple**  **mode** | 0.349 | 0.274 | 1.418 | 0.829-2.426 | 2.033e-01 |
| **Weighted mode** | 0.211 | 0.112 | 1.235 | 0.990-1.539 | 6.197e-02 |

**Table S33. The Association of HDL cholesterol levels with Preeclampsia using five MR methods.**

| **Method** | **Beta** | **SE** | **OR** | **95%CI** | **P Value** |
| --- | --- | --- | --- | --- | --- |
| **IVW** | -0.136 | 0.054 | 0.872 | 0.785-0.970 | 0.011 |
| **MR Egger** | -0.105 | 0.075 | 0.900 | 0.777-1.043 | 0.162 |
| **Weighted median** | -0.119 | 0.102 | 0.888 | 0.727-1.083 | 0.240 |
| **Simple mode** | -0.009 | 0.203 | 0.991 | 0.666-1.474 | 0.964 |
| **Weighted mode** | -0.106 | 0.081 | 0.900 | 0.767-1.055 | 0.194 |

**Table S34. The Association of LDL cholesterol levels with Preeclampsia risk using five MR methods.**

| **Method** | **Beta** | **SE** | **OR** | **95%CI** | **P Value** |
| --- | --- | --- | --- | --- | --- |
| **IVW** | 0.118 | 0.055 | 1.125 | 1.009-1.254 | 0.034 |
| **MR Egger** | 0.156 | 0.075 | 1.169 | 1.009-1.354 | 0.038 |
| **Weighted median** | 0.009 | 0.098 | 1.009 | 0.832-1.224 | 0.924 |
| **Simple mode** | 0.019 | 0.216 | 1.019 | 0.667-1.556 | 0.931 |
| **Weighted mode** | 0.088 | 0.079 | 1.092 | 0.936-1.275 | 0.264 |

**Table S35. The Association of Apolipoprotein A1 levels with Preeclampsia risk using five MR methods.**

| **Method** | **Beta** | **SE** | **OR** | **95%CI** | **P Value** |
| --- | --- | --- | --- | --- | --- |
| **IVW** | -0.081 | 0.063 | 0.922 | 0.815-1.043 | 0.195 |
| **MR Egger** | -0.103 | 0.091 | 0.902 | 0.755-1.078 | 0.258 |
| **Weighted median** | -0.151 | 0.106 | 0.860 | 0.699-1.058 | 0.153 |
| **Simple mode** | 0.166 | 0.205 | 1.181 | 0.790-1.767 | 0.418 |
| **Weighted mode** | -0.071 | 0.088 | 0.931 | 0.784-1.106 | 0.420 |

**Table S36. The Association of Apolipoprotein B levels with Preeclampsia risk using five MR methods.**

| **Method** | **Beta** | **SE** | **OR** | **95%CI** | **P Value** |
| --- | --- | --- | --- | --- | --- |
| **IVW** | 0.043 | 0.066 | 1.044 | 0.917-1.189 | 0.517 |
| **MR Egger** | 0.111 | 0.081 | 1.117 | 0.953-1.310 | 0.174 |
| **Weighted median** | 0.127 | 0.089 | 1.136 | 0.955-1.351 | 0.151 |
| **Simple mode** | 0.032 | 0.239 | 1.032 | 0.646-1.649 | 0.895 |
| **Weighted mode** | 0.068 | 0.072 | 1.071 | 0.931-1.231 | 0.342 |

**Table S37. The Association of Systemic lupus erythematosus with Preeclampsia risk using five MR methods.**

| **Method** | **Beta** | **SE** | **OR** | **95%CI** | **P Value** |
| --- | --- | --- | --- | --- | --- |
| **IVW** | 0.108 | 0.052 | 1.114 | 1.005-1.234 | 3.977e-2 |
| **MR Egger** | -0.260 | 0.145 | 0.771 | 0.580-1.025 | 0.172 |
| **Weighted median** | 0.060 | 0.049 | 1.062 | 0.964-1.170 | 0.222 |
| **Simple mode** | 0.025 | 0.077 | 1.025 | 0.881-1.193 | 0.764 |
| **Weighted mode** | 0.037 | 0.060 | 1.038 | 0.923-1.167 | 0.570 |

**Table S38. The Association of Gout with Preeclampsia risk using five MR methods.**

| **Method** | **Beta** | **SE** | **OR** | **95%CI** | **P Value** |
| --- | --- | --- | --- | --- | --- |
| **IVW** | -1.191 | 2.594 | 3.040e-01 | 1.881e-03-49.121 | 0.646 |
| **MR Egger** | -3.618 | 4.102 | 2.685e-02 | 8.646e-06-83.357 | 0.386 |
| **Weighted median** | -2.070 | 2.985 | 1.263e-01 | 3.635e-04-43.886 | 0.488 |
| **Simple mode** | -10.315 | 5.913 | 3.313e-05 | 3.069e-10-3.576 | 0.093 |
| **Weighted mode** | -2.471 | 2.655 | 8.451e-02 | 4.643e-04-15.383 | 0.361 |

**Table S39. The Association of Rheumatoid arthritis with Preeclampsia risk using five MR methods.**

| **Method** | **Beta** | **SE** | **OR** | **95%CI** | **P Value** |
| --- | --- | --- | --- | --- | --- |
| **IVW** | -0.054 | 4.916 | 0.947 | 6.191e-05-1.450e+04 | 0.991 |
| **MR Egger** | -1.236 | 9.154 | 0.291 | 4.690e-09-1.802e+07 | 0.899 |
| **Weighted median** | -1.718 | 5.546 | 0.180 | 3.414e-06-9.436e+03 | 0.757 |
| **Simple mode** | 1.548 | 8.800 | 4.704 | 1.519e-07-1.456e+08 | 0.867 |
| **Weighted mode** | -2.009 | 5.585 | 0.134 | 2.365e-06-7.608e+03 | 0.734 |

**Table S40. The Association of Sleep duration with Preeclampsia risk using five MR methods.**

| **Method** | **Beta** | **SE** | **OR** | **95%CI** | **P Value** |
| --- | --- | --- | --- | --- | --- |
| **IVW** | 0.262 | 0.376 | 1.300 | 0.622-2.717 | 0.485 |
| **MR Egger** | 0.824 | 1.486 | 2.280 | 0.124-41.952 | 0.581 |
| **Weighted median** | 0.538 | 0.552 | 1.713 | 0.581-5.053 | 0.329 |
| **Simple mode** | 1.613 | 1.321 | 5.016 | 0.377-66.744 | 0.226 |
| **Weighted mode** | 0.804 | 0.991 | 2.235 | 0.321-15.584 | 0.420 |

**Table S41. The Association of Bone mineral density with Preeclampsia risk using five MR methods.**

| **Method** | **Beta** | **SE** | **OR** | **95%CI** | **P Value** |
| --- | --- | --- | --- | --- | --- |
| **IVW** | 0.079 | 0.065 | 1.082 | 0.953-1.229 | 0.222 |
| **MR Egger** | 0.062 | 0.121 | 1.064 | 0.839-1.350 | 0.610 |
| **Weighted median** | 0.104 | 0.108 | 1.109 | 0.897-1.372 | 0.338 |
| **Simple mode** | -0.078 | 0.253 | 0.925 | 0.563-1.518 | 0.757 |
| **Weighted mode** | 0.102 | 0.136 | 1.107 | 0.848-1.446 | 0.455 |

**Table S42. The Association of Calcium levels with Preeclampsia risk using five MR methods.**

| **Method** | **Beta** | **SE** | **OR** | **95%CI** | **P Value** |
| --- | --- | --- | --- | --- | --- |
| **IVW** | -0.022 | 0.103 | 0.978 | 0.799-1.198 | 0.833 |
| **MR Egger** | 0.083 | 0.187 | 1.087 | 0.753-1.569 | 0.656 |
| **Weighted median** | -0.273 | 0.158 | 0.761 | 0.558-1.037 | 0.083 |
| **Simple mode** | -0.350 | 0.364 | 0.705 | 0.345-1.437 | 0.337 |
| **Weighted mode** | -0.211 | 0.172 | 0.810 | 0.578-1.135 | 0.222 |

**Table S43. The Association of Serum 25-Hydroxyvitamin D levels with Preeclampsia risk using five MR methods.**

| **Method** | **Beta** | **SE** | **OR** | **95%CI** | **P Value** |
| --- | --- | --- | --- | --- | --- |
| **IVW** | -0.070 | 0.130 | 0.932 | 0.722-1.203 | 0.589 |
| **MR Egger** | -0.085 | 0.203 | 0.919 | 0.617-1.367 | 0.675 |
| **Weighted median** | -0.081 | 0.200 | 0.922 | 0.624-1.364 | 0.685 |
| **Simple mode** | -0.083 | 0.415 | 0.920 | 0.408-2.076 | 0.842 |
| **Weighted mode** | -0.043 | 0.172 | 0.958 | 0.684-1.342 | 0.803 |

**Table S44. The Association of Chronic kidney disease with Preeclampsia risk using five MR methods.**

| **Method** | **Beta** | **SE** | **OR** | **95%CI** | **P Value** |
| --- | --- | --- | --- | --- | --- |
| **IVW** | -0.209 | 0.116 | 0.811 | 0.646-1.019 | 0.072 |
| **MR Egger** | 0.116 | 0.396 | 1.122 | 0.517-2.439 | 0.798 |
| **Weighted median** | -0.172 | 0.136 | 0.842 | 0.645-1.098 | 0.203 |
| **Simple mode** | -0.188 | 0.194 | 0.829 | 0.566-1.212 | 0.404 |
| **Weighted mode** | -0.141 | 0.145 | 0.868 | 0.653-1.155 | 0.404 |

**Table S45. The Association of Serum uric acid levels with Preeclampsia risk using five MR methods.**

| **Method** | **Beta** | **SE** | **OR** | **95%CI** | **P Value** |
| --- | --- | --- | --- | --- | --- |
| **IVW** | 0.195 | 0.096 | 1.215 | 1.007-1.465 | 0.042 |
| **MR Egger** | 0.277 | 0.143 | 1.319 | 0.997-1.745 | 0.054 |
| **Weighted median** | 0.276 | 0.159 | 1.318 | 0.965-1.801 | 0.082 |
| **Simple mode** | -0.134 | 0.423 | 0.875 | 0.382-2.005 | 0.752 |
| **Weighted mode** | 0.128 | 0.141 | 1.136 | 0.861-1.499 | 0.367 |

**Table S46. The Association of Alanine aminotransferase levels with Preeclampsia risk using five MR methods.**

| **Method** | **Beta** | **SE** | **OR** | **95%CI** | **P Value** |
| --- | --- | --- | --- | --- | --- |
| **IVW** | 0.132 | 0.102 | 1.141 | 0.934-1.393 | 0.196 |
| **MR Egger** | -0.034 | 0.188 | 0.967 | 0.669-1.398 | 0.858 |
| **Weighted median** | 0.143 | 0.170 | 1.154 | 0.827-1.610 | 0.400 |
| **Simple mode** | 0.515 | 0.401 | 1.673 | 0.762-3.672 | 0.201 |
| **Weighted mode** | 0.110 | 0.182 | 1.116 | 0.781-1.596 | 0.548 |

**Table S47. The Association of placental growth factor with Preeclampsia risk using five MR methods.**

| **Method** | **Beta** | **SE** | **OR** | **95%CI** | **P Value** |
| --- | --- | --- | --- | --- | --- |
| **IVW** | -0.009 | 0.043 | 0.991 | 0.912-1.078 | 0.838 |
| **MR Egger** | -0.043 | 0.057 | 0.958 | 0.857-1.071 | 0.492 |
| **Weighted median** | -0.009 | 0.055 | 0.991 | 0.890-1.104 | 0.870 |
| **Simple mode** | -0.020 | 0.079 | 0.980 | 0.840-1.145 | 0.813 |
| **Weighted mode** | -0.017 | 0.062 | 0.983 | 0.871-1.109 | 0.792 |

**Table S48. The Association of Vascular endothelial growth factor levels with Preeclampsia risk using five MR methods.**

| **Method** | **Beta** | **SE** | **OR** | **95%CI** | **P Value** |
| --- | --- | --- | --- | --- | --- |
| **IVW** | -0.091 | 0.059 | 0.913 | 0.813-1.025 | 0.122 |
| **MR Egger** | -0.058 | 0.097 | 0.944 | 0.780-1.142 | 0.594 |
| **Weighted median** | -0.082 | 0.061 | 0.921 | 0.817-1.039 | 0.182 |
| **Simple mode** | -0.002 | 0.124 | 0.998 | 0.783-1.272 | 0.986 |
| **Weighted mode** | -0.089 | 0.061 | 0.915 | 0.813-1.030 | 0.217 |

**Table S49. The Association of Haemoglobin concentration with Preeclampsia risk using five MR methods.**

| **Method** | **Beta** | **SE** | **OR** | **95%CI** | **P Value** |
| --- | --- | --- | --- | --- | --- |
| **IVW** | 0.153 | 0.111 | 1.165 | 0.938-1.448 | 0.167 |
| **MR Egger** | -0.078 | 0.221 | 0.925 | 0.600-1.428 | 0.727 |
| **Weighted median** | 0.181 | 0.191 | 1.199 | 0.824-1.744 | 0.343 |
| **Simple mode** | 0.381 | 0.493 | 1.464 | 0.556-3.851 | 0.441 |
| **Weighted mode** | 0.010 | 0.283 | 1.010 | 0.580-1.759 | 0.973 |

**Table S50. The Association of Platelet count with Preeclampsia risk using five MR methods.**

| **Method** | **Beta** | **SE** | **OR** | **95%CI** | **P Value** |
| --- | --- | --- | --- | --- | --- |
| **IVW** | 0.026 | 0.054 | 1.027 | 0.923-1.142 | 0.626 |
| **MR Egger** | -0.027 | 0.092 | 0.973 | 0.813-1.165 | 0.767 |
| **Weighted median** | -0.052 | 0.085 | 0.950 | 0.803-1.122 | 0.544 |
| **Simple mode** | -0.121 | 0.186 | 0.886 | 0.615-1.275 | 0.515 |
| **Weighted mode** | -0.079 | 0.100 | 0.924 | 0.759-1.125 | 0.431 |
